# Applying the Consensus Criteria for Traumatic Encephalopathy Syndrome Retrospectively to Case Studies of Boxers from the 20th Century

**DOI:** 10.1089/neur.2023.0134

**Published:** 2024-04-03

**Authors:** Grant L. Iverson, Andrew J. Gardner, Rudolph J. Castellani, Alicia Kissinger-Knox

**Affiliations:** ^1^ Department of Physical Medicine and Rehabilitation, Spaulding Rehabilitation Hospital, Charlestown, Massachusetts, USA.; ^2^ Department of Physical Medicine and Rehabilitation, Harvard Medical School, Boston, Massachusetts, USA.; ^3^ Department of Physical Medicine and Rehabilitation, Schoen Adams Research Institute at Spaulding Rehabilitation, Charlestown, Massachusetts, USA.; ^4^ Sports Concussion Program, Mass General for Children, Boston, Massachusetts, USA.; ^5^ Home Base, A Red Sox Foundation and Massachusetts General Hospital Program, Charlestown, Massachusetts, USA.; ^6^ Sydney School of Health Sciences, Faculty of Medicine and Health, The University of Sydney, Camperdown, New South Wales, Australia.; ^7^ Department of Pathology, Northwestern University Feinberg School of Medicine, Chicago, Illinois, USA.

**Keywords:** chronic traumatic encephalopathy, concussion, dementia, neurological disorders, sports, traumatic brain injury

## Abstract

There are no validated diagnostic criteria for traumatic encephalopathy syndrome (TES). During the early and middle 20th century, TES was described as a clinical condition that was experienced by some high-exposure boxers—and it was believed to reflect chronic traumatic brain injury. Consensus criteria for the diagnosis of TES were published in 2021. We applied the consensus criteria for TES retrospectively to cases of chronic brain damage in boxers described in articles published in the 20th century that were obtained from narrative and systematic reviews. The sample included 157 boxers identified in 21 articles published between 1929 and 1999. Two authors reviewed each case description and coded the criteria for TES. For the core clinical features, cognitive impairment was noted in 63.1%, and in 28.7% of cases the person's cognitive functioning appeared to be broadly normal. Neurobehavioral dysregulation was present in 25.5%. One third (34.4%) were identified as progressive, 30.6% were not progressive, and the course could not be clearly determined in 35.0%. In total, 29.9% met the TES consensus criteria, 28.0% did not, and 42.0% had insufficient information to make a diagnostic determination. TES, in the 20th century, was described as a neurological condition, not a psychiatric disorder—and this supports the decision of the 2021 consensus group to remove primary and secondary psychiatric diagnoses from being a core diagnostic feature. Future research is needed to determine whether, or the extent to which, cognitive impairment or neurobehavioral dysregulation described as characterizing TES are associated with chronic traumatic encephalopathy neuropathological change.

## Introduction

The clinical manifestations of chronic brain damage in boxers have been documented in the medical literature for generations. Terms like punch drunk,^1^ traumatic encephalopathy,^2^ and dementia pugilistica^3^ were introduced in the first half of the 20th century to describe the clinical condition. Bowman and Blau^4^ used the terms traumatic encephalopathy of pugilists and chronic traumatic encephalopathy (CTE) of pugilists in 1940, and Critchley used the terms CTE^5^ in 1949 and chronic traumatic progressive encephalopathy^6^ in 1957. Roberts used both brain damage and traumatic encephalopathy in the title and subtitle, respectively, of his book published in 1969.^7^ Jordan, in 2000, used the term chronic traumatic brain injury (TBI).^8^

To date, there are no validated clinical diagnostic criteria for CTE or traumatic encephalopathy syndrome (TES). However, there were several attempts in the past decade to create clinical diagnostic criteria, published between 2013 and 2018—and these criteria included a combination of psychiatric and neurological features.^9–13^ In 2019, the World Health Organization adopted the International Classification of Diseases, 11th Edition,^14^ which included “8A00.25 Post traumatic Parkinsonism,” which they noted may result from “major head trauma.” They also noted that it may occur as the result of “multiple blows to the head,” be associated with dementia, and be referred to as CTE.

Preliminary and influential research diagnostic criteria for TES were published in 2014.^11^ The 2014 criteria set out three core and nine supportive diagnostic criteria. These criteria included cognitive impairment and neurological problems, but also diverse psychiatric and psychosocial problems, such as depression, intermittent explosive disorder, anxiety (e.g., obsessive-compulsive disorder and generalized anxiety disorder), excessive gambling, excessive shopping, substance abuse, suicidality, and paranoia. The diverse psychiatric problems that defined the 2014 TES core and supportive diagnostic criteria, however, were not considered part of the clinical syndrome experienced by boxers in the 20th century^15^—and researchers have illustrated the risk of misdiagnosing TES in persons from the general population based on considering the possible psychiatric features.^16–19^

**FIG. 1.**
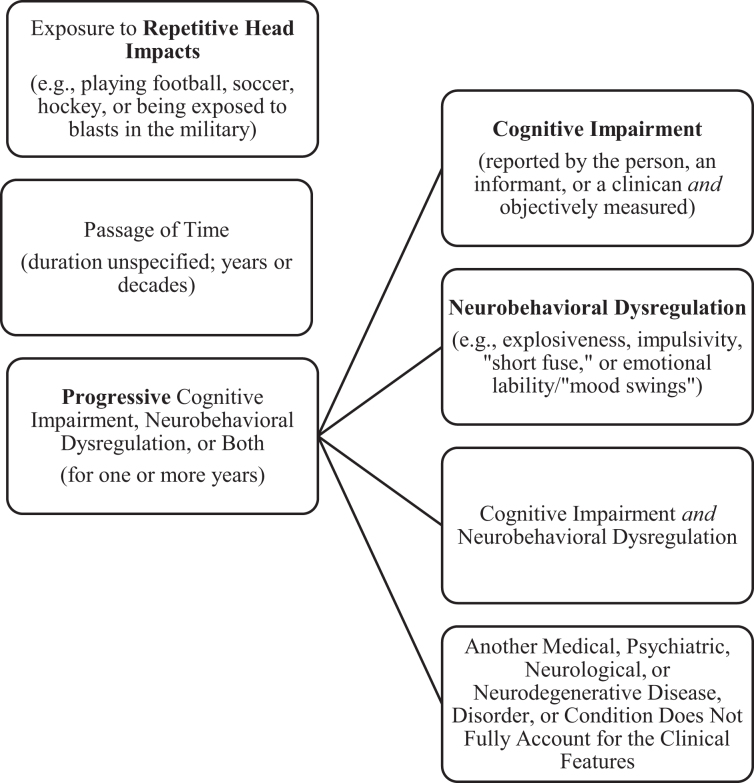
Simplified figure for diagnosing TES based on the new consensus criteria.^20^ Note: The core clinical feature is to have a progressive worsening of cognitive impairment, neurobehavioral dysregulation, or both. If the features depicted in this figure are present, then the next step is to determine the level of functional dependence or dementia, as follows: independent, subtle/mild functional limitation, mild dementia, moderate dementia, or severe dementia. Supportive features are not required for diagnosis; they are used in an algorithm for trying to predict whether CTE neuropathology might be present. The three supportive features are 1) delayed onset of symptoms, 2) motor signs (e.g., a diverse range of parkinsonian signs [e.g., bradykinesia, tremor, or gait disorder], upper motor neuron signs [e.g., spasticity or hyper-reflexia], lower motor neuron signs [e.g., fasciculations and muscle atrophy], and/or amyotrophic lateral sclerosis), and 3) psychiatric features (e.g., a diverse range of psychiatric problems, occurring singly or in combination, that are persistent or progressive, including anxiety disorders, depressive disorders, apathy, and paranoia). This figure was derived from information contained in Tables 1–5 and Figure 1 in the consensus article.^20^ CTE, chronic traumatic encephalopathy; TES, traumatic encephalopathy syndrome.

New consensus criteria for TES, sponsored by research funding from the National Institute of Neurological Disorders and Stroke (NINDS), were published in 2021.^20^ They were developed by a multi-disciplinary group of clinicians and scientists and ultimately agreed upon through a modified Delphi process. The foundation for the criteria were the preliminary 2014 criteria,^11^ but ultimately through the consensus process no parts of the 2014 criteria were retained in their original form.^20^ Importantly, having a psychiatric disorder, such as major depressive disorder, intermittent explosive disorder, generalized anxiety disorder, or obsessive-compulsive disorder, is no longer allowed to meet some of the criteria for TES according to the consensus criteria. Moreover, the clinical condition is now required to be progressive—which was not the case based on the 2014 criteria. Researchers have begun to apply these criteria in clinicopathological case series^21^ and in clinical studies.^22,23^ The consensus criteria are illustrated in Figure 1.

**FIG. 2.**
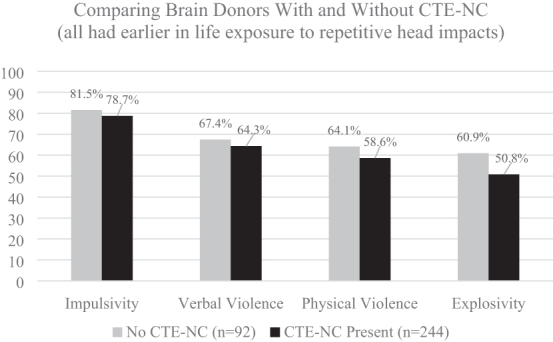
No association between having CTE-NC and neurobehavioral dysregulation features in former athletes.^26^ These data were derived from 336 consecutive brain donors exposed to repetitive head impacts from sports, military service, and/or physical violence, 244 (72.6%) of whom were identified as having CTE-NC and 92 did not have CTE-NC (27.4%).^26^ To create this figure, data were extracted from a table on page 9 of the Supplementary Material for the article by Mez and colleagues: Validity of the 2014 traumatic encephalopathy syndrome criteria for CTE pathology. Alzheimers Dement 2021;17(10):1709-1724; doi: 10.1002/alz.12338. CTE-NC, chronic traumatic encephalopathy neuropathological change.

The authors of the 2021 TES consensus criteria included neurobehavioral dysregulation as one of the core diagnostic features and defined it as follows: “With symptoms and/or observed behaviors representing poor regulation or control of emotions and/or behavior, including (but not limited to) explosiveness, impulsivity, rage, violent outbursts, having a short fuse (exceeding what might be described as periodic episodes of minor irritability), or emotional lability (often reported as mood swings)…” (page 852).^20^ It is questionable, however, whether neurobehavioral dysregulation should be considered part of TES, if TES is assumed to be caused in whole or part by chronic traumatic encephalopathy neuropathological change (CTE-NC),^24,25^ because a large-scale clinicopathological association study did not find an association between several of the features of neurobehavioral dysregulation during life and having the pathology identified after death.^26^ This is illustrated visually in Figure 2, where those brain donors with CTE-NC did not show greater impulsivity, verbal violence, physical violence, or explosivity than brain donors who did not have CTE-NC.

**FIG. 3.**
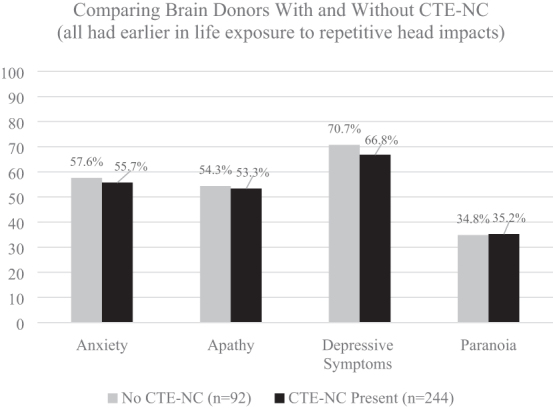
No association between having CTE-NC and the 2021 TES psychiatric features in former athletes.^26^ These data were derived from 336 consecutive brain donors exposed to repetitive head impacts from sports, military service, and/or physical violence, 244 (72.6%) of whom were identified as having CTE-NC and 92 did not have CTE-NC (27.4%).^26^ To create this figure, data were extracted from a table on page 9 of the Supplementary Material for the article by Mez and colleagues: Validity of the 2014 traumatic encephalopathy syndrome criteria for CTE pathology. Alzheimers Dement 2021;17(10):1709–1724; doi: 10.1002/alz.12338. The psychiatric features of TES, according to the 2021 consensus criteria, are as follows^20^: “Anxiety: pervasive worries, excessive fears, agitation, or obsessive or compulsive behavior (or both); a formal diagnosis of anxiety disorder would meet this criterion but is not necessary” (page 856). “Apathy: loss of interest in usual activities and loss of motivation or drive” (page 856). “Depression: feeling overly sad, dysphoric, or hopeless, with or without a history of suicidal thoughts or attempts; a formal diagnosis of major depressive disorder or persistent depressive disorder would meet this criterion but is not necessary” (page 856). “Paranoia: delusional beliefs of suspicion, persecution, or unwarranted jealousy; a formal diagnosis of a psychotic disorder would meet this criterion but is not necessary” (page 856).^20^ CTE-NC, chronic traumatic encephalopathy neuropathological change; TES, traumatic encephalopathy syndrome.

According to the 2021 consensus criteria for TES,^20^ psychiatric problems are considered supportive features. They are not considered in the diagnosis of TES. Instead, researchers and clinicians are encouraged to document four specific psychiatric problems—anxiety, apathy, depression, and paranoia—and if one or more are present, according to the TES consensus criteria, that increases the “provisional levels of certainty” that the person harbors CTE-NC. Similar to neurobehavioral dysregulation (see Fig. 2), this is conceptually and methodologically problematic because the best available evidence suggests that these psychiatric problems are *not*, in fact, associated with CTE-NC. Therefore, their presence should not be used to increase researchers' level of suspicion that the pathology is present in living subjects. The largest study to date on CTE-NC did not find an association between any of these psychiatric features during life and having the pathology identified after death.^26^ This is illustrated visually in Figure 3, where those brain donors with CTE-NC did not show greater anxiety, apathy, depressive symptoms, or paranoia than brain donors who did not have CTE-NC.

The TES consensus criteria were designed to be potentially useful for clinical, epidemiological, risk-factor, neuroimaging, and neuropathology studies, as well as future clinical trials. These criteria were informed by past research, but not empirically validated before publication. As seen in Figures 2 and 3, fundamental aspects of neurobehavioral dysregulation and all the psychiatric features that are included in the criteria for TES are *unrelated* to the neuropathology of CTE. Clearly, foundational research is needed to determine how to revise the TES criteria to make them more useful. The authors of the 2021 consensus criteria^20^ wrote: “the TES criteria will be revised in future NINDS consensus workshops based on updated research on biomarkers, neuropathology, clinical features, and reliability and validity of the new criteria” (page 850). Given that the literature relating to TES is founded upon the case descriptions of ultra-high-exposure boxers from the early and mid-20th century and informed by cases and studies over the past 15 years, it is important to determine whether these high-exposure boxers would meet the modern TES criteria. The purpose of this study is to apply the new 2021 consensus criteria for TES retrospectively to case studies of chronic brain damage in boxers published in the 20th century to determine the extent to which cases met the consensus criteria for TES.

## Methods

We reviewed 26 articles published during the 20^th^ century^1,2,5–7,27–47^ that were identified by authors who have published narrative reviews and systematic reviews on this topic.^10,48,49^ We identified a total of 165 cases, three of which were duplicates, of whom 157 were current or former boxers. A recent review of this same case material, including 155 of these 157 boxers from the 20^th^ century, was focused on whether these men exhibited psychiatric problems, such as depression, anxiety, and suicidality.^15^ That review concluded that TES during the 20^th^ century was described as a neurological condition, including dementia in some cases, and depression, anxiety, and suicidality were not considered to be core clinical features—supporting the decision of the 2021 TES consensus group^20^ removing depression from the 2014 preliminary diagnostic criteria^11^ for TES. For the present study, we extracted information from these same 155 cases (with two additional identified cases) presented in 21 articles^1,2,5–7,27,28,30–32,34,35,37–44,46^ and applied the 2021 TES consensus diagnostic criteria for the three core clinical features (i.e., progressive course, cognitive impairment, and/or neurobehavioral dysregulation), in addition to the supportive features, including whether there was a delayed onset of symptoms, motor signs, or psychiatric features (see the Supplementary Material).

Cognitive impairment on standardized neuropsychological testing is one of the core criteria for diagnosing TES. However, last century traditional neuropsychological testing was rarely done, so documented impairments on standardized tests of memory or executive functioning were not commonly reported. Therefore, we could not apply the 2021 requirement for impairment on standardized testing to most of these cases. For the cases where traditional testing was not conducted, we relied on self, informant, or clinician report of a change in cognitive functioning.

All data used for this study are provided in the Supplementary Material. For some cases, there were differences between the two raters on how they coded core clinical features (e.g., neurobehavioral dysregulation or progressive course), supportive features (e.g., delayed onset of symptoms and problems), or the level of functional independence or dementia. Those differences were resolved through rereview of the source material and discussion. Rater disagreements and resolutions are summarized in Supplementary Table S2. Moreover, after the first round of scientific peer review for this article, we re-examined the original source material with a focus on determining whether some data initially coded as missing (i.e., “not mentioned”) could be coded as not present. We identified some cases that were originally coded as having missing data that could, in fact, be coded as present or absent. This process is described in the Supplementary Material. All information used in this study was derived from previously published articles in the public domain, and this review article is not classified as research involving human subjects.

## Results

The percentages of boxers who met each criterion for TES are depicted in Table 1. Of the total sample, 29.9% met criteria for having TES. Some degree of cognitive impairment was noted in 63.1% of cases, and in approximately one in four (28.7%) cases the person's cognitive functioning appeared to be broadly normal. Impairment in executive functioning could not be determined for most cases. Neurobehavioral dysregulation was present in 25.5%.

Of the 157 cases, 34.4% were considered progressive, 30.6% were not progressive, and the course could not be clearly determined in 35.0%. Progression was often described as worsening of neurological symptoms and problems—but not necessarily progression to severe dementia. Whether the case had a delayed onset was difficult to determine from the case descriptions. In the cases where this information could be extracted, only 8.9% of cases experienced a delay in symptom onset, whereas 38.9% did not. This was not able to be extracted for 52.2% of cases.

**Table 1.** Applying the 2021 Consensus Criteria for TES to 157 Current and Former Boxers

	Present		Not present		Not mentioned, unknown, or presumed not present	
Core features	f	%	*n*	%	f	%
Cognitive or memory impairment	97	61.8	46	29.3	14	8.9
Executive function impairment	34	21.7	33	21.0	90	57.3
Cognitive, memory, or executive function impairment	99	63.1	45	28.7	13	8.3
Neurobehavioral dysregulation	40	25.5	77	49.0	40	25.5
One or more of the core clinical features^a^	104	66.2	44	28.0	9	5.7
Progressive course	54	34.4	48	30.6	55	35.0
Diagnosed traumatic encephalopathy syndrome	47	29.9	44	28.0	66	42.0

Supportive features	f	%	*n*	%	f	%
Depression	17	10.8	52	33.1	88	56.1
Anxiety	7	4.5	48	30.6	102	65.0
Apathy	6	3.8	46	29.3	105	66.9
Paranoia	19	12.1	20	12.7	118	75.2
Suicidality	1^a^	0.6	18	11.5	138	87.9
One or more psychiatric features	35	22.3	29	18.5	93	59.2
Motor signs (e.g., parkinsonism)	88	56.1	48	30.6	21	13.4
Delayed onset of symptoms and problems	14	8.9	61	38.9	82	52.2

	Based on cognitive functioning		Based on cognitive functioning and physical functioning			
Level of functional dependence/dementia	f	%	F	%		
I Independent	36	22.9	32	20.4	—	—
II Subtle/mild functional impairment	52	33.1	52	33.1	—	—
III Mild dementia	35	22.3	36	22.9	—	—
IV Moderate dementia	32	20.4	35	22.3	—	—
V Severe dementia	2	1.3	2	1.3	—	—

Provisional levels of certainty for CTE-NC	f	%				
Missing/unclassifiable	110	70.1	—	—	—	—
Suggestive of CTE-NC	1	0.6	—	—	—	—
Possible CTE-NC	15	9.6	—	—	—	—
Probable CTE-NC	31	19.7	—	—	—	—

The percentages of the current and former boxers meeting each criterion are depicted; *f* = frequency, *n* = sample size, and % = percentage. See the Supplementary Material for details regarding how this information was coded, all the raw data, and a summary for each individual person.

^a^ Includes cognitive impairment, memory impairment, executive functioning impairment, and/or neurobehavioral dysregulation. There was one case that tried to commit suicide in his sixties and he was subsequently admitted to a psychiatric hospital.^30^ Notably, brain tissue from this case was examined by Goldfinger and colleagues,^54^ decades later, and this case did not have evidence of CTE-NC.^24^ The provisional levels of certainty refer to drawing inferences about the likelihood of a person having CTE-NC. “Suggestive” is the lowest threshold, with the least certainty, meaning that the TES criteria are met but the person does not meet additional criteria for possible, probable, or definite CTE-NC, whereas definite means there is post-mortem neuropathological evidence of CTE-NC.

TES, traumatic encephalopathy syndrome; CTE-NC, chronic traumatic encephalopathy neuropathological change.

Supportive psychiatric features extracted from the individual cases included symptoms of depression (10.8%), suicidality (0.6%), anxiety (4.5%), apathy (3.8%), and/or paranoia (12.1%). Having one or more psychiatric features was documented in 22.3%. Motor signs were identified in 56.1% of cases. Fewer than half of the cases had dementia (43.9%).

## Discussion

The 2021 consensus criteria for TES were difficult to apply to cases published in the 20th century. This is because many of the case descriptions lacked details necessary to code some of the features and standardized neuropsychological testing was not administered for most cases last century. That said, we were able to determine the presence or absence of cognitive impairment in more than 90% of cases. Two of three cases (66.2%) met criteria for cognitive impairment, neurobehavioral dysregulation, or both. However, only 28.0% could be classified as clearly meeting all core criteria for TES, primarily because we could not determine whether the person had a progressive course in 35.0% of the cases. It would be reasonable to assume that many of these cases did not have a progressive course, but we did not want to assume that the absence of a documented progressive course meant it was truly absent.

It is apparent from this literature that TES or chronic brain damage in boxers, in the 20th century, was described as a neurological condition, not a psychiatric disorder—and this supports the decision of the TES consensus group to remove psychiatric diagnoses, such as major depressive disorder, intermittent explosive disorder, obsessive-compulsive disorder, and generalized anxiety disorder, from being either a core or supportive *diagnostic* feature of TES.^20^ All of those aforementioned primary psychiatric disorders were considered to be diagnostic features for the preliminary TES criteria published in 2014,^11^ making it very difficult to differentiate or separate primary (or secondary) psychiatric disorders from a diagnosis of presumed TES. Whereas some of the cases from the 20th century clearly had neuropsychiatric problems, such as emotional dyscontrol, personality changes, and paranoia, these problems were almost always described as accompanying frank neurological problems suggestive of chronic TBI, a parkinsonian syndrome, or both. We did not identify any cases that appeared to have primarily a mood (e.g., depression) or anxiety disorder.

### Limitations

We relied on case descriptions from published studies in the 20th century, and by doing so, of course, there were large amounts of missing data. Moreover, our sources for these articles were three narrative or systematic reviews, and some relevant literature from the 20th century might not have been included in any of these reviews. There is the question as to whether the cases with missing data were not reported because the boxers did not exhibit those features. In Mawdsley and Ferguson, for example, the authors wrote: “Details of the general examination and the results of routine investigations are not recorded except in those cases where they are relevant” (p. 7312).^41^ In other articles, it is likely (or certainly possible) that those with missing data were not listed because they did not evidence the feature. To appropriately validate or support the new criteria for TES, the criteria need to be followed exactly, all criteria need to be assessed, and the methods of assessment be clearly defined. Moreover, for all diagnoses, there is clinical judgment, and because of the nature of this study, this could not be applied directly. Given the inclusivity of the TES exposure criteria (i.e., only 5 years of exposure to the sport), all former elite and professional combat, collision, and contact sport athletes who develop mild cognitive impairment or dementia will meet criteria for TES unless another condition can fully account for the cognitive impairment.

Experiencing a stroke, anoxia after cardiac arrest, or a severe TBI are examples of conditions that might rule out the clinical diagnosis of TES because they result in a sudden change in functioning, from normal to impaired. However, those examples are obvious and unlikely to represent a diagnostic challenge. When the change in cognitive functioning is multi-factorial in etiology, and gradual over the course of years, this criterion of “not better accounted for” is much more difficult to apply. The authors of the TES consensus criteria did not offer any guidance for how this differential diagnostic process could be done. From a clinical perspective, there is a major risk for misdiagnosing psychiatric, neurological, and cognitive problems attributable to other causes as being attributable to TES and then by inference attributable to CTE-NC. When the consensus group attempts to revise the TES criteria, it will be important to carefully consider the fact that many features of the current criteria are unrelated to having CTE-NC, as illustrated in Figures 2 and 3.

## Conclusion

There remain important gaps in knowledge relating to TES and CTE-NC, as discussed in critical reviews.^50–52^ CTE-NC is a neuropathological entity^24,25^ and post-mortem diagnosis,^53^ derived from a microscopic examination of brain tissue. Prevalence rates for the neuropathology (CTE-NC) and the consensus clinical diagnosis (TES) in former athletes, military veterans, and persons from the general population are unknown. The consensus criteria for TES require “substantial exposure” to repetitive head impacts and define this as at least 5 years of participation in amateur contact or collision sports, with at least 2 of those years being at the high school level. As written, this means that nearly the entire population of persons who played 4 years of sports in high school, all collegiate athletes, and all professional athletes will likely meet this low threshold for exposure.

From a practical perspective, all former athletes (high school, college, and professional) meeting the exposure criteria who develop mild cognitive impairment or dementia will, by definition, meet criteria for having TES unless the cognitive impairment is deemed to be “fully accounted for by other disorders”—and this distinction will be an important focus for researchers. Of course, this work must carefully consider equifinality—the principle that an end state, such as cognitive impairment, can be reached by many different pathways and trajectories. Directions for future research are provided in Table 2. Future research is needed to determine whether, and the extent to which, the emergence, course, or severity of cognitive impairment, neurobehavioral dysregulation, or both are caused directly or indirectly by CTE-NC.

**Table 2.** Directions for Future Research

1. Determine clear exposure thresholds for repetitive head impacts associated with sports other than football (e.g., hockey, soccer, and rugby) and for repetitive low-level blast exposures during military service. However, any single threshold requirement will likely carry associated risks of over- and underidentification if uniformly applied without other person-specific considerations.
2. Evaluate the inter-rater reliability of the consensus diagnostic criteria for TES.
3. Examine the prevalence of TES clinical features in the general population and in subgroups of persons and patients with clinical conditions, with and without the repetitive head-hits exposure criterion.
4. Conduct specificity studies to determine how often clinical criteria are met in persons who have not had exposure to repetitive neurotrauma.
5. Determine whether neurobehavioral dysregulation believed to be associated with TES in former athletes is different in any way from the neurobehavioral dysregulation that is observed in persons with the mild behavioral impairment^55,56^ that is associated with mild cognitive impairment,^57^ Alzheimer's disease,^58^ Parkinson's disease,^59^ or frontotemporal dementia^60^ from the general population.
6. Determine whether neurobehavioral dysregulation associated with TES can be clearly differentiated from worsening of longstanding intermittent explosive disorder^61^ or anger attacks that are associated with major depressive disorder^62–64^ or anxiety disorders.^65,66^
7. Evaluate the reliability and validity of clinician/researcher ratings of levels of functional dependence and dementia.
8. Develop methods and decision rules for examining and applying the criterion “not better accounted for” by another clinical (or neuropathological) diagnosis or condition.
9. Conduct longitudinal case-series, case-control, and cohort studies of those with and without antemortem TES diagnoses and post-mortem CTE-NC diagnoses.
10. Determine whether CTE-NC directly correlates or is causally related to specific symptoms or problems. The literature to date indicates that it does not correlate with features of neurobehavioral dysregulation, depression, suicidality, anxiety, apathy, or paranoia.

TES, traumatic encephalopathy syndrome; CTE-NC, chronic traumatic encephalopathy neuropathological change.

## Supplementary Material

Supplemental data

## Transparency, Rigor, and Reproducibility Summary

The original contributions presented in the study are included in the article and in the Supplementary Material. The online supplement includes a tabular representation of the data used in this review and extractions of quotes from the published articles relating to the clinical features of boxers who had chronic TBI and/or TES. This review and the analysis plan were not pre-registered. The presentation of the case series was primarily descriptive. Two authors rated the 157 cases on the TES consensus criteria and disagreements were resolved through discussion, as documented in the Supplementary Material. All information used in this study was derived from previously published articles in the public domain, and this review article is not classified as research involving human subjects. This article will be published under a Creative Commons Open Access license and, upon publication, will be freely available.

## Abbreviations Used

CTE: chronic traumatic encephalopathy
CTE-NC: chronic traumatic encephalopathy neuropathological change.
NINDS: National Institute of Neurological Disorders and Stroke
TBI: traumatic brain injury
TES: traumatic encephalopathy syndrome
