# Supplementary material for: Applying the Consensus Criteria for Traumatic Encephalopathy Syndrome Retrospectively to Case Studies of Boxers from the 20th Century
Source: Neurotrauma Rep. 2024 Apr 3;5(1):337–47. doi: 10.1089/neur.2023.0134 (PMC11002329; doi:10.1089/neur.2023.0134)
Supplement: Supplemental data [file Suppl_Data.pdf]

Applying the Consensus Criteria for Traumatic Encephalopathy Syndrome Retrospectively to Case Studies of Boxers from the 20th Century

Grant L. Iverson, Ph.D.[1,2,3,4,5*]
Andrew J. Gardner, Ph.D.[6]
Rudolph J. Castellani, M.D.[7,8]
Alicia Kissinger-Knox, Psy.D.[1,2,4]

[1]Department of Physical Medicine and Rehabilitation, Spaulding Rehabilitation Hospital, Charlestown, MA, USA
[2]Department of Physical Medicine and Rehabilitation, Harvard Medical School, Boston, MA, USA
[3]Department of Physical Medicine and Rehabilitation, Schoen Adams Research Institute at Spaulding Rehabilitation, Charlestown, MA, USA
[4]Sports Concussion Program, Mass General for Children, Boston, MA, USA
[5]Home Base, A Red Sox Foundation and Massachusetts General Hospital Program, Charlestown, MA, USA
[6]Sydney School of Health Sciences, Faculty of Medicine and Health, The University of Sydney, Camperdown, NSW, Australia
[7]Department of Pathology, Northwestern University Feinberg School of Medicine, Chicago, Illinois, USA
[8]Mesulam Center for Cognitive Neurology and Alzheimer's Disease, Northwestern University Feinberg School of Medicine, Chicago, Illinois, USA

The articles from the 20th century were reviewed by two authors independently (AKK and AJG) and each case was coded based on the 2021 Consensus Criteria for Traumatic Encephalopathy Syndrome. The results of that coding are presented in Tables 1 and 2.

The article extractions of text supporting each rating for each case were done by one author (AKK) with supplementation from a second author (AJG). Two other authors (GLI and RJC) reviewed codings for some of the cases to try to further determine whether some items coded as being 'not mentioned' could be coded as 'no or not present.'

After the first round of scientific peer review, we (AKK and GLI) re-examined some of the articles to determine whether we could code some variables that were originally coded as 'not mentioned' as 'not present.' That process is described in the data coding section of this supplement.

Table of Contents

Table 1. Coding the TES Consensus Criteria for Cases from the 20th Century 3
Table 2. Description of Rater Disagreements and Resolution (Rater Agreement) 7
Table 3. Coding the TES Consensus Criteria for Cases from the 20th Century for Statistical Analyses 10
Table 4. Coding the TES Consensus Criteria for Final 157 Cases from the 20th Century for Statistical Analyses 14
Statistical Analyses: Frequencies 18
Article Extractions 21
- Martland (1928) 21
- Parker (1934) 22
- Critchley (1949) 28
- Raevuori-Nallinma (1950) 35
- Critchley (1957) 37
- Corsellis & Brierley (1959) 44
- Neubuerger, Sinton, & Denst (1959) 47
- Courville (1962) 49
- Spillane (1962) 50
- Mawdsley & Ferguson (1963) 56
- Payne (1968) 66
- Johnson (1969) 72
- Roberts (1969) 92
- Corsellis, Bruton, and Freeman-Browne (1973) 105
- Harvey & Newsom Davis (1974) 121
- Kaste et al. (1982) 122
- Casson et al. (1984) 125
- Roberts, Whitwell, Acland, & Bruton (1990) 143
- Hof, Knabe, Bovier, & Bouras (1991) 144
- Hof et al. (1992) 145
- Jordan et al. (1995) 146
- Geddes et al. (1996) 147
- Williams & Tannenberg (1996) 148
- Jordan et al. (1997) 149
- Geddes et al. (1999) 179
- Newell & Drachman (1999) 183

Quotes from the Literature Relating to the Course of the Clinical Condition 185
Cases Included in Other Review Papers 192

Table 1. Coding the TES Consensus Criteria for Cases from the 20th Century

Table Key
0 = Absent (e.g., authors report no change in cognition, psychiatric symptoms, or motor signs)
1 = Present
2 = Unknown (typically the article addressed the topic, but it is unknown if this was a "change," (i.e., when a boxer was described as aggressive throughout his life, if a test was not administered so it is unknown if the boxer would have performed poorly on it, or if there was conflicting information in the clinical evaluation)
NM = Not mentioned. The topic was not mentioned in that case.
NA = Not applicable. There are 5 instances of “Not Applicable” for delayed onset, that was due to the patient either not having a boxing career or a duplicate case where the boxer died during his last boxing match.

Cases Excluded from Analyses: There are 165 cases, although some are duplicates and some were not boxers. The two cases from Corsellis and Brierley (1959) (case #21 and 22) were also included in Corsellis et al. (1973), and in the 1973 article more clinical details were provided. The cases from 1959 were not included in the statistical analyses. The single case presented in Geddes et al. (1996) (#129) was also presented as the first case in Geddes et al. (1999) (#161), and only the case from the 1999 article was included in the statistical analyses. Case #4 in Geddes et al. (1999) (case #164) was not a boxer (he was described as having mental retardation and an intractable seizure disorder). There were two women, one who experienced prolonged interpersonal violence (case #123) and one who had autism and severe head banging behavior (case #124). There was also a man with autism and severe head banging behavior described in the literature (case #163). There was also a case described as an ‘achondroplastic dwarf’ who was a circus clown with severe alcoholism and many head injuries (case #130). These eight aforementioned cases are included in this supplement, but they are not included in the statistical analyses or the main article. The final sample for the statistical analyses included 157 current and former boxers. Johnson (1969) states that 10 cases were already reported by Mawdsley and Ferguson (1963) and were assessed 4-5 years later. However, he does not state which 10 cases these are. Therefore, we assume that some of these cases are not unique cases—they are follow-up cases from Mawdsley and Ferguson. Therefore, in summary, case numbers #21, 22, 123, 124, 129, 130, 163, and 164 were not included in the statistical analyses (i.e., Table 3).

Reference	Case	Case #	Cognitive Impairment	Executive Function Impairment	Neurobehavioral Dysregulation	Progressive Course	Depression	Anxiety	Apathy	Paranoia	Suicidality	Motor Signs	Delayed Onset	Cognitive Only	Also Includes Physical (Motor) Disability
Martland, 1928	2	1	0	NM	NM	Yes	NM	NM	NM	NM	NM	1	0	II	II
Parker, 1934	I	2	1	NM	NM	No	NM	1	NM	NM	NM	1	0	II	II
	II	3	1	NM	NM	No	NM	NM	NM	NM	NM	1	0	II	III
	III	4	1	NM	NM	Unclear	NM	NM	NM	NM	NM	1	0	II	III
Critchley, 1949	A	5	NM	NM	NM	Unclear	NM	NM	NM	NM	NM	2	NM	II	II
	B	6	1	1	NM	Unclear	NM	NM	NM	NM	NM	2	NM	IV	IV
	C	7	1	NM	1	Unclear	NM	1	NM	NM	NM	1	0	III	III
	D	8	1	NM	1	Unclear	1	NM	NM	NM	NM	NM	NM	III	III
	E	9	1	NM	1	Unclear	0	NM	NM	NM	NM	1	NM	III	III
	F	10	1	NM	NM	Unclear	NM	NM	NM	NM	NM	1	NM	III	III
	G	11	NM	NM	NM	Unclear	NM	2	NM	NM	NM	NM	NM	II	II
Raevuori-Nallinma, 1950	1	12	1	NM	0	No	NM	1	NM	NM	NM	2	NM	II	II
	2	13	1	NM	NM	Yes	NM	NM	1	NM	NM	1	NM	IV	IV
Critchley, 1957	2	14	1	NM	NM	Yes	NM	NM	NM	NM	NM	1	0	II	II
	3	15	1	NM	NM	Yes	0	NM	NM	NM	NM	NM	NM	II	II
	8	16	1	NM	NM	Yes	NM	NM	NM	NM	NM	1	0	II	II
	9	17	1	NM	NM	Yes	NM	NM	NM	NM	NM	1	NM	II	II
	10	18	NM	NM	1	Yes	NM	NM	NM	NM	NM	1	NM	II	II
	11	19	1	1	1	Yes	NM	NM	NM	NM	NM	1	NM	II	II
	14	20	1	NM	0	Unclear	NM	NM	NM	NM	NM	2	NM	II	II
Corsellis & Brierley, 1959	1	21	1	1	1	Yes	NM	NM	NM	1	NM	2	NM	IV	IV
	2	22	1	NM	1	Yes	NM	NM	NM	1	NM	1	NM	V	V
Neubuerger, Sinton, & Denst, 1959	1	23	1	0	1	Yes	NM	1	NM	1	NM	1	1	III	III

Reference	Case	Case #	Cognitive Impairment	Executive Function Impairment	Neurobehavioral Dysregulation	Progressive Course	Depression	Anxiety	Apathy	Paranoia	Suicidality	Motor Signs	Delayed Onset	Cognitive Only	Also Includes Physical (Motor) Disability
	2	24	1	NM	0	Yes	NM	1	NM	NM	NM	1	1	IV	IV
Courville, 1962	1	25	1	NM	1	Yes	NM	NM	NM	NM	NM	1	NM	IV	IV
Spillane, 1962	1	26	0	NM	0	Yes	0	NM	NM	NM	NM	1	0	I	II
	2	27	1	NM	NM	Yes	1	1	NM	NM	NM	1	NM	IV	IV
	3	28	1	1	NM	Yes	0	NM	NM	NM	NM	1	1	IV	IV
	4	29	1	NM	1	Unclear	NM	NM	NM	NM	NM	0	NM	II	II
	5	30	0	NM	NM	Yes	1	NM	NM	NM	NM	1	0	II	II
Mawdsley & Ferguson, 1963	1	31	1	1	1	Yes	NM	NM	NM	NM	NM	1	0	IV	IV
	2	32	1	NM	NM	Yes	NM	NM	NM	NM	NM	1	0	IV	IV
	3	33	1	NM	1	Yes	1	NM	NM	NM	NM	0	1	IV	IV
	4	34	1	NM	1	Unclear	NM	NM	NM	1	NM	1	1	IV	IV
	5	35	1	NM	1	Yes	1	NM	NM	1	NM	1	0	IV	IV
	6	36	1	NM	NM	Yes	NM	NM	NM	NM	NM	1	1	III	III
	7	37	1	NM	NM	Yes	NM	NM	NM	NM	NM	1	1	III	III
	8	38	1	NM	1	Unclear	NM	NM	NM	NM	NM	1	NM	IV	IV
	9	39	1	NM	NM	Yes	NM	NM	NM	NM	NM	1	0	III	III
	10	40	1	NM	0	Yes	NM	NM	1	NM	NM	1	0	IV	IV
Payne, 1968	1	41	NM	NM	NM	Unclear	1	NM	NM	NM	NM	NM	NM	III	III
	2	42	NM	NM	1	Yes	1	NM	NM	1	NM	1	0	III	III
	3	43	1	1	1	Unclear	1	NM	NM	NM	NM	1	NM	III	III
	4	44	0	NM	1	Unclear	1	NM	NM	NM	NM	1	NM	I	III
	5	45	NM	NM	NM	Unclear	NM	NM	NM	NM	NM	2	NM	I	II
	6	46	NM	NM	NM	Unclear	NM	NM	NM	NM	NM	NM	NM	II	II
Johnson, 1969	1	47	1	1	1	Yes	NM	NM	1	1	NM	1	0	IV	IV
	2	48	1	NM	0	Yes	NM	NM	NM	NM	NM	1	NM	III	III
	3	49	1	NM	0	No	NM	NM	NM	NM	NM	1	NM	III	III
	4	50	0	0	0	No	0	1	NM	0	NM	0	0	I	I
	5	51	1	NM	0	No	NM	NM	NM	NM	NM	0	NM	II	II
	6	52	1	NM	1	No	NM	NM	NM	1	NM	1	0	III	III
	7	53	1	NM	0	No	NM	NM	NM	NM	NM	1	NM	II	II
	8	54	1	NM	1	Yes	1	NM	NM	1	NM	1	NM	III	III
	9	55	1	NM	1	No	NM	NM	NM	1	NM	1	NM	III	III
	10	56	1	NM	1	No	NM	NM	NM	1	NM	1	NM	III	III
	11	57	1	NM	0	Yes	NM	NM	NM	NM	NM	1	NM	III	III
	12	58	0	NM	0	Yes	NM	NM	NM	0	NM	1	NM	II	III
	13	59	1	NM	0	No	NM	NM	NM	NM	NM	1	NM	III	III
	14	60	0	NM	0	No	NM	NM	NM	NM	NM	1	NM	I	II
	15	61	1	NM	0	Yes	NM	NM	NM	NM	NM	1	NM	III	III
	16	62	1	1	0	Yes	NM	NM	NM	NM	NM	1	NM	IV	IV
	17	63	1	1	1	Yes	NM	NM	1	1	NM	1	1	V	V
Roberts, 1969	1	64	1	1	NM	Yes	NM	NM	NM	1	NM	1	0	IV	IV
	2	65	0	NM	1	Yes	NM	NM	NM	NM	NM	1	0	III	III
	3	66	2	NM	NM	Yes	NM	NM	NM	NM	NM	1	1	III	III
	4	67	0	NM	0	Yes	NM	NM	NM	NM	NM	1	NM	II	II
	5	68	1	NM	1	Unclear	1	NM	1	NM	NM	1	NM	IV	IV
	6	69	1	NM	1	Yes	NM	NM	NM	1	NM	1	0	IV	IV
	7	70	0	NM	NM	Yes	NM	NM	NM	NM	NM	1	NM	II	II
	8	71	0	NM	0	No	NM	NM	NM	NM	NM	1	NM	I	I
	9	72	0	NM	NM	Unclear	NM	NM	NM	NM	NM	1	0	I	I
	10	73	0	NM	0	No	NM	NM	NM	NM	NM	1	0	II	II
	11	74	1	NM	NM	No	NM	NM	NM	NM	NM	2	0	III	III
Corsellis, Bruton, & Freeman-Browne, 1973	1	75	1	1	1	Yes	NM	NM	NM	NM	NM	2	NM	IV	IV

Reference	Case	Case #	Cognitive Impairment	Executive Function Impairment	Neurobehavioral Dysregulation	Progressive Course	Depression	Anxiety	Apathy	Paranoia	Suicidality	Motor Signs	Delayed Onset	Cognitive Only	Also Includes Physical (Motor) Disability
	2	76	1	1	1	Yes	NM	NM	NM	NM	NM	1	0	IV	IV
	3	77	1	NM	1	Yes	NM	NM	NM	1	NM	1	0	IV	IV
	4	78	1	NM	NM	Yes	NM	NM	NM	NM	NM	1	0	IV	IV
	5	79	1	NM	1	Yes	NM	NM	NM	NM	NM	2	0	IV	IV
	6	80	1	NM	1	Yes	NM	NM	NM	1	NM	1	0	III	III
	7	81	1	1	1	Yes	NM	NM	NM	NM	NM	1	NM	IV	IV
	8	82	1	NM	1	Yes	NM	NM	NM	NM	NM	1	1	IV	IV
	9	83	1	NM	NM	Yes	NM	NM	NM	NM	1	1	NM	IV	IV
	10	84	1	NM	1	Yes	NM	NM	NM	1	NM	1	NM	V	V
	11	85	1	NM	1	Unclear	NM	NM	NM	NM	NM	NM	1	IV	IV
	12	86	0	NM	1	No	NM	NM	NM	NM	NM	NM	NM	II	II
	13	87	1	1	1	Yes	NM	NM	NM	1	NM	2	NM	IV	IV
	14	88	0	NM	NM	No	NM	NM	NM	NM	NM	0	NM	II	II
	15	89	NM	NM	NM	No	NM	NM	NM	NM	NM	0	NM	II	II
Harvey & Newsom Davis, 1974	1	90	1	NM	1	Yes	1	NM	NM	1	NM	1	0	IV	IV
Kaste et al., 1982	1	91	1	1	2	Unclear	NM	NM	NM	NM	NM	1	NM	III	III
	2	92	1	1	2	Unclear	NM	NM	NM	NM	NM	0	NM	III	III
	3	93	0	0	0	No	NM	NM	NM	NM	NM	0	0	I	I
	4	94	0	0	0	No	NM	NM	NM	NM	NM	0	0	I	I
	5	95	0	0	0	No	NM	NM	NM	NM	NM	0	0	I	I
	6	96	0	0	0	No	NM	NM	NM	NM	NM	0	0	I	I
	7	97	0	0	0	No	NM	NM	NM	NM	NM	0	0	I	I
	8	98	0	0	0	No	NM	NM	NM	NM	NM	0	0	I	I
	9	99	0	0	0	No	NM	NM	NM	NM	NM	0	0	I	I
	10	100	0	0	0	No	NM	NM	NM	NM	NM	0	0	I	I
	11	101	0	0	0	No	NM	NM	NM	NM	NM	0	0	I	I
	12	102	0	0	0	No	NM	NM	NM	NM	NM	0	0	I	I
	13	103	0	0	0	No	NM	NM	NM	NM	NM	0	0	I	I
	14	104	0	0	0	No	NM	NM	NM	NM	NM	0	0	I	I
Casson et al., 1984	1	105	1	0	0	Unclear	0	0	0	0	0	0	NM	II	II
	2	106	1	1	0	Unclear	0	0	0	0	0	0	NM	II	II
	3	107	1	0	0	Unclear	0	0	0	0	0	0	NM	I	I
	4	108	1	0	0	Unclear	0	0	0	0	0	0	NM	II	II
	5	109	1	1	0	Unclear	0	0	0	0	0	0	NM	II	II
	6	110	1	0	0	Unclear	0	0	0	0	0	0	NM	II	II
	7	111	1	1	0	Unclear	0	0	0	0	0	1	NM	II	II
	8	112	1	1	0	Unclear	0	0	0	0	0	0	NM	II	II
	9	113	1	1	0	Unclear	0	0	0	0	0	0	NM	II	II
	10	114	1	1	0	Unclear	0	0	0	0	0	1	NM	II	II
	11	115	1	1	0	Unclear	0	0	0	0	0	0	NM	II	II
	12	116	1	1	0	Unclear	0	0	0	0	0	0	NM	II	II
	13	117	1	1	0	Unclear	0	0	0	0	0	0	NM	II	II
	14	118	1	1	0	Unclear	0	0	0	0	0	0	0	II	II
	15	119	1	0	0	Unclear	0	0	0	0	0	0	0	II	II
	16	120	0	2	0	Unclear	0	0	0	0	0	0	0	I	I
	17	121	0	2	0	Unclear	0	0	0	0	0	0	0	I	I
	18	122	0	2	0	Unclear	0	0	0	0	0	0	0	I	I
Roberts et al., 1990	1	123	1	NM	NM	Unclear	NM	NM	NM	NM	NM	2	NA	IV	IV
Hof et al., 1991	1	124	2	NM	NM	Yes	NM	NM	NM	NM	NM	1	NM	II	IV
Hof et al., 1992	1	125	1	1	NM	Unclear	1	NM	NM	NM	NM	1	NM	III	IV
	2	126	1	1	NM	Unclear	1	NM	NM	NM	NM	1	NM	III	IV
	3	127	1	1	NM	Unclear	1	NM	NM	NM	NM	1	NM	III	IV

Reference	Case	Case #	Cognitive Impairment	Executive Function Impairment	Neurobehavioral Dysregulation	Progressive Course	Depression	Anxiety	Apathy	Paranoia	Suicidality	Motor Signs	Delayed Onset	Cognitive Only	Also Includes Physical (Motor) Disability
Jordan et al., 1995	1	128	1	NM	NM	Yes	NM	NM	NM	NM	NM	1	1	IV	IV
Geddes et al., 1996	1	129	2	NM	NM	Unclear	NM	NM	NM	NM	NM	0	NA	II	II
Williams & Tannenberg, 1996	1	130	1	1	1	Unclear	NM	1	NM	NM	NM	1	NM	III	III
Jordan et al., 1997	1	131	1	1	1	Yes	0	0	0	NM	NM	1	Yes	IV	IV
	2	132	0	0	0	No	0	0	0	NM	NM	0	No	I	I
	3	133	2	1	0	Unclear	0	0	0	NM	NM	1	NM	II	II
	4	134	0	0	0	No	0	0	0	NM	NM	0	No	I	I
	5	135	2	2	0	No	0	0	0	NM	NM	1	No	II	II
	6	136	0	0	0	No	0	0	0	NM	NM	0	No	I	I
	7	137	0	0	0	No	0	0	0	NM	NM	0	No	I	I
	8	138	0	0	0	No	0	0	0	NM	NM	0	No	I	I
	9	139	0	0	0	No	0	0	0	NM	NM	0	No	I	I
	10	140	1	NM	0	No	0	0	0	NM	NM	2	NM	II	II
	11	141	0	0	0	No	0	0	0	NM	NM	0	No	I	I
	12	142	0	NM	0	No	0	0	0	NM	NM	0	NM	I	I
	13	143	1	NM	0	No	0	0	0	NM	NM	1	NM	III	III
	14	144	1	NM	0	No	0	0	0	NM	NM	NM	NM	III	III
	15	145	1	NM	0	No	0	0	0	NM	NM	1	NM	III	III
	16	146	0	0	0	No	0	0	0	NM	NM	0	No	I	I
	17	147	1	NM	0	No	0	0	0	NM	NM	1	NM	II	II
	18	148	1	1	0	Yes	0	0	1	NM	NM	1	NM	III	III
	19	149	1	1	0	Unclear	0	0	0	NM	NM	1	NM	III	III
	20	150	0	0	0	No	0	0	0	NM	NM	0	No	I	I
	21	151	0	0	0	Unclear	0	0	0	NM	NM	1	NM	II	II
	22	152	1	NM	0	Unclear	0	0	0	NM	NM	2	NM	II	II
	23	153	0	0	0	Unclear	0	0	0	NM	NM	2	No	I	I
	24	154	0	0	0	Unclear	0	0	0	NM	NM	1	NM	II	II
	25	155	2	NM	0	Unclear	0	0	0	NM	NM	1	NM	II	II
	26	156	0	1	1	Unclear	0	0	NM	NM	NM	1	NM	II	II
	27	157	0	0	0	No	0	0	0	NM	NM	0	No	I	I
	28	158	1	NM	0	Unclear	0	0	0	NM	NM	1	NM	II	II
	29	159	1	NM	0	Unclear	1	0	0	NM	NM	1	NM	II	II
	30	160	0	0	0	No	0	0	0	NM	NM	0	No	I	I
Geddes et al., 1999	1	161	2	NM	NM	Unclear	NM	NM	NM	NM	NM	0	NA	II	II
	2	162	NM	NM	NM	Unclear	1	NM	NM	1	NM	NM	0	III	III
	3	163	2	NM	NM	Unclear	NM	NM	NM	NM	NM	NM	NA	III	III
	4	164	2	NM	NM	Unclear	NM	NM	NM	NM	NM	NM	NA	II	II
Newell & Drachman, 1999	1	165	1	1	1	Yes	NM	NM	NM	1	NM	1	1	IV	IV

Note: Not Present / No = 0; Present = 1; Unknown = 2; NM = Not Mentioned; NA = Not Applicable. Level of Functional Dependence/Dementia: I = Independent, II = Subtle/Mild Functional Limitation, III = Mild Dementia, IV = Moderate Dementia, and V = Severe Dementia. Whether the case had a delayed onset was obtained from the age at which the boxer retired or left the ring and the age of symptom onset. Some cases also provided the duration of the boxer's career. There are two categories for level of functional dependence/dementia. One that is strictly according to the new TES guidelines, where physical disability is not taken into account and the other included physical/motor disability in addition to cognition and neuropsychiatric problems. Per the Katz et al. 2021 article, "The level of functional dependence should be based on the impact of cognitive impairment and/or neurobehavioral dysregulation and not on physical limitations or medical illness. Information is obtained from self-report, informant, and/or clinical records. Functional dependence levels (other than independent) should represent a change from previous baseline functioning." (pg. 854) It is important to note that in subtle/mild functional limitation, authors do state, "fully independent in basic ADLs (personal hygiene and grooming [e.g., brushing/combing/styling hair], toilet hygiene [e.g., getting to the toilet, cleaning oneself, and getting back up], bathing/showering, dressing, self-feeding, and functional mobility [e.g., ability to walk, get in and out of bed, and get into and out of a chair])" (pg. 854). The ratings of functional dependence and dementia were particularly difficult due to the scant amount of information included in many articles that could be used for these classifications.

Table 2. Description of Rater Disagreements and Resolution (Rater Agreement)

		Core Clinical Features				Supportive Features								
Reference	Case	Cognitive Impairment	Executive Function Impairment	Neurobehavioral Dysregulation	Progressive Course	Depression	Anxiety	Apathy	Paranoia	Suicidality	Motor Signs (Parkinsonism/ ALS)	Delayed Onset of Symptoms and Problems	Level of Functional Dependence/ Dementia	Level of Functional Dependence/Dementia Including Physical (Motor) Disability
Critchley, 1949	C	1	NM	1	Unclear	NM	1	NM	NM	NM	1	0	III	III
AKK labeled neurobehavioral dysregulation as “Unknown,” while AG labeled as “Present.” After discussion, authors agreed this was present because the article discusses the person “left school at 14 in Standard VII, with an excellent character” and then was classified as a “general nuisance; not intelligent; a bad influence; and finally that he is evil.” (pg. 136)														
Critchley, 1949	E	1	NM	1	Unclear	0	NM	NM	NM	NM	1	NM	III	III
AKK labeled neurobehavioral dysregulation as “Unknown,” while AG labeled as “Present.” After discussion, authors agreed this was present because the article discusses the person “resisted with violence any attempts at further investigation.” (pg. 138) The Katz et al., 2021 criteria lists “violent outbursts” as neurobehavioral dysregulation. It is unknown if this was a change for the patient.														
Raevuori-Nallinma, 1950	1	1	NM	0	No	NM	1	NM	NM	NM	2	NM	II	II
AKK labeled as the course as “Non-Progressive,” while AG labeled as “Progressive.” After discussion, authors agreed this case was “Non-Progressive” because the article states “During a hospital observation of 5 months there were no changes to speak of in his condition, save that the headache grew less. After leaving the hospital he took up manual work lighter than his former job.” (pg. 54)														
Raevuori-Nallinma, 1950	2	1	NM	NM	Yes	NM	NM	1	NM	NM	1	NM	IV	IV
AKK labeled executive function impairment as “Present,” while AG labeled as “Not Mentioned.” After discussion, authors agreed executive function impairment was not mentioned in this case. The patient completed Kraepelin’s addition test as a neuropsychological measure, which we believe does not fall within the category of executive functioning.														
Critchley, 1957	2	1	NM	NM	Yes	NM	NM	NM	NM	NM	1	0	II	II
AKK labeled delayed onset of symptoms and problems as “Not Mentioned,” while AG labeled as “Not Present.” After discussion, authors agreed there was no delayed onset of symptoms as “This boxer, who was always liable to headaches after each contest, had found them more persistent in the last few months. It was noticed by others that he was becoming a little slower, and the speech somewhat thick. On the occasion of his last contest he was knocked out, and headaches thereafter became troublesome. Although the patient maintained that he felt well, he was noticeably dysarthric and slow in cerebration. There was a strong suspicion here of an early punch-drunkenness and he was advised to rest.” (pg. 359)														
Critchley, 1957	8	1	NM	NM	Yes	NM	NM	NM	NM	NM	1	0	II	II
AKK labeled the course as “Progressive,” while AG labeled as “Unable to Classify.” After discussion, authors agreed to label the course as progressive. Critchley (1957) introduces cases 1-11 with the statement “the following clinical notes illustrate some of the characteristics of traumatic progressive encephalopathy in boxers” (pg. 359). We accepted all cases as having a progressive course even though some had insufficient details to classify them as such.														
Critchley, 1957	9	1	NM	NM	Yes	NM	NM	NM	NM	NM	1	NM	II	II
AKK labeled the course as “Progressive,” while AG labeled as “Unable to Classify.” After discussion, authors agreed to label the course as progressive. Critchley (1957) introduces cases 1-11 with the statement “the following clinical notes illustrate some of the characteristics of traumatic progressive encephalopathy in boxers” (pg. 359). We accepted all cases as having a progressive course even though some had insufficient details to classify them as such.														
Critchley, 1957	10	NM	NM	1	Yes	NM	NM	NM	NM	NM	1	NM	II	II
AKK labeled neurobehavioral dysregulation as “Unknown,” while AG labeled as “Present.” After discussion, authors agreed to label neurobehavioral dysregulation as present because the patient lost his temper with his fiancé. AKK labeled the course as “Progressive,” while AG labeled as “Unable to Classify.” After discussion, authors agreed to label the course as progressive. Critchley (1957) introduces cases 1-11 with the statement “the following clinical notes illustrate some of the characteristics of traumatic progressive encephalopathy in boxers” (pg. 359). We accepted all cases as having a progressive course even though some had insufficient details to classify them as such.														
Neubuerger, Sinton, & Denst, 1959	1	1	0	1	Yes	NM	1	NM	1	NM	1	1	III	III
AKK labeled executive function impairment as “Present,” while AG labeled as “Absent.” After discussion, authors agreed to label as absent. The patient was administered digits forwards, serial sevens, simple calculations, and questions recalling the presidents and dates of world wars, which we agreed do not classify as measures of executive functioning. AKK labeled neurobehavioral dysregulation as “Present,” while AG labeled as														

		Core Clinical Features				Supportive Features								
**Reference**	**Case**	**Cognitive Impairment**	**Executive Function Impairment**	**Neurobehavioral Dysregulation**	**Progressive Course**	**Depression**	**Anxiety**	**Apathy**	**Paranoia**	**Suicidality**	**Motor Signs (Parkinsonism/ ALS)**	**Delayed Onset of Symptoms and Problems**	**Level of Functional Dependence/ Dementia**	**Level of Functional Dependence/Dementia Including Physical (Motor) Disability**
"Absent." After discussion, authors agreed neurobehavioral dysregulation was present because "It was noted incidentally that he was tense, tremulous, suspicious, and irritable, with some delusory phenomena." (pg. 19/403).														
Spillane, 1962	1	0	NM	0	Yes	0	NM	NM	NM	NM	1	0	I	II
AKK labeled level of functional dependence/dementia including physical (motor) disability as II / III, while AG labeled as I / II. After discussion, authors agreed on Independent functional limitation (I), as the article states, "Although he is slow and euphoric there was no significant evidence of intellectual deterioration and he has continued to work regularly and is not very disabled." (pg. 1208) The patient's primary disabilities were dysarthria and unsteady gait, thus level of functional dependence/dementia including physical (motor) disability was rated as Subtle/Mild functional limitation (II).														
Mawdsley & Ferguson, 1963	8	1	NM	1	Unclear	NM	NM	NM	NM	NM	1	NM	IV	IV
AKK labeled neurobehavioral dysregulation as "Unknown," while AG labeled as "Present." After discussion, authors agreed neurobehavioral dysregulation was present, because the article states, "In his youth he had been aggressive, and throughout his boxing career was a heavy drinker. He is still liable to outbursts of rage and violence but drinks little alcohol, because small amounts aggravate his ataxia. When he was 34 he was discharged from the Army on psychiatric grounds and he has not worked since. Between the ages of 37 and 44 he was admitted to mental hospitals on three occasions after episodes of violence." (pg. 795)														
Payne, 1968	1	NM	NM	NM	Unclear	1	NM	NM	NM	NM	NM	NM	III	III
AKK labeled paranoia as "Present," while AG labeled as "Not Mentioned." After discussion, authors agree that paranoia was not mentioned. The patient in the case was diagnosed with "Manic Depressive Psychosis," which is not the same as paranoia. AKK labeled level of functional dependence/dementia including physical (motor) disability as II / III, while AG labeled as "Not Mentioned." After discussion, authors agree the level of functional dependence is the same for inclusion of cognitive and physical disability (III, otherwise known as Mild Dementia).														
Payne, 1968	3	1	1	1	Unclear	1	NM	NM	NM	NM	1	NM	III	III
AKK labeled executive function impairment as "Present," while AG labeled as "Absent." After discussion, authors agreed executive function impairment is present due to "lack of concentration" and "becoming dirty and untidy in his dress." (pg. 177)														
Johnson, 1969	12	0	NM	0	Yes	NM	NM	NM	0	NM	1	NM	II	III
AKK labeled level of functional dependence / dementia as "I / II," while AG labeled as "III." After discussion, authors agreed on Subtle / Mild Functional Limitation (II) because the patient did not display any obvious/documented cognitive impairment or neurobehavioral dysregulation. The patient was however rendered "a physical invalid" in a motorized chair due to progression of neurological symptoms, which is why motor disability is rated higher. (pg. 47)														
Roberts, 1969	3	2	NM	NM	Yes	NM	NM	NM	NM	NM	1	1	III	III
AKK labeled cognitive impairment as "Unknown," while AG labeled as "Present." After discussion, authors agreed cognitive impairment was "Unknown." The article reports, "Examined, it was apparent that his mentation was extremely slow and that he had difficulty recalling names and places in his boxing career." (pg. 31) However, performance on neurocognitive measures were average. "He scored in the fiftieth percentile on Raven's Matrices, learned his digit span of seven plus one at the fourth repetition, recalled accurately only one of the pair of Binet designs at the first attempt, but both at the second presentation and both accurately after fifteen minutes delay." (pg. 31)														
Roberts, 1969	4	0	NM	0	Yes	NM	NM	NM	NM	NM	1	NM	II	II
AKK labeled course as "Unable to Classify," while AG labeled as "Progressive." After discussion, authors agreed this case presents as progressive, because "The history was quite clear on the point of progression in two of these, the second and fourth cases, in that an extra-pyramidal tremor had made its appearance some years later on a background of established dysarthria first noticed during their last years boxing." (pg. 44)														
Roberts, 1969	5	1	NM	1	Unclear	1	NM	1	NM	NM	1	NM	IV	IV
AKK labeled neurobehavioral dysregulation as "Unknown," while AG labeled as "Present." After discussion, authors agreed neurobehavioral dysregulation was present. The article discusses the patient was a "chronic alcoholic for many years" and "drank excessively." He was divorced due to his "increasingly trying behavior" and general exam was "precluded by the patient's unwillingness to co-operate further." (pg. 33)														

Note: 0: Not Present / No; 1: Present; 2: Unknown; NM: Not Mentioned. Level of Functional Dependence/Dementia: I = Independent, II = Subtle/Mild Functional Limitation, III = Mild Dementia, IV = Moderate Dementia, and V = Severe Dementia.

After the first round of scientific peer review, the authors re-examined some of the articles to determine if presumed missing data could be coded (i.e., some data originally coded as “Not Mentioned” might be coded as “Not Present”). This was done by two authors (GLI and AKK). All changes are reflected in the case descriptions throughout this supplement.

Changes were made to the Jordan et al. (1997) coding given their use of the Chronic Brain Injury Scale (CBI), and how they tabled their results in the article. Specifically, if a boxer was identified as “Normal” and was given a score of 0 on the CBI scale, he was coded by us as not evidencing any motor signs, cognitive deficits (e.g., MMSE $\geq$28), or behavioral symptoms, not experiencing a delayed onset or progressive course, and labeled as Independent for functional impairment. Paranoia could not be determined given behavioral symptoms, as defined by Jordan et al., included, “agitation or aggression, delusions, hallucinations, dysphoria, anxiety, euphoria, apathy, disinhibition, irritability or lability, or aberrant motor behavior” (pg. 137). This changed 28 cases that had some symptoms coded as “Not Mentioned” to “Not Present.”

In addition, relying on a clinical features and investigations table in Johnson (1969), five boxers were identified to have “Present” motor signs that were previously coded as “Not Mentioned.” This change was due to clinical vignettes not describing motor signs, although some specific neurological symptoms were noted in a table. Similarly, four boxers initially coded as “Not Mentioned” for neurobehavioral dysregulation were later coded as “Not Present” because the table indicated no for any psychiatric symptoms. In Casson et al. (1984), motor signs for three cases and functional impairment in 16 cases were adjusted using the neurological examination table (pg. 2,664). In addition, delayed onset was changed from “Not Mentioned to “Not Present” in five cases of boxers who were still actively competing.

In Kaste et al. (1982), relying on the abnormalities in neurological status, neuropsychological tests, and subjective symptoms, twelve cases were changed from “Not Mentioned” to “Not Present” for neurobehavioral dysregulation, executive function impairment, progressive course, and delayed onset, as well as labeled as Independent for functional impairment, if the boxer was identified as “Normal.” Two cases found the presence of executive function impairment, that we previously coded as “Not Mentioned.”

Additional cases that were initially reported as “Unclear” for progressive course and “Unknown” for delayed onset were also re-evaluated by the authors and resolved through discussion. Of these cases, three were changed from “Unclear” progressive course (Parker, 1934; Roberts, 1969; Corsellis, 1973) and one was changed from “Unknown” delayed onset (Roberts, 1969), in addition to one case changing to “Not Mentioned” delayed onset due to a later in life injury (Spillane, 1962).

Furthermore, Case 4 in Johnson (1969) originally was not included in our coding because he was described as follows: “an amateur, developed anxiety symptoms following a domestic crisis and falsely attributed these to insidious “punch drunkenness.” All investigations were normal and at follow-up he was symptom-free.” He was added, however, and coded as mostly “absent” (0) for clinical features. Similarly, Case 17 in Johnson (1969) which was originally not included because the case was not presented in Victoroff (2013) because it appeared his symptoms resulted from one championship fight and a leucotomy, after which he developed epilepsy, was added to the analyses.

Table 3. Coding the TES Consensus Criteria for Cases from the 20th Century for Statistical Analyses

Reference	Case	Case #	Cognitive Impairment	Executive Function Impairment	Neurobehavioral Dysregulation	Progressive Course	Depression	Anxiety	Apathy	Paranoia	Suicidality	Motor Signs	Delayed Onset	Cognitive Only	Also Includes Physical (Motor) Disability
Martland, 1928	2	1	0			1						1	0	2	2
Parker, 1934	1	2	1			0		1				1	0	2	2
	2	3	1			0						1	0	2	3
	3	4	1									1	0	2	3
Critchley, 1949	A	5												2	2
	B	6	1	1										4	4
	C	7	1		1			1				1	0	3	3
	D	8	1		1		1							3	3
	E	9	1		1		0					1		3	3
	F	10	1									1		3	3
	G	11												2	2
Raevuori-Nallia, 1950	1	12	1		0	0		1						2	2
	2	13	1			1			1			1		4	4
Critchley, 1957	2	14	1			1						1	0	2	2
	3	15	1			1	0							2	2
	8	16	1			1						1	0	2	2
	9	17	1			1						1		2	2
	10	18			1	1						1		2	2
	11	19	1	1	1	1						1		2	2
	14	20	1		0							2		2	2
Corsellis & Brierley, 1959	1	21	1	1	1	1				1		2		4	4
	2	22	1		1	1				1		1		5	5
Neubuerger, Sinton, & Denst, 1959	1	23	1	0	1	1		1		1		1	1	3	3
	2	24	1		0	1		1				1	1	4	4
Courville, 1962	1	25	1		1	1						1		4	4
Spillane, 1962	1	26	0		0	1	0					1	0	1	2
	2	27	1			1	1	1				1		4	4
	3	28	1	1		1	0					1	1	4	4
	4	29	1		1							0		2	2
	5	30	0			1	1					1	0	2	2
Mawdsley & Ferguson, 1963	1	31	1	1	1	1						1	0	4	4
	2	32	1			1						1	0	4	4
	3	33	1		1	1	1					0	1	4	4
	4	34	1		1					1		1	1	4	4
	5	35	1		1	1	1			1		1	0	4	4
	6	36	1			1						1	1	3	3
	7	37	1			1						1	1	3	3
	8	38	1		1							1		4	4
	9	39	1			1						1	0	3	3
	10	40	1		0	1			1			1	0	4	4
Payne, 1968	1	41					1							3	3
	2	42			1	1	1			1		1	0	3	3
	3	43	1	1	1		1					1		3	3
	4	44	0		1		1					1		1	3
	5	45												1	2

Reference	Case	Case #	Cognitive Impairment	Executive Function Impairment	Neurobehavioral Dysregulation	Progressive Course	Depression	Anxiety	Apathy	Paranoia	Suicidality	Motor Signs	Delayed Onset	Cognitive Only	Also Includes Physical (Motor) Disability
	6	46												2	2
Johnson, 1969	1	47	1	1	1	1			1	1		1	0	4	4
	2	48	1		0	1						1		3	3
	3	49	1		0	0						1		3	3
	4	50	0	0	0	0	0	1		0		0	0	1	1
	5	51	1		0	0						0		2	2
	6	52	1		1	0				1		1	0	3	3
	7	53	1		0	0						1		2	2
	8	54	1		1	1	1			1		1		3	3
	9	55	1		1	0				1		1		3	3
	10	56	1		1	0				1		1		3	3
	11	57	1		0	1						1		3	3
	12	58	0		0	1				0		1		2	3
	13	59	1		0	0						1		3	3
	14	60	0		0	0						1		1	2
	15	61	1		0	1						1		3	3
	16	62	1	1	0	1						1		4	4
	17	63	1	1	1	1			1	1		1	1	5	5
Roberts, 1969	1	64	1	1		1				1		1	0	4	4
	2	65	0		1	1						1	0	3	3
	3	66				1						1	1	3	3
	4	67	0		0	1						1		2	2
	5	68	1		1		1		1			1		4	4
	6	69	1		1	1				1		1	0	4	4
	7	70	0			1						1		2	2
	8	71	0		0	0						1		1	1
	9	72	0									1	0	1	1
	10	73	0		0	0						1	0	2	2
	11	74	1			0							0	3	3
Corsellis, Bruton, & Freeman-Browne, 1973	1	75	1	1	1	1								4	4
	2	76	1	1	1	1						1	0	4	4
	3	77	1		1	1				1		1	0	4	4
	4	78	1			1						1	0	4	4
	5	79	1		1	1							0	4	4
	6	80	1		1	1				1		1	0	3	3
	7	81	1	1	1	1						1		4	4
	8	82	1		1	1						1	1	4	4
	9	83	1			1					1	1		4	4
	10	84	1		1	1				1		1		5	5
	11	85	1		1								1	4	4
	12	86	0		1	0								2	2
	13	87	1	1	1	1				1				4	4
	14	88	0			0						0		2	2
	15	89				0						0		2	2
Harvey & Newsom Davis, 1974	1	90	1		1	1	1			1		1	0	4	4
Kaste et al., 1982	1	91	1	1								1		3	3
	2	92	1	1								0		3	3
	3	93	0	0	0	0						0	0	1	1

Reference	Case	Case #	Cognitive Impairment	Executive Function Impairment	Neurobehavioral Dysregulation	Progressive Course	Depression	Anxiety	Apathy	Paranoia	Suicidality	Motor Signs	Delayed Onset	Cognitive Only	Also Includes Physical (Motor) Disability
	4	94	0	0	0	0						0	0	1	1
	5	95	0	0	0	0						0	0	1	1
	6	96	0	0	0	0						0	0	1	1
	7	97	0	0	0	0						0	0	1	1
	8	98	0	0	0	0						0	0	1	1
	9	99	0	0	0	0						0	0	1	1
	10	100	0	0	0	0						0	0	1	1
	11	101	0	0	0	0						0	0	1	1
	12	102	0	0	0	0						0	0	1	1
	13	103	0	0	0	0						0	0	1	1
	14	104	0	0	0	0						0	0	1	1
Casson et al., 1984	1	105	1	0	0		0	0	0	0	0	0		2	2
	2	106	1	1	0		0	0	0	0	0	0		2	2
	3	107	1	0	0		0	0	0	0	0	0		1	1
	4	108	1	0	0		0	0	0	0	0	0		2	2
	5	109	1	1	0		0	0	0	0	0	0		2	2
	6	110	1	0	0		0	0	0	0	0	0		2	2
	7	111	1	1	0		0	0	0	0	0	1		2	2
	8	112	1	1	0		0	0	0	0	0	0		2	2
	9	113	1	1	0		0	0	0	0	0	0		2	2
	10	114	1	1	0		0	0	0	0	0	1		2	2
	11	115	1	1	0		0	0	0	0	0	0		2	2
	12	116	1	1	0		0	0	0	0	0	0		2	2
	13	117	1	1	0		0	0	0	0	0	0		2	2
	14	118	1	1	0		0	0	0	0	0	0	0	2	2
	15	119	1	0	0		0	0	0	0	0	0	0	2	2
	16	120	0		0		0	0	0	0	0	0	0	1	1
	17	121	0		0		0	0	0	0	0	0	0	1	1
	18	122	0		0		0	0	0	0	0	0	0	1	1
Roberts et al., 1990	1	123	1											4	4
Hof et al., 1991	1	124				1						1		2	4
Hof et al., 1992	1	125	1	1			1					1		3	4
	2	126	1	1			1					1		3	4
	3	127	1	1			1					1		3	4
Jordan et al., 1995	1	128	1			1						1	1	4	4
Geddes et al., 1996	1	129										0		2	2
Williams & Tannenberg, 1996	1	130	1	1	1			1				1		3	3
Jordan et al., 1997	1	131	1	1	1	1	0	0	0			1	1	4	4
	2	132	0	0	0	0	0	0	0			0	0	1	1
	3	133	2	1	0		0	0	0			1		2	2
	4	134	0	0	0	0	0	0	0			0	0	1	1
	5	135			0	0	0	0	0			1	0	2	2
	6	136	0	0	0	0	0	0	0			0	0	1	1
	7	137	0	0	0	0	0	0	0			0	0	1	1
	8	138	0	0	0	0	0	0	0			0	0	1	1
	9	139	0	0	0	0	0	0	0			0	0	1	1
	10	140	1		0	0	0	0	0					2	2
	11	141	0	0	0	0	0	0	0			0	0	1	1

Reference	Case	Case #	Cognitive Impairment	Executive Function Impairment	Neurobehavioral Dysregulation	Progressive Course	Depression	Anxiety	Apathy	Paranoia	Suicidality	Motor Signs	Delayed Onset	Cognitive Only	Also Includes Physical (Motor) Disability
	12	142	0		0	0	0	0	0			0		1	1
	13	143	1		0	0	0	0	0			1		3	3
	14	144	1		0	0	0	0	0					3	3
	15	145	1		0	0	0	0	0			1		3	3
	16	146	0	0	0	0	0	0	0			0	0	1	1
	17	147	1		0	0	0	0	0			1		2	2
	18	148	1	1	0	1	0	0	1			1		3	3
	19	149	1	1	0		0	0	0			1		3	3
	20	150	0	0	0	0	0	0	0			0	0	1	1
	21	151	0	0	0		0	0	0			1		2	2
	22	152	1		0		0	0	0					2	2
	23	153	0	0	0		0	0	0				0	1	1
	24	154	0	0	0		0	0	0			1		2	2
	25	155			0		0	0	0			1		2	2
	26	156	0	1	1		0	0				1		2	2
	27	157	0	0	0	0	0	0	0			0	0	1	1
	28	158	1		0		0	0	0			1		2	2
	29	159	1		0		1	0	0			1		2	2
	30	160	0	0	0	0	0	0	0			0	0	1	1
Geddes et al., 1999	1	161										0		2	2
	2	162					1			1			0	3	3
	3	163												3	3
	4	164												2	2
Newell & Drachman, 1999	1	165	1	1	1	1				1		1	1	4	4

Note: Not Present / No = 0; Present = 1. Level of Functional Dependence/Dementia: 1 = Independent, 2 = Subtle/Mild Functional Limitation, 3 = Mild Dementia, 4 = Moderate Dementia, and V = Severe Dementia. Case numbers #21, 22, 123, 124, 129, 130, 163, and 164, from Table 1, were excluded and were not included in the statistical analyses.

Table 4. Coding the TES Consensus Criteria for Final 157 Cases from the 20th Century for Statistical Analyses

	Case #	Cognitive Impairment	Executive Function Impairment	Neurobehavioral Dysregulation	Progressive Course	Depression	Anxiety	Apathy	Paranoia	Motor Signs	Delayed Onset	Functional Impairment	Diagnosed TES	Provisional Levels of Certainty for CTE
1	1	No	.	.	Yes	.	.	.	.	Yes	No	Subtle/Mild Functional Limitation	No	.
2	2	Yes	.	.	No	.	Yes	.	.	Yes	No	Subtle/Mild Functional Limitation	.	.
3	3	Yes	.	.	No	.	.	.	.	Yes	No	Subtle/Mild Functional Limitation	.	.
4	4	Yes	.	.	.	.	.	.	.	Yes	No	Subtle/Mild Functional Limitation	.	.
5	5	.	.	.	.	.	.	.	.	.	.	Subtle/Mild Functional Limitation	.	.
6	6	Yes	Yes	.	.	.	.	.	.	.	.	Moderate Dementia	.	.
7	7	Yes	.	Yes	.	.	Yes	.	.	Yes	No	Mild Dementia	.	.
8	8	Yes	.	Yes	.	Yes	.	.	.	.	.	Mild Dementia	.	.
9	9	Yes	.	Yes	.	No	.	.	.	Yes	.	Mild Dementia	.	.
10	10	Yes	.	.	.	.	.	.	.	Yes	.	Mild Dementia	.	.
11	11	.	.	.	.	.	.	.	.	.	.	Subtle/Mild Functional Limitation	.	.
12	12	Yes	.	No	No	.	Yes	.	.	.	.	Subtle/Mild Functional Limitation	.	.
13	13	Yes	.	.	Yes	.	.	Yes	.	Yes	.	Moderate Dementia	Yes	Probable
14	14	Yes	.	.	Yes	.	.	.	.	Yes	No	Subtle/Mild Functional Limitation	Yes	Possible
15	15	Yes	.	.	Yes	No	.	.	.	.	.	Subtle/Mild Functional Limitation	Yes	Suggestive
16	16	Yes	.	.	Yes	.	.	.	.	Yes	No	Subtle/Mild Functional Limitation	Yes	Possible
17	17	Yes	.	.	Yes	.	.	.	.	Yes	.	Subtle/Mild Functional Limitation	Yes	Possible
18	18	.	.	Yes	Yes	.	.	.	.	Yes	.	Subtle/Mild Functional Limitation	Yes	Possible
19	19	Yes	Yes	Yes	Yes	.	.	.	.	Yes	.	Subtle/Mild Functional Limitation	Yes	Possible
20	20	Yes	.	No	.	.	.	.	.	.	.	Subtle/Mild Functional Limitation	.	.
21	23	Yes	No	Yes	Yes	.	Yes	.	Yes	Yes	Yes	Mild Dementia	Yes	Probable
22	24	Yes	.	No	Yes	.	Yes	.	.	Yes	Yes	Moderate Dementia	Yes	Probable
23	25	Yes	.	Yes	Yes	.	.	.	.	Yes	.	Moderate Dementia	Yes	Probable
24	26	No	.	No	Yes	No	.	.	.	Yes	No	Independent	No	.
25	27	Yes	.	.	Yes	Yes	Yes	.	.	Yes	.	Moderate Dementia	Yes	Probable
26	28	Yes	Yes	.	Yes	No	.	.	.	Yes	Yes	Moderate Dementia	Yes	Probable
27	29	Yes	.	Yes	.	.	.	.	.	No	.	Subtle/Mild Functional Limitation	.	.
28	30	No	.	.	Yes	Yes	.	.	.	Yes	No	Subtle/Mild Functional Limitation	No	.
29	31	Yes	Yes	Yes	Yes	.	.	.	.	Yes	No	Moderate Dementia	Yes	Probable
30	32	Yes	.	.	Yes	.	.	.	.	Yes	No	Moderate Dementia	Yes	Possible
31	33	Yes	.	Yes	Yes	Yes	.	.	.	No	Yes	Moderate Dementia	Yes	Probable
32	34	Yes	.	Yes	.	.	.	.	Yes	Yes	Yes	Moderate Dementia	.	.
33	35	Yes	.	Yes	Yes	Yes	.	.	Yes	Yes	No	Moderate Dementia	Yes	Probable
34	36	Yes	.	.	Yes	.	.	.	.	Yes	Yes	Mild Dementia	Yes	Probable
35	37	Yes	.	.	Yes	.	.	.	.	Yes	Yes	Mild Dementia	Yes	Probable
36	38	Yes	.	Yes	.	.	.	.	.	Yes	.	Moderate Dementia	.	.
37	39	Yes	.	.	Yes	.	.	.	.	Yes	No	Mild Dementia	Yes	Possible
38	40	Yes	.	No	Yes	.	.	Yes	.	Yes	No	Moderate Dementia	Yes	Probable
39	41	.	.	.	.	Yes	.	.	.	.	.	Mild Dementia	.	.
40	42	.	.	Yes	Yes	Yes	.	.	Yes	Yes	No	Mild Dementia	Yes	Probable
41	43	Yes	Yes	Yes	.	Yes	.	.	.	Yes	.	Mild Dementia	.	.
42	44	No	.	Yes	.	Yes	.	.	.	Yes	.	Independent	.	.
43	45	.	.	.	.	.	.	.	.	.	.	Independent	.	.
44	46	.	.	.	.	.	.	.	.	.	.	Subtle/Mild Functional Limitation	.	.
45	47	Yes	Yes	Yes	Yes	.	.	Yes	Yes	Yes	No	Moderate Dementia	Yes	Probable
46	48	Yes	.	No	Yes	.	.	.	.	Yes	.	Mild Dementia	Yes	Possible
47	49	Yes	.	No	No	.	.	.	.	Yes	.	Mild Dementia	.	.

	Case #	Cognitive Impairment	Executive Function Impairment	Neurobehavioral Dysregulation	Progressive Course	Depression	Anxiety	Apathy	Paranoia	Motor Signs	Delayed Onset	Functional Impairment	Diagnosed TES	Provisional Levels of Certainty for CTE
48	50	No	No	No	No	No	Yes	.	No	No	No	Independent	No	.
49	51	Yes	.	No	No	.	.	.	.	No	.	Subtle/Mild Functional Limitation	.	.
50	52	Yes	.	Yes	No	.	.	.	Yes	Yes	No	Mild Dementia	.	.
51	53	Yes	.	No	No	.	.	.	.	Yes	.	Subtle/Mild Functional Limitation	.	.
52	54	Yes	.	Yes	Yes	Yes	.	.	Yes	Yes	.	Mild Dementia	Yes	Probable
53	55	Yes	.	Yes	No	.	.	.	Yes	Yes	.	Mild Dementia	.	.
54	56	Yes	.	Yes	No	.	.	.	Yes	Yes	.	Mild Dementia	.	.
55	57	Yes	.	No	Yes	.	.	.	.	Yes	.	Mild Dementia	Yes	Possible
56	58	No	.	No	Yes	.	.	.	No	Yes	.	Subtle/Mild Functional Limitation	No	.
57	59	Yes	.	No	No	.	.	.	.	Yes	.	Mild Dementia	.	.
58	60	No	.	No	No	.	.	.	.	Yes	.	Independent	No	.
59	61	Yes	.	No	Yes	.	.	.	.	Yes	.	Mild Dementia	Yes	Possible
60	62	Yes	Yes	No	Yes	.	.	.	.	Yes	.	Moderate Dementia	Yes	Possible
61	63	Yes	Yes	Yes	Yes	.	.	Yes	Yes	Yes	Yes	Severe Dementia	Yes	Probable
62	64	Yes	Yes	.	Yes	.	.	.	Yes	Yes	No	Moderate Dementia	Yes	Probable
63	65	No	.	Yes	Yes	.	.	.	.	Yes	No	Mild Dementia	Yes	Probable
64	66	.	.	.	Yes	.	.	.	.	Yes	Yes	Mild Dementia	.	.
65	67	No	.	No	Yes	.	.	.	.	Yes	.	Subtle/Mild Functional Limitation	No	.
66	68	Yes	.	Yes	.	Yes	.	Yes	.	Yes	.	Moderate Dementia	.	.
67	69	Yes	.	Yes	Yes	.	.	.	Yes	Yes	No	Moderate Dementia	Yes	Probable
68	70	No	.	.	Yes	.	.	.	.	Yes	.	Subtle/Mild Functional Limitation	No	.
69	71	No	.	No	No	.	.	.	.	Yes	.	Independent	No	.
70	72	No	.	.	.	.	.	.	.	Yes	No	Independent	No	.
71	73	No	.	No	No	.	.	.	.	Yes	No	Subtle/Mild Functional Limitation	No	.
72	74	Yes	.	.	No	.	.	.	.	.	No	Mild Dementia	.	.
73	75	Yes	Yes	Yes	Yes	.	.	.	.	.	.	Moderate Dementia	Yes	Possible
74	76	Yes	Yes	Yes	Yes	.	.	.	.	Yes	No	Moderate Dementia	Yes	Probable
75	77	Yes	.	Yes	Yes	.	.	.	Yes	Yes	No	Moderate Dementia	Yes	Probable
76	78	Yes	.	.	Yes	.	.	.	.	Yes	No	Moderate Dementia	Yes	Possible
77	79	Yes	.	Yes	Yes	.	.	.	.	.	No	Moderate Dementia	Yes	Possible
78	80	Yes	.	Yes	Yes	.	.	.	Yes	Yes	No	Mild Dementia	Yes	Probable
79	81	Yes	Yes	Yes	Yes	.	.	.	.	Yes	.	Moderate Dementia	Yes	Probable
80	82	Yes	.	Yes	Yes	.	.	.	.	Yes	Yes	Moderate Dementia	Yes	Probable
81	83	Yes	.	.	Yes	.	.	.	.	Yes	.	Moderate Dementia	Yes	Possible
82	84	Yes	.	Yes	Yes	.	.	.	Yes	Yes	.	Severe Dementia	Yes	Probable
83	85	Yes	.	Yes	.	.	.	.	.	.	Yes	Moderate Dementia	.	.
84	86	No	.	Yes	No	.	.	.	.	.	.	Subtle/Mild Functional Limitation	.	.
85	87	Yes	Yes	Yes	Yes	.	.	.	Yes	.	.	Moderate Dementia	Yes	Probable
86	88	No	.	.	No	.	.	.	.	No	.	Subtle/Mild Functional Limitation	No	.
87	89	.	.	.	No	.	.	.	.	No	.	Subtle/Mild Functional Limitation	.	.
88	90	Yes	.	Yes	Yes	Yes	.	.	Yes	Yes	No	Moderate Dementia	Yes	Probable
89	91	Yes	Yes	.	.	.	.	.	.	Yes	.	Mild Dementia	.	.
90	92	Yes	Yes	.	.	.	.	.	.	No	.	Mild Dementia	.	.
91	93	No	No	No	No	.	.	.	.	No	No	Independent	No	.
92	94	No	No	No	No	.	.	.	.	No	No	Independent	No	.
93	95	No	No	No	No	.	.	.	.	No	No	Independent	No	.
94	96	No	No	No	No	.	.	.	.	No	No	Independent	No	.
95	97	No	No	No	No	.	.	.	.	No	No	Independent	No	.
96	98	No	No	No	No	.	.	.	.	No	No	Independent	No	.
97	99	No	No	No	No	.	.	.	.	No	No	Independent	No	.

	Case #	Cognitive Impairment	Executive Function Impairment	Neurobehavioral Dysregulation	Progressive Course	Depression	Anxiety	Apathy	Paranoia	Motor Signs	Delayed Onset	Functional Impairment	Diagnosed TES	Provisional Levels of Certainty for CTE
98	100	No	No	No	No	.	.	.	.	No	No	Independent	No	.
99	101	No	No	No	No	.	.	.	.	No	No	Independent	No	.
100	102	No	No	No	No	.	.	.	.	No	No	Independent	No	.
101	103	No	No	No	No	.	.	.	.	No	No	Independent	No	.
102	104	No	No	No	No	.	.	.	.	No	No	Independent	No	.
103	105	Yes	No	No	.	No	No	No	No	No	.	Subtle/Mild Functional Limitation	.	.
104	106	Yes	Yes	No	.	No	No	No	No	No	.	Subtle/Mild Functional Limitation	.	.
105	107	Yes	No	No	.	No	No	No	No	No	.	Independent	.	.
106	108	Yes	No	No	.	No	No	No	No	No	.	Subtle/Mild Functional Limitation	.	.
107	109	Yes	Yes	No	.	No	No	No	No	No	.	Subtle/Mild Functional Limitation	.	.
108	110	Yes	No	No	.	No	No	No	No	No	.	Subtle/Mild Functional Limitation	.	.
109	111	Yes	Yes	No	.	No	No	No	No	Yes	.	Subtle/Mild Functional Limitation	.	.
110	112	Yes	Yes	No	.	No	No	No	No	No	.	Subtle/Mild Functional Limitation	.	.
111	113	Yes	Yes	No	.	No	No	No	No	No	.	Subtle/Mild Functional Limitation	.	.
112	114	Yes	Yes	No	.	No	No	No	No	Yes	.	Subtle/Mild Functional Limitation	.	.
113	115	Yes	Yes	No	.	No	No	No	No	No	.	Subtle/Mild Functional Limitation	.	.
114	116	Yes	Yes	No	.	No	No	No	No	No	.	Subtle/Mild Functional Limitation	.	.
115	117	Yes	Yes	No	.	No	No	No	No	No	.	Subtle/Mild Functional Limitation	.	.
116	118	Yes	Yes	No	.	No	No	No	No	No	No	Subtle/Mild Functional Limitation	.	.
117	119	Yes	No	No	.	No	No	No	No	No	No	Subtle/Mild Functional Limitation	.	.
118	120	No	.	No	.	No	No	No	No	No	No	Independent	No	.
119	121	No	.	No	.	No	No	No	No	No	No	Independent	No	.
120	122	No	.	No	.	No	No	No	No	No	No	Independent	No	.
121	125	Yes	Yes	.	.	Yes	.	.	.	Yes	.	Mild Dementia	.	.
122	126	Yes	Yes	.	.	Yes	.	.	.	Yes	.	Mild Dementia	.	.
123	127	Yes	Yes	.	.	Yes	.	.	.	Yes	.	Mild Dementia	.	.
124	128	Yes	.	.	Yes	.	.	.	.	Yes	Yes	Moderate Dementia	Yes	Probable
125	131	Yes	Yes	Yes	Yes	No	No	No	.	Yes	Yes	Moderate Dementia	Yes	Probable
126	132	No	No	No	No	No	No	No	.	No	No	Independent	No	.
127	133	.	Yes	No	.	No	No	No	.	Yes	.	Subtle/Mild Functional Limitation	.	.
128	134	No	No	No	No	No	No	No	.	No	No	Independent	No	.
129	135	.	.	No	No	No	No	No	.	Yes	No	Subtle/Mild Functional Limitation	No	.
130	136	No	No	No	No	No	No	No	.	No	No	Independent	No	.
131	137	No	No	No	No	No	No	No	.	No	No	Independent	No	.
132	138	No	No	No	No	No	No	No	.	No	No	Independent	No	.
133	139	No	No	No	No	No	No	No	.	No	No	Independent	No	.
134	140	Yes	.	No	No	No	No	No	.	.	.	Subtle/Mild Functional Limitation	.	.
135	141	No	No	No	No	No	No	No	.	No	No	Independent	No	.
136	142	No	.	No	No	No	No	No	.	No	.	Independent	No	.
137	143	Yes	.	No	No	No	No	No	.	Yes	.	Mild Dementia	.	.
138	144	Yes	.	No	No	No	No	No	.	.	.	Mild Dementia	.	.
139	145	Yes	.	No	No	No	No	No	.	Yes	.	Mild Dementia	.	.
140	146	No	No	No	No	No	No	No	.	No	No	Independent	No	.
141	147	Yes	.	No	No	No	No	No	.	Yes	.	Subtle/Mild Functional Limitation	.	.
142	148	Yes	Yes	No	Yes	No	No	Yes	.	Yes	.	Mild Dementia	Yes	Probable
143	149	Yes	Yes	No	.	No	No	No	.	Yes	.	Mild Dementia	.	.
144	150	No	No	No	No	No	No	No	.	No	No	Independent	.	.
145	151	No	No	No	.	No	No	No	.	Yes	.	Subtle/Mild Functional Limitation	No	.
146	152	Yes	.	No	.	No	No	No	.	.	.	Subtle/Mild Functional Limitation	.	.
147	153	No	No	No	.	No	No	No	.	.	No	Independent	No	.

	Case #	Cognitive Impairment	Executive Function Impairment	Neurobehavioral Dysregulation	Progressive Course	Depression	Anxiety	Apathy	Paranoia	Motor Signs	Delayed Onset	Functional Impairment	Diagnosed TES	Provisional Levels of Certainty for CTE
148	154	No	No	No	.	No	No	No	.	Yes	.	Subtle/Mild Functional Limitation	No	.
149	155	.	.	No	.	No	No	No	.	Yes	.	Subtle/Mild Functional Limitation	No	.
150	156	No	Yes	Yes	.	No	No	.	.	Yes	.	Subtle/Mild Functional Limitation	.	.
151	157	No	No	No	No	No	No	No	.	No	No	Independent	No	.
152	158	Yes	.	No	.	No	No	No	.	Yes	.	Subtle/Mild Functional Limitation	.	.
153	159	Yes	.	No	.	Yes	No	No	.	Yes	.	Subtle/Mild Functional Limitation	.	.
154	160	No	No	No	No	No	No	No	.	No	No	Independent	No	.
155	161	.	.	.	.	.	.	.	.	No	.	Subtle/Mild Functional Limitation	.	.
156	162	.	.	.	.	Yes	.	.	Yes	.	No	Mild Dementia	.	.
157	165	Yes	Yes	Yes	Yes	.	.	.	Yes	Yes	Yes	Moderate Dementia	Yes	Probable
Total	157	143	67	117	102	69	55	52	39	136	75	157	91	47

Statistical Analyses: Frequencies

Cognitive Impairment

		Frequency	Percent	Valid Percent	Cumulative Percent
Valid	No	46	29.3	32.2	32.2
	Yes	97	61.8	67.8	100.0
	Total	143	91.1	100.0	
Missing	System	14	8.9		
Total		157	100.0		

Executive Function Impairment

		Frequency	Percent	Valid Percent	Cumulative Percent
Valid	No	33	21.0	49.3	49.3
	Yes	34	21.7	50.7	100.0
	Total	67	42.7	100.0	
Missing	System	90	57.3		
Total		157	100.0		

Sum Cognitive Impairment TES

		Frequency	Percent	Valid Percent	Cumulative Percent
Valid	.00	45	28.7	31.3	31.3
	1.00	67	42.7	46.5	77.8
	2.00	32	20.4	22.2	100.0
	Total	144	91.7	100.0	
Missing	System	13	8.3		
Total		157	100.0		

Cognitive Impairment TES

		Frequency	Percent	Valid Percent	Cumulative Percent
Valid	1.00	99	63.1	100.0	100.0
Missing	System	58	36.9		
Total		157	100.0		

Neurobehavioral Dysregulation

		Frequency	Percent	Valid Percent	Cumulative Percent
Valid	No	77	49.0	65.8	65.8
	Yes	40	25.5	34.2	100.0
	Total	117	74.5	100.0	
Missing	System	40	25.5		
Total		157	100.0		

Progressive Course

		Frequency	Percent	Valid Percent	Cumulative Percent
Valid	No	48	30.6	47.1	47.1
	Yes	54	34.4	52.9	100.0
	Total	102	65.0	100.0	
Missing	System	55	35.0		
Total		157	100.0		

Depression

		Frequency	Percent	Valid Percent	Cumulative Percent
Valid	No	52	33.1	75.4	75.4
	Yes	17	10.8	24.6	100.0
	Total	69	43.9	100.0	
Missing	System	88	56.1		
Total		157	100.0		

Suicidality

		Frequency	Percent	Valid Percent	Cumulative Percent
Valid	No	18	11.5	94.7	94.7
	Yes	1	.6	5.3	100.0
	Total	19	12.1	100.0	
Missing	System	138	87.9		
Total		157	100.0		

Anxiety

		Frequency	Percent	Valid Percent	Cumulative Percent
Valid	No	48	30.6	87.3	87.3
	Yes	7	4.5	12.7	100.0
	Total	55	35.0	100.0	
Missing	System	102	65.0		
Total		157	100.0		

Apathy

		Frequency	Percent	Valid Percent	Cumulative Percent
Valid	No	46	29.3	88.5	88.5
	Yes	6	3.8	11.5	100.0
	Total	52	33.1	100.0	
Missing	System	105	66.9		
Total		157	100.0		

Paranoia

		Frequency	Percent	Valid Percent	Cumulative Percent
Valid	No	20	12.7	51.3	51.3
	Yes	19	12.1	48.7	100.0
	Total	39	24.8	100.0	
Missing	System	118	75.2		
Total		157	100.0		

Motor Signs

		Frequency	Percent	Valid Percent	Cumulative Percent
Valid	No	48	30.6	35.3	35.3
	Yes	88	56.1	64.7	100.0
	Total	136	86.6	100.0	
Missing	System	21	13.4		
Total		157	100.0		

Delayed Onset

		Frequency	Percent	Valid Percent	Cumulative Percent
Valid	No	61	38.9	81.3	81.3
	Yes	14	8.9	18.7	100.0
	Total	75	47.8	100.0	
Missing	System	82	52.2		
Total		157	100.0		

Functional Impairment

		Frequency	Percent	Valid Percent	Cumulative Percent
Valid	Independent	36	22.9	22.9	22.9
	Subtle/Mild Functional Limitation	52	33.1	33.1	56.1
	Mild Dementia	35	22.3	22.3	78.3
	Moderate Dementia	32	20.4	20.4	98.7
	Severe Dementia	2	1.3	1.3	100.0
	Total	157	100.0	100.0	

Functional Impairment with Motor Signs

		Frequency	Percent	Valid Percent	Cumulative Percent
Valid	Independent	32	20.4	20.4	20.4
	Subtle/Mild Functional Limitation	52	33.1	33.1	53.5
	Mild Dementia	36	22.9	22.9	76.4
	Moderate Dementia	35	22.3	22.3	98.7
	Severe Dementia	2	1.3	1.3	100.0
	Total	157	100.0	100.0	

Sum of 3 Core Features of TES (cognitive impairment, executive function impairment, and neurobehavioral dysregulation)

		Frequency	Percent	Valid Percent	Cumulative Percent
Valid	.00	44	28.0	29.7	29.7
	1.00	48	30.6	32.4	62.2
	2.00	45	28.7	30.4	92.6
	3.00	11	7.0	7.4	100.0
	Total	148	94.3	100.0	
Missing	System	9	5.7		
Total		157	100.0		

Diagnosed TES

		Frequency	Percent	Valid Percent	Cumulative Percent
Valid	No	44	28.0	48.4	48.4
	Yes	47	29.9	51.6	100.0
	Total	91	58.0	100.0	
Missing	System	66	42.0		
Total		157	100.0		

TES Psychiatric Features

		Frequency	Percent	Valid Percent	Cumulative Percent
Valid	No, Not Present	29	18.5	45.3	45.3
	Yes, Present	35	22.3	54.7	100.0
	Total	64	40.8	100.0	
Missing	System	93	59.2		
Total		157	100.0		

Provisional Levels of Certainty for CTE

		Frequency	Percent	Valid Percent	Cumulative Percent
Valid	Suggestive	1	.6	2.1	2.1
	Possible	15	9.6	31.9	34.0
	Probable	31	19.7	66.0	100.0
	Total	47	29.9	100.0	
Missing	System	110	70.1		
Total		157	100.0		

Article Extractions

Martland (1928)

Case 2 (Age: 38; Sport: Boxing)
Age started: 16 years
Age retired: 23 years
Length of career: 7 years
Age of symptom onset: 23 years
Delay between retirement and symptom onset (Age of symptom onset – Age retired): 0 years*
*No delay

Core Clinical Features

- Cognitive Impairment: 0
  - "The intelligence is normal." (pg. 1106)
- Executive Function Impairment: NM
- Neurobehavioral Dysregulation: NM
- Progressive Course: Progressive
  - "Since 1913 [the year he stopped fighting] **his condition has slowly progressed until he now resembles a well marked case of paralysis agitans**." (pg. 1106)

Supportive Features

- Depression: NM
- Anxiety: NM
- Apathy: NM
- Paranoia: NM
- Suicidality: NM
- Motor Signs (Parkinsonism / ALS): 1
  - "One case of advanced parkinsonian syndrome due to punch drunk." (pg. 1106)
  - "He stopped fighting in 1913, when 23 years of age, because of a tremor in his left hand and an unsteadiness on his legs." (pg. 1106)
  - "On account of symptoms of **tremor and unsteadiness**, he was often wrongly accused of being intoxicated." (pg. 1106)
  - "Since 1913 [the year he stopped fighting] **his condition has slowly progressed until he now resembles a well marked case of paralysis agitans**." (pg. 1106)
  - "His **gait is staggering and propulsive**, his **facial expression masklike**. There is marked **stammering and hesitancy in speech**. He has a **fine tremor in his hands and tongue**. The pupils are equal and react to light. The **knee kicks are slightly exaggerated**. Clonus and Babinski phenomenon are absent. The sensations are normal. The intelligence is normal. He has been under treatment in many clinics for paralysis agitans and told that his fighting did not have anything to do with his present condition." (pg. 1106)
  - "From a neurologic aspect this case is one of paralysis agitans. It either is traumatic in origin or is a primary, essential form of paralysis agitans of the juvenile type." (pg. 1106)
- Delayed Onset of Symptoms and Problems: 0
  - "He stopped fighting in 1913, when 23 years of age, because of a **tremor in his left hand and an unsteadiness on his legs**." (pg. 1106)

Level of Functional Dependence / Dementia: II
Level of Functional Dependence / Dementia (Including Physical Disability): II

Parker (1934)

Case I (Age: 24; Sport: Boxing)
Age started: 15 years
Age retired: Likely 22 years (in 1932)
Length of career: 7 years
Age of symptom onset: Likely 22 years (in 1932)
Delay between retirement and symptom onset (Age of symptom onset – Age retired): 0 years*
*No delay
In the opinion of Parker (1934), the patient sustained a moderate TBI that was responsible for the majority of his symptoms upon presentation.

Core Clinical Features

- Cognitive Impairment: 1
  - "…a little **more forgetful** of names and details." (pg. 22)
- Executive Function Impairment: NM
- Neurobehavioral Dysregulation: NM
- Progressive Course: Non-Progressive
  - "…it is to be noted that **in nearly two years the condition had not become worse nor was there much improvement** beyond a certain point." (pg. 23)
  - "In the first [case], the **maximal functional disability appeared immediately after a fight, improved somewhat during the next few weeks, but thereafter remained at a standstill.**" (pg. 27)

Supportive Features

- Depression: NM
- Anxiety: 1
  - "His mother complained that he was **more nervous since his last fight."** (pg. 22)
- Apathy: NM
- Paranoia: NM
- Suicidality: NM
- Motor Signs (Parkinsonism / ALS): 1
  - "A man, age 24, came to The Mayo Clinic on February 9, 1934, because of **weakness of the legs** and **tremor of the hands.**" (pg. 21)
  - "Two months later, in June, 1932, chance of another fight had come to him, and, in spite of his **poor balance**, he thought to train for it and overcome this difficulty." (pg. 22)
  - "He seemed to be fast enough on his feet in the ring at the beginning, but was unable to endure any considerable length of time, because of a **sense of fatigue of his lower extremities and a tendency of them to drag**." (pg. 22)
  - "When I first saw him he still had trouble in walking, in that his **feet seemed to drag**. The right foot was worse than the left and he wore away the toes of his shoes." The **sense of balance was not normal**, but slightly better than it had been; yet he **frequently staggered**, more at times than at others." By conscious attention to walking he would pick up his feet better than he usually did, but he soon **relapsed into a dragging gait**." His mother complained that he was **more nervous since his last fight**, a little **more forgetful** of names and details, and that his hands shook. Under excitement, **he tended to have a general tremor**." (pg. 22)
  - "He **walked slowly and unsteadily**. His **lower limbs were stiff and slow**, the right being the worse of the two. At the same time, he **staggered on turning suddenly** and was **somewhat ataxic**. He held the **right arm flexed when walking, and stiffly at his side**, and did not swing it like the other. He hopped clumsily on the right foot, but did better with the left. The toes of both shoes were well worn down. There was **a coarse, pill-rolling tremor of both hands,** more marked on the right. **Slight tremor of the head** was present, but speech was unaltered. **Speed was diminished on the right side**, in both the upper and lower extremity, especially for rapid movements like drumming with fingers on a table. **All tendon reflexes were greatly exaggerated,** but there was no Babinski's sign on plantar stimulation. Romberg's sign was positive, and slight, horizontal nystagmus was present. Facial expression was normal and labile." (pg. 22)
  - "No specific nervous syndrome appeared, such as Parkinson's disease." (pg. 23)
- Delayed Onset of Symptoms and Problems: 0

Level of Functional Dependence / Dementia: II

- "In two weeks he returned to his duty as a filling-station attendant… From that time up to his admission at the clinic (1934) his condition had changed little." (pg. 22)
- "There is but little doubt that the severe beating received by this pugilist in his last fight was responsible for the chain of circumstances which followed. Although he finished the fight, **the symptoms which appeared almost immediately thereafter**

suggested at least moderately severe injury to the brain, from which he made very far from complete recovery…**he attributed all his difficulties to his last and futile appearance in the ring**." (pg. 22) In the opinion of Parker (1934), the patient sustained a moderate TBI that was responsible for the majority of his symptoms upon presentation. A more detailed description of this fight and subsequent symptoms is provided here:

- "In April, 1932, the chance of making money from a bout presented itself and he re-entered the ring. He was knocked down twice in the third round, once by a blow over the right side of his jaw and the other time by a blow over the left eye and temple. He took the count of nine and then finished the fight of six round. Immediately after the fight, he felt as usual, but a few minutes later he took a drink of water, which he promptly vomited. He continued to vomit and retch, even on an empty stomach, and an hour after the fight, when attempting to go home, he reeled and staggered as if drunk. He managed to make his way home, and went to bed, but slept poorly because of nausea. Finally he slept and awakened at 10 a.m. When he attempted to arise he found himself very unsteady and was unable to balance himself. He was able to walk after a fashion, and actually had more difficulty in equilibrium than during the previous night. After consulting a physician, he returned to bed, where he stayed for three days, feeling nauseated and complaining of diplopia. He improved somewhat after this rest, and again he arose, but, while the diplopia had disappeared, he was still unsteady, although very much less so than previously. In two weeks he returned to his duty as a filling-station attendant, still slightly ataxic." (pg. 21-22)

Level of Functional Dependence / Dementia (Including Physical Disability): II

Case II (Age: 30; Sport: Boxing)
Age started: 16 years
Age retired: Likely 23 years (in 1921)
Length of career: 7 years
Age of symptom onset: Likely 23 years (in 1921)
Delay between retirement and symptom onset (Age of symptom onset – Age retired): 0 years*
*No delay

Core Clinical Features

- Cognitive Impairment: 1
    - "…and his **powers of mental concentration, attention and memory were considerably reduced**." (pg. 23)
    - **"Mentally the man seemed childish**, and below par even when his social standing and previous education were discounted." (pg. 24)
- Executive Function Impairment: NM
- Neurobehavioral Dysregulation: NM
- Progressive Course: Non-Progressive
    - "[he] **had not become any worse,** and, if anything, there was a slight improvement during the six years that had elapsed since his examination at the clinic." (pg. 24)
    - "All that is known is that after seven years of strenuous fighting and during the course of his career, between one bout and the next, symptoms appeared insidiously, increased in severity, and put an end to his fighting. Even after he had left the ring and had tried other pursuits, **his difficulties increased steadily up to the time he appeared at the clinic, seven years after his last fight**." (pg. 25)
    - "In the second case the **progressively crippling syndrome appeared insidiously during the last few fights of the patient's career**. His difficulties had **progressed up to the time of his examination four years later, but apparently had not become worse, and were, if anything, improved in the subsequent six years**." (pg. 28)

Supportive Features

- Depression: NM
- Anxiety: NM
- Apathy: NM
- Paranoia: NM
- Suicidality: NM
- Motor Signs (Parkinsonism / ALS): 1
    - A man, age 30 years, came to The Mayo Clinic on January 9, 1928, because of **difficulty in walking and in speech**." (pg. 23)
    - "During this period of training he noticed that, when he was skipping rope, **his right leg tended to catch on the rope**, but he took little notice of it at the time." (pg. 23)
    - "The fourth fight was in June, 1921, and it proved to be his last. He was knocked down by the first blow of his opponent, which was a moderately hard one, and when he attempted to get up he was dizzy, had a severe headache, and promptly gave up the fight. While walking home, he found that the difficulty with his right leg had become decidedly noticeable; both lower limbs felt numb and they dragged as he walked. He decided, after this, that he was no longer fit to fight in the ring, and became a labourer in a steel mill. During the first month that elapsed after his last fight, and while he was working in the mill, the **stiffness and slowness of movement of his lower extremities** increased. This was **more marked on the right side**, for, as time went on, he wore out two soles of the right shoe to one of the left. **Walking became increasingly difficult**; he noticed that his **voice had become nasal**, and that articulate **speech was becoming more and more indistinct**." (pg. 23)
    - "In the latter part of 1923, **movements of the right arm became stiff, clumsy, and awkward**. **Spasmodic contractions of the muscles of the right arm, right leg and back appeared and gradually increased** so that in walking he felt a **tendency to be pulled backward**." (pg. 23)
    - "**Through the subsequent four years, his difficulties slowly and gradually increased.** He experienced **more and more difficulty in walking and in speech**, as well as **increasing spasms of the muscles of the extremities and trunk**." (pg. 24)
    - "…various contractions and spasms continuously at play in his muscles. In walking, his **gait was unsteady and spastic**; the **spasticity was more marked on the right** than on the left side. More conspicuous, however, were the **continuous, irregular spasms of the muscles of the right arm and back**. These were sufficiently violent to pull the man backward and to the right, with the right arm jerking and his hand slapping his thigh. These contractions were rapid, ever changing and quickly repeated." When the right arm was extended forward, **fully supinated, continuous jerking occurred**, tending to flex and pronate the arm. When, on the other hand, the arm was drawn behind the trunk in full pronation, these movements ceased. **While the patient was seated the muscles of the back were continuously jerking, pulling him**

backward and to the right." The muscles of the neck and face were quiet. There was no paralysis, but all of the man's **movements were slow and clumsy**, except when he was running and jumping, which acts he performed, on the whole, better than slow walking." When he was asked to perform **rapid movements of the fingers, these movements were slow on the right side**, but **in the left leg and foot the movements were equally slow and spastic**. The **tendon reflexes on the left side were exaggerated,** and plantar stimulation on the left side produced an extensor reflex. Stroking the lips with a hard object-produced a sucking reflex (**Oppenheim's sign**). Tests of coordination, applied to the upper and lower extremities, did not disclose marked dysmetria or dyssynergia, but all tests were interfered with by the **irregular, spasmodic contractions**." There was no nystagmus. **Speech was very indistinct, slow, laboured, grating and nasal in tone**, and the breath escaped through the nose while the man was talking, apparently because of some weakness of his soft palate. During animated conversation, he had the **tendency to break into a silly guffaw**. This, with the mouth wide open and with the tone of voice, seemed to suggest the **spasmodic laugh** seen in pseudobulbar palsy or progressive lenticular degeneration." (pg. 24)
    - "The clinical picture was exceedingly complicated and did not resemble at least the more usual sequelae of epidemic encephalitis. There was first, **evident dystonia**, **producing grotesque muscular contractions**, but the corticospinal motor system was affected also, as shown by the **spasticity and Babinski phenomenon.** There was also the **dysarthria and the spasmodic laugh** to account for." (pg. 25)
- Delayed Onset of Symptoms and Problems: 0

Level of Functional Dependence / Dementia: II
- "He continued at the mill about 18 months, when he was **discharged because his infirmity had become obvious**." (pg. 23)
- "**Through the subsequent four years, his difficulties slowly and gradually increased.** He experienced **more and more difficulty in walking and in speech**, as well as **increasing spasms of the muscles of the extremities and trunk**. At the time of his examination, however, he still was able to walk alone, and he could feed and dress himself." (pg. 24)

Level of Functional Dependence / Dementia (Including Physical Disability): III

Case III (Age: 28; Sport: Boxing)
Age started: 18 years
Age retired: 28 years
Length of career: 10 years
Age of symptom onset: Likely 24 years (in 1919)
Delay between retirement and symptom onset (Age of symptom onset – Age retired): -5 years*
*Symptom onset during career

Core Clinical Features

- Cognitive Impairment: 1
  - "He seemed **below par mentally**; **his general intelligence and his memory were somewhat reduced**." (pg. 26)
- Executive Function Impairment: NM
- Neurobehavioral Dysregulation: NM
- Progressive Course: Unable to Classify
  - "Nevertheless, **remorselessly his disease advanced, speed and precision were slower in appearing with each bout,** and a few days before the patient's first visit to the clinic his career ended in an inglorious fashion." (pg. 26)
  - "…he reappeared on March 7, 1934, **almost exactly eleven years after his first examination**. To our surprise we found that time had dealt gently with him. **He seemed in no way worse**, and beyond acquiring a certain amount of obesity he presented an identically similar picture to that encountered on his first examination, and was, if anything, somewhat improved, discounting the increased weight and lack of training. His disturbance of speech and gait were as conspicuous as before, but he had not deteriorated any more, physically or mentally. He had taken no antisyphilitic treatment in the meantime" (pgs. 26-27)
  - "The patient, a high-grade pugilist, **developed symptoms during a period of four years, coincident with his heaviest fighting. The symptoms slowly progressed up to his final, ignominious failure in the ring, and then, for eleven years thereafter, his condition remained much the same as it was when he ceased fighting.** The picture was also different from that presented in Cases I and II; it was mainly that of injury to the corticospinal motor tracts on both sides; hence the original impression of lateral sclerosis." (pg. 27)
  - "The third patient also gave evidence of **the insidious development of an affliction of the central nervous system appearing five years before he ceased to fight and progressing up to the time of his leaving the ring**. However, **after the patient's fighting career was over his condition remained stationary or perhaps improved slightly**." (pg. 28)
  - "In the case of the third patient, his **difficulties appeared during his fighting career but stopped progressing as soon as he left the ring**. The probability here, that his disease was connected with his occupation, is good." (pg. 28)

Supportive Features

- Depression: NM
- Anxiety: NM
- Apathy: NM
- Paranoia: NM
- Suicidality: NM
- Motor Signs (Parkinsonism / ALS): 1
  - "A man, age 28, came to The Mayo Clinic first on March 23, 1923, because of **stiffness and dragging of his legs in walking, unsteadiness of gait, and indistinct speech**." (pg. 25)
  - "While serving as a mess waiter [in the Army], just before discharge in 1919, he noticed that his **left hand and foot were clumsy**; he spilled glasses of water because the left foot hit the ground awkwardly and the hand shook." (pg. 25)
  - "In 1920 episodes of staggering while walking in the street appeared, and his **speech became thick**, so that he was accused unjustly a number of times of being drunk." (pg. 25)
  - "Gradually, during 1921 and 1922, the **increasing stiffness of the left arm and leg** and the **unsteady gait** became more obvious. He would be slow, awkward and clumsy in the first few rounds." (pgs. 25-26)
  - "The **left arm would go into clonus**, puzzling his opponent by its unexpected and grotesque trepidation." (pg. 26)
  - "When he was examined in 1923 his disability was obvious. His **speech was thick, muffled and hard to understand;** there was a rasping, laboured quality in the voice. His **gait was spastic**. The spasticity was marked on the left side, and he dragged his legs, wearing out the toe of his left shoe. There was no marked weakness. The grip of the left hand was weaker than that of the right, but the outstanding feature was the **slowness and clumsiness of the fingers, hand and arm.** In certain positions the left arm went into clonus." **Patellar clonus** was also present, and there were a few **strokes of ankle clonus** on the left. **Tendon reflexes were everywhere enormously exaggerated**, more so on the left, and on that side a Babinski phenomenon could be elicited. The jaw reflex was exaggerated, and sucking reflexes were present on stroking the lips. The man was unable to hop on the left leg, and on performing active movements, such as shadow-boxing, he was so **stiff and clumsy** that he nearly fell, and his left arm was slow and awkward. The right arm and hand

seemed but little affected. There were no sensory changes, tremors or nystagmus; facial expression was mobile and pupillary reactions were normal." (pg. 26)

    - "A diagnosis was made of lateral sclerosis of the spinal cord, and some doubt was expressed as to the origin of the condition. Syphilis was suspected because of the history, and a course of antisyphilitic treatment was advised on empirical grounds. A very poor prognosis was given, since it was expected that progression of the disease could be expected." (pg. 26)

- Delayed Onset of Symptoms and Problems: 0

Level of Functional Dependence / Dementia: II

- "When he was examined in 1923 his disability was obvious" (pg. 26)
- "…he reappeared on March 7, 1934, **almost exactly eleven years after his first examination**. To our surprise we found that time had dealt gently with him. **He seemed in no way worse**, and beyond acquiring a certain amount of obesity he presented an identically similar picture to that encountered on his first examination, and was, if anything, somewhat improved, discounting the increased weight and lack of training. His disturbance of speech and gait were as conspicuous as before, but he had not deteriorated any more, physically or mentally. He had taken no antisyphilitic treatment in the meantime" (pgs. 26-27)

Level of Functional Dependence / Dementia (Including Physical Disability): III

Critchley (1949)

Case A (Age: 31; Sport: Boxing)
Age started: 10 years
Age retired: Not mentioned
Length of career: Not mentioned
Age of symptom onset: Not mentioned
Delay between retirement and symptom onset (Age of symptom onset – Age retired): Not mentioned

Core Clinical Features
- Cognitive Impairment: NM
- Executive Function Impairment: NM
- Neurobehavioral Dysregulation: NM
- Progressive Course: Unable to Classify

Supportive Features
- Depression: NM
- Anxiety: NM
- Apathy: NM
- Paranoia: NM
- Suicidality: NM
- Motor Signs (Parkinsonism / ALS): 2
    - "An airman, aged 31, had been complaining of **intractable and persistent headaches for some weeks, associated with postural giddiness, and nocturnal restlessness**" (pg. 135)
- Delayed Onset of Symptoms and Problems: NM

Level of Functional Dependence / Dementia: II
- "An **Airman**, aged 31…" (pg. 135)
- "After some of the later contests he would feel 'groggy' and this malaise would last for days. At such times he would notice that his eyesight was temporarily impaired and he could not judge distances properly." (pg. 135). *Note: these symptoms seem to be more typical of acute post-concussion symptoms.*

Level of Functional Dependence / Dementia (Including Physical Disability): II

Case B (Age: 21; Sport: Boxing)
Age started: 14 years
Age retired: Not mentioned
Length of career: Not mentioned
Age of symptom onset: Not mentioned
Delay between retirement and symptom onset (Age of symptom onset – Age retired): Not mentioned

Core Clinical Features

- Cognitive Impairment: 1
  - "Simple demential type of punch-drunk syndrome." (pg. 135)
  - "He revealed **considerable retardation in speech, thought and action**. His facial appearance showed a poverty of expressional movement, and **he appeared dazed and bewildered**. Frequently he **failed to grasp even simple ideas**. He often forgot to finish an action which he had started; thus when told to take off his shoes and socks, he did so on one side and then had to be told to take off the other… he seemed **bemused and unaware of what was expected of him.**" (pg. 136)
  - "He regarded Edward V as the reigning King of England. After a very long pause he named correctly the capital of France. Asked the capital of Germany he replied "Moscow, no that is in Russia…Berlin." (pg. 136)
- Executive Function Impairment: 1
  - "Simple demential type of punch-drunk syndrome." (pg. 135)
  - "He revealed **considerable retardation in speech, thought and action**. His facial appearance showed a poverty of expressional movement, and **he appeared dazed and bewildered**. Frequently he **failed to grasp even simple ideas**. He often forgot to finish an action which he had started; thus when told to take off his shoes and socks, he did so on one side and then had to be told to take off the other… he seemed **bemused and unaware of what was expected of him.**" (pg. 136)
- Neurobehavioral Dysregulation: NM
- Progressive Course: Unable to Classify

Supportive Features

- Depression: NM
- Anxiety: NM
- Apathy: NM
- Paranoia: NM
- Motor Signs (Parkinsonism / ALS): 2
  - "…gave a very inadequate account of **headaches for six months; ringing in the ears; postural giddiness; poor sleep; and a sense of heaviness over the top of the head**." (pg. 135)
  - "…**no dysarthria**. The **tongue deviated to the left**. **All tendon reflexes were rather sluggish**. No other neurological abnormality could be traced." (pg. 136)
- Delayed Onset of Symptoms and Problems: NM

Level of Functional Dependence / Dementia: IV

- "A leading seaman, aged 21…" (pg. 135)

Level of Functional Dependence / Dementia (Including Physical Disability): IV

Case C (Age: 29; Sport: Boxing)
Age started: 13 years
Age retired: Still active
Length of career: Not applicable
Age of symptom onset: Not mentioned
Delay between retirement and symptom onset (Age of symptom onset – Age retired): Symptom onset during career

Core Clinical Features

- Cognitive Impairment: 1
    - "Euphoric dementia in a punch-drunk delinquent." (pg. 136)
    - "He left school at 14 in Standard VII, with an excellent character." (pg. 136)
    - "Was put upon a charge in the following official terms: <<that he had been adrift 2 ½ hours; that he had lost his respirator in a pub; that when spoken to he belched offensively without regard; that he is a general nuisance; not intelligent; a bad influence; and finally that he is evil>>." (pg. 136)
- Executive Function Impairment: NM
- Neurobehavioral Dysregulation: 1
    - "He left school at 14 in Standard VII, with an excellent character." (pg. 136)
    - "Was put upon a charge in the following official terms: <<that he had been adrift 2 ½ hours; that he had lost his respirator in a pub; that when spoken to (by law enforcement) he belched offensively without regard; that he is a general nuisance; not intelligent; a bad influence; and finally that he is evil>>." (pg. 136)
- Progressive Course: Unable to Classify

Supportive Features

- Depression: NM
- Anxiety: 1
    - "For the last two years **he had been drinking rather more,** i.e., about 8 pints of beer a night. His wife said he was good-natured and cheery – perhaps unduly so; he did not mix with others very much; **of late he had been getting nervous."** (pg. 136)
- Apathy: NM
- Paranoia: NM
- Suicidality: NM
- Motor Signs (Parkinsonism / ALS): 1
    - "The main objective feature in his case was a **spastic, stammering type of dysarthria,** and a habit of **talking without opening his mouth sufficiently.**" (pg. 136)
- Delayed Onset of Symptoms and Problems: 0

Level of Functional Dependence / Dementia: III

- "Euphoric dementia in a punch-drunk delinquent." (pg. 136)
- "A stoker, aged 29…" (pg. 136)

Level of Functional Dependence / Dementia (Including Physical Disability): III

Case D (Age: 27; Sport: Boxing)
Age started: 15 years
Age retired: Not mentioned
Length of career: Not mentioned
Age of symptom onset: Not mentioned
Delay between retirement and symptom onset (Age of symptom onset – Age retired): Not mentioned

Core Clinical Features

- Cognitive Impairment: 1
  - "His **memory began to play tricks**; he might lose himself in his own street." (pg. 137)
- Executive Function Impairment: NM
- Neurobehavioral Dysregulation: 1
  - "As a boy, he had been a bright, even-tempered scholar, winning three scholarships and leaving school at 14 in Standard VII." (pg. 136)
  - Critchley describes a consistent pattern of desertion and impulsivity: "At the age of 22 he was charged with inflicting grievous bodily hurt, but was acquitted. Shortly after joining the Royal Navy he was adrift for 24 hours and later he deserted for 14 days. He received 90 days detention. Almost immediately afterwards he deserted for 21 days. Later he deserted a third time and was arrested for theft and sent to a civil jail for three months. He then deserted for seven weeks and married a woman he had met the day before. Immediately after the marriage ceremony he left her and had not seen her since." (pg. 137)
- Progressive Course: Unable to Classify

Supportive Features

- Depression: 1
  - **"At times he was depressed**." (pg. 137)
- Anxiety: NM
- Apathy: NM
- Paranoia: NM
- Suicidality: NM
- Motor Signs (Parkinsonism / ALS): NM
- Delayed Onset of Symptoms and Problems: NM

Level of Functional Dependence / Dementia: III

- "A **stoker**, aged 27…" (pg. 136)

Level of Functional Dependence / Dementia (Including Physical Disability): III

Case E (Age: 20; Sport: Boxing)
Age started: 15 years
Age retired: Not mentioned
Length of career: Not mentioned
Age of symptom onset: Not mentioned
Delay between retirement and symptom onset (Age of symptom onset – Age retired): Not mentioned

Core Clinical Features

- Cognitive Impairment: 1
    - "A stoker, aged 20, described his symptoms in the following terms… 'I **can't think properly** and I **can't remember things**… My **head goes blank at times**…" (pg. 137)
    - "His **memory was extremely bad for both recent and remote events**, and he could not repeat 4 digits. His knowledge of current events was very poor." (pg. 137)
- Executive Function Impairment: NM
- Neurobehavioral Dysregulation: 1
    - "He **resisted with violence** any attempts at further investigation" (pg. 138)
- Progressive Course: Unable to Classify

Supportive Features

- Depression: 0
    - "He was **not unduly emotional or depressed**." (pg. 137)
- Anxiety: NM
- Apathy: NM
- Paranoia: NM
- Suicidality: NM
- Motor Signs (Parkinsonism / ALS): 1
    - "A stoker, aged 20, described his symptoms in the following terms… 'I **can't think properly** and I **can't remember things**. My **speech has been getting bad for 18 months**… I can't talk any quicker than I am now. My **head goes blank at times**. I get **pain over the left eye nearly every morning** when I wake. If I rest it may go away, but I **feel thick-headed all day** long. **I get dizzy** but I have never lost consciousness. **My left ear is slightly deaf and my sight is not good in the left eye**. I cannot give my mind to reading, it makes my head worse; I just look at pictures." (pg. 137)
    - "His **speech was extraordinarily slow and dysarthric**; he talked with a curious over-action of the lips. Cerebration was slow, as also his movements." (pg. 137)
    - "Slight nerve type deafness in the left ear. There was **some facial and ocular apraxia**, as shown by the difficulty in getting him to screw up his eyelids, show his teeth, or follow a moving object. His **hand grips were reduced in power**. There was **no tremor, but the finger-nose test performed awkwardly**. **Tendon reflexes were sluggish**; planters flexor. The **gait was slow, lumbering and shuffling**." (pgs. 137-138)
- Delayed Onset of Symptoms and Problems: NM

Level of Functional Dependence / Dementia: III

- "A stoker, aged 20…" (pg. 137)
- "Insight was fair." (pg. 137)
- "He was invalided from the Service and was lost sight of." (pg. 138)

Level of Functional Dependence / Dementia (Including Physical Disability): III

Case F (Age: 38; Sport: Boxing)
Age started: 16 years
Age retired: Likely 34 or 35 years ("3 or 4 years ago" (pg. 138))
Length of career: Likely 18 or 19 years
Age of symptom onset: Not mentioned
Delay between retirement and symptom onset (Age of symptom onset – Age retired): Not mentioned

Core Clinical Features

- Cognitive Impairment: 1
    - "A stoker, aged 38… complained of **early morning headaches** (3 years); **constant diplopia to the left** (3-4 years); **reeling gait**, as if drunk; **impaired memory**; and **'silly feelings' in the head.**" (pg. 138)
    - "Examination showed him to be a cheery, simple-minded fellow, **slow in cerebration**, with fair insight but with **impaired memory**…" (pg. 138)
- Executive Function Impairment: NM
- Neurobehavioral Dysregulation: NM
- Progressive Course: Unable to Classify

Supportive Features

- Depression: NM
- Anxiety: NM
- Apathy: NM
- Paranoia: NM
- Suicidality: NM
- Motor Signs (Parkinsonism / ALS): 1
    - "Punch-drunk syndrome reminiscent of disseminated sclerosis." (pg. 138)
    - "A stoker, aged 38… complained of **early morning headaches** (3 years); **constant diplopia to the left** (3-4 years); **reeling gait**, as if drunk; **impaired memory**; and **'silly feelings' in the head.**" (pg. 138)
    - "Examination showed him to be a cheery, simple-minded fellow, **slow in cerebration**, with fair insight but with **impaired memory.** The **right pupil was greater than the left**. **On left ocular deviation, the right eye turned up and did not turn inwards fully, while the left eye gave one or two slow nystagmoid jerks. On looking right, the left eye did not move in completely. Alternating movements of the upper limbs were carried out slowly and clumsily,** especially on the left. There was **no loss of power; no tremor; no inc[o]ordination.** The **gait was rolling, and the stance unsteady.** All **tendon reflexes were exaggerated**, the knee-jerks being pendular. The **abdominals were sluggish in the left upper quadrant**. **The right plantar response was equivocal, the left one being probably extensor in type**." (pg. 138)
- Delayed Onset of Symptoms and Problems: NM

Level of Functional Dependence / Dementia: III

- "A stoker, aged 38…" (pg. 138)
- "He retired from the ring, and took on his father's job which he described as <<pennies from heaven>>. By this he referred to the profession of a barrow boy." (pg. 138)
- "Examination showed him to be a cheery, simple-minded fellow, **slow in cerebration**, with fair insight but with **impaired memory." (pg. 138)**

Level of Functional Dependence / Dementia (Including Physical Disability): III

Case G (Age: 25; Sport: Boxing)
Age started: 13 years (professional at 17)
Age retired: 23 years (“two years ago, he gave up the ring” (pg. 138))
Length of career: Likely 10 years
Age of symptom onset: Not mentioned
Delay between retirement and symptom onset (Age of symptom onset – Age retired): Not mentioned

Core Clinical Features
- Cognitive Impairment: NM
- Executive Function Impairment: NM
- Neurobehavioral Dysregulation: NM
- Progressive Course: Unable to Classify

Supportive Features
- Depression: NM
- Anxiety: 2
    - “Punch-drunk syndrome associated with recurrent hysterical blindness.” (pg. 138)
    - “A stoker, aged 25, complained of **six attacks of blindness during the past four years**; the first and longest attack had lasted two months, and the shortest one only a few hours.” (pg. 138)
    - “Later on, while still in hospital, his blindness returned, possibly as the result of receiving an anonymous letter impugning the fidelity of his wife.” (pg. 139)
    - He could deviate his eyes in any direction on command, but there was a tendency towards a convergence-spasm. His optic discs were normal.” (pg. 139)
- Apathy: NM
- Paranoia: NM
- Suicidality: NM
- Motor Signs (Parkinsonism / ALS): NM
    - “**Neurological examination, including an E.E.G., proved negative.**” (pg. 139)
- Delayed Onset of Symptoms and Problems: NM

Level of Functional Dependence / Dementia: II
- “A stoker, aged 25…” (pg. 138)

Level of Functional Dependence / Dementia (Including Physical Disability): II

Raevuori-Nallinma (1950)

Case 1 (Age: 33; Sport: Boxing)
Age started: Not mentioned ("since his youth" (pg. 53))
Age retired: Not mentioned
Length of career: 10 years
Age of symptom onset: Not mentioned
Delay between retirement and symptom onset (Age of symptom onset – Age retired): Not mentioned

Core Clinical Features
- Cognitive Impairment: 1
  - "The **patient himself complained of his bad memory**. **When speaking, he sometimes had difficulty in finding words, and when writing, in finding capital letter**s, especially the letter N. Objectively, too, **a slight decrease of memory could be ascertained**. **Intellectually he proved quite normal**." (pg. 54)
- Executive Function Impairment: NM
- Neurobehavioral Dysregulation: 0
  - "In recent years has become **irritable and easily falls out with others**." (pg. 53)
  - "Has slept badly, the nervous system continually sensitive, the patient is **easily irritated and loses patience**." (pg. 54)
  - **"**His behaviour was quiet and matter-of-fact. **It is true the patient himself complained of irritability, but was, however, able to control himself outwardly."** (pg. 54)
- Progressive Course: Non-Progressive
  - **"During a hospital observation of 5 months there were no changes to speak of in his condition, save that the headache grew less**. After leaving the hospital he took up manual work lighter than his former job." (pg. 54)

Supportive Features
- Depression: NM
- Anxiety: 1
  - "Has slept badly, the nervous system continually sensitive, the patient is **easily irritated and loses patience**." (pg. 54)
- Apathy: NM
- Paranoia: NM
- Suicidality: NM
- Motor Signs (Parkinsonism / ALS): 2
  - **"Other neurological symptoms - including balance disturbances could not be ascertained**." (pg. 54)
- Delayed Onset of Symptoms and Problems: NM

Level of Functional Dependence / Dementia: II
- "A chauffeur of 33 years…" (pg. 53)
- **"During a hospital observation of 5 months there were no changes to speak of in his condition, save that the headache grew less**. After leaving the hospital he took up manual work lighter than his former job." (pg. 54)

Level of Functional Dependence / Dementia (Including Physical Disability): II

Case 2 (Age: 37; Sport: Boxing)
Age started: 17 years
Age retired: 33 (Had not boxed for four years prior to visit, at age 37)
Length of career: 16 years
Age of symptom onset: Not mentioned (in 1938)
Delay between retirement and symptom onset (Age of symptom onset – Age retired): Not mentioned

Core Clinical Features

- Cognitive Impairment: 1
  - "**Thinking has become slower** than before. **Memory grown worse**." (pg. 54)
  - "Psychically the patient makes a primitive impression. **Clearly seems demented. Thinking and reaction slow and heavy**." (pg. 54)
  - "Is only with difficulty able to talk about his own life. **Memory is perceptibly weakened**, so that he in the ten words test is not even at the tenth time able to repeat more than 0 words, and after 5 minutes remembers 8 words. **The intellectual stage defined according to Binet-Bobertag 13 years 4 months**." (pg. 54)
- Executive Function Impairment: NM
  - "In Kraepelin's addition test the slackening of mental activity proves less than expected, so that in 10 minutes' time the patient does on average 18.4 sums a minute." (pg. 54)
- Neurobehavioral Dysregulation: NM
- Progressive Course: Progressive
  - "The condition of the patient did not change during a hospital observation of more than a month. Because of his illness he was entirely released from military service." (pgs. 54-55)
  - "In the latter case we have a typical boxer's dementia, "punch-drunkenness." It is noteworthy that **the symptoms here had clearly increased during the last 4 years,** when the patient had no longer boxed. **Besides psychical changes, there were also neurological focal symptoms.**" (pg. 55)

Supportive Features

- Depression: NM
- Anxiety: NM
- Apathy: 1
  - **"The expressions of the face slack and dull, the look heavy and somnolent.** The patient is **apathetic, unable to take the initiative, and indifferent. The speech slow and indistinct**." (pg. 54)
- Paranoia: NM
- Suicidality: NM
- Motor Signs (Parkinsonism / ALS): 1
  - **"During 2 years the speech has been indistinct and by degrees grown worse**. The **throat feels stiff and sometimes he gets "water in the throat" at night**." (pg. 54)
  - **"Neurologically symptoms of a left side hemiplegia are verified.** When walking, the left leg stiffer than the right one. The movements of the left upper and lower limb stiffer and clumsier than those of the right one, which is seen also in the diadochokinetic movements. The muscular strength of the left upper and lower limb weakened. The left side stomach and cremaster reflexes weaker than the right side ones. The left side knee and achilles jerks more active than usual. In Romberg's test he sways perceptibly, in Fournier's test likewise. In position reflexes the left hand falls lower. Pain sensibility in the left upper limb, in the left half of the neck, head and face somewhat decreased. Otherwise no sensibility disturbances." (pg. 54)
  - **"About the cranial nerves it may be mentioned that the patient says he sees double pictures when looking to either side and up sidewards. The convergence reaction weakened.** The lower and partly the middle branch of the left facial nerve function more faintly than the right one. Uvula wound and withdrawn to the left. The left faucial pillar more contracted and when intoning it places itself higher. **The articulation is very indistinct and stiff, like that of an intoxicated person**. Slight "Silbenstolpern". In the liquor no pathological changes. **A slight lability of the pulse appear**. (pg. 54-55)
  - **"Besides psychical changes, there were also neurological focal symptoms.**" (pg. 55)
- Delayed Onset of Symptoms and Problems: NM

Level of Functional Dependence / Dementia: IV

- "A worker of 37 years…" (pg. 54)
- "The condition of the patient did not change during a hospital observation of more than a month. Because of his illness he was entirely released from military service." (pgs. 54-55)

Level of Functional Dependence / Dementia (Including Physical Disability): IV

Critchley (1957)

Note: Victoroff (2013) identified and included 6 cases (cases 2, 3, 8-11) of what Critchley (1957) refers to as "traumatic progressive encephalopathy" from Critchley's (1957) article. Critchley (1957) included a total of 21 cases, 11 of which he identified as examples of the "punch-drunk state" (i.e., traumatic progressive encephalopathy; cases 1-11), and 10 of which he identified as example of a "groggy state". Consistent with Victoroff (2013), we excluded all cases that Critchley (1957) identified as example of the "groggy state" except Case 14. Critchley (1957) notes that Case 14 had been diagnosed with "punch-drunkenness." Victoroff excluded Critchley's (1957) Case 4. Consistent with Victoroff (2013), we also excluded this case. Case 4 appears to be a case of epilepsy and he is described as such by Critchley: "clinical examination was negative, but the E.E.G. was abnormal, with bilateral synchronous rhythmic slow-wave discharges suggestive of idiopathic epilepsy." (pgs. 359-360)

Note: Critchley (1957) introduces cases 1-11 with the statement "the following clinical notes illustrate some of the characteristics of traumatic progressive encephalopathy in boxers." (pg. 359) We accepted all cases as having a progressive course even though some had insufficient details to classify them as such.

Case 2 (Age: not provided; Sport: Boxing)
Age started: Not mentioned
Age retired: Not mentioned
Length of career: Not mentioned
Age of symptom onset: Not mentioned
Delay between retirement and symptom onset (Age of symptom onset – Age retired): No delay

Core Clinical Features
- Cognitive Impairment: 1
    - "It was noticed by others that **he was becoming a little slower, and the speech somewhat thick.** On the occasion of his last contest he was knocked out, and headaches thereafter became troublesome. Although the patient maintained that he felt well, he was **noticeably dysarthric and slow in cerebration.** There was a strong suspicion here of an early punch-drunkenness and he was advised to rest." (pg. 359)
- Executive Function Impairment: NM
- Neurobehavioral Dysregulation: NM
- Progressive Course: Progressive

Supportive Features
- Depression: NM
- Anxiety: NM
- Apathy: NM
- Paranoia: NM
- Suicidality: NM
- Motor Signs (Parkinsonism / ALS): 1
    - "**This boxer, who was always liable to headaches after each contest, had found them more persistent in the last few months.** It was noticed by others that **he was becoming a little slower, and the speech somewhat thick.** On the occasion of his last contest he was knocked out, and headaches thereafter became troublesome. Although the patient maintained that he felt well, he was **noticeably dysarthric and slow in cerebration.** There was a strong suspicion here of an early punch-drunkenness and he was advised to rest." (pg. 359)
- Delayed Onset of Symptoms and Problems: 0

Level of Functional Dependence / Dementia: II
- "Incipient Punch-drunk Disability in a First-class Heavyweight." (pg. 359)
- "There was strong suspicion here of an early punch-drunkenness and he was advised to rest. In this view the British Boxing Board of Control concurred, but the U.S. authorities dissented. They insisted on his fulfilling his American contracts, but thereafter **he rapidly deteriorated both medically and professionally**, and the diagnosis of **punch-drunkenness** was ultimately agreed upon." (pg. 359)

Level of Functional Dependence / Dementia (Including Physical Disability): II

Case 3 (Age: 61; Sport: Boxing)
Age started: Not mentioned
Age retired: Not mentioned
Length of career: 16 years (professional lightweight from 1910-1916)
Age of symptom onset: Not mentioned
Delay between retirement and symptom onset (Age of symptom onset – Age retired): Not mentioned

Core Clinical Features

- Cognitive Impairment: 1
  - "Mild Case of Punch-drunkenness with Dementia, Euphoria, and Dysarthria." (pg. 359)
  - "When examined he was complaining of **poor powers of concentration and forgetfulness.** He would lose the thread in his conversation and had to rely on written memoranda." (pg. 359)
- Executive Function Impairment: NM
- Neurobehavioral Dysregulation: NM
  - **"His mood was extremely euphoric…"** (pg. 359)
- Progressive Course: Progressive

Supportive Features

- Depression: 0
  - **"His mood was extremely euphoric…"** (pg. 359)
- Anxiety: NM
- Apathy: NM
- Paranoia: NM
- Suicidality: NM
- Motor Signs (Parkinsonism / ALS): NM
  - **"…his speech grossly dysarthric**." (pg. 359)
- Delayed Onset of Symptoms and Problems: NM

Level of Functional Dependence / Dementia: II

- "Mild Case of Punch-drunkenness with Dementia, Euphoria, and Dysarthria." (pg. 359)

Level of Functional Dependence / Dementia (Including Physical Disability): II

Case 8 (Age: 27; Sport: Boxing)
Age started: 16 years
Age retired: Likely 27 years
Length of career: Likely 11 years
Age of symptom onset: 27 years
Delay between retirement and symptom onset (Age of symptom onset – Age retired): 0 years*
*No delay

Core Clinical Features

- Cognitive Impairment: 1
    - "…On examination **he was found to be slightly forgetful**." (pg. 360)
- Executive Function Impairment: NM
- Neurobehavioral Dysregulation: NM
- Progressive Course: Progressive

Supportive Features

- Depression: NM
- Anxiety: NM
- Apathy: NM
- Paranoia: NM
- Suicidality: NM
- Motor Signs (Parkinsonism / ALS): 1
    - "Later he appeared in boxing booths, for it was becoming increasingly obvious to him that he was deteriorating. About this time - that is, when aged 27 years - he was rejected at the recruiting office, and **he realized then that his sight was poor and that his gait was unsteady**. **Three months later his hands became tremulous**. His symptoms increased up to a point **and then remained stationary**. On examination **he was found to be slightly forgetful**. **He had a nasal type of dysarthria, his face was expressionless, and he tended to dribble**. He had a **tremor of the outstretched hands, some increase in muscle tonus, and slight but definite ataxia**. **Tendon-jerks were increased** and there was a **right extensor response**. The **gait was titubant, short-steppage, and stiff**." (pg. 360).
- Delayed Onset of Symptoms and Problems: 0

Level of Functional Dependence / Dementia: II

- "Striatal Type of Punch-drunk Syndrome." (pg. 360)
- "About this time - that is, when aged 27 years - he was rejected at the recruiting office, and **he realized then that his sight was poor and that his gait was unsteady**..." (pg. 360)

Level of Functional Dependence / Dementia (Including Physical Disability): II

Case 9 (Age: 37; Sport: Boxing)
Age started: 21 years
Age retired: 31 years
Length of career: 10 years
Age of symptom onset: Not mentioned
Delay between retirement and symptom onset (Age of symptom onset – Age retired): Not Mentioned

Core Clinical Features
- Cognitive Impairment: 1
    - "Appropriate tests brought to light **a mild degree of intellectual deterioration**." (pg. 360)
- Executive Function Impairment: NM
- Neurobehavioral Dysregulation: NM
- Progressive Course: Progressive

Supportive Features
- Depression: NM
- Anxiety: NM
- Apathy: NM
- Paranoia: NM
- Suicidality: NM
- Motor Signs (Parkinsonism / ALS): 1
    - "He did not think he came to much harm until a particular fight in the Albert Hall when-being distracted by a spectator calling out his name-he was knocked down. He got up after a short count and was floored a second time. This state of affairs went on again and again, and though he finished the fight he subsequently remembered very little of what happened. His symptoms consisted of **progressively increasing tremor and clumsiness of the right upper limb**. Examination revealed a **slight dysarthria, bilateral intention tremor, increased tendon-jerks, but flexor plantar responses**." (pg. 360)
- Delayed Onset of Symptoms and Problems: NM
    - Note: The period of time between a particular fight and the onset of his symptoms is not explicitly stated. If this was his last fight, at age 31, and he was seen at age 37, the time period would be 6 years. However, it is not stated whether he continued to box after this encounter, and his signs and symptoms were due more so to repeated brain injuries.

Level of Functional Dependence / Dementia: II
- "Cerebellar Type of Punch-drunkenness, reminiscent of Disseminated Sclerosis." (pg. 360)

Level of Functional Dependence / Dementia (Including Physical Disability): II

Case 10 (Age: 27; Sport: Boxing)
Age started: Not mentioned
Age retired: Not mentioned
Length of career: Not mentioned
Age of symptom onset: 24 years
Delay between retirement and symptom onset (Age of symptom onset – Age retired): Not mentioned

Core Clinical Features

- Cognitive Impairment: NM
- Executive Function Impairment: NM
- Neurobehavioral Dysregulation: 1
    - "In 1948 (aged 24) after losing his temper with his fiancée, he started to tremble all over." (pg. 360)
- Progressive Course: Progressive

Supportive Features

- Depression: NM
- Anxiety: NM
- Apathy: NM
- Paranoia: NM
- Suicidality: NM
- Motor Signs (Parkinsonism / ALS): 1
    - "In 1948 (aged 24) after losing his temper with his fiancée, he started to tremble all over. **This subsided except in the left hand, where the tremor persisted with increasing amplitude**. **After six months it involved the head, and his leg began to drag**. **His articulation altered**. Examination revealed a **fine tremor of the head, which was held in a curious retracted posture**. The **speech was dysarthric and staccato**. **On the left side there was an unusual type of intention tremor, associated with dysdiadochokinesis**. **The gait showed a steppage abnormality of the left leg. Both plantar responses were extensor in type.**" (pg. 360)
- Delayed Onset of Symptoms and Problems: NM

Level of Functional Dependence / Dementia: II

- "Striato-cerebellar Type of Punch-drunkenness." (pg. 360)

Level of Functional Dependence / Dementia (Including Physical Disability): II

Case 11 (Age: 39; Sport: Boxing)
Age started: Not mentioned
Age retired: Not mentioned
Length of career: Not mentioned
Age of symptom onset: Not mentioned
Delay between retirement and symptom onset (Age of symptom onset – Age retired): Not mentioned

Core Clinical Features
- Cognitive Impairment: 1
    - "**Originally of superior intelligence, he later showed both an intellectual falling-off…**" (pg. 360)
- Executive Function Impairment: 1
    - "…**he later showed both an intellectual falling-off and a curious alteration in personality**." (pg. 360)
- Neurobehavioral Dysregulation: 1
    - "…**he later showed both an intellectual falling-off and a curious alteration in personality**." (pg. 360)
- Progressive Course: Progressive

Supportive Features
- Depression: NM
- Anxiety: NM
- Apathy: NM
- Paranoia: NM
- Suicidality: NM
- Motor Signs (Parkinsonism / ALS): 1
    - "A man aged 39, who had been four times schoolboy champion of Great Britain, **began to develop tremor of the arm during the war,** on which account he was invalided from the Army. **His symptoms increased slightly and he showed an impassive, rather mask-like face; marked dysarthria; titubation of the head, with some hypertrophy of the muscles of the neck; incoordination of the arms and legs, more so on the left; with intention tremor and dysdiadochokinesis**." (pg. 360)
- Delayed Onset of Symptoms and Problems: NM

Level of Functional Dependence / Dementia: II
- "Cerebellar Type of Punch-drunkenness in an Amateur Boxer." (pg. 360)

Level of Functional Dependence / Dementia (Including Physical Disability): II

Case 14* (Age: not provided; Sport: Boxing)
Age started: Not mentioned
Age retired: Not mentioned
Length of career: Not mentioned
Age of symptom onset: Not mentioned
Delay between retirement and symptom onset (Age of symptom onset – Age retired): Not mentioned
*Note: The clinical details for this case were very limited. However, it was included in this review because the case was described as a case of punch drunk by the authors.

Core Clinical Features

- Cognitive Impairment: 1
    - "Latterly he took longer and longer to recover from each fight, **because of headaches, defective memory, and impaired speech**." (pg. 361)
- Executive Function Impairment: NM
- Neurobehavioral Dysregulation: 0
    - "**Ex-lightweight professional boxer with punch-drunkenness in which headaches and irritability were conspicuous**." (pg. 361)
- Progressive Course: Unable to Classify

Supportive Features

- Depression: NM
- Anxiety: NM
- Apathy: NM
- Paranoia: NM
- Suicidality: NM
- Motor Signs (Parkinsonism / ALS): 2
    - "Latterly he took longer and longer to recover from each fight, **because of headaches, defective memory, and impaired speech**." (pg. 361)
- Delayed Onset of Symptoms and Problems: NM

Level of Functional Dependence / Dementia: II
Level of Functional Dependence / Dementia (Including Physical Disability): II

Corsellis & Brierley (1959)

Case 1 (Age: 56; Sport: Boxing)
Age started: 14 years
Age retired: Not mentioned
Length of career: Not mentioned
Age of symptom onset: Not mentioned
Delay between retirement and symptom onset (Age of symptom onset – Age retired): Not mentioned

Core Clinical Features

- Cognitive Impairment: 1
    - No clinical features, as described at the beginning of this document, are reported. Only post-mortem neuropathological findings are reported. The authors note "one was that of a man of 56 in whom a study of the brain was particularly requested because 'the patient's experience as a boxer may have contributed to his dementia.' (pg. 719)
    - "At necropsy… the only localized lesion was a small scar in the corpus callosum (presumably a tear due to past injury). A moderate degree both of cortical atrophy and of hydrocephalus was present. The essential histological findings were the same as those seen in Alzheimer's disease." (pg. 719)
    - Per Corsellis, Bruton, & Freeman-Browne, 1973 article where this case was also reported, "After his discharge a few years later [after rejoining the Navy when aged 43] he **became 'more vague' and 'spoke more slowly'**. He **began to neglect his appearance** and he **complained that he could not grip things or get on a bus**. His **sight and his memory seemed a little faulty**." (pg. 285)
    - "He **wandered away bemused and had to be taken to a mental hospital. He was then 53**. On admission he was **mildly confused and complained of blackouts**. His wife, from whom he was separated, but who was still concerned for him, said that **his personality had been changing for the worse.** A brief **neurological report confirmed this and mentioned a deterioration of habits**. He **could not recall his address or the date**… **During the next year his memory deteriorated further, he was completely disorientated and needed help with dressing**. These features were emphasized in a detailed neurological investigation… He **could not undress himself**." (pg. 285)
    - "…it was concluded that **'the cortical atrophy and dementia may be related to his previous experiences as a boxer.'**" (pg. 285)
- Executive Function Impairment: 1
    - Per Corsellis, Bruton, & Freeman-Browne, 1973 article where this case was also reported, "He **wandered away bemused and had to be taken to a mental hospital. He was then 53**. On admission he was **mildly confused and complained of blackouts**. His wife, from whom he was separated, but who was still concerned for him, said that **his personality had been changing for the worse.** A brief **neurological report confirmed this and mentioned a deterioration of habits**. He **could not recall his address or the date**. No incoordination was found but a **'little dyspraxia'** was queried. **During the next year his memory deteriorated further, he was completely disorientated and needed help with dressing**. These features were emphasized in a detailed neurological investigation. In addition his **speech was slurred, and like his movements was slow and tremulous**. He **could not undress himself**." (pg. 285)
- Neurobehavioral Dysregulation: 1
    - Per Corsellis, Bruton, & Freeman-Browne, 1973 article where this case was also reported, "He had never drunk alcohol excessively but **he now became violent after two pints of beer, sometimes 'fighting with his wife**.'" (pg. 285)
    - "His wife, from whom he was separated, but who was still concerned for him, said that **his personality had been changing for the worse.** A brief **neurological report confirmed this and mentioned a deterioration of habits**." (pg. 285)
- Progressive Course: Progressive

Supportive Features

- Depression: NM
- Anxiety: NM
- Apathy: NM
- Paranoia: 1
    - Per Corsellis, Bruton, & Freeman-Browne, 1973 article where this case was also reported, "He became **incontinent, paranoid, and aggressive**…" (pg. 285)
- Suicidality: NM
- Motor Signs (Parkinsonism / ALS): 2
    - Per Corsellis, Bruton, & Freeman-Browne, 1973 article where this case was also reported, "No incoordination was found but a **'little dyspraxia'** was queried. **During the next year his memory deteriorated further, he was completely disorientated and needed help with dressing**. These features were emphasized in a detailed neurological investigation.

In addition his **speech was slurred, and like his movements was slow and tremulous**. He **could not undress himself**." (pg. 285)
    - "The **deterioration continued**. He became **incontinent, paranoid, and aggressive**. He ate off the table with his fingers' a cigarette packet he called a flower; he undid buttons when asked to put his tongue out. He continued to have blackouts and a few days after a convulsion he died from bronchopneumonia, aged 57 years." (pg. 285)
- Delayed Onset of Symptoms and Problems: NM

Level of Functional Dependence / Dementia: IV
- "At necropsy… the only localized lesion was a small scar in the corpus callosum (presumably a tear due to past injury). A moderate degree both of cortical atrophy and of hydrocephalus was present. The essential histological findings were the same as those seen in Alzheimer's disease." (pg. 719)
- Per Corsellis, Bruton, & Freeman-Browne, 1973 article where this case was also reported, "After leaving the Navy he worked for several years in the police, followed by a period in the docks. His daughter first remembered him some years later when he was a labourer employed by the local council." (pg. 285)
- "He rejoined the Navy when aged 43 and trained cadets during the war." (pg. 285)
- "He worked as a milk roundsman, a bus conductor, and later as a machine operator. When he could no longer manage this, the firm transferred him to labouring and caretaking. After two years he was given notice without warning." (pg. 285)
- "…it was concluded that **'the cortical atrophy and dementia may be related to his previous experiences as a boxer.'**" (pg. 285)
- "The **deterioration continued**. He became **incontinent, paranoid, and aggressive**. He ate off the table with his fingers' a cigarette packet he called a flower; he undid buttons when asked to put his tongue out. He continued to have blackouts and a few days after a convulsion he died from bronchopneumonia, aged 57 years." (pg. 285)

Level of Functional Dependence / Dementia (Including Physical Disability): IV

Case 2 (Age: 63; Sport: Boxing)
Age started: Not mentioned
Age retired: Not mentioned
Length of career: Not mentioned
Age of symptom onset: about 40 years
Delay between retirement and symptom onset (Age of symptom onset – Age retired): Not mentioned

Core Clinical Features

- Cognitive Impairment: 1
  - "A man of 63 with a history of past championship boxing and **severe memory defect** for twenty years was admitted with a diagnosis or 'Organic Dementia, presenile or punch-drunk.' Again, after death four years later, the salient features were those of Alzheimer's Disease, although in addition there was also a haemorrhage destroying the palladium on one side, with considerable hyaline thickening of the smaller vessels throughout the hemispheres." (pg. 720)
  - Per Corsellis, Bruton, & Freeman-Browne, 1973 article where this case was also reported, "[His second wife] **first noticed that his memory was poor when, aged about 40**, he was serving in her shop." (pg. 283)
  - "At the **age of 60 he developed a left hemiparesis and was admitted to a mental hospital three years later**. He was **disorientated** and had **a marked loss of recent memory**. He was **paranoid and deluded, and became confused and aggressive**." (pg. 283)
- Executive Function Impairment: NM
- Neurobehavioral Dysregulation: 1
  - Per Corsellis, Bruton, & Freeman-Browne, 1973 article where this case was also reported, "He and his wife had **drinking bouts** and he was **known in his locality as a violent man**, although 'he had been a good husband'. At the **age of 60 he developed a left hemiparesis and was admitted to a mental hospital three years later**. He was **disorientated** and had **a marked loss of recent memory**. He was **paranoid and deluded, and became confused and aggressive**." (pg. 283)
- Progressive Course: Progressive

Supportive Features

- Depression: NM
- Anxiety: NM
- Apathy: NM
- Paranoia: 1
  - Per Corsellis, Bruton, & Freeman-Browne, 1973 article where this case was also reported, "He was **paranoid and deluded, and became confused and aggressive**." (pg. 283)
- Suicidality: NM
- Motor Signs (Parkinsonism / ALS): 1
  - Per Corsellis, Bruton, & Freeman-Browne, 1973 article where this case was also reported, "At the **age of 60 he developed a left hemiparesis and was admitted to a mental hospital three years later**…"(pg. 283)
- Delayed Onset of Symptoms and Problems: NM

Level of Functional Dependence / Dementia: V

- "A man of 63 with a history of past championship boxing and **severe memory defect** for twenty years was admitted with a diagnosis or 'Organic Dementia, presenile or punch-drunk.' Again, after death four years later, the salient features were those of Alzheimer's Disease, although in addition there was also a haemorrhage destroying the palladium on one side, with considerable hyaline thickening of the smaller vessels throughout the hemispheres." (pg. 720)
- Per Corsellis, Bruton, & Freeman-Browne, 1973 article where this case was also reported, "She first noticed that his memory was poor when, aged about 40, he was serving in her shop. Later he worked as a labourer, and became unemployed for five years after the loss of an eye (unrelated to boxing). He returned to work as a road sweeper." (pg. 283)
- "The **diagnosis was of cerebral arteriosclerosis and organic dementia, probably post-traumatic in origin**. The term **'punch-drunk'** was used. He **gradually deteriorated over the next four years** and died aged 67." (pg. 283)

Level of Functional Dependence / Dementia (Including Physical Disability): V

Neubuerger, Sinton, & Denst (1959)

Case 1 (Age: 46; Sport: Boxing)
Age started: 14; Age retired: 36; Length of career: 22 years
Age of symptom onset: 46 years (Note: this is only when symptoms were first noted medically)
Delay between retirement and symptom onset (Age of symptom onset – Age retired): 10 years

Core Clinical Features

- Cognitive Impairment: 1
  - "Simple tests of mental function showed inability to recall five digits forward, to subtract 7 from 100 serially, to perform any but the simplest calculations, and to recall presidents and dates of world wars. **Psychologic tests revealed loss of immediate recall and of the ability to learn new tasks. The full-scale intelligence performance was 84**." (pg. 20/404)
  - "There was clinical and electroencephalographic evidence of **slowly progressive mental deterioration**." (pg. 22/406)
- Executive Function Impairment: 0
  - "Simple tests of mental function showed inability to recall five digits forward, to subtract 7 from 100 serially, to perform any but the simplest calculations, and to recall presidents and dates of world wars. **Psychologic tests revealed loss of immediate recall and of the ability to learn new tasks. The full-scale intelligence performance was 84**." (pg. 20/404)
- Neurobehavioral Dysregulation: 1
  - "It was noted incidentally that **he was tense, tremulous, suspicious, and irritable, with some delusory phenomena**." (pg. 19/403)
- Progressive Course: Progressive
  - "There was clinical and electroencephalographic evidence of **slowly progressive mental deterioration**." (pg. 22/406)

Supportive Features

- Depression: NM
- Anxiety: 1
  - "It was noted incidentally that **he was tense, tremulous, suspicious, and irritable, with some delusory phenomena**." (pg. 19/403)
- Apathy: NM
- Paranoia: 1
  - "It was noted incidentally that **he was tense, tremulous, suspicious, and irritable, with some delusory phenomena**." (pg. 19/403)
- Suicidality: NM
- Motor Signs (Parkinsonism / ALS): 1
  - "**Neurologic examination revealed only paresis of upward gaze**. **Four years later he returned because of persistent bilateral frontal and temporal headaches**… **From the age of 50 to 53, he was observed frequently, continuing to complain of headaches, occasional blackout spells, and tremulousness**." (pg. 19/403)
  - "Recent examinations revealed an **intermittent fine, rapid tremor of the head and neck, which occasionally spread to the arms, particularly the right. A slight increase in tone was noted in the right arm, along with some decrease in ability to perform rapid alternating movements. Strength, tendon reflexes, coordination, and sensation were normal. There was a defect of upward gaze."** (pg. 19/403)
- Delayed Onset of Symptoms and Problems: 1
  - "A 46-year-old white man was seen first for a minor coronary complaint. It was noted incidentally that **he was tense, tremulous, suspicious, and irritable, with some delusory phenomena**. **Neurologic examination revealed only paresis of upward gaze**. **Four years later he returned because of persistent bilateral frontal and temporal headaches**. At this time a long history of boxing was elicited. He had boxed from the age of 14 to 36 and had 130 professional fights as a light heavy or heavyweight. He was knocked out 30 times. After this, he traveled with a circus as a fighter who would "take on all comers." He claimed never to have been knocked down during this time, but was frequently "out on my feet." **From the age of 50 to 53, he was observed frequently, continuing to complain of headaches, occasional blackout spells, and tremulousness…"** (pg. 19/403)

Level of Functional Dependence / Dementia: III

- **"From the age of 50 to 53, he was observed frequently, continuing to complain of headaches, occasional blackout spells, and tremulousness**. He was unable to work consistently, but held odd jobs, such as night clerk in "skid-row" hotels." (pg. 19/403)

Level of Functional Dependence / Dementia (Including Physical Disability): III

Case 2 (Age: 53; Sport: Boxing)
Age started: 18 years
Age retired: 24 years
Length of career: 6 years
Age of symptom onset: Likely 49 years* (in the year following his admission at age 48 for cholecystectomy, see below)
Delay between retirement and symptom onset (Age of symptom onset – Age retired): 25 years
*Note: This does not include the 'paralyzed left side' that he suffered in one of his last fights.

Core Clinical Features
- Cognitive Impairment: 1
  - "His wife stated that **during the following year he became forgetful, confused, irritable, and moody**. He was examined at the Mayo Clinic, where the following observations were recorded: He was an **affable, alert, restless patient, disoriented as to time and place, able to perform only the simplest calculations, and unable to find his way about Rochester unescorted.**" (pg. 20/404)
- Executive Function Impairment: NM
- Neurobehavioral Dysregulation: 0
  - "His first known admission to a hospital was at the age of 48, for a cholecystectomy. **Neurologic and personality changes were not recorded.** His wife stated that **during the following year he became forgetful, confused, irritable, and moody."** (pg. 20/404)
- Progressive Course: Progressive
  - "The patient's **condition deteriorated progressively**." (pg. 20/404)

Supportive Features
- Depression: NM
- Anxiety: 1
  - "Several hospitalizations were necessary because of **confusion, hyperactivity, loquaciousness, and 'nervous breakdown.'"** (pg. 20/404)
- Apathy: NM
- Paranoia: NM
- Suicidality: NM
- Motor Signs (Parkinsonism / ALS): 1
  - "The first clipping in his scrapbook states that he was knocked down three times in the first round; with **his left leg almost paralyzed and barely able to support his weight,** he continued to receive numerous blows and was knocked down again. The fight was stopped in the seventh round. **He quit the profession because of a 'paralyzed left side,'** but tried an unsuccessful comeback one year later." (20/404)
  - "He showed an **ataxic gait, decreased speed of motion in the left hand, increased tendon reflexes, and extensor plantar reflexes on the left**." (pg. 20/404)
- Delayed Onset of Symptoms and Problems: 1
  - "A 53-year-old white man was a boxer from the age of 18 to 24, having had 10 bouts before the age of 20… His first known admission to a hospital was at the age of 48, for a cholecystectomy. **Neurologic and personality changes were not recorded.** His wife stated that **during the following year he became forgetful, confused, irritable, and moody**… (pg. 20/404)

Level of Functional Dependence / Dementia: IV
- "The impression was "**psychotic reaction**—the result of organic brain disease—most likely in the nature of a traumatic encephalopathy (punch drunk)." (20/404)
- "The pathological picture in the fatal case was that of a diffuse atrophy of the brain, accentuated in the frontal lobes. The gross appearance was suggestive of Pick's disease." (pg. 22/406)

Level of Functional Dependence / Dementia (Including Physical Disability): IV

Courville (1962)

Case 1 (Age: 49; Sport: Boxing)
Age started: Not mentioned
Age retired: Not mentioned
Length of career: 4 years or more (professional career described as relatively short)
Age of symptom onset: Not mentioned
Delay between retirement and symptom onset (Age of symptom onset – Age retired): Not mentioned

Core Clinical Features

- Cognitive Impairment: 1
  - "The patient was admitted to a local hospital in a **confused state**, but he was able to say that he had tripped over a rug at the rest home where he was staying (he could not remember the name of the rest home)." (pg. 161)
  - "Several features in this case are of interest, although it must be recognized that it is an example of only moderately well developed case of so-called 'punch drunk,' or (more accurately) '**psychopathic deterioration** of pugilists.' The patient's professional career as a boxer was relatively short, which is in keeping with his only **moderately advanced intellectual deterioration**. This state may also have been modified by the presence of diabetes mellitus." (pg. 164)
- Executive Function Impairment: NM
- Neurobehavioral Dysregulation: 1
  - "Ultimate placement in a rest home because of **erratic behavior**. **Uncertain habits regarding consumption of alcohol**..." (pg. 161)
- Progressive Course: Progressive
  - Note: Although Courville refers to this patient as having deteriorated, there seems to be insufficient clinical description provided in the case report to definitively classify it as progressive—although we did so for this review.

Supportive Features

- Depression: NM
- Anxiety: NM
- Apathy: NM
- Paranoia: NM
- Suicidality: NM
- Motor Signs (Parkinsonism / ALS): 1
  - "The case presented here may also be thus classified, for it was characterized clinically by a parkinsonism state (with severe histological changes in the lenticular nucleus) as well as by a severe cortical cell loss." (pg. 167)
- Delayed Onset of Symptoms and Problems: NM

Level of Functional Dependence / Dementia: IV

- "Development of 'chronic brain syndrome' characteristic of 'punch drunk' after a career of 4 years or more as a professional boxer. Ultimate placement in a rest home because of **erratic behavior**. **Uncertain habits regarding consumption of alcohol**. Death at age 49 from an episode of hypoglycemia, incident to treatment of diabetes mellitus. Autopsy: Cirrhosis of the liver, atrophy of pancreas with calculosis of pancreatic duct and atrophy of brain." (pg. 161)
- "Ultimate placement in a rest home because of **erratic behavior**..." (pg. 161)
- "Several features in this case are of interest, although it must be recognized that it is an example of only moderately well developed case of so-called 'punch drunk,' or (more accurately) '**psychopathic deterioration** of pugilists.' The patient's professional career as a boxer was relatively short, which is in keeping with his only **moderately advanced intellectual deterioration**. This state may also have been modified by the presence of diabetes mellitus." (pg. 164)

Level of Functional Dependence / Dementia (Including Physical Disability): IV

Spillane (1962)

Case 1 (Age: 45; Sport: Boxing)
Age started: 13 years (professional at age 17)
Age retired: 35 years
Length of career: 18 years
Age of symptom onset: Likely 32 years (13 years ago at the time of visit, aged 45 years)
Delay between retirement and symptom onset (Age of symptom onset – Age retired): -3 years*
*Symptom onset during career

Core Clinical Features

- Cognitive Impairment: 0
    - "**He possessed a pleasant, frank personality, and there was nothing to suggest any deterioration.** He was rather slow, but there was **no evidence of intellectual impairment**." (pg. 1205)
    - "**He is euphoric, and psychometric testing indicated that he possesses average intelligence (I.Q. 106) with little evidence of intellectual deterioration**." (pg. 1205)
- Executive Function Impairment: NM
- Neurobehavioral Dysregulation: 0
    - "**He possessed a pleasant, frank personality, and there was nothing to suggest any deterioration.**" (pg. 1205)
    - "**He is euphoric**..." (pg. 1205)
- Progressive Course: Progressive
    - "The disorder has slowly and **progressively** increased, without remission or exacerbations, for thirteen years." (pg. 1208)

Supportive Features

- Depression: 0
    - "**He is euphoric**…" (pg. 1205)
- Anxiety: NM
- Apathy: NM
- Paranoia: NM
- Suicidality: NM
- Motor Signs (Parkinsonism / ALS): 1
    - "A former professional boxer since the age of 17, he had retired from the ring at 35 'because my friends said I was **walking unsteady and throwing a leg out**, and they said that was a sign of punch-drunk.'" (pg. 1205)
    - "His **"peculiar" gait had developed in an insidious manner about 13 years ago,** and for several years **he was unaware of it** despite the comments of his wife and friends. **He was said to throw out the left leg at times, and some thought he was drunk**. He only **gradually became aware of this** unsteadiness about eight years ago. **The symptom fluctuated and was aggravated by alcohol**, but he was able to do his work and had never fallen. **If he hurries he catches his left foot in obstacles and frequently stumbles**. **Slight clumsiness of the fingers,** as when tying shoelaces or fastening buttons, **has developed in recent years**. The second symptom, again one which was noticed by others for some years before he became aware of it, was of **slurring of speech**. He has found that he has to speak slowly and carefully if he wishes to be understood. The difficulty seems to consist solely in **defective articulation; there have been no aphasic symptoms. Both the unsteadiness and slurring of speech have gradually increased over the years and seem to fluctuate**. In the past two years two further symptoms made their appearance. **There has been urgency and frequency of micturition with occasional incontinence and bouts of blurring of vision**. The latter are infrequent and sometimes consist of transient altitudinal hemianopia" (pg. 1205).
    - "He had seen several ophthalmologists, who had found no abnormality. There were **no complaints of headache, vertigo, diplopia, numbness, or paraesthesiae**, and his general health was satisfactory. He complained of dyspepsia and anorexia in 1955 and was investigated in a hospital out-patient department, where it was noted that 'there is a **marked slurring of speech and unsteadiness of gait.**'" (pg. 1205)
    - "The **positive neurological signs consisted of slurring dysarthria, a spastic-ataxic type of gait, and an extensor plantar reflex (left).** There was no ocular or optic abnormality; no nystagmus; optic disks, fundi, and visual fields were normal. Power in his limbs was good, although there was **some distal weakness in the left lower limb. Finger and hand movements revealed slight clumsiness**. All reflexes were brisk. There was no sensory loss." (pg. 1205)
    - "In the past two years he has been kept under observation… **dysarthria and an unsteady gait remain the two chief disabilities.**" (pg. 1205)
- Delayed Onset of Symptoms and Problems: 0
    - "A former professional boxer since the age of 17, he had retired from the ring at 35 'because my friends said I was **walking unsteady and throwing a leg out**, and they said that was a sign of punch-drunk.'" (pg. 1205)

  - “His **"peculiar" gait had developed in an insidious manner about 13 years ago,** and for several years **he was unaware of it** despite the comments of his wife and friends. **He was said to throw out the left leg at times, and some thought he was drunk**. He only **gradually became aware of this** unsteadiness about eight years ago.” (pg. 1205)

Level of Functional Dependence / Dementia: I

- “Although he is slow and euphoric there was no significant evidence of intellectual deterioration and he has continued to work regularly and is not very disabled.” (pg. 1208)

Level of Functional Dependence / Dementia (Including Physical Disability): II

Case 2 (Age: 52; Sport: Boxing)
Age started: Not mentioned; Age retired: 35 years; Length of career: 20 years
Age of symptom onset: Likely 40 years (12 years previously at time of visit, aged 52)
Delay between retirement and symptom onset (Age of symptom onset – Age retired): 5 years

Core Clinical Features

- Cognitive Impairment: 1
  - "He was **not aware of any difficulty with speech**, and had **no complaints to make suggesting any alteration of personality or intellectual impairment**." (pg. 1206)
  - "**His memory was unreliable**. Psychometric testing revealed **moderate intellectual impairment**." (pg. 1206)
  - "He has been kept under observation during the past two years and air encephalography was recently repeated. He is still working, although under some difficulty, but **he is slower mentally**... Psychometry showed that **his level of intelligence was dull normal (I.Q. 81) and that there was significant intellectual deterioration**." (pg. 1206)
- Executive Function Impairment: NM
- Neurobehavioral Dysregulation: NM
- Progressive Course: Progressive

Supportive Features

- Depression: 1
  - "He has been kept under observation during the past two years and air encephalography was recently repeated... **At times he is anxious or depressed**. but on the whole his personality has not significantly altered." (pg. 1206)
- Anxiety: 1
  - "He has been kept under observation during the past two years and air encephalography was recently repeated... **At times he is anxious or depressed**. but on the whole his personality has not significantly altered." (pg. 1206)
- Apathy: NM
- Paranoia: NM
- Suicidality: NM
- Motor Signs (Parkinsonism / ALS): 1
  - "His doctor had referred him because of **increasing weakness and lack of control of his right leg**. The patient stated that **this trouble had begun 12 years previously**. He recalled a sudden weakness of his right leg while carrying a sack of coal. At first the symptom was intermittent, but **over the years it has increased and he has found that he catches the toes of the right foot in the ground as he walks**. He stumbles, rarely falls, and there is no story of fluctuation of the complaint. During the past 12 years **there have been periods when he has experienced paraesthesiae and numbness in both his feet**. **His right arm is weak and clumsy at times. He finds difficulty in writing and holding a teacup**. **Ten years ago he began to have difficulty in urination**. There has been frequency, hesitancy, nocturia, occasional dribbling incontinence, and, on one occasion eight years ago, a bout of retention requiring catheterization. There has been no prostatic enlargement. **During the past three years his gait has been increasingly slow and unsteady, and he has often been thought to be drunk.** He is abstemious but has found that **alcohol aggravates his ataxia**. He works regularly and still feels strong. **He has no headache, vertigo, or diplopia**. He was **not aware of any difficulty with speech**, and had **no complaints to make suggesting any alteration of personality or intellectual impairment**." (pg. 1206)
  - "The **positive neurological signs consisted of slow slurred speech, unsteady gait, moderate right-sided hemiparesis, and partial right optic atrophy**. There was **no dysphasia, he dragged his right foot when walking, and all movements were performed slowly and carefully**. There was **moderate ataxia on formal testing in all limbs, more marked on the right**. **There was no diplopia, ocular movements were normal**, but visual acuity on the right was 6/24 and the optic disk was very pale. Both visual fields showed moderate peripheral contraction. Visual acuity on the left was 6/12. Pupillary reflexes were sluggish on the left and practically absent on the right. **The right pupil was larger than the left.** The **remaining cranial nerves were normal.** There was **diffuse moderate weakness of the right arm and leg; the arm reflexes were normal and symmetrical. The right knee- and ankle-jerks were brisker than the left. Both plantar reflexes were flexor**. Sensory testing was not very reliable, but there was probably some impairment of superficial sensation in the distal parts of the right limbs; **vibration sensation was also impaired on the right side, but joint position sense was apparently completely absent in the right hand and foot**." (pg. 1206)
- Delayed Onset of Symptoms and Problems: NM

Level of Functional Dependence / Dementia: IV

- "He has been kept under observation during the past two years and air encephalography was recently repeated. He is still working, although under some difficulty, but **he is slower mentally**. **At times he is anxious or depressed**. but on the whole his personality has not significantly altered. Psychometry showed that **his level of intelligence was dull normal (I.Q. 81) and that there was significant intellectual deterioration**." (pg. 1206)

Level of Functional Dependence / Dementia (Including Physical Disability): IV

Case 3 (Age: 69; Sport: Boxing)
Age started: 13 years; Age retired: 32 years; Length of career: 19 years
Age of symptom onset: Likely 59 years (it had been evident for about 10 years, at the time of visit, that his mental faculties were failing). Delay between retirement and symptom onset (Age of symptom onset – Age retired): Likely 10 years

Core Clinical Features

- Cognitive Impairment: 1
    - "This man, aged 69, retired from the ring at 32… For about ten years it had been evident to his friends that his **mental faculties were failing**. His **memory was seriously impaired**, there were **episodes of gross amnesia and confusion and disorientation**. He was **childish at times, had little insight, and was content and euphoric**. He was **nearly always smiling and optimistic**. Short journeys often resulted in disorientation. On one occasion, while driving a car, he was involved in an accident and sustained a minor head injury, but was confused and disorientated for several days; he had complete amnesia for the accident itself." (pg. 1206)
    - "He was found to be **confused, disorientated, and unsteady**, and he complained of **headache**." (1206)
    - "On admission **he did not know what hospital he was in, did not know the day of the week, or the year, and was generally childish and petulant in his behaviour**. **He would sulk and complain if left unattended, and smile and giggle when anyone approached his bedside**. His memories of his boxing career were reasonably retained, but places and boxers he could rarely name. He was talkative and happy." (pgs. 1206-1207)
    - "He now has a **progressive dementia**" (pg. 1208)
- Executive Function Impairment: 1
    - "This man, aged 69, retired from the ring at 32… For about ten years it had been evident to his friends that his **mental faculties were failing**. His **memory was seriously impaired**, there were **episodes of gross amnesia and confusion and disorientation**. He was **childish at times, had little insight, and was content and euphoric**. He was **nearly always smiling and optimistic**. Short journeys often resulted in disorientation. On one occasion, while driving a car, he was involved in an accident and sustained a minor head injury, but was confused and disorientated for several days; he had complete amnesia for the accident itself." (pg. 1206)
    - "He was found to be **confused, disorientated, and unsteady**, and he complained of **headache**." (pg. 1206)
    - "On admission **he did not know what hospital he was in, did not know the day of the week, or the year, and was generally childish and petulant in his behaviour**. **He would sulk and complain if left unattended, and smile and giggle when anyone approached his bedside**. His memories of his boxing career were reasonably retained, but places and boxers he could rarely name. He was talkative and happy." (pgs. 1206-1207)
    - "He now has a **progressive dementia**" (pg. 1208)
- Neurobehavioral Dysregulation: NM
- Progressive Course: Progressive

Supportive Features

- Depression: 0
    - "He was **childish at times, had little insight, and was content and euphoric**. He was **nearly always smiling and optimistic**." (pg. 1206)
    - "On admission **he did not know what hospital he was in, did not know the day of the week, or the year, and was generally childish and petulant in his behaviour**. **He would sulk and complain if left unattended, and smile and giggle when anyone approached his bedside**. His memories of his boxing career were reasonably retained, but places and boxers he could rarely name. He was talkative and happy." (pgs. 1206-1207)
- Anxiety: NM
- Apathy: NM
- Paranoia: NM
- Suicidality: NM
- Motor Signs (Parkinsonism / ALS): 1
    - "His speech was clear, but he was **unsteady on his feet and stumbled frequently**. His pupils were small but active; optic disks were normal. There was **slight generalized tremor of his upper limbs at times**, but power in all limbs was good. He was **ataxic on formal testing**; fell on the Romberg test; **could not stand on one leg or walk heel-toe fashion**. There was **no significant reflex or sensory abnormality. Plantar responses were flexor.** His peripheral and retinal arteries were not sclerotic." (pg. 1206-1207)
- Delayed Onset of Symptoms and Problems: 1
    - Delay between retirement and symptom onset (Age of symptom onset – Age retired): Likely 10 years

Level of Functional Dependence / Dementia: IV

- "He now has a **progressive dementia**" (pg. 1208)

Level of Functional Dependence / Dementia (Including Physical Disability): IV

Case 4 (Age: 33; Sport: Boxing)
Age started: 13 years (professional at age 15)
Age retired: 28 years
Length of career: 15 years
Age of symptom onset: Not mentioned
Delay between retirement and symptom onset (Age of symptom onset – Age retired): Not mentioned

Core Clinical Features

- Cognitive Impairment: 1
  - "He was a powerfully built man, in excellent physical condition, **without any outward abnormality**. **He admitted that his memory was failing…**" (pg. 1207)
- Executive Function Impairment: NM
- Neurobehavioral Dysregulation: 1
  - "**He was a wild type** but not unpleasant. He served in the Forces, enjoyed the life, had a considerable following, but resented discipline and deserted. He said he acquired and lost a fortune." (pg. 1207)
  - "He had many friends and possessed considerable charm, but his fighting instincts were a nuisance." (pg. 1207)
  - "The fourth patient, aged 33, is in excellent physical health, there is no abnormality on neurological examination, but he is **aggressive and violent**, especially when he has been drinking." (pg. 1208)
- Progressive Course: Unable to Classify

Supportive Features

- Depression: NM
- Anxiety: NM
- Apathy: NM
- Paranoia: NM
- Suicidality: NM
- Motor Signs (Parkinsonism / ALS): 0
  - **"He felt his sense of balance was uncertain and that he was now intolerant of alcohol**. His speech was quite normal, and I could find **no abnormality on physical examination**." (pg. 1207)
- Delayed Onset of Symptoms and Problems: NM

Level of Functional Dependence / Dementia: II
Level of Functional Dependence / Dementia (Including Physical Disability): II

Case 5 (Age: 45; Sport: Boxing)
Age started: 10 years (professional at age 18)
Age retired: 32 years
Length of career: 22 years
Age of symptom onset: Likely 32 years (His wife noted ill-health beginning at about this time)
Delay between retirement and symptom onset (Age of symptom onset – Age retired): Likely 0 years*
*No delay

Core Clinical Features

- Cognitive Impairment: 0
  - **"He was slow, his power of concentration and his memory for recent events were poor, but he was not demented**. He was correctly orientated and his **behaviour was quite normal**. A speech therapist formed the view that **in addition to his gross dysarthria there was slight executive dysphasia**. **Psychometry revealed a dull normal level of intelligence (I.Q. 85), with no indication of intellectual deterioration**." (pg. 1207)
- Executive Function Impairment: NM
- Neurobehavioral Dysregulation: NM
- Progressive Course: Progressive

Supportive Features

- Depression: 1
  - "He had been **drinking heavily for a few years**, and **there were bouts of depression**. However, despite the alcoholism there were long periods of abstention, but his family and friends were puzzled because **his speech remained slurred**. Two pints of beer would often render it incomprehensible. His wife also noticed that **nocturnal urinary incontinence**, which she had formerly attributed to inebriation, persisted during his periods of sobriety. **In the past ten years he has been a patient in a mental hospital on several occasions**." (pg. 1207)
  - "He was **morose and depressed**." (pg. 1207)
- Anxiety: NM
- Apathy: NM
- Paranoia: NM
- Suicidality: NM
- Motor Signs (Parkinsonism / ALS): 1
  - "His wife thought that his ill-health began about that time. The **first symptom noted was progressive slurring of speech.** He had been **drinking heavily for a few years**, and **there were bouts of depression**. However, despite the alcoholism there were long periods of abstention, but his family and friends were puzzled because **his speech remained slurred**. Two pints of beer would often render it incomprehensible. His wife also noticed that **nocturnal urinary incontinence**, which she had formerly attributed to inebriation, persisted during his periods of sobriety. **In the past ten years he has been a patient in a mental hospital on several occasions**. During one such admission, about three years ago, it was observed that he was **dragging his left leg when he walked** and **tended to hold his left arm flexed across his body**." (pg. 1207)
  - "His **speech was thick and slurred and his gait unsteady**. His pupils were normal. There was **nystagmus on right and left lateral gaze**. Power in all limbs was good, reflexes were brisk and symmetrical, and the plantar responses were flexor. There was **some diminution of sensation** (tactile, pin-prick, and vibration) **in the left leg**. B.P. 130/80. **He was slow, his power of concentration and his memory for recent events were poor, but he was not demented**. He was correctly orientated and his **behaviour was quite normal**. A speech therapist formed the view that **in addition to his gross dysarthria there was slight executive dysphasia**." (pg. 1207)
  - "The fifth patient, an alcoholic, had **permanent dysarthria and ataxia.** At necropsy the only intracranial lesions consisted of small foci of softening in the cortex of the parietal, temporal, and left cerebellar lobes and the left internal capsule." (pg. 1209)
- Delayed Onset of Symptoms and Problems: 0

Level of Functional Dependence / Dementia: II

- "A laborer aged 45 entered the Department of Neurology in March 1962. He started to box when he was 10, and turned professional at 18… he retired at the age of 32." (pg. 1207)
- "On admission to the hospital he was cooperative, and his general physical condition was good. He had been working regularly at a steel mill since August, 1961, and his wife informed me that he had taken no alcohol since then." (pg. 1207)
- "He died suddenly at work of acute myocardial infarction two months after leaving hospital." (pg. 1207)

Level of Functional Dependence / Dementia (Including Physical Disability): II

Mawdsley & Ferguson (1963)

Case 1 (Age: 54; Sport: Boxing)
Age started: 14 years (professional at 14)
Age retired: 32 years
Length of career: 18 years
Age of symptom onset: 30 years
Delay between retirement and symptom onset (Age of symptom onset – Age retired): -2 years*
*Symptom onset during career

Core Clinical Features

- Cognitive Impairment: 1
  - "His family noted a **progressive deterioration in his memory in the past 20 years**. His wife commented, too, on a **falling off in his social habits**, and on the slovenliness of his dress." (pg. 795)
  - "There was clinical and psychometric evidence of **dementia with a distinct defect of retentive memory**." (pg. 795)
- Executive Function Impairment: 1
  - "His family noted a **progressive deterioration in his memory in the past 20 years**. His wife commented, too, on a **falling off in his social habits**, and on the slovenliness of his dress." (pg. 795)
- Neurobehavioral Dysregulation: 1
  - "In his youth he had been aggressive, and throughout his boxing career was a heavy drinker. He is still **liable to outbursts of rage and violence but drinks little alcohol**, because small amounts aggravate his ataxia. When he was 34 he **was discharged from the Army on psychiatric grounds** and he has not worked since. Between the ages of 37 and 44 he **was admitted to mental hospitals on three occasions after episodes of violence.** His family noted a **progressive deterioration in his memory in the past 20 years**. His wife commented, too, on a **falling off in his social habits**, and on the slovenliness of his dress." (pg. 795)
- Progressive Course: Progressive

Supportive Features

- Depression: NM
- Anxiety: NM
- Apathy: NM
- Paranoia: NM
- Suicidality: NM
- Motor Signs (Parkinsonism / ALS): 1
  - "**From the age of 30** he noticed **slurring of his speech**, **difficulty in sleeping** at night, and **increasing lethargy and drowsiness during the day**. **Shortly after he had given up boxing he became unsteady in his gait** and **clumsy with his hands**, fastening laces and buttons and writing became difficult. His **speech and gait slowly deteriorated**." (pg. 795)
  - "His **speech was slurred; his gait was ataxic, particularly when he turned.** His **pupils were small; there was restriction of upward gaze and fine nystagmus on lateral deviation of the eyes**. Muscular power was excellent, but he showed **slight intention tremor in the finger-nose test and was ataxic in the heel-knee test. Fine finger movements were impaired**. The **tendon reflexes were brisker on the left side** and the **left plantar response was equivocally extensor**." (pgs. 795-796)
- Delayed Onset of Symptoms and Problems: 0
  - "**From the age of 30** he noticed **slurring of his speech**, **difficulty in sleeping** at night, and **increasing lethargy and drowsiness during the day**. **Shortly after he had given up boxing he became unsteady in his gait** and **clumsy with his hands**, fastening laces and buttons and writing became difficult." (pg. 795)

Level of Functional Dependence / Dementia: IV
Level of Functional Dependence / Dementia (Including Physical Disability): IV

Case 2 (Age: 58; Sport: Boxing)
Age started: 16 years (professional at 16)
Age retired: 36 years
Length of career: 20 years
Age of symptom onset: Likely 36 years (Retired because of unsteadiness in legs, see below)
Delay between retirement and symptom onset (Age of symptom onset – Age retired): Likely 0 years*
*No delay

Core Clinical Features

- Cognitive Impairment: 1
  - "A pleasant, garrulous man who **clinically showed a gross defect of retentive memory which was confirmed on psychometric testing**. (pg. 796)
- Executive Function Impairment: NM
- Neurobehavioral Dysregulation: NM
- Progressive Course: Progressive

Supportive Features

- Depression: NM
- Anxiety: NM
- Apathy: NM
- Paranoia: NM
- Suicidality: NM
- Motor Signs (Parkinsonism / ALS): 1
  - "He retired from the ring because his **legs were becoming unsteady**…The **unsteadiness of his gait slowly worsened**. When he was about 38 his family and his friends commented on the **slurring of his speech**, and **2 years later he noticed tremor of his hands**… He has **had frequent falls during the past 5 years and now cannot walk unaided.**" (pg. 796)
  - "His **speech was dysarthric and monotonous**, his **gait distinctly ataxic**. The **pupils were small**; there was **restriction of upward gaze**, and **fine nystagmus on full lateral deviation of the eyes**. His **facial expression was fixed**, and he showed a **constant, coarse tremor of the arms and hands which was more obvious on the right**. **Tone was increased in his arms and legs**. **Finger movements were clumsy**. **Plantar responses were flexor**." (pg. 796)
- Delayed Onset of Symptoms and Problems: 0
  - "He retired from the ring because his **legs were becoming unsteady**. People accused him of being drunk though he is a lifelong teetotaller. The **unsteadiness of his gait slowly worsened**. When he was about 38 his family and his friends commented on the **slurring of his speech**, and **2 years later he noticed tremor of his hands**." (pg. 796)

Level of Functional Dependence / Dementia: IV

- "His **disability gradually increased** though he managed to work as a road sweeper until 10 years ago. Since then he has been unemployed. He has **had frequent falls during the past 5 years and now cannot walk unaided.**" (pg. 796)

Level of Functional Dependence / Dementia (Including Physical Disability): IV

Case 3 (Age: 69; Sport: Boxing)
Age started: 16 years
Age retired: 24 years (after a blow which impaired vision in his left eye)
Length of career: 8 years
Age of symptom onset: 50 years
Delay between retirement and symptom onset (Age of symptom onset – Age retired): 26 years

Core Clinical Features

- Cognitive Impairment: 1
  - "At the age of 50 he and his family noticed a **deterioration in his memory**. **This defect was severe for recent events and progressed.** On occasion he forgot where to get off the bus on his way to work and would forget in mid-sentence what he wanted to say." (pg. 796)
  - "Clinical testing showed **grossly defective memory, which was confirmed by psychometry**." (pg. 796)
- Executive Function Impairment: NM
- Neurobehavioral Dysregulation: 1
  - **"He became increasingly depressed and irritable**, and was apt to fall asleep during the day and to have **occipital, throbbing headaches almost daily."** (pg. 796)
- Progressive Course: Progressive
  - "At the age of 50 he and his family noticed a **deterioration in his memory**. **This defect was severe for recent events and progressed.**" (pg. 796)

Supportive Features

- Depression: 1
  - **"He became increasingly depressed and irritable**, and was apt to fall asleep during the day and to have **occipital, throbbing headaches almost daily."** (pg. 796)
  - "The patient was a reserved, **depressed man** with unscarred face and hands." (pg. 796)
- Anxiety: NM
- Apathy: NM
- Paranoia: NM
- Suicidality: NM
- Motor Signs (Parkinsonism / ALS): 0
  - **"Speech and gait were normal**. The left optic disc was abnormally pale. Visual acuity in the left eye was 6/60. There was **sustained nystagmus on lateral deviation of the eyes**. **Both plantar responses were equivocally extensor**." (pg. 796)
- Delayed Onset of Symptoms and Problems: 1 (26 years)

Level of Functional Dependence / Dementia: IV
Level of Functional Dependence / Dementia (Including Physical Disability): IV

Case 4 (Age: 57; Sport: Boxing)
Age started: 15 years
Age retired: 24 years
Length of career: 9 years
Age of symptom onset: 24 years (convulsions), 52 years (paranoid hallucinations)
Delay between retirement and symptom onset (Age of symptom onset – Age retired): No delay for convulsions, 28 years (other symptoms)

Core Clinical Features
- Cognitive Impairment: 1
  - "In the past 3 years his wife noticed **deterioration in his memory**." (pg. 796)
  - "A **gross defect of retentive memory was apparent clinically, and psychometric testing showed evidence of intellectual deterioration**." (pg. 796)
- Executive Function Impairment: NM
- Neurobehavioral Dysregulation: 1
  - "He **became violent at such times**, and **twice was admitted to a mental hospital**. These **psychotic states lasted 3 or 4 days, and recovery was spontaneous and complete on each occasion**." (pg. 796)
- Progressive Course: Unable to Classify

Supportive Features
- Depression: NM
- Anxiety: NM
- Apathy: NM
- Paranoia: 1
  - "On four occasions during the past five years, he experienced **paranoid hallucinatory states which were accompanied by clouding of consciousness and usually followed a series of grand-mal fits**. He **became violent at such times**, and **twice was admitted to a mental hospital**. These **psychotic states lasted 3 or 4 days, and recovery was spontaneous and complete on each occasion**." (pg. 796)
- Suicidality: NM
- Motor Signs (Parkinsonism / ALS): 1
  - "The only abnormal neurological signs were **fine nystagmus on lateral deviation of the eyes and severe impairment of fine hand movements**." (pg. 796-797)
- Delayed Onset of Symptoms and Problems: 1 (28 years)
  - "He stopped boxing at the age of 24 because he had a **grand-mal convulsion**. For some years thereafter he had fits at intervals of a few days; their frequency gradually decreased, but he still has at least one major attack each month." (pg. 796)

Level of Functional Dependence / Dementia: IV
- "He lost many jobs after having fits at work and has been unemployed for the past 2 years." (pg. 796)

Level of Functional Dependence / Dementia (Including Physical Disability): IV

Case 5 (Age: 55; Sport: Boxing)
Age started: 15 years (professional at 15)
Age retired: 30 years (retired professionally at 21)
Length of career: 15 years
Age of symptom onset: 30 years
Delay between retirement and symptom onset (Age of symptom onset – Age retired): 0 years*
*No delay

Core Clinical Features

- Cognitive Impairment: 1
  - "He then began to show **defects of memory and developed paranoid delusions**." (pg. 797)
  - "There was **clinical and psychometric evidence of dementia and gross memory defect**." (pg. 797)
- Executive Function Impairment: NM
- Neurobehavioral Dysregulation: 1
  - "Though he was always a querulous person, quick to take offence, **his domestic life was fairly placid until about 15 years ago.** He then began to show **defects of memory and developed paranoid delusions**. He was **twice admitted to psychiatric units after outbursts of violence**." (pg. 797)
  - "He was **morose and irritable**." (pg. 797)
- Progressive Course: Progressive
  - "**These symptoms have progressed**." (pg. 797)

Supportive Features

- Depression: 1
  - "He was **morose and irritable**." (pg. 797)
- Anxiety: NM
- Apathy: NM
- Paranoia: 1
  - "He then began to show **defects of memory and developed paranoid delusions**. He was **twice admitted to psychiatric units after outbursts of violence**." (pg. 797)
  - "His conversation revolved around his wife's supposed infidelity, his family's enmity, and the ill-treatment he had received." (pg. 797)
- Suicidality: NM
- Motor Signs (Parkinsonism / ALS): 1
  - "When he was 30 **tremor of his right hand became manifest**, particularly when he lifted a cup to his lips. The **tremor slowly increased in amplitude**, and his writing steadily deteriorated into illegibility. When he was about 35 **his gait became unsteady and his speech became slurred**." (pg. 797)
  - "He was **grossly dysarthric**; **his gait was spastic and ataxic**; but his muscular development and power were exceptionally good. There **was cog-wheel rigidity of all four limbs, more obvious on the right side**. He had a constant **'pill-rolling' tremor of both hands at rest and gross intention tremor on purposive movements of the right arm**. There was **ataxia of the left arm and in the heel-shin tests**. **Both plantar responses were extensor**." (pg. 797)
- Delayed Onset of Symptoms and Problems: 0
  - "When he was 30 **tremor of his right hand became manifest**…" (pg. 797)

Level of Functional Dependence / Dementia: IV
Level of Functional Dependence / Dementia (Including Physical Disability): IV

Case 6 (Age: 55; Sport: Boxing)
Age started: 12 years (professional at 16)
Age retired: 30 years
Length of career: 18 years
Age of symptom onset: 33 years
Delay between retirement and symptom onset (Age of symptom onset – Age retired): 3 years

Core Clinical Features

- Cognitive Impairment: 1
  - "The patient showed **clinical and psychometric evidence of intellectual impairment and memory defect**." (pg. 797)
- Executive Function Impairment: NM
- Neurobehavioral Dysregulation: NM
- Progressive Course: Progressive

Supportive Features

- Depression: NM
- Anxiety: NM
- Apathy: NM
- Paranoia: NM
- Suicidality: NM
- Motor Signs (Parkinsonism / ALS): 1
  - "At the age of 33 his **left arm and hand became weak and unsteady**. **5 years ago his wife noticed slurring of his speech and unsteadiness of his gait**. His **writing slowly deteriorated**. During the past 4 years, **on seven occasions he lost consciousness for 30 to 45 minutes**." (pg. 797)
  - "His **facial expression was " fixed " and parkinsonian**, and his **speech was dysarthric**. He **had an ataxic gait**. There was **sustained nystagmus on lateral deviation of the eyes and restriction of upward gaze**. **Tone was increased in the left arm and leg**. **Fine movements of the left hand were clumsy**, and there was **gross intention tremor on purposive movements of the left arm**. There were similar, but slighter, signs on the right**.** There was **ataxia in the heel-knee test**. **Tendon reflexes were brisker on the left side**. **Both plantar responses were flexor**." (pg. 797)
- Delayed Onset of Symptoms and Problems: 1 (3 years)
  - "At the age of 33 his **left arm and hand became weak and unsteady**…" (pg. 797)

Level of Functional Dependence / Dementia: III

- "He has been unemployed for a year." (pg. 797)

Level of Functional Dependence / Dementia (Including Physical Disability): III

Case 7 (Age: 63; Sport: Boxing)
Age started: 16 years
Age retired: 26 years
Length of career: 10 years
Age of symptom onset: 38 years
Delay between retirement and symptom onset (Age of symptom onset – Age retired): 12 years

Core Clinical Features

- Cognitive Impairment: 1
  - "**In the past 10 years he and his wife had noted a deterioration in his memory."** (pg. 797)
- Executive Function Impairment: NM
- Neurobehavioral Dysregulation: NM
- Progressive Course: Progressive

Supportive Features

- Depression: NM
- Anxiety: NM
- Apathy: NM
- Paranoia: NM
- Suicidality: NM
- Motor Signs (Parkinsonism / ALS): 1
  - "When he was about 38 a nursing sister drew his attention to his **staggering gait**. Thereafter many people called him punch-drunk though he was conscious of little disability until his early fifties. He then noticed **unsteadiness of his gait and heaviness of his legs**, and found he was unable to run or to walk quickly. He became aware that his **speech was slurred**, though **his wife had commented on this years before**." (pg. 797)
  - "His **unsteadiness of gait and his difficulty with speech slowly worsened**. He is **now unable to walk unaided**." (pg. 797)
  - "The patient's **speech was dysarthric**, his **gait was grossly ataxi**c, and he was **unable to walk without support**. **Upward gaze was restricted**. He had **nystagmus on full lateral deviation of the eyes, slower and coarser in amplitude on looking to the right**. There was **intention tremor of the right hand in the finger-nose test and gross ataxia in each leg when performing the heel-knee tests**. An **increase in tone of " clasp-knife " type was found in both legs**. The **tendon reflexes were exaggerated, particularly on the right side. Both plantar responses were extensor**." (pg. 797)
- Delayed Onset of Symptoms and Problems: 1 (12 years)
  - "When he was about 38 a nursing sister drew his attention to his **staggering gait**…" (pg. 797)

Level of Functional Dependence / Dementia: III
Level of Functional Dependence / Dementia (Including Physical Disability): III

Case 8 (Age: 63; Sport: Boxing)
Age started: 14 years (professional at 14)
Age retired: 35 years
Length of career: 21 years
Age of symptom onset: Not mentioned
Delay between retirement and symptom onset (Age of symptom onset – Age retired): Not mentioned

Core Clinical Features

- Cognitive Impairment: 1
  - "He denied any other disability and it was impossible to obtain a detailed history from him**.**" (pg. 798)
  - "There was **gross impairment of intellect and memory**." (pg. 798)
- Executive Function Impairment: NM
- Neurobehavioral Dysregulation: 1
  - "**He had always been a heavy beer drinker and was involved in many brawls and had convictions for assault. Because of his violent behaviour he was admitted to a mental hospital when he was 40**. His wife left him 3 years ago because of his **drunkenness and violence**… He frequently escaped to get drunk and often assaulted his fellow-inmates." (pg. 798)
- Progressive Course: Unable to Classify

Supportive Features

- Depression: NM
- Anxiety: NM
- Apathy: NM
- Paranoia: NM
- Suicidality: NM
- Motor Signs (Parkinsonism / ALS): 1
  - "His **facial expression was fixed**. His **speech was slow and dysarthric**. He **walked with an ataxic gait on a broad base, taking short, shuffling steps**. The optic fundi showed **macular degeneration**. Visual acuity in the right eye was 6/36. The left eye was amblyopic. The **pupils were small and reacted sluggishly to light.** There was a **defect in conjugate upward deviation and in convergence of the eyes.** The **jaw jerk was brisk**. **There was rigidity of all four limbs, and a coarse tremor of the hands. Fine movements of the fingers were impaired,** and there was **slight intention tremor in both hands**. **The tendon reflexes were brisk, particularly in the arms. Hoffman's sign was positive bilaterally. Both plantar responses were equivocally extensor. The abdominal reflexes were unobtainable**." (pg. 798)
- Delayed Onset of Symptoms and Problems: NM
  - "He gave up boxing because of **deterioration in his vision**. His only complaint was of poor eyesight. He denied any other disability and it was impossible to obtain a detailed history from him…" (pg. 798)

Level of Functional Dependence / Dementia: IV

- "He has not worked for three years. Whilst unemployed he lived as a tramp, begging drinks and sleeping in public lavatories, until he was admitted to a hostel 18 months ago. During his stay there he made himself a nuisance. He frequently escaped to get drunk and often assaulted his fellow-inmates." (pg. 798)

Level of Functional Dependence / Dementia (Including Physical Disability): IV

Case 9 (Age: 33; Sport: Boxing)
Age started: 14 years
Age retired: 26 years
Length of career: 12 years
Age of symptom onset: 19 years
Delay between retirement and symptom onset (Age of symptom onset – Age retired): -7 years*
*Symptom onset during career

Core Clinical Features

- Cognitive Impairment: 1
  - "At this time, too, **his memory deteriorated and his handwriting became untidy**. In the next two years these symptoms persisted, and he attributed his failure in examinations to the **falling off in his memory**… He **remained conscious of a speech difficulty**, particularly on the telephone, and **believed that his memory was faulty**." (pg. 798)
- Executive Function Impairment: NM
- Neurobehavioral Dysregulation: NM
- Progressive Course: Progressive

Supportive Features

- Depression: NM
- Anxiety: NM
- Apathy: NM
- Paranoia: NM
- Suicidality: NM
- Motor Signs (Parkinsonism / ALS): 1
  - "When he was 19 he noticed that **his speech, previously precise and clipped, was becoming slurred**. This was apparent when he spoke quickly and when he was tired. At this time, too, **his memory deteriorated and his handwriting became untidy**. In the next two years these symptoms persisted, and he attributed his failure in examinations to the **falling off in his memory**. Fearing he was becoming punch-drunk he stopped boxing regularly, and fought only occasionally in the next 5 years. About the time he stopped boxing completely he began to have **almost constant occipital, throbbing headaches, which latterly came on at weekly intervals**. He **remained conscious of a speech difficulty**, particularly on the telephone, and **believed that his memory was faulty**. His only neurological abnormality was **slight but definite slurring dysarthria**." (pg. 798)
- Delayed Onset of Symptoms and Problems: 0

Level of Functional Dependence / Dementia: III
Level of Functional Dependence / Dementia (Including Physical Disability): III

Case 10 (Age: 51; Sport: Boxing)
Age started: 14 years
Age retired: 35 years
Length of career: 21 years
Age of symptom onset: Likely 35 years (see below)
Delay between retirement and symptom onset (Age of symptom onset – Age retired): 0 years*
*No delay

Core Clinical Features

- Cognitive Impairment: 1
  - "His wife noticed a **progressive mental impairment from the age of 35**." (pg. 798)
  - "His memory deteriorated…" (pg. 798)
  - "There was **clinical evidence of dementia**…" (pg. 798)
- Executive Function Impairment: NM
- Neurobehavioral Dysregulation: 0
  - "He was an amiable man and had **no record of aggressive or violent behaviour.**" (pg. 798)
- Progressive Course: Progressive
  - "He had a **set, expressionless face and slurring of his speech, and was noticeably clumsy in the use of his hands**. His wife noticed a **progressive mental impairment from the age of 35**." (pg. 798)

Supportive Features

- Depression: NM
- Anxiety: NM
- Apathy: 1
  - "**During the last 10 years of his life he spent most of the day sitting apathetically at home**. He became slovenly in his dress and habits, wearing the same clothes and underclothes for weeks on end unless he was badgered to change. He took no interest in his family's welfare. **The lethargy was in striking contrast to the liveliness and alertness described by people who knew him in his twenties."** (pg. 798)
- Paranoia: NM
- Suicidality: NM
- Motor Signs (Parkinsonism / ALS): 1
  - "He had a **set, expressionless face and slurring of his speech, and was noticeably clumsy in the use of his hands**. His wife noticed a **progressive mental impairment from the age of 35**." (pg. 798)
  - "Never a heavy drinker, he was easily rendered ataxic by small amounts of alcohol." (pg. 798)
  - "He had **pronounced slurring dysarthria and an expressionless parkinsonian-like face**. The **tendon reflexes were abnormally brisk**. The **abdominal reflexes could not be elicited.** The **plantar responses were flexor**." (pg. 798)
- Delayed Onset of Symptoms and Problems: 0

Level of Functional Dependence / Dementia: IV

- "**During the last 10 years of his life he spent most of the day sitting apathetically at home**. He became slovenly in his dress and habits, wearing the same clothes and underclothes for weeks on end unless he was badgered to change. He took no interest in his family's welfare. **The lethargy was in striking contrast to the liveliness and alertness described by people who knew him in his twenties**…" (pg. 798)
- "… and he lost his last job when he was 42 because he could not remember his duties and was unreliable in keeping to a schedule." (pg. 798)

Level of Functional Dependence / Dementia (Including Physical Disability): IV

Payne (1968)

Note: This article focuses on postmortem neuropathological findings. Payne states in the introduction that “this is a report of the postmortem findings in the brains of six ex-professional boxers” (pg. 173). However, there are some clinical features described for most of the cases. These are presented below.

Case 1 (Age: Not Mentioned; Sport: Boxing)
Age started: Not mentioned
Age retired: Not mentioned
Length of career: 12 years
Age of symptom onset: Not mentioned
Delay between retirement and symptom onset (Age of symptom onset – Age retired): Not mentioned

Core Clinical Features
- Cognitive Impairment: NM
- Executive Function Impairment: NM
- Neurobehavioral Dysregulation: NM
- Progressive Course: Unable to Classify

Supportive Features
- Depression: 1
    - “In 1914 he was admitted to hospital with ‘**Manic Depressive Psychosis**’ and thereafter he spent many years in mental institutions. He had a **guilt complex** about his mis-spent youth.” (pg. 175)
- Anxiety: NM
- Apathy: NM
- Paranoia: NM
- Suicidality: NM
- Motor Signs (Parkinsonism / ALS): NM
- Delayed Onset of Symptoms and Problems: NM

Level of Functional Dependence / Dementia: III
- “In 1914 he was admitted to hospital with ‘**Manic Depressive Psychosis**’ and thereafter he spent many years in mental institutions.” (pg. 175)

Level of Functional Dependence / Dementia (Including Physical Disability): III

Case 2 (Age: 46; Sport: Boxing)
Age started: Early teens (specific age not specified; professional at 20 years)
Age retired: 33 years
Length of career: 13 years (professional only)
Age of symptom onset: 33 years
Delay between retirement and symptom onset (Age of symptom onset – Age retired): 0 years*
*No delay

Core Clinical Features

- Cognitive Impairment: NM
- Executive Function Impairment: NM
- Neurobehavioral Dysregulation: 1
  - "Episodes of strife occurred between his mother and himself and **he became violent and paranoid.** In the month before his death one of the attacks led him to an attempt to set his house on fire." (pg. 176)
  - "He was **emotionally labile** and required constant supervision." (pg. 176)
- Progressive Course: Progressive

Supportive Features

- Depression: 1
  - "His hypertension was treated but he became **depressed**, partly because of unemployment… He was **emotionally labile** and required constant supervision." (pg. 176)
- Anxiety: NM
- Apathy: NM
- Paranoia: 1
  - "Episodes of strife occurred between his mother and himself and **he became violent and paranoid.** In the month before his death one of the attacks led him to an attempt to set his house on fire." (pg. 176)
- Suicidality: NM
- Motor Signs (Parkinsonism / ALS): 1
  - "Examination at the time noted that he had **slurring of speech and unsteady gait**. His hypertension was treated but he became **depressed**, partly because of unemployment, and his **dysarthria and ataxia became more severe**… On admission to hospital he had **dysarthria, ataxia, papilloedema and a left III nerve palsy**." (pg. 176)
- Delayed Onset of Symptoms and Problems: 0

Level of Functional Dependence / Dementia: III

- "He… gave up boxing at 33 years of age; then he went to work in a factory. At about that time he **complained of headaches** and the noise of the machinery in the factory." (pg. 176)
- "His hypertension was treated but he became **depressed**, partly because of unemployment, and his **dysarthria and ataxia became more severe**. Episodes of strife occurred between his mother and himself and **he became violent and paranoid.** In the month before his death one of the attacks led him to an attempt to set his house on fire. On admission to hospital he had **dysarthria, ataxia, papilloedema and a left III nerve palsy**. He was **emotionally labile** and required constant supervision." (pg. 176)

Level of Functional Dependence / Dementia (Including Physical Disability): III

Case 3 (Age: 46; Sport: Boxing)
Age started: 19 years
Age retired: 31 years
Length of career: 12 years
Age of symptom onset: Not specified ("after giving up boxing," see below)
Delay between retirement and symptom onset (Age of symptom onset – Age retired): Not mentioned

Core Clinical Features

- Cognitive Impairment: 1
  - "After giving up boxing his main complaint was of **headaches and lack of concentration** although he also suffered with **insomnia, enuresis and periods of depression.**" (pg. 177)
  - "In the last year of his life his **memory was poor**, he was **sometimes confused and vague** and he **became dirty and untidy in his dress**." (pg. 177)
- Executive Function Impairment: 1
  - "After giving up boxing his main complaint was of **headaches and lack of concentration** although he also suffered with **insomnia, enuresis and periods of depression.**" (pg. 177)
  - "In the last year of his life his **memory was poor**, he was **sometimes confused and vague** and he **became dirty and untidy in his dress**." (pg. 177)
- Neurobehavioral Dysregulation: 1
  - "On most of his many admissions to hospital he was **pleasant and cooperative** although **on a few occasions he was abusive and threatening** to the medical staff and to other patients." (pg. 177)
- Progressive Course: Unable to Classify

Supportive Features

- Depression: 1
  - "After giving up boxing his main complaint was of **headaches and lack of concentration** although he also suffered with **insomnia, enuresis and periods of depression."** (pg. 177)
- Anxiety: NM
- Apathy: NM
- Paranoia: NM
- Suicidality: NM
- Motor Signs (Parkinsonism / ALS): 1
  - "Eight years after the end of his boxing career it was first noted that he had **slurring of speech**…" (pg. 177)
  - "…his **speech was slightly abnormal and there was mild impairment of coordination in his left arm**." (pg. 177)
- Delayed Onset of Symptoms and Problems: NM

Level of Functional Dependence / Dementia: III

- "He became a heavy drinker of spirits and was treated in several hospitals for alcoholism." (pg. 177)
- "He worked intermittently as a laborer but was always work-shy; in fifteen years his longest period of continuous work was three months. He was unable to get on with his wife and he left her." (pg. 177)
- "In the last year of his life his **memory was poor**, he was **sometimes confused and vague** and he **became dirty and untidy in his dress**." (pg. 177)
- "He was found dead in bed after an alcoholic bout…" (pg. 177)

Level of Functional Dependence / Dementia (Including Physical Disability): III

Case 4 (Age: 45; Sport: Boxing)
Age started: 19 years
Age retired: 32 years
Length of career: 14 years professional in addition to “several” years as an amateur
Age of symptom onset: Likely 40 years (see below) (“after giving up boxing,” see below)
Delay between retirement and symptom onset (Age of symptom onset – Age retired): Not mentioned

Core Clinical Features

- Cognitive Impairment: 0
    - “During this period **he complained of headaches, depression, lack of concentration…** His intelligence quotient was at the lower level of normal, there was **no evidence of intellectual deterioration**.” (pg. 178)
- Executive Function Impairment: NM
- Neurobehavioral Dysregulation: 1
    - “He had been drinking about ten pints of beer a day for many years but at the time **two pints of beer made him impulsively violent**.” (pg. 178)
- Progressive Course: Unable to Classify

Supportive Features

- Depression: 1
    - “His intemperate habits had resulted in marital disharmony. In the following five years he undertook repeated medical treatment for his alcoholism but in the intervals he was consuming large quantities of beer. During this period **he complained of headaches, depression…**” (pg. 178)
- Anxiety: NM
- Apathy: NM
- Paranoia: NM
- Suicidality: NM
- Motor Signs (Parkinsonism / ALS): 1
    - “One year later [likely age 40; one year after the ensuing seven years after retiring at age 32] he was under care as an alcoholic; **his speech was slurred and his gait shuffling**…” (pg. 178)
    - “During this period **he complained of headaches, depression, lack of concentration, insomnia and enuresis**: **his speech was persistently slurred, he had nystagmus, incoordination of his limbs and a mild sensory impairment in the left leg…**” (pg. 178)
    - “He died suddenly as the result of myocardial infarction.” (pg. 178)
- Delayed Onset of Symptoms and Problems: NM

Level of Functional Dependence / Dementia: I
Level of Functional Dependence / Dementia (Including Physical Disability): III

Case 5 (Age: 44; Sport: Boxing)
Age started: 15 years
Age retired: 28 years
Length of career: 13 years
Age of symptom onset: Not mentioned
Delay between retirement and symptom onset (Age of symptom onset – Age retired): Not mentioned

Core Clinical Features
- Cognitive Impairment: NM
- Executive Function Impairment: NM
- Neurobehavioral Dysregulation: NM
- Progressive Course: Unable to Classify

Supportive Features
- Depression: NM
- Anxiety: NM
- Apathy: NM
- Paranoia: NM
- Suicidality: NM
- Motor Signs (Parkinsonism / ALS): 2
    - "**His speech was occasionally indistinct and he was sometimes unsteady on his feet**." (pg. 181)
- Delayed Onset of Symptoms and Problems: NM

Level of Functional Dependence / Dementia: I
- "Some of his acquaintances thought that he was quite normal while **others considered that he was 'early punchy.' …** He died age 44 years from coronary atheroma and thrombosis." (pg. 181)
- "Cases 5 and 6 did not complain of any symptoms" (pg. 186)

Level of Functional Dependence / Dementia (Including Physical Disability): II

Case 6 (Age: Not Mentioned; Sport: Boxing)
Age started: 12 years
Age retired: Not mentioned
Length of career: Professional career lasted for 4 years
Age of symptom onset: Not mentioned
Delay between retirement and symptom onset (Age of symptom onset – Age retired): Not mentioned

Core Clinical Features
- Cognitive Impairment: NM
- Executive Function Impairment: NM
- Neurobehavioral Dysregulation: NM
- Progressive Course: Unable to Classify

Supportive Features
- Depression: NM
- Anxiety: NM
- Apathy: NM
- Paranoia: NM
- Suicidality: NM
- Motor Signs (Parkinsonism / ALS): NM
- Delayed Onset of Symptoms and Problems: NM

Level of Functional Dependence / Dementia: II
- "His boxing career ended prematurely because of a prison sentence. He died four years later as the result of a stab wound of the heart." (pg. 181)
- "Cases 5 and 6 did not complain of any symptoms" (pg. 186)

Level of Functional Dependence / Dementia (Including Physical Disability): II

Johnson (1969)

Note. Johnson (1969) presents 17 cases, four of which he describes in detail (Cases 1, 6, 10, and 17). However, throughout the article, Johnson provides scattered descriptions of the clinical features of different cases. Although limited, these descriptions were extracted for each case and are described below. Moreover, he presented a Figure in which certain features were marked as present or not. Johnson states that 10 cases were already reported by Mawdsley and Ferguson (1963) and are now being assessed 4-5 years later. However, he does not state which 10 cases these are. Therefore, we assume that some of these cases are not unique cases—they are follow-up cases from Mawdsley and Ferguson.

Case 1 (Age: 60; Sport: Boxing)
Age started: Not mentioned
Age retired: 32 years
Length of career: Not mentioned
Age of symptom onset: 32 years
Delay between retirement and symptom onset (Age of symptom onset – Age retired): 0 years (i.e., no delay)

Core Clinical Features

- Cognitive Impairment: 1
    - "**Mental torpor**, with a **slow, sluggish response to questions** and an **absence of spontaneity in conversation**, was present in three cases (2, 8, 15), although it was a more **prominent feature** in the cases with **dementia** (**1**, 16, 17)." (pg. 47)
    - "Three cases (**1**, 16, 17) suffered from **post-traumatic dementia** with **mental torpor** and **flat affective responses.**" (pg. 47)
    - "A 60-year-old ex-professional flyweight developed **slurred speech**, **tremor of the hands** and **defects of recent memory at the age of 32 years**, **which forced his retirement**." (pg. 47)
    - "**Over the past 20 years** there has been a **progressive, insidious deterioration of memory and intellect**. According to his wife he mislaid objects forgot the names of familiar persons and would reiterate three or four times the same account of some recent happening. **His personal habits have deteriorated** and he has become slovenly in his dress and has had to be pressed by his family to work." (pg. 47)
    - "At the age of 55 years **psychometric testing showed gross intellectual deterioration**." (pg. 47)
    - "In Cases **1** and 16, **dementia was paralleled by progressive neurological disability**." (pg. 47)
    - "In three cases (**1**, 16, 17) there was **dementia due to a progressive cerebral degenerative process**." (pg. 50)
- Executive Function Impairment: 1
    - "**Mental torpor**, with a **slow, sluggish response to questions** and an **absence of spontaneity in conversation**, was present in three cases (2, 8, 15), although it was a more **prominent feature** in the cases with **dementia** (**1**, 16, 17)." (pg. 47)
    - "Three cases (**1**, 16, 17) suffered from **post-traumatic dementia** with **mental torpor** and **flat affective responses.**" (pg. 47)
    - "At the age of 55 years **psychometric testing showed gross intellectual deterioration**." (pg. 47)
    - "In Cases **1** and 16, **dementia was paralleled by progressive neurological disability**." (pg. 47)
    - "In three cases (**1**, 16, 17) there was **dementia due to a progressive cerebral degenerative process**." (pg. 50)
- Neurobehavioral Dysregulation: 1
    - **"About 15 years ago he developed partial impotence** and would make **furious sexual demands on his wife**. **Morbid ideas of jealousy developed** about her and he would have **frequent outbursts of temper in his accusations**. His sexual interests were maintained, although he has been completely impotent for ten years." (pg. 47)
    - "He **had always been an impulsive, aggressive person** and was discharged from the Army as a 'psychopathic personality.' His **drinking pattern became 'compulsive' in type** and he was subsequently **admitted to mental hospital on four occasions for uncontrollable outbursts of rage**." (pg. 47)
    - "**Severe personality disorders** were present in four cases (**1**, 8, 9, 10). In three of these (**1**, 9, 10) **impulsive aggressive behavior was prominent as a life-long trait** and justified the description of '**explosive, psychopathic personality**.' **Rage reaction**, **uncontrolled outbursts of anger and violence**, were prominent in the case histories of these men after their boxing careers and were attributed by them to their decreased tolerance to alcohol. All three cases had had numerous emergency **psychiatric admissions for impulsive acts of violence,** of short duration." (pg. 48)
    - "Aggression must be a prominent personality characteristic of any successful pugilist. Three cases (1, 9, 10) had always been described as **sensitive, suspicious, impulsive and aggressive**, particularly when primed with alcohol, and all justified the label of "explosive psychopaths." (pg. 51)
- Progressive Course: Progressive

    - "**Progression in severity of the neurological symptoms** was evident in eight cases (**1**, 2, 8, 11, 12, 15, 16, 17) and was confirmed in the four years of the follow-up." (pg. 45)
    - "In those with progressive neurological disorder only two (**1** and 16) had **progressive psychiatric disability.**" (pg. 47)
    - "**Over the past 20 years** there has been a **progressive, insidious deterioration of memory and intellect**." (pg. 47)
    - "**Neurological and psychiatric symptoms have progressed**. He now presents severe dementia and is disabled with severe Parkinsonian symptoms, although he still lives at home with his family." (pg. 47)
    - In three cases (**1**, 16, 17) there was **dementia due to a progressive cerebral degenerative process**." (pg. 50)

Supportive Features

- Depression: NM
- Anxiety: NM
- Apathy: 1
    - "Three cases (**1**, 16, 17) suffered from **post-traumatic dementia** with **mental torpor** and **flat affective responses.**" (pg. 47)
- Paranoia: 1
    - "**About 15 years ago he developed partial impotence** and would make **furious sexual demands on his wife**. **Morbid ideas of jealousy developed** about her and he would have **frequent outbursts of temper in his accusations**. His sexual interests were maintained, although he has been completely impotent for ten years." (pg. 47)
    - "**Persistent accusations against the wife's supposed sexual infidelity** led to the primary **psychiatric referral** in five cases (**1**, 6, 8, 9, 10)." (pg. 48)
    - "The patient's **accusations and suspicions of the wife's infidelity occurred episodically** and were seen as **abnormal psychogenic reactions** in sensitive personalities. It is difficult not to presume that impotence in these cases had played some part as a pathoplastic feature in determining this particular reaction." (pgs. 51-52)
- Suicidality: NM
- Motor Signs (Parkinsonism / ALS): 1
    - "**Progression in severity of the neurological symptoms** was evident in eight cases (**1**, 2, 8, 11, 12, 15, 16, 17) and was confirmed in the four years of the follow-up… **Ataxia** combined with **festination of gait** were the most disabling features." (pgs. 45-47)
    - "**Neurological and psychiatric symptoms have progressed**. He now presents severe dementia and is disabled with severe Parkinsonian symptoms, although he still lives at home with his family." (pg. 47).
    - "In Cases **1** and 16, **dementia was paralleled by progressive neurological disability**." (pg. 47)
- Delayed Onset of Symptoms and Problems: 0

Level of Functional Dependence / Dementia: IV

- "At the age of 55 years **psychometric testing showed gross intellectual deterioration**, whilst the AEG showed ventricular dilatation, cortical atrophy and a **cavum septum pellucidum**. **Neurological and psychiatric symptoms have progressed**. He now presents severe dementia and is disabled with severe Parkinsonian symptoms, although he still lives at home with his family." (pg. 47)
- "Diagnosis: **Dementia** due to traumatic encephalopathy." (pg. 47)
- "Occupational adjustments related to the extent of the **neurological and psychiatric disability**. Eight patients (**1**, 6, 9, 11, 13, 15, 16, 17) were permanently unemployed." (pg. 48)
- In three cases (**1**, 16, 17) there was **dementia due to a progressive cerebral degenerative process**." (pg. 50)

Level of Functional Dependence / Dementia (Including Physical Disability): IV

Case 2 (Age: Not mentioned: Sport: Boxing)
Age started: Not mentioned
Age retired: Not Mentioned
Length of career: Not mentioned
Age of symptom onset: Not Mentioned
Delay between retirement and symptom onset (Age of symptom onset – Age retired): Not Mentioned

Core Clinical Features

- Cognitive Impairment: 1
  - "A **defect of memory** affecting the **retention and immediate recall of recent events** was present in 11 cases (**2**, 3, 5, 6, 7, 8, 9, 10, 11, 13, 15) and has usually been present since the end of the boxer's career." (pg. 47)
  - "**Mental torpor**, with a **slow, sluggish response to questions** and an **absence of spontaneity in conversation**, was present in three cases (**2**, 8, 15)." (pg. 47)
- Executive Function Impairment: NM
- Neurobehavioral Dysregulation: 0
  - "Seven cases (**2**, 3, 4, 5, 7, 12, 14) **remained well adjusted** in marriage and occupation and showed **no personality deviation**." (pg. 48)
- Progressive Course: Progressive
  - "Case 2 showed a **severe neurological, progressive disease**, but had a **normal air encephalogram** and **no evidence of brain damage on psychometric testing**. It is presumed that the responsible lesion in Case 2 was limited to the brain stem and did not interfere with higher cerebral function." (pg. 49)

Supportive Features

- Depression: NM
- Anxiety: NM
- Apathy: NM
- Paranoia: NM
- Suicidality: NM
- Motor Signs (Parkinsonism / ALS): 1
  - "**Progression in severity of the neurological symptoms** was evident in eight cases (1, **2**, 8, 11, 12, 15, 16, 17) and was confirmed in the four years of the follow-up… **Ataxia** combined with **festination of gait** were the most disabling features." (pgs. 45-47)
- Delayed Onset of Symptoms and Problems: NM

Level of Functional Dependence / Dementia: III
Level of Functional Dependence / Dementia (Including Physical Disability): III

Case 3 (Age: Not mentioned: Sport: Boxing)
Age started: Not mentioned
Age retired: Not Mentioned
Length of career: Not mentioned
Age of symptom onset: Not Mentioned
Delay between retirement and symptom onset (Age of symptom onset – Age retired): Not Mentioned

Core Clinical Features

- Cognitive Impairment: 1
  - "A **defect of memory** affecting the **retention and immediate recall of recent events** was present in 11 cases (2, **3**, 5, 6, 7, 8, 9, 10, 11, 13, 15) and has usually been present since the end of the boxer's career." (pg. 47)
  - "In Case 3 a **severe registration and retention defect** was present and resembled that seen in a Korsakov state, although other features of this syndrome were absent. The patient had been a heavy drinker early in his career and since **little evidence of the traumatic encephalopathy was present** it was felt that this retention defect was largely **attributable to alcohol**." (pg. 47)
- Executive Function Impairment: NM
- Neurobehavioral Dysregulation: 0
  - "Seven cases (2, **3**, 4, 5, 7, 12, 14) **remained well adjusted** in marriage and occupation and showed **no personality deviation**." (pg. 48)
- Progressive Course: Non-Progressive
  - "…there was **no evidence of progression of the neurological syndrome** in eight cases (**3**, 5, 6, 7, 9, 10, 13, 14)." (pg. 47)
  - "Cases **3**, 5, 6, 7, 9, 10, 13, 14 **had not deteriorated over the years of the follow-up of this study**, and indeed according to relatives' accounts they had **deteriorated little since the end of their fighting careers**." (pg. 52)

Supportive Features

- Depression: NM
- Anxiety: NM
- Apathy: NM
- Paranoia: NM
- Suicidality: NM
- Motor Signs (Parkinsonism / ALS): 1 (based on Figure 1, pg. 46)
  - Partial syndrome or borderline abnormal investigation -pyramidal degen. (pg. 46)
- Delayed Onset of Symptoms and Problems: NM

Level of Functional Dependence / Dementia: III
Level of Functional Dependence / Dementia (Including Physical Disability): III

Case 4 (Age: Not mentioned: Sport: Boxing)
Age started: Not mentioned
Age retired: Not Mentioned
Length of career: Not mentioned
Age of symptom onset: Not Mentioned
Delay between retirement and symptom onset (Age of symptom onset – Age retired): Not Mentioned

Core Clinical Features

- Cognitive Impairment: 0 (based on Figure 1, pg. 46)
- Executive Function Impairment: 0 (based on Figure 1, pg. 46)
- Neurobehavioral Dysregulation: 0
  - "Seven cases (2, 3, **4**, 5, 7, 12, 14) **remained well adjusted** in marriage and occupation and showed **no personality deviation**." (pg. 48)
- Progressive Course: Non-Progressive
  - "Case 4 was not considered to show any evidence of traumatic encephalography." (pg. 49)

Supportive Features

- Depression: 0 (based on Figure 1, pg. 46)
- Anxiety: 1
  - "Case 4, an amateur, developed anxiety symptoms following a domestic crisis and falsely attributed these to insidious 'punch drunkenness'. All investigations were normal and at follow-up he was symptom free." (pg. 45)
- Apathy: NM
- Paranoia: 0 (based on Figure 1, pg. 46)
- Suicidality: NM
- Motor Signs (Parkinsonism / ALS): 0 (based on Figure 1, pg. 46)
- Delayed Onset of Symptoms and Problems: 0 (based on Figure 1, pg. 46)

Level of Functional Dependence / Dementia: I
Level of Functional Dependence / Dementia (Including Physical Disability): I

Case 5 (Age: Not mentioned: Sport: Boxing)
Age started: Not mentioned
Age retired: Not Mentioned
Length of career: Not mentioned
Age of symptom onset: Not Mentioned
Delay between retirement and symptom onset (Age of symptom onset – Age retired): Not Mentioned

Core Clinical Features
- Cognitive Impairment: 1
  - "A **defect of memory** affecting the **retention and immediate recall of recent events** was present in 11 cases (2, 3, **5**, 6, 7, 8, 9, 10, 11, 13, 15) and has usually been present since the end of the boxer's career." (pg. 47)
- Executive Function Impairment: NM
- Neurobehavioral Dysregulation: 0
  - "Seven cases (2, 3, 4, **5,** 7, 12, 14) **remained well adjusted** in marriage and occupation and showed **no personality deviation**." (pg. 48)
- Progressive Course: Non-Progressive
  - "…there was **no evidence of progression of the neurological syndrome** in eight cases (3, **5**, 6, 7, 9, 10, 13, 14)." (pg. 47)
  - "Cases 3, **5**, 6, 7, 9, 10, 13, 14 **had not deteriorated over the years of the follow-up of this study**, and indeed according to relatives' accounts they had **deteriorated little since the end of their fighting careers**." (pg. 52)

Supportive Features
- Depression: NM
- Anxiety: NM
- Apathy: NM
- Paranoia: NM
- Suicidality: NM
- Motor Signs (Parkinsonism / ALS): 0 (based on Figure 1, pg. 46)
- Delayed Onset of Symptoms and Problems: NM

Level of Functional Dependence / Dementia: II
Level of Functional Dependence / Dementia (Including Physical Disability): II

Case 6 (Age: 60; Sport: Boxing)
Age started: Not mentioned
Age retired: 30 years
Length of career: Not mentioned
Age of symptom onset: 30 years
Delay between retirement and symptom onset (Age of symptom onset – Age retired): 0 years (i.e., no delay)

Core Clinical Features

- Cognitive Impairment: 1
  - "A **defect of memory** affecting the **retention and immediate recall of recent events** was present in 11 cases (2, 3, 5, **6**, 7, 8, 9, 10, 11, 13, 15) and has usually been present since the end of the boxer's career." (pg. 47)
  - "A 60-year-old light-weight, ex-professional boxer **retired at 30 years from the ring when he developed severe intention tremor of the righthand**, **ataxic gait** and **poor memory for recent events**. The condition **progressed for five years** but **has since been relatively stationary**… He was noted to have a **marked impairment in retentive memory**: there were no first rank symptoms of schizophrenia… **Apart from some recent further deterioration in his memory, the neurological and psychiatric syndromes have changed little over the past fifteen years**." (pg. 48)
- Executive Function Impairment: NM
- Neurobehavioral Dysregulation: 1
  - "At **45 years he became impotent**, although making excessive sexual demands upon his wife. **He then began to accuse her of being pregnant by his son, brother, nephew and later his neighbours**. He became **increasingly querulous and argumentative**, and was **finally admitted to a local psychiatric unit where he was diagnosed as 'paranoia due to brain injury**.' … Four E.C.T.s made him more irritable, and the treatment was abandoned and he returned home… Owing to his **quarrelsome attitudes** he has not worked for ten years." (pg. 48)
- Progressive Course: Non-Progressive
  - "…there was **no evidence of progression of the neurological syndrome** in eight cases (3, 5, **6**, 7, 9, 10, 13, 14)." (pg. 47)
  - "The condition **progressed for five years** but **has since been relatively stationary**… **Apart from some recent further deterioration in his memory, the neurological and psychiatric syndromes have changed little over the past fifteen years**." (pg. 48)
  - "Cases 3, 5, **6**, 7, 9, 10, 13, 14 **had not deteriorated over the years of the follow-up of this study**, and indeed according to relatives' accounts they had **deteriorated little since the end of their fighting careers**." (pg. 52)

Supportive Features

- Depression: NM
- Anxiety: NM
- Apathy: NM
- Paranoia: 1
  - "**Persistent accusations against the wife's supposed sexual infidelity** led to the primary **psychiatric referral** in five cases (1, **6**, 8, 9, 10)." The **morbid jealously persisted** in all cases at follow-up…" (pg. 48)
  - "At **45 years he became impotent**, although making excessive sexual demands upon his wife. **He then began to accuse her of being pregnant by his son, brother, nephew and later his neighbours**. He became **increasingly querulous and argumentative**, and was **finally admitted to a local psychiatric unit where he was diagnosed as 'paranoia due to brain injury**.' … Over the past fifteen years his family have tolerated his **incessant suspicion and accusations about his wife's supposed infidelity with neighbours**." (pg. 48)
  - "Diagnosis: Chronic symptomatic **paranoid psychosis**." (pg. 48)
  - "A **persistent psychosis** was present…Case 6… had a **chronic paranoid psychosis** which a **chronic amnesic state** due to underlying brain damage." (pgs. 48-49)
  - "Case 6 was a chronic organic psychosyndrome due to brain damage, with a florid delusional system relating to impotence and morbid jealousy of the wife." (pg. 51)
  - "In Case 6 morbid jealousy was a central persistent delusion of a chronic organic psychosis of 15 years' duration." (pg. 51)
- Suicidality: NM
- Motor Signs (Parkinsonism / ALS): 1
  - "A 60-year-old light-weight, ex-professional boxer **retired at 30 years from the ring when he developed severe intention tremor of the righthand**, **ataxic gait** and **poor memory for recent events**. The condition **progressed for five years** but **has since been relatively stationary**… **Apart from some recent further deterioration in his memory, the neurological and psychiatric syndromes have changed little over the past fifteen years**." (pg. 48)
- Delayed Onset of Symptoms and Problems: 0

- “A **defect of memory** affecting the **retention and immediate recall of recent events** was present in 11 cases (2, 3, 5, **6**, 7, 8, 9, 10, 11, 13, 15) and has usually been present since the end of the boxer’s career.” (pg. 47)

Level of Functional Dependence / Dementia: III

- “Owing to his **quarrelsome attitudes** he has not worked for ten years. **Apart from some recent further deterioration in his memory, the neurological and psychiatric syndromes have changed little over the past fifteen years**.” (pg. 48)
- “Occupational adjustments related to the extent of the **neurological and psychiatric disability**. Eight patients (1, **6**, 9, 11, 13, 15, 16, 17) were permanently unemployed.” (pg. 48)
- “Diagnosis: Chronic symptomatic **paranoid psychosis**.” (pg. 48)

Level of Functional Dependence / Dementia (Including Physical Disability): III

Case 7 (Age: Not mentioned: Sport: Boxing)
Age started: Not mentioned
Age retired: Not Mentioned
Length of career: Not mentioned
Age of symptom onset: Not Mentioned
Delay between retirement and symptom onset (Age of symptom onset – Age retired): Not Mentioned

Core Clinical Features

- Cognitive Impairment: 1
    - "A **defect of memory** affecting the **retention and immediate recall of recent events** was present in 11 cases (2, 3, 5, 6, **7**, 8, 9, 10, 11, 13, 15) and has usually been present since the end of the boxer's career." (pg. 47)
- Executive Function Impairment: NM
- Neurobehavioral Dysregulation: 0
    - "Seven cases (2, 3, 4, 5, **7**, 12, 14) **remained well adjusted** in marriage and occupation and showed **no personality deviation**." (pg. 48)
- Progressive Course: Non-Progressive
    - "…there was **no evidence of progression of the neurological syndrome** in eight cases (3, 5, 6, **7**, 9, 10, 13, 14)." (pg. 47)
    - "Cases 3, 5, 6, **7**, 9, 10, 13, 14 **had not deteriorated over the years of the follow-up of this study**, and indeed according to relatives' accounts they had **deteriorated little since the end of their fighting careers**." (pg. 52)

Supportive Features

- Depression: NM
- Anxiety: NM
- Apathy: NM
- Paranoia: NM
- Suicidality: NM
- Motor Signs (Parkinsonism / ALS): 1 (based on Figure 1, pg. 46)
    - "Pyramidal degen." (pg. 46)
- Delayed Onset of Symptoms and Problems: NM

Level of Functional Dependence / Dementia: II
Level of Functional Dependence / Dementia (Including Physical Disability): II

Case 8 (Age: Not mentioned; Sport: Boxing)
Age started: Not mentioned
Age retired: Not Mentioned
Length of career: Not mentioned
Age of symptom onset: Not Mentioned
Delay between retirement and symptom onset (Age of symptom onset – Age retired): Not Mentioned

Core Clinical Features

- Cognitive Impairment: 1
    - "A **defect of memory** affecting the **retention and immediate recall of recent events** was present in 11 cases (2, 3, 5, 6, 7, **8**, 9, 10, 11, 13, 15) and has usually been present since the end of the boxer's career." (pg. 47)
    - "**Mental torpor**, with a **slow, sluggish response to questions** and an **absence of spontaneity in conversation**, was present in three cases (2, **8**, 15)" (pg. 47)
- Executive Function Impairment: NM
- Neurobehavioral Dysregulation: 1
    - "**Persistent accusations against the wife's supposed sexual infidelity** led to the primary **psychiatric referral** in five cases (1, 6, **8**, 9, 10)." The **morbid jealously persisted** in all cases at follow-up…" (pg. 48)
    - "**Severe personality disorders** were present in four cases (1, **8**, 9, 10)." (pg. 48)
    - "Case 8 has a sensitive, querulous, suspicious type of personality and had always been 'touchy.' His **development of a morbid jealously syndrome in late life** was seen as a personality reaction related to brain damage." (pg. 48)
- Progressive Course: Progressive
    - "**Progression in severity of the neurological symptoms** was evident in eight cases (1, 2, **8**, 11, 12, 15, 16, 17) and was confirmed in the four years of the follow-up… **Ataxia** combined with **festination of gait** were the most disabling features." (pgs. 45-47)

Supportive Features

- Depression: 1
    - "Case 8 had an **episode of endogenous depression** at the age of 30 years which responded to E.C.T." (pg. 49)
- Anxiety: NM
- Apathy: NM
- Paranoia: 1
    - "**Persistent accusations against the wife's supposed sexual infidelity** led to the primary **psychiatric referral** in five cases (1, 6, **8**, 9, 10)." The **morbid jealously persisted** in all cases at follow-up." (pg. 48)
    - "**Severe personality disorders** were present in four cases (1, **8**, 9, 10)." (pg. 48)
    - "Case 8 has a sensitive, querulous, suspicious type of personality and had always been 'touchy.' His **development of a morbid jealously syndrome in late life** was seen as a personality reaction related to brain damage." (pg. 48)
    - "The patient's **accusations and suspicions of the wife's infidelity occurred episodically** and were seen as **abnormal psychogenic reactions** in sensitive personalities. It is difficult not to presume that impotence in these cases had played some part as a pathoplastic feature in determining this particular reaction." (pgs. 51-52)
- Suicidality: NM
- Motor Signs (Parkinsonism / ALS): 1
    - **"Ataxia** combined with **festination of gait** were the most disabling features." (pg. 47)
- Delayed Onset of Symptoms and Problems: NM

Level of Functional Dependence / Dementia: III
Level of Functional Dependence / Dementia (Including Physical Disability): III

Case 9 (Age: Not mentioned: Sport: Boxing)
Age started: Not mentioned
Age retired: Not Mentioned
Length of career: Not mentioned
Age of symptom onset: Not Mentioned
Delay between retirement and symptom onset (Age of symptom onset – Age retired): Not Mentioned

Core Clinical Features

- Cognitive Impairment: 1
  - "A **defect of memory** affecting the **retention and immediate recall of recent events** was present in 11 cases (2, 3, 5, 6, 7, 8, **9**, 10, 11, 13, 15) and has usually been present since the end of the boxer's career." (pg. 47)
- Executive Function Impairment: NM
- Neurobehavioral Dysregulation: 1
  - "**Severe personality disorders** were present in four cases (1, 8, **9**, 10). In three of these (1, **9**, 10) **impulsive aggressive behavior was prominent as a life-long trait** and justified the description of '**explosive, psychopathic personality**.' **Rage reaction**, **uncontrolled outbursts of anger and violence**, were prominent in the case histories of these men after their boxing careers and were attributed by them to their decreased tolerance to alcohol. All three cases had had numerous emergency **psychiatric admissions for impulsive acts of violence,** of short duration." (pg. 48)
  - "Aggression must be a prominent personality characteristic of any successful pugilist. Three cases (1, **9**, 10) had always been described as **sensitive, suspicious, impulsive and aggressive**, particularly when primed with alcohol, and all justified the label of "explosive psychopaths." (pg. 51)
- Progressive Course: Non-Progressive
  - "…there was **no evidence of progression of the neurological syndrome** in eight cases (3, 5, 6, 7, **9**, 10, 13, 14)." (pg. 47)
  - "Cases 3, 5, 6, 7, **9**, 10, 13, 14 **had not deteriorated over the years of the follow-up of this study**, and indeed according to relatives' accounts they had **deteriorated little since the end of their fighting careers**." (pg. 52)

Supportive Features

- Depression: NM
- Anxiety: NM
- Apathy: NM
- Paranoia: 1
  - "**Persistent accusations against the wife's supposed sexual infidelity** led to the primary **psychiatric referral** in five cases (1, 6, 8, **9**, 10). The **morbid jealously persisted** in all cases at follow-up, except in Case 1, who was by then severely demented." (pg. 48)
  - "**Transient paranoid-hallucinatory states** occurred in Cases **9** and 12. Case 9 has **twenty emergency psychiatric admissions** because of **aggressive post-ictal behavior**, **related to post-traumatic epilepsy** which commenced at the end of his boxing career." (pg. 49)
- Suicidality: NM
- Motor Signs (Parkinsonism / ALS): 1 (based on Figure 1, pg. 46)
  - "Pyramidal degen. and epilepsy" (pg. 46)
- Delayed Onset of Symptoms and Problems: NM

Level of Functional Dependence / Dementia: III

- "Occupational adjustments related to the extent of the **neurological and psychiatric disability**. Eight patients (1, 6, **9**, 11, 13, 15, 16, 17) were permanently unemployed." (pg. 48)

Level of Functional Dependence / Dementia (Including Physical Disability): III

Case 10 (Age: 40; Sport: Boxing)
Age started: Not mentioned Age retired: 33 years
Length of career: Not mentioned Age of symptom onset: Not mentioned
Delay between retirement and symptom onset (Age of symptom onset – Age retired): Not mentioned

Core Clinical Features
- Cognitive Impairment: 1
  - "A **defect of memory** affecting the **retention and immediate recall of recent events** was present in 11 cases (2, 3, 5, 6, 7, 8, 9, **10**, 11, 13, 15) and has usually been present since the end of the boxer's career." (pg. 47)
- Executive Function Impairment: NM
- Neurobehavioral Dysregulation: 1
  - "**Severe personality disorders** were present in four cases (1, 8, 9, **10**). In three of these (1, 9, **10**) **impulsive aggressive behavior was prominent as a life-long trait** and justified the description of '**explosive, psychopathic personality**.' **Rage reaction**, **uncontrolled outbursts of anger and violence**, were prominent in the case histories of these men after their boxing careers and were attributed by them to their decreased tolerance to alcohol. All three cases had had numerous emergency **psychiatric admissions for impulsive acts of violence,** of short duration." (pg. 48)
  - "He had **always** been **an impulsive, aggressive person.**" (pg. 48)
    - Note: The author mentions how this case had always been impulsive. Therefore, his violent outbursts may not be completely attributable to sequalae of repetitive neurotrauma.
  - "During his Army Service the patient was court-martialled and discharged as "psychopathic". He was **frequently involved in drunken brawls**, and was well known to become 'fighting mad' after a few drinks. **He had three prison sentences on account of his behaviour, and finally, while serving a prison sentence for attacking his second wife, he was transferred to a mental hospital under Section 72**. His first two marriages ended in divorce because of his **violent outbursts based upon his constant suspicion of his wives' infidelity, associated with his recurrent impotence**. His third marriage, since leaving hospital, is so far well adjusted, although his sensitive, suspicious attitudes have to be tactfully countered by his wife." (pg. 48)
  - "Diagnosis: **Aggressive psychopathic personality** associated with brain damage and alcoholism." (pg. 48)
  - "Aggression must be a prominent personality characteristic of any successful pugilist. Three cases (1, 9, 10) had always been described as **sensitive, suspicious, impulsive and aggressive**, particularly when primed with alcohol, and all justified the label of "explosive psychopaths." (pg. 51)
- Progressive Course: Non-Progressive
  - "…there was **no evidence of progression of the neurological syndrome** in eight cases (3, 5, 6, 7, 9, **10,** 13, 14)." (pg. 47)
  - "According to records of his previous neurological examinations **these symptoms are not progressive**." (pg. 48)
  - "Cases 3, 5, 6, 7, 9, **10**, 13, 14 **had not deteriorated over the years of the follow-up of this study**, and indeed according to relatives' accounts they had **deteriorated little since the end of their fighting careers**." (pg. 52)

Supportive Features
- Depression: NM
- Anxiety: NM
- Apathy: NM
- Paranoia: 1
  - "**Persistent accusations against the wife's supposed sexual infidelity** led to the primary **psychiatric referral** in five cases (1, 6, 8, 9, **10**). The **morbid jealously persisted** in all cases at follow-up…" (pg. 48)
  - "His first two marriages ended in divorce because of his **violent outbursts based upon his constant suspicion of his wives' infidelity, associated with his recurrent impotence**. His third marriage, since leaving hospital, is so far well adjusted, although his sensitive, suspicious attitudes have to be tactfully countered by his wife." (pg. 48)
  - "The patient's **accusations and suspicions of the wife's infidelity occurred episodically** and were seen as **abnormal psychogenic reactions** in sensitive personalities. It is difficult not to presume that impotence in these cases had played some part as a pathoplastic feature in determining this particular reaction." (pgs. 51-52)
    Note: The author notes that "it is difficult not to presume that impotence in these cases had played some part as a pathoplastic feature in determining [morbid jealousy and/or accusations and suspicions of the wife's infidelity] in these brain-damaged boxers." (pgs. 51-52)
- Suicidality: NM
- Motor Signs (Parkinsonism / ALS): 1
  - **"He has a marked amnesia**, **slurred speech** and **bilateral cerebellar signs in his upper limbs**." (pg. 48)
- Delayed Onset of Symptoms and Problems: NM

Level of Functional Dependence / Dementia: III
- "Diagnosis: **Aggressive psychopathic personality** associated with brain damage and alcoholism." (pg. 48)

Level of Functional Dependence / Dementia (Including Physical Disability): III

Case 11 (Age: Not mentioned; Sport: Boxing)
Age started: Not mentioned
Age retired: Not Mentioned
Length of career: Not mentioned
Age of symptom onset: Not Mentioned
Delay between retirement and symptom onset (Age of symptom onset – Age retired): Not Mentioned

Core Clinical Features

- Cognitive Impairment: 1
  - "A **defect of memory** affecting the **retention and immediate recall of recent events** was present in 11 cases (2, 3, 5, 6, 7, 8, 9, 10, **11**, 13, 15) and has usually been present since the end of the boxer's career." (pg. 47)
- Executive Function Impairment: NM
- Neurobehavioral Dysregulation: 0 (based on Figure 1 and the fact that other cases were described as having some aspects of neurobehavioral dysregulation, pg. 46)
- Progressive Course: Progressive
  - "**Progression in severity of the neurological symptoms** was evident in eight cases (1, 2, 8, **11**, 12, 15, 16, 17) and was confirmed in the four years of the follow-up… **Ataxia** combined with **festination of gait** were the most disabling features." (pgs. 45-47)

Supportive Features

- Depression: NM
- Anxiety: NM
- Apathy: NM
- Paranoia: NM
- Suicidality: NM
- Motor Signs (Parkinsonism / ALS): 1
  - "**Progression in severity of the neurological symptoms** was evident in eight cases (1, 2, 8, **11**, 12, 15, 16, 17) and was confirmed in the four years of the follow-up… **Ataxia** combined with **festination of gait** were the most disabling features." (pgs. 45-47)
- Delayed Onset of Symptoms and Problems: NM

Level of Functional Dependence / Dementia: III

- "Occupational adjustments related to the extent of the **neurological and psychiatric disability**. Eight patients (1, 6, 9, **11**, 13, 15, 16, 17) were permanently unemployed." (pg. 48)

Level of Functional Dependence / Dementia (Including Physical Disability): III

Case 12 (Age: Not mentioned; Sport: Boxing)
Age started: Not mentioned
Age retired: Not Mentioned
Length of career: Not mentioned
Age of symptom onset: Not Mentioned
Delay between retirement and symptom onset (Age of symptom onset – Age retired): Not Mentioned

Core Clinical Features

- Cognitive Impairment: 0
  - "Discordance of these two features was particularly obvious in Case 12, where progressive neurological symptoms rendered the patient a physical invalid, yet there was no evidence of psychic impairment either clinically or psychometrically." (pg. 47)
- Executive Function Impairment: NM
- Neurobehavioral Dysregulation: 0
  - "Seven cases (2, 3, 4, 5, 7, **12**, 14) **remained well adjusted** in marriage and occupation and showed **no personality deviation**. Case 12 was particularly outstanding in his normal personality adjustment in spite of **severe, progressive neurological disability**." (pg. 48)
- Progressive Course: Progressive
  - "**Progression in severity of the neurological symptoms** was evident in eight cases (1, 2, 8, 11, **12**, 15, 16, 17) and was confirmed in the four years of the follow-up." (pg. 45)

Supportive Features

- Depression: NM
- Anxiety: NM
- Apathy: NM
- Paranoia: 0
  - "**Transient paranoid-hallucinatory states** occurred in Cases 9 and **12**… Case 12 had a single acute paranoid-hallucinatory psychosis after taking Preludin for slimming; there was no residual personality defect." (pg. 49)
    Note: It seems that this psychosis was due to the medication side effects and not a neurological or psychiatric condition.
- Suicidality: NM
- Motor Signs (Parkinsonism / ALS): 1
  - "**Progression in severity of the neurological symptoms** was evident in eight cases (1, 2, 8, 11, **12**, 15, 16, 17) and was confirmed in the four years of the follow-up…**Ataxia combined with festination of gait** were the most disabling features, and in Case 12 had led to **invalidism in a motorized chair**." (pgs. 45-47)
- Delayed Onset of Symptoms and Problems: NM

Level of Functional Dependence / Dementia: II

- "In Case 12 had led to invalidism in a motorized chair." (pg. 47)
- "Discordance of these two features was particularly obvious in Case 12, where progressive neurological symptoms rendered the patient a physical invalid, yet there was no evidence of psychic impairment either clinically or psychometrically." (pg. 47)

Level of Functional Dependence / Dementia (Including Physical Disability): III

Case 13 (Age: Not mentioned: Sport: Boxing)
Age started: Not mentioned
Age retired: Not Mentioned
Length of career: Not mentioned
Age of symptom onset: Not Mentioned
Delay between retirement and symptom onset (Age of symptom onset – Age retired): Not Mentioned

Core Clinical Features

- Cognitive Impairment: 1
  - "A **defect of memory** affecting the **retention and immediate recall of recent events** was present in 11 cases (2, 3, 5, 6, 7, 8, 9, 10, 11, **13**, 15) and has usually been present since the end of the boxer's career." (pg. 47)
- Executive Function Impairment: NM
- Neurobehavioral Dysregulation: 0 (based on Figure 1 and the fact that other cases were described as having some aspects of neurobehavioral dysregulation, pg. 46)
- Progressive Course: Non-Progressive
  - "There was **no evidence of progression of the neurological syndrome** in eight cases (3, 5, 6, 7, 9, 10, **13**, 14)." (pg. 47)
  - "Cases 3, 5, 6, 7, 9, 10, **13**, 14 **had not deteriorated over the years of the follow-up of this study**, and indeed according to relatives' accounts they had **deteriorated little since the end of their fighting careers**." (pg. 52)

Supportive Features

- Depression: NM
- Anxiety: NM
- Apathy: NM
- Paranoia: NM
- Suicidality: NM
- Motor Signs (Parkinsonism / ALS): 1 (based on Figure 1, pg. 46)
  - "Pyramidal degen. and cerebellar" (pg. 46)
- Delayed Onset of Symptoms and Problems: NM

Level of Functional Dependence / Dementia: III

- "Occupational adjustments related to the extent of the **neurological and psychiatric disability**. Eight patients (1, 6, 9, 11, **13**, 15, 16, 17) were permanently unemployed." (pg. 48)

Level of Functional Dependence / Dementia (Including Physical Disability): III

Case 14 (Age: Not mentioned: Sport: Boxing)
Age started: Not mentioned
Age retired: Not Mentioned
Length of career: Not mentioned
Age of symptom onset: Not Mentioned
Delay between retirement and symptom onset (Age of symptom onset – Age retired): Not Mentioned

Core Clinical Features

- Cognitive Impairment: 0 (based on Figure 1, pg. 46)
- Executive Function Impairment: NM
- Neurobehavioral Dysregulation: 0
  - "Seven cases (2, 3, 4, 5, 7, 12, **14**) **remained well adjusted** in marriage and occupation and showed **no personality deviation**." (pg. 48)
- Progressive Course: Non-Progressive
  - "…there was **no evidence of progression of the neurological syndrome** in eight cases (3, 5, 6, 7, 9, 10, 13, **14**)." (pg. 47)
  - "Cases 3, 5, 6, 7, 9, 10, 13, **14 had not deteriorated over the years of the follow-up of this study**, and indeed according to relatives' accounts they had **deteriorated little since the end of their fighting careers**." (pg. 52)

Supportive Features

- Depression: NM
- Anxiety: NM
- Apathy: NM
- Paranoia: NM
- Suicidality: NM
- Motor Signs (Parkinsonism / ALS): 1 (based on Figure 1, pg. 46)
  - Partial syndrome or borderline abnormal investigation- pyramidal degen. (pg. 46)
- Delayed Onset of Symptoms and Problems: NM

Level of Functional Dependence / Dementia: I
Level of Functional Dependence / Dementia (Including Physical Disability): II

Case 15 (Age: Not mentioned; Sport: Boxing)
Age started: Not mentioned
Age retired: Not Mentioned
Length of career: Not mentioned
Age of symptom onset: Not Mentioned
Delay between retirement and symptom onset (Age of symptom onset – Age retired): Not Mentioned

Core Clinical Features

- Cognitive Impairment: 1
  - "A **defect of memory** affecting the **retention and immediate recall of recent events** was present in 11 cases (2, 3, 5, 6, 7, 8, 9, 10, 11, 13, **15**) and has usually been present since the end of the boxer's career." (pg. 47)
  - "**Mental torpor**, with a **slow, sluggish response to questions** and an **absence of spontaneity in conversation**, was present in three cases (2, 8, **15**)." (pg. 47)
- Executive Function Impairment: NM
- Neurobehavioral Dysregulation: 0 (based on Figure 1 and the fact that other cases were described as having some aspects of neurobehavioral dysregulation, pg. 46)
- Progressive Course: Progressive
  - "**Progression in severity of the neurological symptoms** was evident in eight cases (1, 2, 8, 11, 12, **15**, 16, 17) and was confirmed in the four years of the follow-up… **Ataxia** combined with **festination of gait** were the most disabling features." (pgs. 45-47)

Supportive Features

- Depression: NM
- Anxiety: NM
- Apathy: NM
- Paranoia: NM
- Suicidality: NM
- Motor Signs (Parkinsonism / ALS): 1
  - "**Progression in severity of the neurological symptoms** was evident in eight cases (1, 2, 8, 11, 12, **15**, 16, 17) and was confirmed in the four years of the follow-up… **Ataxia** combined with **festination of gait** were the most disabling features." (pgs. 45-47)
- Delayed Onset of Symptoms and Problems: NM

Level of Functional Dependence / Dementia: III

- "Occupational adjustments related to the extent of the **neurological and psychiatric disability**. Eight patients (1, 6, 9, 11, 13, **15**, 16, 17) were permanently unemployed." (pg. 48)

Level of Functional Dependence / Dementia (Including Physical Disability): III

Case 16 (Age: Not mentioned; Sport: Boxing)
Age started: Not mentioned
Age retired: Not Mentioned
Length of career: Not mentioned
Age of symptom onset: Not Mentioned
Delay between retirement and symptom onset (Age of symptom onset – Age retired): Not Mentioned

Core Clinical Features

- Cognitive Impairment: 1
  - "**Mental torpor**, with a **slow, sluggish response to questions** and an **absence of spontaneity in conversation**, was present in three cases (2, 8, 15), although it was a more **prominent feature** in the cases with **dementia** (1, **16**, 17)." (pg. 47)
  - "Three cases (1, 16, 17) suffered from **post-traumatic dementia** with **mental torpor** and **flat affective responses.**" (pg. 47)
  - "In Cases 1 and **16**, **dementia was paralleled by progressive neurological disability**." (pg. 47)
  - "In three cases (1, **16**, 17) there was **dementia due to a progressive cerebral degenerative process**." (pg. 50)
  - "Case 16 was **so severely affected both intellectually and neurologically** that it was felt unjustified to carry out air encephalography on him." (pg. 47)
- Executive Function Impairment: 1
  - "**Mental torpor**, with a **slow, sluggish response to questions** and an **absence of spontaneity in conversation**, was present in three cases (2, 8, 15), although it was a more **prominent feature** in the cases with **dementia** (1, **16**, 17)." (pg. 47)
  - "Three cases (1, **16**, 17) suffered from **post-traumatic dementia** with **mental torpor** and **flat affective responses.**" (pg. 47)
- Neurobehavioral Dysregulation: 0 (based on Figure 1 and the fact that other cases were described as having some aspects of neurobehavioral dysregulation, pg. 46)
- Progressive Course: Progressive
  - "**Progression in severity of the neurological symptoms** was evident in eight cases (1, 2, 8, 11, 12, 15, **16**, 17) and was confirmed in the four years of the follow-up. In Case 16 the neurological disability was **confirmed as progressive** from the clinical history in hospital. **Ataxia combined with festination of gait** were the most disabling features" (pgs. 45-47)
  - "In those with progressive neurological disorder only two (**1** and 16) had **progressive psychiatric disability.**" (pg. 47)

Supportive Features

- Depression: NM
- Anxiety: NM
- Apathy: NM
- Paranoia: NM
- Suicidality: NM
- Motor Signs (Parkinsonism / ALS): 1
  - "**Ataxia combined with festination of gait** were the most disabling features." (pg. 47)
- Delayed Onset of Symptoms and Problems: NM

Level of Functional Dependence / Dementia: IV

- "Cases **16** and 17 were **permanently in hospital**" (pg. 47)
- "Case 16 was **so severely affected both intellectually and neurologically** that it was felt unjustified to carry out air encephalography on him." (pg. 47)
- "Occupational adjustments related to the extent of the **neurological and psychiatric disability**. Eight patients (1, 6, 9, 11, 13, 15, **16**, 17) were permanently unemployed." (pg. 48)

Level of Functional Dependence / Dementia (Including Physical Disability): IV

Case 17 (Age: 50; Sport: Boxing)
Age started: Not mentioned
Age retired: Not Mentioned
Length of career: Not mentioned
Age of symptom onset: Not Mentioned
Delay between retirement and symptom onset (Age of symptom onset – Age retired): Not Mentioned

Core Clinical Features

- Cognitive Impairment: 1
    - "**Mental torpor**, with a **slow, sluggish response to questions** and an **absence of spontaneity in conversation**, was present in three cases (2, 8, 15), although it was a more **prominent feature** in the cases with **dementia** (1, 16, **17**)." (pg. 47)
    - "Three cases (1, 16, **17**) suffered from **post-traumatic dementia** with **mental torpor** and **flat affective responses.**" (pg. 47)
    - "He has had 80 E.C.T.s, and ten years ago he had a leucotomy, following which he developed epilepsy. At operation the brain was noted to be **atrophic**. Although he has **deteriorated intellectually** and socially over this period he has become placid with the help of phenothiazines." (pg. 49)
    - "Mentally, he was **disoriented in time and place and showed severe impairment for learning and recalling new information**. Mental torpor was severe, and he held loose confabulatory delusions. He was deteriorated in his social and personal habits." (pg. 49)
    - "Psychometric testing gave evidence of brain damage and **intellectual deterioration**." (pg. 49)
    - "In three cases (1, 16, **17**) there was **dementia due to a progressive cerebral degenerative process**." (pg. 50)
- Executive Function Impairment: 1
    - "**Mental torpor**, with a **slow, sluggish response to questions** and an **absence of spontaneity in conversation**, was present in three cases (2, 8, 15), although it was a more **prominent feature** in the cases with **dementia** (1, 16, **17**)." (pg. 47)
    - "Three cases (1, 16, **17**) suffered from **post-traumatic dementia** with **mental torpor** and **flat affective responses.**" (pg. 47)
- Neurobehavioral Dysregulation: 1
    - "On admission he was described as **inert and apathetic** for long periods, but was prone to long periods of **aggression** during which he hallucinated and deluded and would eat faeces and swallow cutlery." (pg. 49)
- Progressive Course: Progressive
    - "**Progression in severity of the neurological symptoms** was evident in eight cases (1, 2, 8, 11, 12, 15, 16, **17**) and was confirmed in the four years of the follow-up. (pg. 45)

Supportive Features

- Depression: NM
- Anxiety: NM
- Apathy: 1
    - "Three cases (1, 16, **17**) suffered from **post-traumatic dementia** with **mental torpor** and **flat affective responses.**" (pg. 47)
    - "On admission he was described as **inert and apathetic** for long periods, but was prone to long periods of aggression during which he hallucinated and deluded and would eat faeces and swallow cutlery." (pg. 49)
- Paranoia: 1 (based on Figure 1, pg. 46)
    - "A **persistent psychosis** was present in two cases (6 and 17)." (pg. 48)
    - "A 50-year-old, single, amateur champion and later professional welter-weight, developed symptoms of acute catatonic schizophrenia after a championship fight in London; he improved with E.C.T. and deep insulin. Two years later he was readmitted to hospital, where he has remained for 17 years." (pg. 49)
    - "Mental torpor was severe, and he held **loose confabulatory delusions**. He was deteriorated in his social and personal habits." (pg. 49)
    - "Diagnosis: (i) Schizophrenic defect state..." (pg. 49)
- Suicidality: NM
- Motor Signs (Parkinsonism / ALS): 1
    - "On examination, he showed incoordination in the **upper limbs, slurred dysarthria, and Parkinsonism** (probably due to phenothiazines)." (pg. 49)
- Delayed Onset of Symptoms and Problems: 1

Level of Functional Dependence / Dementia: V

- "Cases 16 and **17** were **permanently in hospital**" (pg. 47)

- "Occupational adjustments related to the extent of the **neurological and psychiatric disability**. Eight patients (1, 6, 9, 11, 13, 15, 16, **17**) were permanently unemployed." (pg. 48)
- "Two new patients (16, **17**) who were in a local mental hospital have been added because of their psychiatric interest." (pg.45)
- "Diagnosis: (i) Schizophrenic defect state. (ii) Chronic organic psychosyndrome probably due to a traumatic encephalopathy caused by boxing and/or physical treatment." (pg. 49)
- "In Case 17 there is some doubt about the nature of the encephalopathy, and it could be argued that the neurological, psychometric and neuroradiological evidence of brain damage was due to previous physical forms of treatment superimposed upon a schizophrenic defect state." (pgs. 50-51)
- "In Case 17, brain damage was demonstrated clinically, radiologically and psychometrically, but none of the findings were typical of the traumatic encephalopathy, and they could equally well be attributed to the physical treatment. The present mental state was a combination of an organic psychosyndrome and schizophrenic defect state. On balance it was felt that this was a case of process schizophrenia precipitated by head injury in an individual who was already genetically predisposed." (pg. 51)

Level of Functional Dependence / Dementia (Including Physical Disability): V

Roberts (1969)

Note: As is noted by Gardner et al. (2014) and Victoroff et al. (2013), Roberts reports 37 cases. However, he only provides details on 11 of these cases (Cases 1-11). Therefore, we only included these 11 cases for the purposes of this review.

Note: For these cases, the evidence for a progressive course is very limited to a comment made by the boxers' wives or colleagues, the progression of only a one/a couple symptoms, or the appearance of a couple more symptoms over time. Roberts' general tone is that the disease does not have a progressive course, or progresses only very slowly, except for a few cases.

Case 1 (Age: 60; Sport: Boxing)
Age started: 15 years (professional at 16)
Age retired: 29 years
Length of career: 13 years
Age of symptom onset: Not specified, but likely within his last year of boxing, see below
Delay between retirement and symptom onset (Age of symptom onset – Age retired): Not specified, but symptom onset was likely during career

Core Clinical Features

- Cognitive Impairment: 1
    - "He had been **forgetful for many years**…" (pg. 28)
    - "Examined he was found to be devoid of insight and euphoric..." (pg. 28)
    - "He was **demented** as judged by the **extreme slowness or this thinking and his striking inability to register, during the course of the interview, various pieces of information**, including greetings from another boxer whom he had known well, in spite of the fact that these were several times repeated to him. His recall of details of his boxing career seemed accurate, as far as names and places were concerned, but was **hopelessly inaccurate for dates**, even those when he was champion. Tested with Raven's Matrices he scores in the fiftieth percentile, and in the twenty-fifth percentile with the Mill Hill Vocabulary. **These tests, used on his initial admission to hospital and repeated seven years later, did not show any decline in the scores, nor had his dementia, as judges clinically and by his behavior generally, progresses notably in that time**." (pg. 28)
    - "In the first case described it seemed likely that his disability, due to the extra-pyramidal lesion, had **become more obvious as he grew older**, but it was **difficult to be sure that this indicated something other than the normal degenerative processes of ageing**. His **dementia had clearly progressed over the years**, and the **development of a paranoid illness** in the setting of his dementia was a certain measure of this." (pg. 44)
- Executive Function Impairment: 1
    - "Examined he was found to be devoid of insight and euphoric..." (pg. 28)
    - "He was **demented** as judged by the **extreme slowness or this thinking and his striking inability to register, during the course of the interview, various pieces of information**, including greetings from another boxer whom he had known well, in spite of the fact that these were several times repeated to him. His recall of details of his boxing career seemed accurate, as far as names and places were concerned, but was **hopelessly inaccurate for dates**, even those when he was champion. Tested with Raven's Matrices he scores in the fiftieth percentile." (pg. 28)
- Neurobehavioral Dysregulation: NM
- Progressive Course: Progressive
    - "In the first case described it seemed likely that his disability, due to the extra-pyramidal lesion, had **become more obvious as he grew older**, but it was **difficult to be sure that this indicated something other than the normal degenerative processes of ageing**. His **dementia had clearly progressed over the years**, and the **development of a paranoid illness** in the setting of his dementia was a certain measure of this." (pg. 44)

Supportive Features

- Depression: NM
- Anxiety: NM
- Apathy: NM
- Paranoia: 1
    - "The death of his wife precipitated a **paranoid delusional illness** which necessitate his admission, finally for long-term care, to a mental hospital." (pg. 28)
    - "Examined he was found to be devoid of insight and euphoric, but it was possible to elicit some poorly systematized paranoid delusions about his wife's relatives who he felt had dispossessed him of his inheritance." (pg. 28)
    - "His **dementia had clearly progressed over the years**, and the **development of a paranoid illness** in the setting of his dementia was a certain measure of this." (pg. 44)
- Suicidality: NM

- Motor Signs (Parkinsonism / ALS): 1
    - "He had no serious injuries or illness in the past, apart from, towards the end of his career, what was probably cellulitis of the hand which he held responsible for the **tremor** he had been aware of since." (pg. 28)
        - Note: His hand tremor is presented as a consequence of cellulitis and not a neurological symptom of repetitive neurotrauma. Roberts doesn't say whether it is due to one or the other.
    - "His relatives had noticed that his **speech was slurred** and that he had **developed a tremor in his left arm**, and his manager that he was **limping with his left leg in his last year boxing**. He had been **forgetful for many years**, and the **tremor in his left arm and the dragging of his left leg had become more apparent** as he grew older." (pg. 28)
    - "His **speech was slurred, his face expressionless**, and there was **an increase in tone more marked in the left than the right limbs**. **All his movements were slow** and he was **markedly akinetic on attempting to walk**, **his gait thereafter short-paced and hemi-paretic with the left arm partially flexed and the leg intorted and dragging**. He was **unsteady on his feet and unable to walk tandem** fashion but there was **only dubious pyramidal weakness of the left limbs, no ataxia**. There was **an intermittent rhythmical parkinsonian rest tremor of the left limbs**. The **tendon reflexes were brisker in the left arm than the right** and **both plantar responses were extensor**. He was **normotensive and apart from a few scattered rhonchi** in his chest general examination was unremarkable." (pg. 29)
    - "In the first case described it seemed likely that his disability, due to the extra-pyramidal lesion, had **become more obvious as he grew older**, but it was **difficult to be sure that this indicated something other than the normal degenerative processes of ageing**. His **dementia had clearly progressed over the years**, and the **development of a paranoid illness** in the setting of his dementia was a certain measure of this." (pg. 44)
- Delayed Onset of Symptoms and Problems: 0 (Not specified, but likely occurred during the course of his career)
    - "His relatives had noticed that his **speech was slurred** and that he had **developed a tremor in his left arm**, and his manager that he was **limping with his left leg in his last year boxing**." (pg. 28)

Level of Functional Dependence / Dementia: IV

- "This man, aged 60, an ex-laborer and store-keeper, had been unemployed for thirteen years and a patient in a mental hospital for seven [years]." (pg. 27)
- "The death of his wife precipitated a **paranoid delusional illness** which necessitate his admission, finally for long-term care, to a mental hospital." (pg. 28)

Level of Functional Dependence / Dementia (Including Physical Disability): IV

Case 2 (Age: 50; Sport: Boxing)
Age started: 12 years (professional at 18)
Age retired: Likely 31 years (10-year professional career followed by 3 years in boxing booths)
Length of career: 19 years
Age of symptom onset: Likely 28 years (since his last promoted (i.e., professional) fight)
Delay between retirement and symptom onset (Age of symptom onset – Age retired): -3 years*
*Symptom onset during career

Core Clinical Features
- Cognitive Impairment: 0
    - "**Although his memory did not seem to have deteriorated…"** (pg. 30)
    - "His **memory**, as judged by his recall of the details of his career and the development of his symptoms, **was not grossly impaired**, but formal psychometric tests were not done." (pg. 30)
- Executive Function Impairment: NM
- Neurobehavioral Dysregulation: 1
    - "Examined, he was found to be **emotionally labile, tearful and laughing alternately during the course of the interview**." (pg. 30)
- Progressive Course: Progressive
    - "**His condition had remained little changed** except that he had become increasingly sensitive to alcohol, small quantities apparently making him very drunk, until the **gradual development ten years later of a progressively worsening tremor of his limbs**." (pg. 30)
    - "The history was **quite clear on the point of progression** in two of these, the **second** and fourth cases [Case 4], in that an **extra-pyramidal tremor had made its appearance some years later** on a background of established dysarthria first noticed during their last years boxing." (pg. 44)

Supportive Features
- Depression: NM
- Anxiety: NM
- Apathy: NM
- Paranoia: NM
- Suicidality: NM
- Motor Signs (Parkinsonism / ALS): 1
    - "His wife had noticed that **his speech had been slurred** and the **he had been unsteady on his feet since his last promoted fight** in which he had received a severe beating. He had been admitted to hospital for observation at the time and had **remained unwell for several days**. **His condition had remained little changed** except that he had become increasingly sensitive to alcohol, small quantities apparently making him very drunk, until the **gradual development ten years later of a progressively worsening tremor of his limbs**." (pg. 30)
    - "His **speech was so slurred as to be almost unintelligible**, **his face was impassive**, and there was a **severe asymmetrical parkinsonian tremor of all four limbs, left more than right at rest, increased by emotion and abolished on volition. Tremor also affected his head and trunk**, his **gait was unsteady and festinant**, and **his limbs were rigid**, **more so on the left than the right**. **All tendon reflexes were pathologically brisk**, there was **sustained clonus at knees and ankles**, and **both plantar responses were extensor**. The **visual acuity in the left eye was impaired to perception of finger movements by an extensive retinal detachment** and **conjugate elevation of the eyes was poor**." (pg. 30)
    - "The history was **quite clear on the point of progression** in two of these, the **second** and fourth cases [Case 4], in that an **extra-pyramidal tremor had made its appearance some years later** on a background of established dysarthria first noticed during their last years boxing." (pg. 44)
- Delayed Onset of Symptoms and Problems: 0
    - "His wife had noticed that **his speech had been slurred** and the **he had been unsteady on his feet since his last promoted fight** in which he had received a severe beating. " (pg. 30)

Level of Functional Dependence / Dementia: III
- "This man, aged 50, an ex-labourer and merchant seaman, had been unemployed for eight years." (pg. 29)

Level of Functional Dependence / Dementia (Including Physical Disability): III

Case 3 (Age: 58; Sport: Boxing)
Age started: 14 years (professional at 15)
Age retired: 30 years
Length of career: 16 years
Age of symptom onset: Not specified (The past ten years (i.e., since age 48) and perhaps longer, see below)
Delay between retirement and symptom onset (Age of symptom onset – Age retired): Not specified

Core Clinical Features

- Cognitive Impairment: 2
    - "Examined, it was apparent that his **mentation was extremely slow** and that he had **difficulty recalling names and places in his boxing career**. He scored in the fiftieth percentile on Raven's Matrices, learned his digit span of seven plus one at the fourth repetition, recalled accurately only one of the pair of Binet designs at the first attempt, but both at the second presentation and both accurately after fifteen minutes delay." (pg. 31)
- Executive Function Impairment: NM
- Neurobehavioral Dysregulation: NM
- Progressive Course: Progressive
    - "Both he and his wife had been aware that **his speech, for at least the past ten years and perhaps longer,** had been **'thick and getting thicker.'**" (pg. 31)

Supportive Features

- Depression: NM
- Anxiety: NM
- Apathy: NM
- Paranoia: NM
- Suicidality: NM
- Motor Signs (Parkinsonism / ALS): 1
    - "Both he and his wife had been aware that **his speech, for at least the past ten years and perhaps longer,** had been **'thick and getting thicker'**" (pg. 31)
    - **"His cerebellar dysarthria was so marked as to make his speech almost unintelligible at times**, there was a **mild degree of ataxia of gait on beginning to walk**, **on getting out of a chair and on turning, and the left leg was slightly circumducted walking**. There was a **terminal intention tremor in both arms and all movements were markedly slow**. **An inconstant rhythmical tremor, apparent at rest and suppressed on volition, was evident in the right arm when he was engaged in tests of intellectual function**. The only focal weakness present was in **the right triceps which was wasted and this was associated with a depressed triceps tendon reflex**. The **left plantar response was extensor**. He was **normotensive and general examination was otherwise unremarkable**." (pg. 31)
- Delayed Onset of Symptoms and Problems: 1
    - "Both he and his wife had been aware that **his speech, for at least the past ten years and perhaps longer,** had been **'thick and getting thicker.'**" (pg. 31)
    - "He retired at the age of thirty when he began slowing up and losing too many fights." (pg. 31)

Level of Functional Dependence / Dementia: III

- "This man, aged 58, had been a skilled craftsman and had built up a small but successful business on his own when he gave up boxing." (pg. 30)

Level of Functional Dependence / Dementia (Including Physical Disability): III

Case 4 (Age: 48; Sport: Boxing)
Age started: 9 years (professional at 18)
Age retired: 30 years
Length of career: 21 years
Age of symptom onset: Not specified ("speech thick since his last years in boxing," see below)
Delay between retirement and symptom onset (Age of symptom onset – Age retired): Not specified

Core Clinical Features

- Cognitive Impairment: 0
  - "He **felt that this memory had deteriorated in recent years**, so that he had great difficulty remembering people's names and errands his wife gave him." (pg. 32)
  - "Examined, there was **no overt intellectual abnormality** and he appeared to have **good insight into his minor disabilities**. He scored in the twenty-fifth percentile on Raven's Matrices and on the synonym selection test of the Mill Hill Vocabulary. He learnt his digit span plus one at the third repetition, but his immediate recall of one of a pair of Binet designs was inaccurate, though after fifteen minutes delay he was able to reproduce faultlessly one of the original designs and his own version of the other." (pg. 32)
- Executive Function Impairment: NM
- Neurobehavioral Dysregulation: 0
  - "His wife had been aware of no alteration in this speech or personality…" (pg. 32)
- Progressive Course: Progressive
  - "The history was **quite clear on the point of progression** in two of these, the second and **fourth** cases, in that an **extra-pyramidal tremor had made its appearance some years later** on a background of established dysarthria first noticed during their last years boxing." (pg. 44)

Supportive Features

- Depression: NM
- Anxiety: NM
- Apathy: NM
- Paranoia: NM
- Suicidality: NM
- Motor Signs (Parkinsonism / ALS): 1
  - "When, during the course of the examination, **an inconstant parkinsonian tremor of his right arm was drawn to his attention, he claimed it had been present only for a few months**." (pg. 32)
  - "His **speech was slurred, his face expressionless, and there was an inconstant rhythmical parkinsonian tremor of his right arm at rest, abolished on volition**. The **tendon reflexes were brisker in the right than the left arm but the tone was only marginally increased in the right arm and both plantar responses were flexor**. There was **no apparent disequilibrium**, and his **gait was normal**. He was **normotensive** and, apart from clinical evidence of chronic bronchitis, **general examination was unremarkable**." (pg. 33)
- Delayed Onset of Symptoms and Problems: NM

Level of Functional Dependence / Dementia: II

- "This man, aged 48, a dockyard worker, had been an unskilled manual worker in various trades all his life." (pg. 31)

Level of Functional Dependence / Dementia (Including Physical Disability): II

Case 5 (Age: 53; Sport: Boxing)
Age started: 15 years (professional at 18)
Age retired: Not mentioned
Length of career: 10 years (professional only)
Age of symptom onset: Not mentioned
Delay between retirement and symptom onset (Age of symptom onset – Age retired): Not mentioned

Core Clinical Features

- Cognitive Impairment: 1
  - "The records made by the neurologist who had examined him twenty years earlier, during the war, were available. At the time his **speech was slurred** and **he was complaining of headaches**, **loss of memory and depression**. An independent account was also available from his present general practitioner who had known him as a **chronic alcoholic for many years**." (pg. 33)
  - "Examined, **he appeared apathetic, his speech slurred and his mentation slow**. **His memory for the details of his professional career was very poor**, judging by the record he had kept, and he had considerable difficulty grasping news of former colleagues mentioned to him during the course of the interview. No formal psychometric tests were used, but according to his own account of his poor scholastic performance at school **he was constitutionally below average intelligence**, **though his evident memory difficulties and slow mentation were a certain measure of a degree of dementia superimposed."** (pg. 33)
- Executive Function Impairment: NM
- Neurobehavioral Dysregulation: 1
  - "He drank excessively and had done for years… He had finally been divorced by his wife because of his **increasingly trying behavior**." (pg. 33)
  - "An independent account was also available from his present general practitioner who had known him as a **chronic alcoholic for many years**." (pg. 33)
  - "General examination was precluded by the patient's unwillingness to co-operate further." (pg. 34)
- Progressive Course: Unable to Classify

Supportive Features

- Depression: 1
  - "The records made by the neurologist who had examined him twenty years earlier, during the war, were available. At the time his **speech was slurred** and **he was complaining of headaches**, **loss of memory and depression**. An independent account was also available from his present general practitioner who had known him as a **chronic alcoholic for many years**." (pg. 33)
- Anxiety: NM
- Apathy: 1
  - "Examined, **he appeared apathetic, his speech slurred and his mentation slow**…" (pg. 33)
- Paranoia: NM
- Suicidality: NM
- Motor Signs (Parkinsonism / ALS): 1
  - "**In addition to his dysarthria, his face was expressionless, he was markedly unsteady on his feet and there was a moderate ataxia in the left more than the right arm.** There was **some spasticity in the pronators of the forearms and wrists, on the left more than the right**, the **tendon reflexes were brisker in the left limbs**, and the **left plantar response was extensor**." (pg. 34)
  - "…it seemed that the **ataxia** and evidence of **pyramidal lesions were new developments**, though the **dysarthria and sluggish mentation were not**." (pg. 45)
- Delayed Onset of Symptoms and Problems: NM

Level of Functional Dependence / Dementia: IV

- "He was discharged from the services as punchdrunk, had done various labouring jobs for some years thereafter and been unemployed again for the past nine years." (pg. 33)

Level of Functional Dependence / Dementia (Including Physical Disability): IV

Case 6 (Age: 57; Sport: Boxing)
Age started: 17 years
Age retired: 27 years
Length of career: 10 years
Age of symptom onset: 27 years
Delay between retirement and symptom onset (Age of symptom onset – Age retired): 0 years*
*No delay

Core Clinical Features

- Cognitive Impairment: 1
  - **"…until his mining accident. Since then his speech and gait had progressively deteriorated and his memory, always poor, had become so defective that in recent years he did not know what day it was**. The professional colleague, who had known him well, did **not think there had been much change in the years immediately following the mining accident**, and said it had been **well recognized locally that the man had been made punch drunk** by the champion heavy weights he had sparred with for too long." (pg. 35)
  - "Examined, he was **euphoric and evidently demented, but insight was retained and he asserted that he was punchdrunk** and that 'punchdrunk is a terrible thing.' His **mentation was markedly slow** and he was **unable to recall any details of his boxing career without considerable prompting**. He was **disorientated in time and place.** He scored in the twenty-fifth percentile in Raven's Matrices and in the synonym selection test of the Mill Hill Vocabulary. His digit span was six but he was quite unable to learn another digit in addition, and although his copy of the Binet designs was accurate, he was unable to recall either." (pg. 35)
- Executive Function Impairment: NM
- Neurobehavioral Dysregulation: 1
  - **"In recent years he had become increasingly irritable and liable to outbursts of uncontrollable temper**...." (pg. 35)
- Progressive Course: Progressive
  - "His wife and a professional colleague, who were interviewed, and the individual himself had been aware that he **dribbled and that his speech was slurred and his gait unsteady since his last years in the ring**. The wife felt that his **speech had remained unchanged,** and certainly his **dribbling had cleared up, until his mining accident. Since then his speech and gait had progressively deteriorated and his memory, always poor, had become so defective that in recent years he did not know what day it was**. The professional colleague, who had known him well, did **not think there had been much change in the years immediately following the mining accident**, and said it had been **well recognized locally that the man had been made punch drunk** by the champion heavy weights he had sparred with for too long**. In recent years he had become increasingly irritable and liable to outbursts of uncontrollable temper**, and **two years earlier had had a severe paranoid delusional illness**, making unfounded accusations against his wife." (pg. 35)

Supportive Features

- Depression: NM
- Anxiety: NM
- Apathy: NM
- Paranoia: 1
  - **"Two years earlier had had a severe paranoid delusional illness**, making unfounded accusations against his wife." (pg. 35)
- Suicidality: NM
- Motor Signs (Parkinsonism / ALS): 1
  - "His wife and a professional colleague, who were interviewed, and the individual himself had been aware that he **dribbled and that his speech was slurred and his gait unsteady since his last years in the ring**. The wife felt that his **speech had remained unchanged,** and certainly his **dribbling had cleared up, until his mining accident. Since then his speech and gait had progressively deteriorated and his memory, always poor, had become so defective that in recent years he did not know what day it was**." (pg. 35)
  - "There was a **mild dysphasia, his speech was so slurred as to be occasionally unintelligible**, his **face was impassive,** and there was **nystagmus on lateral gaze.** His **gait was unsteady and high-stepping and there was titubation of head and trunk. All four limbs were ataxic, more so in the right arm and left leg.** There was **slight spasticity in the pronators of the right forearm and right quadriceps**, the **tendon reflexes were brisker on that side**, and **both plantar responses were indefinite extensors**" (pg. 35)
  - "A small area of sacral sensory loss, depressed ankle and absent left knee jerks, together with his urinary incontinence, which necessitates wearing a urinal, were sequelae of the conus or root lesions caused by the mining accident." (pg. 35) Note: Roberts (1969) identifies these symptoms as sequalae of this patient's mining accident, and does not attribute them to repetitive neurotrauma.

    - “General examination was unremarkable apart from evidence of chronic bronchitis and a blood pressure of 145/105.” (pg. 35)
- Delayed Onset of Symptoms and Problems: 0
    - “He retired from the ring, at the age of twenty-seven, when his wife and friends told him he was **getting ‘punchy.’**” (pg. 34)

Level of Functional Dependence / Dementia: IV

- “This man, aged 57, a miner all his life apart from a period of three years when he had lived entirely on the proceeds of his boxing, had been unemployed for twenty years since a mining accident.” (pg. 34)
- “Examined, he was **euphoric and evidently demented, but insight was retained and he asserted that he was punchdrunk** and that ‘punchdrunk is a terrible thing.’ His **mentation was markedly slow** and he was **unable to recall any details of his boxing career without considerable prompting**. He was **disorientated in time and place.”** (pg. 35)

Level of Functional Dependence / Dementia (Including Physical Disability): IV

Case 7 (Age: 57; Sport: Boxing)
Age started: 12 years (professional at 18)
Age retired: 26 years
Length of career: 12 years
Age of symptom onset: Not mentioned
Delay between retirement and symptom onset (Age of symptom onset – Age retired): Not mentioned

Core Clinical Features

- Cognitive Impairment: 0
  - "Examined, **apart from his poor recall of the details of his boxing career**, there was **no overt clinical evidence of intellectual defect**. He scores at the fiftieth percentile in the Raven's Matrices and in the synonym selection test of the Mill Hill Vocabulary, learned his digit span of five plus one at the second repetition and recalled accurately, both immediately and after fifteen minutes delay, both Binet designs." (pg. 36)
- Executive Function Impairment: NM
- Neurobehavioral Dysregulation: NM
- Progressive Course: Progressive
  - "The evidence for progression in these lesions among this group [cases similar to Case 7, not described in detail by Roberts (1969)] was poor except in the case of the individual reported in detail [Case 7] whose **condition seemed to have worsened**, **or at least become more noticeable** to his relatives, after an apparently trivial head injury." (pg. 45)

Supportive Features

- Depression: NM
- Anxiety: NM
- Apathy: NM
- Paranoia: NM
- Suicidality: NM
- Motor Signs (Parkinsonism / ALS): 1
  - "His wife had noticed **for years that his speech was slurred when he was excited**, but thought that **this had become much more obvious since his mild head injury**, **since when she had also noticed that he was unsteady on his feet and tended to drag his left leg**." (pg. 36)
    Note: A description of the mild head injury, referred to above, is provided here: "a minor head injury, which did not cause loss of consciousness, incapacitated him with **headaches and phobic symptoms**, necessitating his retirement [from being a driver]." (pg. 35-36)
  - "His **speech was markedly slurred,** his **gait was unsteady with a mild degree of truncal ataxia,** and there was an **asymmetrical ataxia of the left limbs more than the right**. He was **generally hypotonic**, though the **tendon reflexes were brisk**, **more so on the left than the right**. **Both plantar responses were flexor**. **The only weakness present was characteristic of a ruptured supraspinatus tendon. General examination was unremarkable** apart from a blood pressure of 145/100." (pg. 36)
- Delayed Onset of Symptoms and Problems: NM

Level of Functional Dependence / Dementia: II

- "This man, aged 57, had been a labourer and subsequently a driver for many years until a minor head injury, which did not cause loss of consciousness, incapacitated him with headaches and phobic symptoms, necessitating his retirement." (pg. 35)

Level of Functional Dependence / Dementia (Including Physical Disability): II

Case 8 (Age: 45; Sport: Boxing)
Age started: 11 years (professional at 17)
Age retired: Not specified, likely 30 years (13-year professional career starting at age 17)
Length of career: 13 years (professional only)
Age of symptom onset: Not mentioned
Delay between retirement and symptom onset (Age of symptom onset – Age retired): Not mentioned

Core Clinical Features

- Cognitive Impairment: 0
  - "**His wife commented that he was forgetful and agreed that his speech was slurred**, **but did not think there had been any change in the latter or in his equable temperament since she had known him a year before he became a professional boxer**. **He himself had noted with surprise that his speech was slurred** when he had tape recorded himself, but did not think it was due to boxing, and was aware of no other disabilities." (pg. 37-38)
  - "Examined, **he seemed mildly euphoric**, but there was **no clinical suggestion of intellectual impairment, and his memory, as judged by his detailed recall of his career, was good**. He scored in the ninety-fifth percentile in Raven's Matrices, and in the fiftieth percentile in the synonym selection test of the Mill Hill Vocabulary. He learned his digit span of six plus one at the third repetition, and his recall of the Binet designs, both immediate and delayed, was accurate." (pg. 38)
- Executive Function Impairment: NM
- Neurobehavioral Dysregulation: 0
  - "**His wife commented that he was forgetful and agreed that his speech was slurred**, **but did not think there had been any change in the latter or in his equable temperament since she had known him a year before he became a professional boxer**." (pg. 37)
- Progressive Course: Non-Progressive

Supportive Features

- Depression: NM
- Anxiety: NM
- Apathy: NM
- Paranoia: NM
- Suicidality: NM
- Motor Signs (Parkinsonism / ALS): 1
  - "**His wife commented that he was forgetful and agreed that his speech was slurred**, **but did not think there had been any change in the latter or in his equable temperament since she had known him a year before he became a professional boxer**. **He himself had noted with surprise that his speech was slurred** when he had tape recorded himself, but did not think it was due to boxing, and was aware of no other disabilities." (pg. 37-38)
  - "There was an **obvious slurring dysarthria**, his **face was expressionless**, and **in repose a mild inconstant ocular divergence made him appear glassy eyed**. **All his movements were slow**, and **repetitive movements of his arms were a little clumsily performed**, but there was **no definite ataxia.** He was **unsteady walking tandem and on turning**, and his **gait was mildly spastic**, **as were his forearm pronators and both quadriceps, more so in the left limbs than the right**. The **tendon reflexes were pathologically but symmetrically brisk**, there were **a few beats of clonus at both ankles**, but the **plantar responses were flexor and the jaw jerk normal**. There was **no weakness and no sensory deficit**. He was normotensive and **general examination was unremarkable**." (pg. 38)
  - "In cases 8 and 9 it was this poverty and slowness of movement, together with the dysarthria and unsteadiness of gait, that dominated the clinical picture." (pg. 45)
- Delayed Onset of Symptoms and Problems: NM

Level of Functional Dependence / Dementia: I

- "This man, aged 45, had been a street trader and salesman most of his life and had lately become a sales supervisor." (pg. 37)

Level of Functional Dependence / Dementia (Including Physical Disability): I

Case 9 (Age: 58; Sport: Boxing)
Age started: 13 years (professional at 19)
Age retired: 28 years
Length of career: Not mentioned
Age of symptom onset: Not specified (unsteady on feet during last years boxing, see below)
Delay between retirement and symptom onset (Age of symptom onset – Age retired): No delay (see Age of symptom onset)

Core Clinical Features

- Cognitive Impairment: 0
  - "He was aware that **his memory was poor**." (pgs. 39-40)
  - "Examined, there was **no impression of intellectual defect clinically,** and he was **notably insightful and frank.** He recalled the details of his boxing career accurately except for the dates. He learnt his digit span of six plus one at the fifth repetition, and his immediate recall of the two Binet designs was accurate, though he was unable to recall one of the figures accurately after fifteen minutes delay. His immediate recall of a flower, a colour and an address were accurate. He scored in the fiftieth percentile in the Raven's Matrices and in the twenty-fifth on the synonym selection test of the Mill Hill Vocabulary." (pg. 40)
- Executive Function Impairment: NM
- Neurobehavioral Dysregulation: NM
- Progressive Course: Unable to Classify
  - "A philanthropic admirer, who had followed closely his boxing career to the end, had offered him the job telling him it was pitiful to see what had become of him." (pg. 39)
  - "He himself was not aware of this but had noticed that he was unsteady on his feet during his last years boxing and that this had become worse in recent years so that he often lost his balance." (pg. 39)

Supportive Features

- Depression: NM
- Anxiety: NM
- Apathy: NM
- Paranoia: NM
- Suicidality: NM
- Motor Signs (Parkinsonism / ALS): 1
  - "**His wife considered him punchdrunk in that his speech was slurred when he was excited**, and **he dribbled and had done for years**. He himself was not aware of this but had noticed that he was **unsteady on his feet during his last years boxing and that this had become worse in recent years so that he often lost his balance**. He was aware that **his memory was poor**." (pgs. 39-40)
  - "His **speech was slurred and his gait unsteady, particularly on turning**. There was **slight clumsiness, not amounting to definite ataxia, in all four limbs, but no weakness**. **Slight spasticity was evident in both quadriceps** and the **tendon reflexes were pathologically but symmetrically brisk**. The **left plantar response was an indefinite extensor and the right flexor**. He was normotensive, and **apart from clinical evidence of severe chronic bronchitis and emphysema general examination was unremarkable**." (pg. 40)
  - "In cases 8 and 9 it was this poverty and slowness of movement, together with the dysarthria and unsteadiness of gait, that dominated the clinical picture." (pg. 45)
- Delayed Onset of Symptoms and Problems: 0
  - "He himself was not aware of this but had noticed that he was unsteady on his feet during his last years boxing and that this had become worse in recent years so that he often lost his balance." (pg. 39)

Level of Functional Dependence / Dementia: I

- "This man, aged 58, originally a labourer, had been a driver for thirty years since his retirement from the ring." (pg. 39)
- "A philanthropic admirer, who had followed closely his boxing career to the end, had offered him the job telling him it was pitiful to see what had become of him." (pg. 39)

Level of Functional Dependence / Dementia (Including Physical Disability): I

Case 10 (Age: 35; Sport: Boxing)
Age started: 11 years (professional at 19)
Age retired: Not mentioned
Length of career: Not mentioned
Age of symptom onset: Not specified (Speech slurred about the time he retired, see below)
Delay between retirement and symptom onset (Age of symptom onset – Age retired): Not mentioned, possibly no delay (see Age of symptom onset)

Core Clinical Features

- Cognitive Impairment: 0
  - "**His wife was aware of no change in his amiable and equable temperament** in the years she had known him, **considered his memory as good as her own** and **had never seen him unsteady on his feet**..." (pg. 41)
  - "Examined, he was **undoubtedly jovial but by no means pathologically so**, and there was **no clinical impression of intellectual defect**. His history of his boxing career was accurate and detailed. He learnt his digit span of seven plus one at the fifth repetition. His immediate and delayed recall of the two Binet designs was accurate. He scored at the twenty-fifth percentile in Raven's Matrices, and at the fiftieth in the synonym selection test of the Mill Hill Vocabulary" (pg. 41)
- Executive Function Impairment: NM
- Neurobehavioral Dysregulation: 0
  - "**His wife was aware of no change in his amiable and equable temperament** in the years she had known him…" (pg. 41)
- Progressive Course: Non-Progressive
  - "There was **no suggestion of progression** in any of these cases [Cases 10 and 11 and seven similar cases]." (pg. 46)

Supportive Features

- Depression: NM
- Anxiety: NM
- Apathy: NM
- Paranoia: NM
- Suicidality: NM
- Motor Signs (Parkinsonism / ALS): 1
  - "The **only evidence of neurological lesions was a slight, but quite definite, slurring dysarthria, and an extensor plantar response on the left**." (pg. 41)
- Delayed Onset of Symptoms and Problems: 0
  - "She admitted that **she had noticed that his speech was slurred at about the time he retired from boxing, but did not think there had been any change, for better or worse, since**." (pg. 41)

Level of Functional Dependence / Dementia: II

- "This man, aged 35, at one time a window cleaner and labourer, subsequently took a correspondence course to learn a trade and had then built up a successful small business on his own when he retired from the ring." (pg. 40)

Level of Functional Dependence / Dementia (Including Physical Disability): II

Case 11 (Age: 39; Sport: Boxing)
Age started: 17 years (professional at 17)
Age retired: Not mentioned
Length of career: Not mentioned
Age of symptom onset: Not specified, but likely at the time of retirement as he retired "when, on medical grounds, renewal of his license was refused." (pg. 42)
Delay between retirement and symptom onset (Age of symptom onset – Age retired): No delay

Core Clinical Features

- Cognitive Impairment: 1
    - "An independent account was not available from his wife, but the findings of a neurologist, who examined him when the question of renewal of his license was under consideration **fifteen years earlier**, were. **He had been noted then to be dysarthric and very slow in cerebration**. **He himself was aware that his memory was appalling** having for several years to write everything down he needed to remember, and commented on the fact that **it had always astonished him how slurred his speech was when he heard it on tape recordings**." (pgs. 42-43)
    - "Examined, **it was evident that his memory, judged by his recall of the details of his boxing career, was poor**, indeed the area championship he claimed to have held was not the one he had. **His mentation was not, however, notably slow**, and he scored in the fiftieth percentile in Raven's Matrices and in the twenty-fifth in the synonym selection test of the Mill Hill Vocabulary. He learned his digit span of seven plus one at the second repetition but was unable to recall accurately, either immediately or after fifteen minutes delay, more than one of the pair of Binet designs." (pg. 43)
- Executive Function Impairment: NM
- Neurobehavioral Dysregulation: NM
- Progressive Course: Non-Progressive
    - "There was **no suggestion of progression** in any of these cases [Cases 10 and 11 and seven similar cases]." (pg. 46)

Supportive Features

- Depression: NM
- Anxiety: NM
- Apathy: NM
- Paranoia: NM
- Suicidality: NM
- Motor Signs (Parkinsonism / ALS): 2
    - "He had a **severe slurring dysarthria, but there were no other abnormal physical signs in his central nervous system** and he was clearly otherwise extremely fit." (pg. 43)
- Delayed Onset of Symptoms and Problems: 0
    - "He retired from the ring when, on medical grounds, renewal of his license was refused." (pg. 42)

Level of Functional Dependence / Dementia: III

- "This man, aged 39, had been a clerk before, and a labourer after, his boxing career. In recent years he had built up a successful small business in a competitive trade." (pg. 42)

Level of Functional Dependence / Dementia (Including Physical Disability): III

Corsellis, Bruton, and Freeman-Browne (1973)

Case 1 (Age: 63; Sport: Boxing)
Age started: 11 years
Age retired: Not mentioned, likely 25 years
Length of career: 14 years
Age of symptom onset: Not mentioned, likely in his 20s (see below)
Delay between retirement and symptom onset (Age of symptom onset – Age retired): Not mentioned

Core Clinical Features

- Cognitive Impairment: 1
  - "As a young man he was quiet, generous, and abstemious. He married in his early 20s… soon his life became more hectic and 'he changed completely.' He **wenched and drank and gambled heavily**. His **memory began to fail him**." (pg. 271)
  - "His brother remarked that 'his brain was not functioning – he made **mistakes in reckoning**.' He could not settle in a job and he became **vagrant**." (pg. 271)
- Executive Function Impairment: 1
  - "His brother remarked that 'his brain was not functioning – he made **mistakes in reckoning**.' He could not settle in a job and he became **vagrant**." (pg. 271)
- Neurobehavioral Dysregulation: 1
  - "As a young man he was quiet, generous, and abstemious. He married in his early 20s… soon his life became more hectic and 'he changed completely.' He **wenched and drank and gambled heavily**." (pg. 271)
  - "His marriage broke up and he **drifted away from his family.**" (pg. 271)
  - "He had **violent outbursts**, he was **'knocked out' by only a small amount of alcohol**, his **behavior was 'disgusting.'** His brother remarked that 'his brain was not functioning – he made **mistakes in reckoning**.' He could not settle in a job and he became **vagrant**." (pg. 271)
- Progressive Course: Progressive

Supportive Features

- Depression: NM
- Anxiety: NM
- Apathy: NM
- Paranoia: NM
- Suicidality: NM
- Motor Signs (Parkinsonism / ALS): 2
  - "At the age of 62 he was found lying neglected and louse-ridden in the boiler house of a hotel. In hospital he could answer questions to the point but his **speech was slurred and indistinct**. He was continent. A routine physical examination revealed a **right-sided ptosis** and a **cataract of the right eye**. He was **unsteady on his feet**." (pg. 271)
- Delayed Onset of Symptoms and Problems: NM

Level of Functional Dependence / Dementia: IV

- "He could not settle in a job and he became **vagrant**." (pg. 271)
- "At the age of 62 he was found lying neglected and louse-ridden in the boiler house of a hotel. In hospital he could answer questions to the point but his **speech was slurred and indistinct**. He was continent. A routine physical examination revealed a **right-sided ptosis** and a **cataract of the right eye**. He was **unsteady on his feet**." (pg. 271)

Level of Functional Dependence / Dementia (Including Physical Disability): IV

Case 2 (Age: 77; Sport: Boxing)
Age started: Not specified, "boxed from boyhood" (272)
Age retired: early 30s
Length of career: Not mentioned
Age of symptom onset: Not mentioned, likely during or before his earl 30s (see below)
Delay between retirement and symptom onset (Age of symptom onset – Age retired): No delay

Core Clinical Features

- Cognitive Impairment: 1
  - "**During the following decade he became childlike**, often wanting to be cuddled and reassured. He **suffered two head injuries** which were not severe but **they may have aggravated the deterioration in memory and in behaviour.**" (pg. 273)
  - "**When he was 67** he developed an acute appendicitis and was noticed to be **confused, incontinent of urine, and disoriented; his memory was severely impaired**. **His manner was still childish**; he would sulk when left alone and would smile happily when approached. He retained some idea of his boxing cusses but had forgotten the details." (pg. 273)
- Executive Function Impairment: 1
  - "**During the following decade he became childlike**, often wanting to be cuddled and reassured. He **suffered two head injuries** which were not severe but **they may have aggravated the deterioration in memory and in behaviour.**" (pg. 273)
  - "**When he was 67** he developed an acute appendicitis and was noticed to be **confused, incontinent of urine, and disoriented; his memory was severely impaired**. **His manner was still childish**; he would sulk when left alone and would smile happily when approached. He retained some idea of his boxing cusses but had forgotten the details." (pg. 273)
- Neurobehavioral Dysregulation: 1
  - "**During the following decade he became childlike**, often wanting to be cuddled and reassured. He **suffered two head injuries** which were not severe but **they may have aggravated the deterioration in memory and in behaviour.**" (pg. 273)
- Progressive Course: Progressive

Supportive Features

- Depression: NM
- Anxiety: NM
- Apathy: NM
- Paranoia: NM
- Suicidality: NM
- Motor Signs (Parkinsonism / ALS): 1
  - "**By the age of 50 he staggered** slightly when he walked and **his speech was slow and slurred.**" (pg. 273)
  - "He **spoke indistinctly**. He was **markedly ataxic,** walking on a wide base and often stumbling. He **could not stand on one leg** and **heel-toe walking led him to fall to the right**. He had **nystagmus to the right and a tremor of his upper limbs**. Muscle power, tendon reflexes, pupillary responses, and blood pressure were all normal." (pg. 273)
- Delayed Onset of Symptoms and Problems: 0
  - "He retired in his early 30s after a severe battering and was **described at that time as 'a little old man**… playing the part of an animated punching bag…'" (pg. 272)

Level of Functional Dependence / Dementia: IV

- "He died demented and doubly incontinent, in a psychiatric hospital at the age of 77." (pg. 273)

Level of Functional Dependence / Dementia (Including Physical Disability): IV

Case 3 (Age: 63; Sport: Boxing)
Age started: 16 years
Age retired: Not specified, likely just soon before 30 years (see below)
Length of career: 13 years
Age of symptom onset: Not specified, likely around time of retirement, according to wife (see below)
Delay between retirement and symptom onset (Age of symptom onset – Age retired): No delay

Core Clinical Features

- Cognitive Impairment: 1
  - "His wife met him shortly after this and she recalled that **'he had already begun to get a bit muddled.**'" (pg. 274)
  - "…his **memory was poor…**" (pg. 275)
  - "It was concluded in hospital that he had '**diffuse degenerative brain disease affecting extrapyramidal and pyramidal systems as well as causing mental impairment**.'" (pg. 275)
- Executive Function Impairment: NM
- Neurobehavioral Dysregulation: 1
  - "His **wife recalled that he had a bad temper**, '**the scenes were awful**,' '**he raved at night**' but was 'so kind and nice afterwards." (pg. 275)
- Progressive Course: Progressive

Supportive Features

- Depression: NM
- Anxiety: NM
- Apathy: NM
- Paranoia: 1
  - "He suffered a neck injury (unrelated to boxing) when about 26 and boxed less after this, becoming a manager when approaching 30. His wife met him shortly after this and she recalled that **'he had already begun to get a bit muddled.**' **By the age of 36 he would often fall backwards. He began to suspect his mother of theft and his wife of infidelity**." (pgs. 274-275)
- Suicidality: NM
- Motor Signs (Parkinsonism / ALS): 1
  - "He was seen in hospital **when aged 44** because of back pain. Osteoarthritis of his cervical vertebrae was found on x-ray; **a rigid gait was noted, both arms were slightly spastic; early Parkinsonism was queried**." (pg. 275)
  - "**At 50, slurring of speech** was mentioned and the term **'punch-drunk'** was used." (pg. 275)
  - "**Six years later** a **neurological report included an extra-pyramidal tremor of both hands and ataxia of both legs**. There was a **slight dysarthria**." (pg. 275)
  - "A further report, **when he was 61**, stated that **his unsteadiness and shaking hands had become worse**. He was '**dribbling a bit,**' he had **Parkinsonian facies**, his **speech was difficult to understand…**" (pg. 275)
  - "It was concluded in hospital that he had '**diffuse degenerative brain disease affecting extrapyramidal and pyramidal systems as well as causing mental impairment**.' He **could not talk or swallow properly**; he had **several falls**." (pg. 275)
- Delayed Onset of Symptoms and Problems: 0

Level of Functional Dependence / Dementia: IV

- "It was concluded in hospital that he had '**diffuse degenerative brain disease affecting extrapyramidal and pyramidal systems as well as causing mental impairment**.' He **could not talk or swallow properly**; he had **several falls** and after the last one he died from bronchitis and bronchopneumonia when aged 63." (pg. 275)

Level of Functional Dependence / Dementia (Including Physical Disability): IV

Case 4 (Age: 69; Sport: Boxing)
Age started: 15 years
Age retired: 40 years
Length of career: 25 years
Age of symptom onset: mid-30s
Delay between retirement and symptom onset (Age of symptom onset – Age retired): Symptom onset during career

Core Clinical Features
- Cognitive Impairment: 1
  - "As a young man he was considered shrewd and not unintelligent." (pg. 276)
  - "**At the age of 64** he was reinvestigated in a neurosurgical unit. A **history of progressive unsteadiness on his feet and mental deterioration with impairment of memory were described by his wife**. He **lost all sense of date and time** and **did not recognize his relatives**…He was considered **mildly demented**." (pg. 276)
- Executive Function Impairment: NM
- Neurobehavioral Dysregulation: NM
- Progressive Course: Progressive

Supportive Features
- Depression: NM
- Anxiety: NM
- Apathy: NM
- Paranoia: NM
- Suicidality: NM
- Motor Signs (Parkinsonism / ALS): 1
  - "In his **mid-30s he became unsteady on his feet**, and **after the age of 40 he had a shuffling gait**. His **legs were apt to give way** and he **developed a marked tremor of the hands**. He had **difficulty in dressing himself**." (pg. 276)
  - "**When he was 57** he attended hospital for backache and **was found to have a spastic gait and slurred speech**, both of which were recorded as having been present for some years. **At the age of 64** he was reinvestigated in a neurosurgical unit. A **history of progressive unsteadiness on his feet and mental deterioration with impairment of memory were described by his wife**. He **lost all sense of date and time** and **did not recognize his relatives**. His **speech was dysarthric**, he had a **wide-based ataxic gait,** and **Romberg's sign was positive.** He was considered **mildly demented**. **No tremor was recorded**. Air studies showed general enlargement of the ventricles without displacement; the septum was not mentioned, and the **punch-drunk syndrome and spinal osteoarthritis were diagnosed**. **When aged 69** he entered hospital with acute retention of urine. He had a **tremor of both legs and a mask-like facies**. He developed a chest infection and died three weeks later." (pgs. 276-277)
- Delayed Onset of Symptoms and Problems: 0
  - "In his **mid-30s he became unsteady on his feet**, and **after the age of 40 he had a shuffling gait**." (pg. 276)

Level of Functional Dependence / Dementia: IV
- "He retired from boxing when he was 40 years old and worked as a carpenter for 15 years. He was then registered as a disabled person and could only manage unskilled work." (pg. 276)
- "He was considered **mildly demented**." (pg. 276)

Level of Functional Dependence / Dementia (Including Physical Disability): IV

Case 5 (Age: 61; Sport: Boxing)
Age started: As a child (professional at 18)
Age retired: Not mentioned
Length of career: 18 years
Age of symptom onset: about 30 years (see below)
Delay between retirement and symptom onset (Age of symptom onset – Age retired): Symptom onset during career

Core Clinical Features

- Cognitive Impairment: 1
  - "**Towards the end of his career at the age of about 30 he was no longer able to look after his personal affairs**. He **became enuretic when about 35 and eventually had to be cared for in a home**. He was noted to be **'irascible, forgetful, but clean and tidy and quite nimble on his feet**.'" (pg. 278)
- Executive Function Impairment: NM
- Neurobehavioral Dysregulation: 1
  - "He was always aggressive and spiteful and was so unpopular with the crowds that they liked to see him beaten… He drank excessively in his 20s" (pg. 278)
- Progressive Course: Progressive

Supportive Features

- Depression: NM
- Anxiety: NM
- Apathy: NM
- Paranoia: NM
- Suicidality: NM
- Motor Signs (Parkinsonism / ALS): 2
  - **"When 60 years old he developed a right facial weakness and a right homonymous hemianopia**." (pg. 278)
- Delayed Onset of Symptoms and Problems: 0

Level of Functional Dependence / Dementia: IV

- "In the same year, and a few months before he died, he was **transferred to a psychiatric hospital**. Death was attributed to bronchopneumonia and cardiac ischaemia." (pg. 278)

Level of Functional Dependence / Dementia (Including Physical Disability): IV

Case 6 (Age: 83; Sport: Boxing)
Age started: 13 years
Age retired: Not specified, likely 38 years ("six years later" (pg. 279) from the age of 32)
Length of career: 25 years
Age of symptom onset: 32 years
Delay between retirement and symptom onset (Age of symptom onset – Age retired): -6 years*
*Symptom onset during career

Core Clinical Features

- Cognitive Impairment: 1
  - "He was fit **until the age of 32 when his legs 'began to give way', he developed a 'hoping-dancing walk', and his speech became slurred**. His memory was good." (pg. 279)
  - "On admission he was **orientated but his memory was poor**. He **thought people were robbing him**. He was **dysarthric, and ataxic, with a slow shuffling gait**. He had a **Parkinsonian look and some weakness of his left leg**. He was **incontinent of urine**. His death, at 83, was attributed to bronchopneumonia." (pg. 279)
- Executive Function Impairment: NM
- Neurobehavioral Dysregulation: 1
  - "His **two marriages broke up**, largely because of his **violent nature**. When 65 years old he went to live with his son but **aggressive outbursts led to his removal to an old people's home**. He became more subdued but he still had the occasional scuffle and one of these brought him to a **psychiatric hospital**." (pg. 279)
- Progressive Course: Progressive

Supportive Features

- Depression: NM
- Anxiety: NM
- Apathy: NM
- Paranoia: 1
  - "He **thought people were robbing him**." (pg. 279)
- Suicidality: NM
- Motor Signs (Parkinsonism / ALS): 1
  - "He was fit **until the age of 32 when his legs 'began to give way', he developed a 'hoping-dancing walk', and his speech became slurred**." (pg. 279)
  - "He was **dysarthric, and ataxic, with a slow shuffling gait**. He had a **Parkinsonian look and some weakness of his left leg**. He was **incontinent of urine**. His death, at 83, was attributed to bronchopneumonia." (pg. 279)
- Delayed Onset of Symptoms and Problems: 0

Level of Functional Dependence / Dementia: III

- "He retired from boxing six years later and became a referee and a promoter. Eventually he took any job he could get." (pg. 279)

Level of Functional Dependence / Dementia (Including Physical Disability): III

Case 7 (Age: 62; Sport: Boxing)
Age started: 16 years
Age retired: Not specified, likely 36 years
Length of career: 20 years
Age of symptom onset: Not mentioned
Delay between retirement and symptom onset (Age of symptom onset – Age retired): Not mentioned

Core Clinical Features

- Cognitive Impairment: 1
    - "He **wandered, out, half-dressed, at night and would importune for money**. He had **'glassy-looking eyes'**; he was **doubly incontinent at times**. He spoke normally and had no tremor**; he could not be left alone**. **When 59 he was admitted to a psychiatric hospital and found to be grossly demented**." (pg. 280)
    - "The **dementia deepened and in the last two years of his life** 'he aged, became like an old man'; he developed bronchopneumonia and died at the age of 62." (pg. 280)
- Executive Function Impairment: 1
    - "He **wandered, out, half-dressed, at night and would importune for money**. He had **'glassy-looking eyes'**; he was **doubly incontinent at times**. He spoke normally and had no tremor**; he could not be left alone**. **When 59 he was admitted to a psychiatric hospital and found to be grossly demented**." (pg. 280)
    - "The **dementia deepened and in the last two years of his life** 'he aged, became like an old man'; he developed bronchopneumonia and died at the age of 62." (pg. 280)
- Neurobehavioral Dysregulation: 1
    - "He **wandered, out, half-dressed, at night and would importune for money**. He had **'glassy-looking eyes'**; he was **doubly incontinent at times**. He spoke normally and had no tremor**; he could not be left alone**. **When 59 he was admitted to a psychiatric hospital and found to be grossly demented**." (pg. 280)
    - "He retired from the ring after a particularly damaging fight, and started to teach physical training and boxing in boarding schools. He gave this up after a few years at a time when he was **drinking heavily and beginning to become moody and violent**." (pg. 280)
    - "He had **spells of 'going within himself' followed by aggressive attacks on his wife and his home**. He complained of **violent headaches**; his sexual demands gave his wife little rest." (pg. 280)
- Progressive Course: Progressive

Supportive Features

- Depression: NM
- Anxiety: NM
- Apathy: NM
- Paranoia: NM
- Suicidality: NM
- Motor Signs (Parkinsonism / ALS): 1
    - "**When about 54 he began to be 'very unsteady on his feet' and kept falling about**." (pg. 280)
    - "He spoke normally and had no tremor… The summary stated that '**examination of the CNS, including gait, revealed no abnormality**." (pg. 280)
- Delayed Onset of Symptoms and Problems: NM

Level of Functional Dependence / Dementia: IV

- "He retired from the ring after a particularly damaging fight, and started to teach physical training and boxing in boarding schools. He gave this up after a few years at a time when he was **drinking heavily and beginning to become moody and violent**. A few years later he lost a job in a printing works for hiding away to sleep and he seems not to have worked again." (pg. 280)
- "He **wandered, out, half-dressed, at night and would importune for money**. He had **'glassy-looking eyes'**; he was **doubly incontinent at times**. He spoke normally and had no tremor**; he could not be left alone**. **When 59 he was admitted to a psychiatric hospital and found to be grossly demented**." (pg. 280)
- "The **dementia deepened and in the last two years of his life** 'he aged, became like an old man'; he developed bronchopneumonia and died at the age of 62." (pg. 280)

Level of Functional Dependence / Dementia (Including Physical Disability): IV

Case 8 (Age: 71; Sport: Boxing)
Age started: 17 years
Age retired: 40 years
Length of career: 23 years
Age of symptom onset: 60 years
Delay between retirement and symptom onset (Age of symptom onset – Age retired): 20 years

Core Clinical Features

- Cognitive Impairment: 1
    - "He **began to go downhill when he was 60 years old**… The **last attack affected his right side**, and his **intellect and behavior then** deteriorated… He burnt all his newspaper cuttings and then asked for them back. He would **try to go unclothed into the street**. He died at home aged 71." (pg. 281)
- Executive Function Impairment: NM
- Neurobehavioral Dysregulation: 1
    - "He **became violent towards his wife** but would afterwards apologize. He burnt all his newspaper cuttings and then asked for them back." (pg. 281)
- Progressive Course: Progressive

Supportive Features

- Depression: NM
- Anxiety: NM
- Apathy: NM
- Paranoia: NM
- Suicidality: NM
- Motor Signs (Parkinsonism / ALS): 1
    - "He **began to go downhill when he was 60 years old**. **He then lost consciousness for two hours and on recovery he was ataxic and had some weakness of his left side**. **Several similar episodes occurred during the next 10 years**; **between them he remained ataxic with a tendency to fall**. He **had a tremor** and could not tie a bow. The **last attack affected his right side**…" (pg. 281)
- Delayed Onset of Symptoms and Problems: 1
    - "He retired at about the age of 40 years… He began to go downhill when he was 60 years old." (pg. 281)

Level of Functional Dependence / Dementia: IV

- "He retired at about the age of 40 years. After this he worked until he was 68 as a labourer, although at some time he was registered as disabled." (pg. 281)
- "He **began to go downhill when he was 60 years old**. **He then lost consciousness for two hours and on recovery he was ataxic and had some weakness of his left side**. **Several similar episodes occurred during the next 10 years**; **between them he remained ataxic with a tendency to fall**. He **had a tremor** and could not tie a bow. The **last attack affected his right side**, and his **intellect and behavior then deteriorated.** He **became violent towards his wife** but would afterwards apologize. He burnt all his newspaper cuttings and then asked for them back. He would **try to go unclothed into the street**. He died at home aged 71." (pg. 281)

Level of Functional Dependence / Dementia (Including Physical Disability): IV

Case 9 (Age: 72; Sport: Boxing)
Age started: Not mentioned
Age retired: Not mentioned
Length of career: Not mentioned
Age of symptom onset: 31 years
Delay between retirement and symptom onset (Age of symptom onset – Age retired): Not mentioned

Core Clinical Features

- Cognitive Impairment: 1
  - **“**The blood pressure ranged around 210/130, and **he was considered to be demented possibly as a result of cerebral trauma**.” (pg. 282)
- Executive Function Impairment: NM
- Neurobehavioral Dysregulation: NM
- Progressive Course: Progressive
  - “This man was a patient in a psychiatric hospital for the last seven years of his life. His younger brother was interviewed. As a young man the patient had taught physical training. He boxed in the Army and had later been ‘quite a successful’ professional boxer… On his return to England, **when aged 31, his brother noticed that there was a tremor of his left hand and that his speech was slurred and hoarse.** The hoarseness had followed a blow to the throat. He **started drinking heavily and drifted from job to job**. He **tried to kill himself when aged 65** and was **admitted to a psychiatric hospital**.” (pg. 282)

Supportive Features

- Depression: NM
- Anxiety: NM
- Apathy: NM
- Paranoia: NM
- Suicidality: 1
  - “He **started drinking heavily and drifted from job to job**. He **tried to kill himself when aged 65** and was **admitted to a psychiatric hospital**.” (pg. 282)
- Motor Signs (Parkinsonism / ALS): 1
  - “On his return to England, **when aged 31, his brother noticed that there was a tremor of his left hand and that his speech was slurred and hoarse.** The hoarseness had followed a blow to the throat.” (pg. 282)
  - “**A Parkinsonian facies** was noted, **with tremor and rigidity of the left upper limb**. The **left vocal chords were markedly thickened and he was dysarthric**. The blood pressure ranged around 210/130, and **he was considered to be demented possibly as a result of cerebral trauma**. A **transient left-sided hemiparesis was noticed nine months before his death** from bronchopneumonia at the age of 72 years.” (pg. 282)
- Delayed Onset of Symptoms and Problems: NM

Level of Functional Dependence / Dementia: IV

- “This man was a patient in a **psychiatric hospital for the last seven years of his life**.” (pg. 282)
- “He **started drinking heavily and drifted from job to job**.” (pg. 282)

Level of Functional Dependence / Dementia (Including Physical Disability): IV

Case 10 (Age: 67; Sport: Boxing)
Age started: Not mentioned
Age retired: Not mentioned
Length of career: Not mentioned
Age of symptom onset: about 40 years
Delay between retirement and symptom onset (Age of symptom onset – Age retired): Not mentioned

Core Clinical Features

- Cognitive Impairment: 1
  - "[His second wife] **first noticed that his memory was poor when, aged about 40**, he was serving in her shop." (pg. 283)
  - "At the **age of 60 he developed a left hemiparesis and was admitted to a mental hospital three years later**. He was **disorientated** and had **a marked loss of recent memory**. He was **paranoid and deluded, and became confused and aggressive**." (pg. 283)
- Executive Function Impairment: NM
- Neurobehavioral Dysregulation: 1
  - "He and his wife had **drinking bouts** and he was **known in his locality as a violent man**, although 'he had been a good husband'. At the **age of 60 he developed a left hemiparesis and was admitted to a mental hospital three years later**. He was **disorientated** and had **a marked loss of recent memory**. He was **paranoid and deluded, and became confused and aggressive**." (pg. 283)
- Progressive Course: Progressive

Supportive Features

- Depression: NM
- Anxiety: NM
- Apathy: NM
- Paranoia: 1
  - "He was **paranoid and deluded, and became confused and aggressive**." (pg. 283)
- Suicidality: NM
- Motor Signs (Parkinsonism / ALS): 1
  - At the **age of 60 he developed a left hemiparesis and was admitted to a mental hospital three years later**…"(pg. 283)
- Delayed Onset of Symptoms and Problems: NM

Level of Functional Dependence / Dementia: V

- "She first noticed that his memory was poor when, aged about 40, he was serving in her shop. Later he worked as a labourer, and became unemployed for five years after the loss of an eye (unrelated to boxing). He returned to work as a road sweeper." (pg. 283)
- "The **diagnosis was of cerebral arteriosclerosis and organic dementia, probably post-traumatic in origin**. The term **'punch-drunk'** was used. He **gradually deteriorated over the next four years** and died aged 67." (pg. 283)

Level of Functional Dependence / Dementia (Including Physical Disability): V

Case 11 (Age: 67; Sport: Boxing)
Age started: Not mentioned
Age retired: about 35 years
Length of career: Not mentioned
Age of symptom onset: 67 years
Delay between retirement and symptom onset (Age of symptom onset – Age retired): 32 years

Core Clinical Features

- Cognitive Impairment: 1
    - "**When aged 67 he was admitted to hospital in heart failure** with a **history of increasing confusion**. He was **disorientated and aggressive**...." (pg. 284)
- Executive Function Impairment: NM
- Neurobehavioral Dysregulation: 1
    - "He was **disorientated and aggressive**…" (pg. 284)
- Progressive Course: Unable to Classify
    - "During the last six years of his life **he remained bright and alert and worked regularly** as a road sweeper. **When aged 67 he was admitted to hospital in heart failure** with a **history of increasing confusion**. He was **disorientated and aggressive**; **Argyll Robertson pupils were present**." (pg. 284)
    - "He died three months later, aged 67." (pg. 284)

Supportive Features

- Depression: NM
- Anxiety: NM
- Apathy: NM
- Paranoia: NM
- Suicidality: NM
- Motor Signs (Parkinsonism / ALS): NM
- Delayed Onset of Symptoms and Problems: 1

Level of Functional Dependence / Dementia: IV

- "He had boxed professionally until about 35 years old and had then been in the Army during the war. His wife believed that he was discharged from the Army, and later from the maritime service, on health grounds. He took small jobs interspersed with National Assistance for many years." (pg. 284)
- "During the last six years of his life **he remained bright and alert and worked regularly** as a road sweeper. **When aged 67 he was admitted to hospital in heart failure** with a **history of increasing confusion**. He was **disorientated and aggressive**; **Argyll Robertson pupils were present**." (pg. 284)
- "He died three months later, aged 67." (pg. 284)

Level of Functional Dependence / Dementia (Including Physical Disability): IV

Case 12 (Age: 91; Sport: Boxing)
Age started: Not mentioned
Age retired: Not mentioned
Length of career: Not mentioned
Age of symptom onset: Not mentioned
Delay between retirement and symptom onset (Age of symptom onset – Age retired): Not mentioned

Core Clinical Features

- Cognitive Impairment: 0
  - "He **spent his last years in a home for the partially disabled as he had become almost blind**, **for reasons that were never stated**. He was **solitary and placid** but **occasionally became aggressive**. **He was considered active and mentally alert up to the time of his death, at 91**, from cardiac ischaemia." (pg. 284)
  - "It was noted at the post-mortem examination that there were no stigmata of his former profession and that he was a **'remarkably well-preserved old man.'**" (pg. 284)
- Executive Function Impairment: NM
- Neurobehavioral Dysregulation: 1
  - "He was **solitary and placid** but **occasionally became aggressive**. **He was considered active and mentally alert up to the time of his death, at 91**, from cardiac ischaemia." (pg. 284)
- Progressive Course: Non-Progressive

Supportive Features

- Depression: NM
- Anxiety: NM
- Apathy: NM
- Paranoia: NM
- Suicidality: NM
- Motor Signs (Parkinsonism / ALS): NM
- Delayed Onset of Symptoms and Problems: NM

Level of Functional Dependence / Dementia: II

- **"**He **spent his last years in a home for the partially disabled as he had become almost blind**, **for reasons that were never stated**… **He was considered active and mentally alert up to the time of his death, at 91**, from cardiac ischaemia." (pg. 284)
- "It was noted at the post-mortem examination that there were no stigmata of his former profession and that he was a **'remarkably well-preserved old man.'**" (pg. 284)

Level of Functional Dependence / Dementia (Including Physical Disability): II

Case 13 (Age: 57; Sport: Boxing)
Age started: 14 years
Age retired: Not mentioned
Length of career: Not mentioned
Age of symptom onset: Not mentioned
Delay between retirement and symptom onset (Age of symptom onset – Age retired): Not mentioned

Core Clinical Features

- Cognitive Impairment: 1
  - "After his discharge a few years later [after rejoining the Navy when aged 43] he **became 'more vague' and 'spoke more slowly'**. He **began to neglect his appearance** and he **complained that he could not grip things or get on a bus**. His **sight and his memory seemed a little faulty**." (pg. 285)
  - "He **wandered away bemused and had to be taken to a mental hospital. He was then 53**. On admission he was **mildly confused and complained of blackouts**. His wife, from whom he was separated, but who was still concerned for him, said that **his personality had been changing for the worse.** A brief **neurological report confirmed this and mentioned a deterioration of habits**. He **could not recall his address or the date**… **During the next year his memory deteriorated further, he was completely disorientated and needed help with dressing**. These features were emphasized in a detailed neurological investigation… He **could not undress himself**." (pg. 285)
  - "…it was concluded that **'the cortical atrophy and dementia may be related to his previous experiences as a boxer.'**" (pg. 285)
- Executive Function Impairment: 1
  - "He **wandered away bemused and had to be taken to a mental hospital. He was then 53**. On admission he was **mildly confused and complained of blackouts**. His wife, from whom he was separated, but who was still concerned for him, said that **his personality had been changing for the worse.** A brief **neurological report confirmed this and mentioned a deterioration of habits**. He **could not recall his address or the date**. No incoordination was found but a **'little dyspraxia'** was queried. **During the next year his memory deteriorated further, he was completely disorientated and needed help with dressing**. These features were emphasized in a detailed neurological investigation. In addition his **speech was slurred, and like his movements was slow and tremulous**. He **could not undress himself**." (pg. 285)
- Neurobehavioral Dysregulation: 1
  - "He had never drunk alcohol excessively but **he now became violent after two pints of beer, sometimes 'fighting with his wife**.'" (pg. 285)
  - "His wife, from whom he was separated, but who was still concerned for him, said that **his personality had been changing for the worse.** A brief **neurological report confirmed this and mentioned a deterioration of habits**." (pg. 285)
- Progressive Course: Progressive

Supportive Features

- Depression: NM
- Anxiety: NM
- Apathy: NM
- Paranoia: 1
  - "He became **incontinent, paranoid, and aggressive**…" (pg. 285)
- Suicidality: NM
- Motor Signs (Parkinsonism / ALS): 2
  - "No incoordination was found but a **'little dyspraxia'** was queried. **During the next year his memory deteriorated further, he was completely disorientated and needed help with dressing**. These features were emphasized in a detailed neurological investigation. In addition his **speech was slurred, and like his movements was slow and tremulous**. He **could not undress himself**." (pg. 285)
  - "The **deterioration continued**. He became **incontinent, paranoid, and aggressive**. He ate off the table with his fingers' a cigarette packet he called a flower; he undid buttons when asked to put his tongue out. He continued to have blackouts and a few days after a convulsion he died from bronchopneumonia, aged 57 years." (pg. 285)
- Delayed Onset of Symptoms and Problems: NM

Level of Functional Dependence / Dementia: IV

- "After leaving the Navy he worked for several years in the police, followed by a period in the docks. His daughter first remembered him some years later when he was a labourer employed by the local council." (pg. 285)
- "He rejoined the Navy when aged 43 and trained cadets during the war." (pg. 285)

- “He worked as a milk roundsman, a bus conductor, and later as a machine operator. When he could no longer manage this, the firm transferred him to labouring and caretaking. After two years he was given notice without warning.” (pg. 285)
- “…it was concluded that **‘the cortical atrophy and dementia may be related to his previous experiences as a boxer.’**” (pg. 285)
- “The **deterioration continued**. He became **incontinent, paranoid, and aggressive**. He ate off the table with his fingers’ a cigarette packet he called a flower; he undid buttons when asked to put his tongue out. He continued to have blackouts and a few days after a convulsion he died from bronchopneumonia, aged 57 years.” (pg. 285)

Level of Functional Dependence / Dementia (Including Physical Disability): IV

Case 14 (Age: 61; Sport: Boxing)
Age started: Not specified, sometime after joining the Royal Air Force at age 18
Age retired: Not mentioned
Length of career: Not mentioned
Age of symptom onset: Not mentioned
Delay between retirement and symptom onset (Age of symptom onset – Age retired): Not mentioned

Core Clinical Features

- Cognitive Impairment: 0
    - "He was a placid, popular man, happily married with five children. He drank and smoked little. He suffered no injuries during his boxing career and had no serious illnesses. His wife said that his **eyesight and hearing**, his **speech and his memory were normal up to the time of his death**, from a cerebral haemorrhage, at the age of 61." (pg. 286)
- Executive Function Impairment: NM
- Neurobehavioral Dysregulation: NM
- Progressive Course: Non-Progressive

Supportive Features

- Depression: NM
- Anxiety: NM
- Apathy: NM
- Paranoia: NM
- Suicidality: NM
- Motor Signs (Parkinsonism / ALS): 0
    - "He was a placid, popular man, happily married with five children. He drank and smoked little. He suffered no injuries during his boxing career and had no serious illnesses. His wife said that his **eyesight and hearing**, his **speech and his memory were normal up to the time of his death**, from a cerebral haemorrhage, at the age of 61." (pg. 286)
- Delayed Onset of Symptoms and Problems: NM

Level of Functional Dependence / Dementia: II
Level of Functional Dependence / Dementia (Including Physical Disability): II

Case 15 (Age: 58; Sport: Boxing)
Age started: Not mentioned
Age retired: Not mentioned
Length of career: Not mentioned
Age of symptom onset: Not mentioned
Delay between retirement and symptom onset (Age of symptom onset – Age retired): Not mentioned

Core Clinical Features
- Cognitive Impairment: NM
- Executive Function Impairment: NM
- Neurobehavioral Dysregulation: NM
- Progressive Course: Non-Progressive

Supportive Features
- Depression: NM
- Anxiety: NM
- Apathy: NM
- Paranoia: NM
- Suicidality: NM
- Motor Signs (Parkinsonism / ALS): 0
    - "**No neurological or psychological abnormality was noted at any time**. He neither drank nor smoked. His work record was satisfactory until he dies from a fractured skull and cerebral haemorrhage after a road traffic accident at the age of 58." (pg. 287)
- Delayed Onset of Symptoms and Problems: NM

Level of Functional Dependence / Dementia: II
- "**No neurological or psychological abnormality was noted at any time**. He neither drank nor smoked. His work record was satisfactory until he dies from a fractured skull and cerebral haemorrhage after a road traffic accident at the age of 58." (pg. 287)

Level of Functional Dependence / Dementia (Including Physical Disability): II

Harvey & Newsom Davis (1974)

Case 1 (Age: 25; Sport: Boxing)
Age started: 14 years (professional at 21)
Age retired: Not specified, likely 24 years ("three and a half years later" (pg. 928) from age 21)
Length of career: Not specified, likely about 10 years
Age of symptom onset: Not specified, but after 16 professional bouts
Delay between retirement and symptom onset (Age of symptom onset – Age retired): Symptom onset during career (after 16 bouts in a 25-bout professional career)

Core Clinical Features
- Cognitive Impairment: 1
    - "He was **cooperative but mentally slow**. He had a verbal I.Q. of 95 and a performance I.Q. of 88." (pg. 928)
- Executive Function Impairment: NM
- Neurobehavioral Dysregulation: 1
    - "He **developed lability of mood, becoming more aggressive and socially unstable**." (pg. 928)
    - "After discharge he had **another episode of disturbed behavior and had to be readmitted** for a period to a psychiatric hospital." (pg. 928)
- Progressive Course: Progressive

Supportive Features
- Depression: 1
    - "He **developed lability of mood, becoming more aggressive and socially unstable**. An **acute depressive illness with paranoid features then necessitated his admission to a psychiatric unit**, where for a few days he received a small dose of chlorpromazine followed by trifluoperazine 5 mg. twice daily for about two weeks. It was recognized that **he also had a neurological disorder,** and he was therefore later transferred to the national Hospital at which time he had not taken phenothiazines for four weeks." (pg. 928)
- Anxiety: NM
- Apathy: NM
- Paranoia: 1
    - "He **developed lability of mood, becoming more aggressive and socially unstable**. An **acute depressive illness with paranoid features then necessitated his admission to a psychiatric unit**, where for a few days he received a small dose of chlorpromazine followed by trifluoperazine 5 mg. twice daily for about two weeks. It was recognized that **he also had a neurological disorder,** and he was therefore later transferred to the national Hospital at which time he had not taken phenothiazines for four weeks." (pg. 928)
- Suicidality: NM
- Motor Signs (Parkinsonism / ALS): 1
    - "His family first noticed that this **speech was slurred** after he had had 16 professional bouts. **Six months later he started to shuffle slightly when walking, his legs appearing stiff**. Despite his success in his last few fights, **he was aware of his slowness on his feet** and of increasing difficulty in avoiding trouble in the ring." (pg. 928)
    - "He retired from the ring a year before his admission to hospital. During that time, **his gait and speech deteriorated."** (pg. 928)
    - "He had a **symmetrical extrapyramidal disorder** characterized by an **expressionless face, generalized poverty of movement, cogwheel rigidity in all four limbs, and typically extrapyramidal speech and gait**. His **tendon reflexes were brisk**, with **left extensor plantar and variable right plantar response**. There was no sensory deficit, and sphincter function was normal." (pg. 928)
- Delayed Onset of Symptoms and Problems: 0
    - "In our patient **the onset of symptoms was at a relatively early age** and at the time in his career when most of his boxing experience had been as an amateur." (pg. 928)

Level of Functional Dependence / Dementia: IV
- "The development in the young boxer of **extrapyramidal and pyramidal dysfunction, coupled with depressive and paranoid mood swings,** leaves us in no doubt that he had the traumatic encephalopathy of boxers. This clinical diagnosis is supported by the radiological demonstration of a **cavum septum pellucidum** which is a characteristic feature of this syndrome." (pg. 928)

Level of Functional Dependence / Dementia (Including Physical Disability): IV

Kaste et al. (1982)

Note. The authors report the neurological findings, psychological test findings, electroencephalogram (EEG) and brainstem auditory evoked potential (BEP) findings, and computed tomography (CT) findings of 14 boxers (8 amateurs and 6 professionals) with a mean age of 31 years, one of whom was still actively competing. Although they summarize the findings in Table 2 (pg. 1187), they only provide descriptions for Cases 1 and 2. The descriptions of these two cases are limited and presented together, but they are included below. The neurological status and psychological test performance of all other cases were normal (Table 2, pg. 1187).

Case 1 (Age: 53; Sport: Boxing)
Age started: Not mentioned
Age retired: Not mentioned
Length of career: Not mentioned
Age of symptom onset: Not mentioned
Delay between retirement and symptom onset (Age of symptom onset – Age retired): Not mentioned

Core Clinical Features

- Cognitive Impairment: 1
  - "Two professionals (nos 1 and 2), however, showed **obvious deviations from the normal [psychological] test performance**. They both had subjective symptoms and objective signs of brain injury (Table 2)." (pg. 1187)
- Executive Function Impairment: 1
  - "One of the two performed below the normal average in all the tests, and he had very poor scores on the digit symbol, trail-making, and Benton visual retention test, which require rapid and accurate perception, learning, and memory. The other had **poor scores in the associative learning and in the Wisconsin card-sorting tests; these results suggest slow and inflexible learning.**" (pg. 1187)
- Neurobehavioral Dysregulation: 2
  - "He was one of two professionals (nos 1 and 2) who also had had episodes of **embarrassing inappropriate behaviour** which were attributed to their boxing careers." (pg. 1186)
- Progressive Course: Unable to Classify

Supportive Features

- Depression: NM
- Anxiety: NM
- Apathy: NM
- Paranoia: NM
- Suicidality: NM
- Suicidality: NM
- Motor Signs (Parkinsonism / ALS): 1
  - "Only one professional (no. 1) had **abnormal neurological findings: apraxia and slight unsteadiness and slight slowness and uncertainty in mental functions were observed**." (pg. 1186)
- Delayed Onset of Symptoms and Problems: NM

Level of Functional Dependence / Dementia: III

- "Although only one of the boxers, the oldest, had **dysfunctions that affected his normal daily living and social relations**, another, the second oldest [Case 2], had already had episodes of **inappropriate behaviour** which were attributed to his boxing career." (pg. 1187)

Level of Functional Dependence / Dementia (Including Physical Disability): III

Case 2 (Age: Not specified, between 19-53; Sport: Boxing)
Age started: Not mentioned
Age retired: Not mentioned
Length of career: Not mentioned
Age of symptom onset: Not mentioned
Delay between retirement and symptom onset (Age of symptom onset – Age retired): Not mentioned

Core Clinical Features

- Cognitive Impairment: 1
    - "Two professionals (nos 1 and 2), however, showed **obvious deviations from the normal [psychological] test performance**. They both had subjective symptoms and objective signs of brain injury (Table 2)." (pg. 1187)
- Executive Function Impairment: 1
    - "One of the two performed below the normal average in all the tests, and he had very poor scores on the digit symbol, trail-making, and Benton visual retention test, which require rapid and accurate perception, learning, and memory. The other had **poor scores in the associative learning and in the Wisconsin card-sorting tests; these results suggest slow and inflexible learning.**" (pg. 1187)
- Neurobehavioral Dysregulation: 2
    - "He was one of two professionals (nos 1 and 2) who also had had episodes of **embarrassing inappropriate behaviour** which were attributed to their boxing careers." (pg. 1186)
- Progressive Course: Unable to Classify

Supportive Features

- Depression: NM
- Anxiety: NM
- Apathy: NM
- Paranoia: NM
- Suicidality: NM
- Motor Signs (Parkinsonism / ALS): 0
    - "The neurological status of the other boxers, including subject 2, was within **normal limits** (table II)." (pg. 1186)
- Delayed Onset of Symptoms and Problems: NM

Level of Functional Dependence / Dementia: III

- "Although only one of the boxers, the oldest, had **dysfunctions that affected his normal daily living and social relations**, another, the second oldest [Case 2], had already had episodes of **inappropriate behaviour** which were attributed to his boxing career." (pg. 1187)

Level of Functional Dependence / Dementia (Including Physical Disability): III

Cases 3-14

Core Clinical Features

- Cognitive Impairment: 0
    - "Two professionals (nos 1 and 2), however, showed **obvious deviations from the normal [psychological] test performance**. They both had subjective symptoms and objective signs of brain injury (Table 2)." (pg. 1187)
    - "In the remainder of the tests the average scores of our boxers did not differ from the normal average level." (pg. 1186)
- Executive Function Impairment: 0
    - "Two professionals (nos 1 and 2), however, showed **obvious deviations from the normal [psychological] test performance**. They both had subjective symptoms and objective signs of brain injury (Table 2)." (pg. 1187)
- Neurobehavioral Dysregulation: 0
    - "Boxers were also asked about symptoms connected with fights, especially amnesia and post-concussion symptoms, and about possible sequelae of their boxing careers, such as clumsiness of speech or movements, loss of memory, **changes in personality**, or other subjective symptoms." (pg. 1186)
    - Table II lists "Normal" neurological status, psychological tests, and other symptoms for boxers 3-14 (pg. 1187)
- Progressive Course: Non-Progressive

Supportive Features

- Depression: NM
- Anxiety: NM
- Apathy: NM
- Paranoia: NM
- Suicidality: NM
- Motor Signs (Parkinsonism / ALS): 0
    - "The neurological status of the other boxers, including subject 2, was within normal limits (table II)." (pg. 1186)
- Delayed Onset of Symptoms and Problems: 0

Level of Functional Dependence / Dementia: I

- "Although only one of the boxers, the oldest, had **dysfunctions that affected his normal daily living and social relations**, another, the second oldest [Case 2], had already had episodes of **inappropriate behaviour** which were attributed to his boxing career." (pg. 1187)

Level of Functional Dependence / Dementia (Including Physical Disability): I

Casson et al. (1984)

Note. The authors present the findings from the neurological examinations, EEGs, head CT scans, and neuropsychological testing of 18 former and active boxers (13 former, 5 active). The boxers had not retired from boxing for medical, neurological, or psychiatric reasons and had no known history of neurological, psychiatric, or serious mental illness, or known history of drug or alcohol abuse. None had any history of neurological, psychiatric, or serious medical illness, or a history of drug or alcohol abuse. All were employed full time. The authors only report individual clinical features in Table 2 (2664-2665). They refer to them in the context of the group in text. Although they do not provide any individual case reports or descriptions of clinical features, the relevant clinical features specific to each case, as reported/written in Table 2, are presented below. The Impairment Index was calculated by the authors as a ratio of abnormal neuropsychological test scores over the number of tests administered.

Case 1 (Age: 37; Sport: Boxing)
Age started: Not mentioned
Age retired: Not mentioned, but at least one year before the present study (inclusion criteria for sample)
Length of career: 12 years
Age of symptom onset: Not mentioned
Delay between retirement and symptom onset (Age of symptom onset – Age retired): Not mentioned

Core Clinical Features
- Cognitive Impairment: 1
  - Neurological Examination: **Impaired recent memory**
  - Digit Symbol Test: Normal
  - Wechsler Memory Test
    - Verbal immediate: Abnormal
    - Verbal delayed: Abnormal
    - Visual immediate: Abnormal
    - Visual delayed: Abnormal
  - Bender Gestalt Test
    - Standard administration: Normal
    - 5-s recall administration: Abnormal
  - Impairment Index: 0.50
- Executive Function Impairment: 0
  - Trail Making Test: Normal
- Neurobehavioral Dysregulation: 0
- Progressive Course: Unable to Classify

Supportive Features
- Depression: 0
- Anxiety: 0
- Apathy: 0
- Paranoia: 0
- Suicidality: 0
- Motor Signs (Parkinsonism / ALS): 0
- Delayed Onset of Symptoms and Problems: NM

Level of Functional Dependence / Dementia: II
Level of Functional Dependence / Dementia (Including Physical Disability): II

Case 2 (Age: 42; Sport: Boxing)
Age started: Not mentioned
Age retired: Not mentioned, but at least one year before the present study (inclusion criteria for sample)
Length of career: 10 years
Age of symptom onset: Not mentioned
Delay between retirement and symptom onset (Age of symptom onset – Age retired): Not mentioned

Core Clinical Features

- Cognitive Impairment: 1
  - Neurological Examination: **Mild OMS** (Organic Mental Syndrome – disorientation, confusion, and memory loss)
  - Digit Symbol Test: Abnormal
  - Wechsler Memory Test
    - Verbal immediate: Abnormal
    - Verbal delayed: Abnormal
    - Visual immediate: Abnormal
    - Visual delayed: Abnormal
  - Bender Gestalt Test
    - Standard administration: Abnormal
    - 5-s recall administration: Abnormal
  - Impairment Index: 1.00
- Executive Function Impairment: 1
  - Trail Making Test: Abnormal
- Neurobehavioral Dysregulation: 0
- Progressive Course: Unable to Classify

Supportive Features

- Depression: 0
- Anxiety: 0
- Apathy: 0
- Paranoia: 0
- Suicidality: 0
- Motor Signs (Parkinsonism / ALS): 0
- Delayed Onset of Symptoms and Problems: NM

Level of Functional Dependence / Dementia: II
Level of Functional Dependence / Dementia (Including Physical Disability): II

Case 3 (Age: 33; Sport: Boxing)
Age started: Not mentioned
Age retired: Not mentioned, but at least one year before the present study (inclusion criteria for sample)
Length of career: 13 years
Age of symptom onset: Not mentioned
Delay between retirement and symptom onset (Age of symptom onset – Age retired): Not mentioned

Core Clinical Features

- Cognitive Impairment: 1
  - Digit Symbol Test: Normal
  - Wechsler Memory Test
    - Verbal immediate: Abnormal
    - Verbal delayed: Abnormal
    - Visual immediate: Normal
    - Visual delayed: Normal
  - Bender Gestalt Test
    - Standard administration: Normal
    - 5-s recall administration: Abnormal
  - Impairment Index: 0.38
- Executive Function Impairment: 0
  - Trail Making Test: Normal
- Neurobehavioral Dysregulation: 0
- Progressive Course: Unable to Classify

Supportive Features

- Depression: 0
- Anxiety: 0
- Apathy: 0
- Paranoia: 0
- Suicidality: 0
- Motor Signs (Parkinsonism / ALS): 0
  - Neurological Examination: Normal
- Delayed Onset of Symptoms and Problems: NM

Level of Functional Dependence / Dementia: I
Level of Functional Dependence / Dementia (Including Physical Disability): I

Case 4 (Age: 37; Sport: Boxing)
Age started: Not mentioned
Age retired: Not mentioned, but at least one year before the present study (inclusion criteria for sample)
Length of career: 10 years
Age of symptom onset: Not mentioned
Delay between retirement and symptom onset (Age of symptom onset – Age retired): Not mentioned

Core Clinical Features
- Cognitive Impairment: 1
    - Digit Symbol Test: Abnormal
    - Wechsler Memory Test
        - Verbal immediate: Abnormal
        - Verbal delayed: Abnormal
        - Visual immediate: Abnormal
        - Visual delayed: Abnormal
    - Bender Gestalt Test
        - Standard administration: Abnormal
        - 5-s recall administration: Abnormal
    - Impairment Index: 0.88
- Executive Function Impairment: 0
    - Trail Making Test: Normal
- Neurobehavioral Dysregulation: 0
- Progressive Course: Unable to Classify

Supportive Features
- Depression: 0
- Anxiety: 0
- Apathy: 0
- Paranoia: 0
- Suicidality: 0
- Motor Signs (Parkinsonism / ALS): 0
    - Neurological Examination: Normal
- Delayed Onset of Symptoms and Problems: NM

Level of Functional Dependence / Dementia: II
Level of Functional Dependence / Dementia (Including Physical Disability): II

Case 5 (Age: 39; Sport: Boxing)
Age started: Not mentioned
Age retired: Not mentioned, but at least one year before the present study (inclusion criteria for sample)
Length of career: 14 years
Age of symptom onset: Not mentioned
Delay between retirement and symptom onset (Age of symptom onset – Age retired): Not mentioned

Core Clinical Features
- Cognitive Impairment: 1
    - Digit Symbol Test: Abnormal
    - Wechsler Memory Test
        - Verbal immediate: Abnormal
        - Verbal delayed: Abnormal
        - Visual immediate: Abnormal
        - Visual delayed: Abnormal
    - Bender Gestalt Test
        - Standard administration: Normal
        - 5-s recall administration: Abnormal
    - Impairment Index: 0.88
- Executive Function Impairment: 1
    - Trail Making Test: Abnormal
- Neurobehavioral Dysregulation: 0
- Progressive Course: Unable to Classify

Supportive Features
- Depression: 0
- Anxiety: 0
- Apathy: 0
- Paranoia: 0
- Suicidality: 0
- Motor Signs (Parkinsonism / ALS): 0
    - Neurological Examination: Normal
- Delayed Onset of Symptoms and Problems: NM

Level of Functional Dependence / Dementia: II
Level of Functional Dependence / Dementia (Including Physical Disability): II

Case 6 (Age: 58; Sport: Boxing)
Age started: Not mentioned
Age retired: Not mentioned, but at least one year before the present study (inclusion criteria for sample)
Length of career: 8 years
Age of symptom onset: Not mentioned
Delay between retirement and symptom onset (Age of symptom onset – Age retired): Not mentioned

Core Clinical Features

- Cognitive Impairment: 1
  - Digit Symbol Test: Normal
  - Wechsler Memory Test
    - Verbal immediate: Abnormal
    - Verbal delayed: Abnormal
    - Visual immediate: Abnormal
    - Visual delayed: Abnormal
  - Bender Gestalt Test
    - Standard administration: Normal
    - 5-s recall administration: Abnormal
  - Impairment Index: 0.75
- Executive Function Impairment: 0
  - Trail Making Test: Normal
- Neurobehavioral Dysregulation: 0
- Progressive Course: Unable to Classify

Supportive Features

- Depression: 0
- Anxiety: 0
- Apathy: 0
- Paranoia: 0
- Suicidality: 0
- Motor Signs (Parkinsonism / ALS): 0
  - Neurological Examination: Normal (**cortical release phenomena**)
- Delayed Onset of Symptoms and Problems: NM

Level of Functional Dependence / Dementia: II
Level of Functional Dependence / Dementia (Including Physical Disability): II

Case 7 (Age: 46; Sport: Boxing)
Age started: Not mentioned
Age retired: Not mentioned, but at least one year before the present study (inclusion criteria for sample)
Length of career: 22 years
Age of symptom onset: Not mentioned
Delay between retirement and symptom onset (Age of symptom onset – Age retired): Not mentioned

Core Clinical Features

- Cognitive Impairment: 1
    - Digit Symbol Test: Normal
    - Wechsler Memory Test
        - Verbal immediate: Abnormal
        - Verbal delayed: Abnormal
        - Visual immediate: Abnormal
        - Visual delayed: Abnormal
    - Bender Gestalt Test
        - Standard administration: Abnormal
        - 5-s recall administration: Abnormal
    - Impairment Index: 0.88
- Executive Function Impairment: 1
    - Trail Making Test: Abnormal
- Neurobehavioral Dysregulation: 0
- Progressive Course: Unable to Classify

Supportive Features

- Depression: 0
- Anxiety: 0
- Apathy: 0
- Paranoia: 0
- Suicidality: 0
- Motor Signs (Parkinsonism / ALS): 1
    - Neurological Examination: **Dysarthria nystagmus**
- Delayed Onset of Symptoms and Problems: NM

Level of Functional Dependence / Dementia: II
Level of Functional Dependence / Dementia (Including Physical Disability): II

Case 8 (Age: 30; Sport: Boxing)
Age started: Not mentioned
Age retired: Not mentioned, but at least one year before the present study (inclusion criteria for sample)
Length of career: 15 years
Age of symptom onset: Not mentioned
Delay between retirement and symptom onset (Age of symptom onset – Age retired): Not mentioned

Core Clinical Features

- Cognitive Impairment: 1
    - Digit Symbol Test: Abnormal
    - Wechsler Memory Test
        - Verbal immediate: Abnormal
        - Verbal delayed: Abnormal
        - Visual immediate: Abnormal
        - Visual delayed: Abnormal
    - Bender Gestalt Test
        - Standard administration: Normal
        - 5-s recall administration: Abnormal
    - Impairment Index: 0.88
- Executive Function Impairment: 1
    - Trail Making Test: Abnormal
- Neurobehavioral Dysregulation: 0
- Progressive Course: Unable to Classify

Supportive Features

- Depression: 0
- Anxiety: 0
- Apathy: 0
- Paranoia: 0
- Suicidality: 0
- Motor Signs (Parkinsonism / ALS): 0
    - Neurological Examination: Normal
- Delayed Onset of Symptoms and Problems: NM

Level of Functional Dependence / Dementia: II
Level of Functional Dependence / Dementia (Including Physical Disability): II

Case 9 (Age: 37; Sport: Boxing)
Age started: Not mentioned
Age retired: Not mentioned, but at least one year before the present study (inclusion criteria for sample)
Length of career: 5 years
Age of symptom onset: Not mentioned
Delay between retirement and symptom onset (Age of symptom onset – Age retired): Not mentioned

Core Clinical Features

- Cognitive Impairment: 1
    - Digit Symbol Test: Normal
    - Wechsler Memory Test
        - Verbal immediate: Abnormal
        - Verbal delayed: Abnormal
        - Visual immediate: Normal
        - Visual delayed: Normal
    - Bender Gestalt Test
        - Standard administration: Abnormal
        - 5-s recall administration: Abnormal
    - Impairment Index: 0.62
- Executive Function Impairment: 1
    - Trail Making Test: Abnormal
- Neurobehavioral Dysregulation: 0
- Progressive Course: Unable to Classify

Supportive Features

- Depression: 0
- Anxiety: 0
- Apathy: 0
- Paranoia: 0
- Suicidality: 0
- Motor Signs (Parkinsonism / ALS): 0
    - Neurological Examination: Normal
- Delayed Onset of Symptoms and Problems: NM

Level of Functional Dependence / Dementia: II
Level of Functional Dependence / Dementia (Including Physical Disability): II

Case 10 (Age: 55; Sport: Boxing)
Age started: Not mentioned
Age retired: Not mentioned, but at least one year before the present study (inclusion criteria for sample)
Length of career: 5 years
Age of symptom onset: Not mentioned
Delay between retirement and symptom onset (Age of symptom onset – Age retired): Not mentioned

Core Clinical Features

- Cognitive Impairment: 1
    - Digit Symbol Test: Abnormal
    - Wechsler Memory Test
        - Verbal immediate: Abnormal
        - Verbal delayed: Abnormal
        - Visual immediate: Abnormal
        - Visual delayed: Abnormal
    - Bender Gestalt Test
        - Standard administration: Abnormal
        - 5-s recall administration: Abnormal
    - Impairment Index: 1.00
    - Neurological Examination: **Mild OMS** (Organic Mental Syndrome – disorientation, confusion, and memory loss)
- Executive Function Impairment: 1
    - Trail Making Test: Abnormal
- Neurobehavioral Dysregulation: 0
- Progressive Course: Unable to Classify

Supportive Features

- Depression: 0
- Anxiety: 0
- Apathy: 0
- Paranoia: 0
- Suicidality: 0
- Motor Signs (Parkinsonism / ALS): 1
    - Neurological Examination: **Mild OMS, right Babinski**
- Delayed Onset of Symptoms and Problems: NM

Level of Functional Dependence / Dementia: II
Level of Functional Dependence / Dementia (Including Physical Disability): II

Case 11 (Age: 40; Sport: Boxing)
Age started: Not mentioned
Age retired: Not mentioned, but at least one year before the present study (inclusion criteria for sample)
Length of career: 20 years
Age of symptom onset: Not mentioned
Delay between retirement and symptom onset (Age of symptom onset – Age retired): Not mentioned

Core Clinical Features

- Cognitive Impairment: 1
    - Digit Symbol Test: Normal
    - Wechsler Memory Test
        - Verbal immediate: Not administered
        - Verbal delayed: Not administered
        - Visual immediate: Not administered
        - Visual delayed: Not administered
    - Bender Gestalt Test
        - Standard administration: Normal
        - 5-s recall administration: Abnormal
    - Impairment Index: 0.50
- Executive Function Impairment: 1
    - Trail Making Test: Abnormal
- Neurobehavioral Dysregulation: 0
- Progressive Course: Unable to Classify

Supportive Features

- Depression: 0
- Anxiety: 0
- Apathy: 0
- Paranoia: 0
- Suicidality: 0
- Motor Signs (Parkinsonism / ALS): 0
    - Neurological Examination: Normal
- Delayed Onset of Symptoms and Problems: NM

Level of Functional Dependence / Dementia: II
Level of Functional Dependence / Dementia (Including Physical Disability): II

Case 12 (Age: 60; Sport: Boxing)
Age started: Not mentioned
Age retired: Not mentioned, but at least one year before the present study (inclusion criteria for sample)
Length of career: 15 years
Age of symptom onset: Not mentioned
Delay between retirement and symptom onset (Age of symptom onset – Age retired): Not mentioned

Core Clinical Features
- Cognitive Impairment: 1
    - Digit Symbol Test: Abnormal
    - Wechsler Memory Test
        - Verbal immediate: Not administered
        - Verbal delayed: Not administered
        - Visual immediate: Not administered
        - Visual delayed: Not administered
    - Bender Gestalt Test
        - Standard administration: Abnormal
        - 5-s recall administration: Abnormal
    - Impairment Index: 1.00
    - Neurological Examination: **Mild OMS** (Organic Mental Syndrome – disorientation, confusion, and memory loss)
- Executive Function Impairment: 1
    - Trail Making Test: Abnormal
- Neurobehavioral Dysregulation: 0
- Progressive Course: Unable to Classify

Supportive Features
- Depression: 0
- Anxiety: 0
- Apathy: 0
- Paranoia: 0
- Suicidality: 0
- Motor Signs (Parkinsonism / ALS): 0
- Delayed Onset of Symptoms and Problems: NM

Level of Functional Dependence / Dementia: II
Level of Functional Dependence / Dementia (Including Physical Disability): II

Case 13 (Age: 27; Sport: Boxing)
Age started: Not mentioned
Age retired: Not mentioned, but at least one year before the present study (inclusion criteria for sample)
Length of career: 6 years
Age of symptom onset: Not mentioned
Delay between retirement and symptom onset (Age of symptom onset – Age retired): Not mentioned

Core Clinical Features

- Cognitive Impairment: 1
    - Digit Symbol Test: Abnormal
    - Wechsler Memory Test
        - Verbal immediate: Abnormal
        - Verbal delayed: Abnormal
        - Visual immediate: Abnormal
        - Visual delayed: Abnormal
    - Bender Gestalt Test
        - Standard administration: Abnormal
        - 5-s recall administration: Abnormal
    - Impairment Index: 1.00
- Executive Function Impairment: 1
    - Trail Making Test: Abnormal
- Neurobehavioral Dysregulation: 0
- Progressive Course: Unable to Classify

Supportive Features

- Depression: 0
- Anxiety: 0
- Apathy: 0
- Paranoia: 0
- Suicidality: 0
- Motor Signs (Parkinsonism / ALS): 0
    - Neurological Examination: Normal
- Delayed Onset of Symptoms and Problems: NM

Level of Functional Dependence / Dementia: II
Level of Functional Dependence / Dementia (Including Physical Disability): II

Case 14 (Age: 24; Sport: Boxing)
Age started: Not mentioned
Age retired: Still active
Length of career: 8 years
Age of symptom onset: Not mentioned
Delay between retirement and symptom onset (Age of symptom onset – Age retired): Not mentioned

Core Clinical Features

- Cognitive Impairment: 1
    - Wechsler Memory Test
        - Verbal immediate: Abnormal
        - Verbal delayed: Abnormal
        - Visual immediate: Abnormal
        - Visual delayed: Abnormal
    - Bender Gestalt Test
        - Standard administration: Abnormal
        - 5-s recall administration: Abnormal
    - Impairment Index: 0.88
- Executive Function Impairment: 1
    - Trail Making Test: Abnormal
- Neurobehavioral Dysregulation: 0
- Progressive Course: Unable to Classify

Supportive Features

- Depression: 0
- Anxiety: 0
- Apathy: 0
- Paranoia: 0
- Suicidality: 0
- Motor Signs (Parkinsonism / ALS): 0
    - Neurological Examination: Normal
- Delayed Onset of Symptoms and Problems: 0 (still active)

Level of Functional Dependence / Dementia: II
Level of Functional Dependence / Dementia (Including Physical Disability): II

Case 15 (Age: 25; Sport: Boxing)
Age started: Not mentioned
Age retired: Still active
Length of career: 6 years
Age of symptom onset: Not mentioned
Delay between retirement and symptom onset (Age of symptom onset – Age retired): Not mentioned

Core Clinical Features

- Cognitive Impairment: 1
    - Digit Symbol Test: Normal
    - Wechsler Memory Test
        - Verbal immediate: Abnormal
        - Verbal delayed: Abnormal
        - Visual immediate: Abnormal
        - Visual delayed: Abnormal
    - Bender Gestalt Test
        - Standard administration: Normal
        - 5-s recall administration: Abnormal
    - Impairment Index: 0.62
- Executive Function Impairment: 0
    - Trail Making Test: Normal
- Neurobehavioral Dysregulation: 0
- Progressive Course: Unable to Classify

Supportive Features

- Depression: 0
- Anxiety: 0
- Apathy: 0
- Paranoia: 0
- Suicidality: 0
- Motor Signs (Parkinsonism / ALS): 0
    - Neurological Examination: Normal
- Delayed Onset of Symptoms and Problems: 0 (still active)

Level of Functional Dependence / Dementia: II
Level of Functional Dependence / Dementia (Including Physical Disability): II

Case 16 (Age: 23; Sport: Boxing)
Age started: Not mentioned
Age retired: Still active
Length of career: 4 years
Age of symptom onset: Not mentioned
Delay between retirement and symptom onset (Age of symptom onset – Age retired): Not mentioned

Core Clinical Features

- Cognitive Impairment: 0
    - Digit Symbol Test: Not administered
    - Wechsler Memory Test
        - Verbal immediate: Abnormal
        - Verbal delayed: Abnormal
        - Visual immediate: Normal
        - Visual delayed: Normal
    - Bender Gestalt Test
        - Standard administration: Normal
        - 5-s recall administration: Normal
    - Impairment Index: 0.33
- Executive Function Impairment: 2
    - Trail Making Test: Not administered
- Neurobehavioral Dysregulation: 0
- Progressive Course: Unable to Classify

Supportive Features

- Depression: 0
- Anxiety: 0
- Apathy: 0
- Paranoia: 0
- Suicidality: 0
- Motor Signs (Parkinsonism / ALS): 0
    - Neurological Examination: Normal
- Delayed Onset of Symptoms and Problems: 0 (still active)

Level of Functional Dependence / Dementia: I
Level of Functional Dependence / Dementia (Including Physical Disability): I

Case 17 (Age: 18; Sport: Boxing)
Age started: Not mentioned
Age retired: Still active
Length of career: ¼ year
Age of symptom onset: Not mentioned
Delay between retirement and symptom onset (Age of symptom onset – Age retired): Not mentioned

Core Clinical Features

- Cognitive Impairment: 0
    - Digit Symbol Test: Not administered
    - Wechsler Memory Test
        - Verbal immediate: Abnormal
        - Verbal delayed: Abnormal
        - Visual immediate: Normal
        - Visual delayed: Normal
    - Bender Gestalt Test
        - Standard administration: Normal
        - 5-s recall administration: Normal
    - Impairment Index: 0.33
- Executive Function Impairment: 2
    - Trail Making Test: Not administered
- Neurobehavioral Dysregulation: 0
- Progressive Course: Unable to Classify

Supportive Features

- Depression: 0
- Anxiety: 0
- Apathy: 0
- Paranoia: 0
- Suicidality: 0
- Motor Signs (Parkinsonism / ALS): 0
    - Neurological Examination: Normal
- Delayed Onset of Symptoms and Problems: 0 (still active)

Level of Functional Dependence / Dementia: I
Level of Functional Dependence / Dementia (Including Physical Disability): I

Case 18 (Age: 18; Sport: Boxing)
Age started: Not mentioned
Age retired: Still active
Length of career: 1 year
Age of symptom onset: Not mentioned
Delay between retirement and symptom onset (Age of symptom onset – Age retired): Not mentioned

Core Clinical Features

- Cognitive Impairment: 0
  - Wechsler Memory Test
    - Verbal immediate: Abnormal
    - Verbal delayed: Abnormal
    - Visual immediate: Normal
    - Visual delayed: Normal
  - Bender Gestalt Test
    - Standard administration: Normal
    - 5-s recall administration: Normal
  - Impairment Index: 0.33
- Executive Function Impairment: 2
  - Trail Making Test: Not administered
- Neurobehavioral Dysregulation: 0
- Progressive Course: Unable to Classify

Supportive Features

- Depression: 0
- Anxiety: 0
- Apathy: 0
- Paranoia: 0
- Suicidality: 0
- Motor Signs (Parkinsonism / ALS): 0
  - Neurological Examination: Normal
- Delayed Onset of Symptoms and Problems: 0 (still active)

Level of Functional Dependence / Dementia: I
Level of Functional Dependence / Dementia (Including Physical Disability): I

Roberts, Whitwell, Acland, & Bruton (1990)

Note: The authors appear to assume that “punch drunk syndrome” (dementia pugilistica) is progressive because they state that it is “a condition characterized by damage to the pyramidal, extrapyramidal, and cerebellar systems and by progression from affective disturbance and memory loss, through psychosis, to progressive dementia.” However, the clinical description of this case is so limited, and there is not enough evidence to clearly classify this specific case as progressive or non-progressive.

Case 1 (Age: 76; Gender: Female; Physical Abuse Victim)
Age of first exposure: Not mentioned
Age of last exposure: Not mentioned
Duration of physical abuse: Not specified, but husband had been violent for many years (see below)
Age of symptom onset: Not mentioned
Delay between last exposure and symptom onset (Age of symptom onset – Age retired): Not mentioned

Core Clinical Features

- Cognitive Impairment: 1
  - “She had a history of a stroke and **had become demented over the past few years, this manifesting predominantly as memory loss and mental confusion**.” (pg. 918)
  - “The appearance of the brain of this repeatedly battered woman resembled that seen in dementia pugilistica.” (pg. 919)
  - “Our case helps to fill the gap in the evidence for the concept that head injury can be followed by **Alzheimer-type degeneration**.” (pg. 919)
- Executive Function Impairment: NM
- Neurobehavioral Dysregulation: NM
- Progressive Course: Unable to Classify

Supportive Features

- Depression: NM
- Anxiety: NM
- Apathy: NM
- Paranoia: NM
- Suicidality: NM
- Motor Signs (Parkinsonism / ALS): 2
  - “A 76-year-old woman was admitted to hospital unconscious after being found at home with multiple injuries. She had rib fractures, multiple bruises and abrasions to the head, and **signs of left-sided weakness**. She had a history of a stroke and **had become demented over the past few years, this manifesting predominantly as memory loss and mental confusion**. Relatives told us that **her husband had been violent towards her for many years**, particularly in relation to his drinking, and the patient had often been seen with cuts and bruises.” (pg. 918)
- Delayed Onset of Symptoms and Problems: NA

Level of Functional Dependence / Dementia: IV
Level of Functional Dependence / Dementia (Including Physical Disability): IV

Hof, Knabe, Bovier, & Bouras (1991)

Note: This article is focused on neuropathology with no mention of the clinical features of traumatic encephalopathy. We included descriptions of the clinical features of this case. However, they all seem to be due to the patient's severe autism with self-injury behavior (SIB). The authors provide evidence that the neurofibrillary tangles (NFTs) in her brain had a similar distribution to the pattern observed in cases of dementia pugilistica (pg. 324).

Case 1 (Age: 24; Sex: Female; Autism diagnosis with head banging and self-injury behavior)
Age of first head banging behavior: 7 years
Age of last head banging behavior: Not mentioned
Duration of head banging: Not mentioned
Age of symptom onset: Not mentioned
Delay between last head banging behavior and symptom onset (Age of symptom onset – Age retired): Not mentioned

Core Clinical Features

- Cognitive Impairment: 2
  - "A psychological evaluation showed a 6-month mental retardation. At this time, the first symptoms of infantile autism were observed…" (pg. 321)
  - "She progressively lost her ability to walk and stopped using the few words she had learned." (pg. 322)
- Executive Function Impairment: NM
- Neurobehavioral Dysregulation: NM
- Progressive Course: Progressive
  - "Moreover, chronic SIB may cause long-term brain lesions which could be related to the **progressive** deterioration observed in our patient." (pg. 325)

Supportive Features

- Depression: NM
- Anxiety: NM
- Apathy: NM
- Paranoia: NM
- Suicidality: NM
- Motor Signs (Parkinsonism / ALS): 1
  - "A neurological examination a few days before her death revealed no sign of cerebral or neuromuscular palsy." (pg. 322)
  - "She progressively lost her ability to walk and stopped using the few words she had learned." (pg. 322)
- Delayed Onset of Symptoms and Problems: NM

Level of Functional Dependence / Dementia: II

- "However, when she was 7 years old, she began to display a very serious form of SIB including head-hiting, head-banging, eye-gouging, and self-biting. She became blind as a bilateral detachment of the retina and dullness of the cornea resulted from repeated head and eye trauma. She was later hospitalized in the psychiatric clinic where she stayed until her death. The next 14 years in psychiatric care were characterized by continuous SIB, particularly directed at her head, which she knocked incessantly against the walls, bed sides or persons." (pg. 322)
- "She also used to pull out her hair and scream for hours. She was apparently completely insensitive to pain. She played with a few object and could not tolerate changes in her daily schedule and environment (whenever changes occurred, she would immediately display SIB and scream for hours)." (pg. 322)

Level of Functional Dependence / Dementia (Including Physical Disability): IV

Hof et al. (1992)

Note: The authors briefly present some of the clinical features of three cases (Case 1 was previously reported by Constantinidis & Tissot (1967)) of former professional boxers with dementia pugilistica. However, they present the clinical features as a group in the Materials and Methods section and say that all three boxers demonstrated these symptoms. We included this small description below. It is limited beyond merely listing symptoms. In other words, it does not give much insight into the development of these symptoms, their severity, and their impact relative to each other. Additionally, the introduction, results, and discussion sections are focused on neuropathology differences between dementia pugilistica and Alzheimer's disease, so there is little other information to use for coding.

Cases 1-3 (Case 1 Age: 58; Case 2 Age: 63; Case 3 Age: 69; Sport: Boxing)
Ages started: Not mentioned
Ages retired: Not mentioned
Length of careers: > 25 years for Cases 1-3
Ages of symptom onset: Not mentioned
Delays between retirement and symptom onset (Age of symptom onset – Age retired): Not mentioned

Core Clinical Features

- Cognitive Impairment: 1
  - "The clinical presentation of all three boxers was consistent with the diagnosis of dementia pugilistica and revealed **severe memory disturbances…"** (pg. 24)
- Executive Function Impairment: 1
  - "…**impairment of judgement** and **abstract thinking**, **temporospatial disorientation**…" (pg. 24)
- Neurobehavioral Dysregulation: NM
- Progressive Course: Unable to Classify

Supportive Features

- Depression: 1
  - "The clinical presentation of all three boxers was consistent with the diagnosis of dementia pugilistica and revealed **severe memory disturbances**, **impairment of judgement** and **abstract thinking**, **temporospatial disorientation**, and **dysphoria**…" (pg. 24)
- Anxiety: NM
- Apathy: NM
- Paranoia: NM
- Suicidality: NM
- Motor Signs (Parkinsonism / ALS): 1
  - "… combined with a **constellation of neurological symptoms** including to various degrees **extrapyramidal rigidity, resting tremor, gait disturbances, hypomimia and dysarthria.**" (pg. 24)
- Delayed Onset of Symptoms and Problems: NM

Level of Functional Dependence / Dementia: III
Level of Functional Dependence / Dementia (Including Physical Disability): IV

Jordan et al. (1995)

Case 1 (Age: 71, Sport: Boxing)
Age started: Not mentioned
Age retired: Not mentioned
Length of career: 11 years
Age of symptom onset: Not mentioned
Delay between retirement and symptom onset (Stated in article): 37 years

Core Clinical Features
- Cognitive Impairment: 1
  - "A **neurological syndrome** of **progressive cognitive decline began 37 years after his last bout (10 years antemortem)…"** (pg. 698)
- Executive Function Impairment: NM
- Neurobehavioral Dysregulation: NM
- Progressive Course: Progressive
  - "A **neurological syndrome** of **progressive cognitive decline began 37 years after his last bout (10 years antemortem)…"** (pg. 698)

Supportive Features
- Depression: NM
- Anxiety: NM
- Apathy: NM
- Paranoia: NM
- Suicidality: NM
- Motor Signs (Parkinsonism / ALS): 1
  - "… and **an acute right hemiplegia developed 8 years antemortem**, **following which he no longer recognized his family**. Six weeks antemortem, he suffered a fall and died following two successive left parietooccipital cerebral hemorrhages." (pg. 698)
- Delayed Onset of Symptoms and Problems: 1
  - "A **neurological syndrome** of **progressive cognitive decline began 37 years after his last bout (10 years antemortem)…"** (pg. 698)

Level of Functional Dependence / Dementia: IV
- A **neurological syndrome** of **progressive cognitive decline began 37 years after his last bout (10 years antemortem)** and **an acute right hemiplegia developed 8 years antemortem**, **following which he no longer recognized his family**. Six weeks antemortem, he suffered a fall and died following two successive left parietooccipital cerebral hemorrhages." (pg. 698)

Level of Functional Dependence / Dementia (Including Physical Disability): IV

Geddes et al. (1996)

Case 1 (Age: 23; Sport: Boxing)
Age started: 11 years (professional for 4 years)
Age retired: 23 years (death from acute subdural haemmorhage sustained in a boxing match)
Length of career: 12 years
Age of symptom onset: Not applicable
Delay between retirement and symptom onset (Age of symptom onset – Age retired): Not applicable

Core Clinical Features

- Cognitive Impairment: 2
  - "[He] has suffered only one knock-out, in his penultimate contest. He had not previously sustained a serious head injury as a result of a fight, had **no neurological symptoms**, although he was described as being **'somewhat forgetful.'** He had not undergone psychometric testing." (pg. 12)
- Executive Function Impairment: NM
- Neurobehavioral Dysregulation: NM
- Progressive Course: Unable to Classify

Supportive Features

- Depression: NM
- Anxiety: NM
- Apathy: NM
- Paranoia: NM
- Suicidality: NM
- Motor Signs (Parkinsonism / ALS): 0
  - "He had not previously sustained a serious head injury as a result of a fight, had **no neurological symptoms**…" (pg. 12)
- Delayed Onset of Symptoms and Problems: NA

Level of Functional Dependence / Dementia: II

- "During his last contest, however, our subject sustained a severe head injury, as a result he developed an acute subdural haemorrhage with marked swelling of the underlying hemisphere… he died 46 h after admission, despite evacuation of the haematoma." (pgs. 12-13)

Level of Functional Dependence / Dementia (Including Physical Disability): II

Williams & Tannenberg (1996)

Case 1 (Age: 33; Sport: Circus clown / dwarf throwing)
Age started: Not mentioned
Age retired: Not mentioned
Length of career: 15 years in the circus, 8-10 years in dwarf throwing
Age of symptom onset: Not mentioned
Delay between retirement and symptom onset (Age of symptom onset – Age retired): Not mentioned, but symptom onset occurred prior to retirement from dwarf throwing, as he was still participating at time of death.

Core Clinical Features

- Cognitive Impairment: 1
  - "The hospital notes recorded **aggression**, **poor concentration**, **impatience, panic symptoms** and **blackout**..." (pg. 102)
  - "Clinical examination had revealed **ataxia**, **nystagmus**, **mild Rombergism** and **brisk reflexes** and he had received treatment for Wernicke's encephalopathy. The **clinical problems were attributed to alcohol abuse and dietary indiscretions**. A diagnosis of dementia pugilistica had not been considered." (pg. 102)
- Executive Function Impairment: 1
  - "The hospital notes recorded **aggression**, **poor concentration**, **impatience, panic symptoms** and **blackout**…" (pg. 102)
- Neurobehavioral Dysregulation: 1
  - "The hospital notes recorded **aggression**, **poor concentration**, **impatience, panic symptoms** and **blackout**…" (pg. 102)
- Progressive Course: Unable to Classify

Supportive Features

- Depression: NM
- Anxiety: 1
  - "The hospital notes recorded **aggression**, **poor concentration**, **impatience, panic symptoms** and **blackout**…" (pg. 102)
- Apathy: NM
- Paranoia: NM
- Suicidality: NM
- Motor Signs (Parkinsonism / ALS): 1
  - "Clinical examination had revealed **ataxia**, **nystagmus**, **mild Rombergism** and **brisk reflexes** and he had received treatment for Wernicke's encephalopathy. The **clinical problems were attributed to alcohol abuse and dietary indiscretions**. A diagnosis of dementia pugilistica had not been considered." (pg. 102)
- Delayed Onset of Symptoms and Problems: NM

Level of Functional Dependence / Dementia: III

- "Four years prior to his death, he had been sacked for persistent drunkenness from his job as a circus clown. He had worked in the circus 15 years and had apparently been knocked unconscious "a dozen times" during circus routines, without receiving hospitalization." (pg. 102)

Level of Functional Dependence / Dementia (Including Physical Disability): III

Jordan et al. (1997)

Note. The authors do not provide individual narrative case reports in text. However, they do list clinical features of the 30 cases in Table 3 (pg. 138). Although limited relative to other narrative descriptions in this document, relevant individual clinical features of each case are presented below. The age at which the boxers started boxing, retired, and experienced symptom onset were not reported. Therefore, this information is not included at the beginning of each case. The authors did include each case's Mini-Mental State Examination (MMSE) score, which quantifies cognitive functioning; their Chronic Brain Injury (CBI) scale, which quantifies clinical findings of motor, cognitive, and psychiatric deficits; and the authors' chronic traumatic brain injury (CTBI) classification in Table 3. These variables are provided at the beginning of each case instead. On the MMSE, the authors considered a score of 28-30 indicates "normal" cognitive functioning, a score of 20-27 indicates "mild" impairment, a score of 10-19 indicates "moderate" impairment, and a score of ≤9 indicates "severe" impairment. The CBI ranges from 0-9. The authors considered a score of 0 is classified as "normal", a score of 1-2 indicates "mild impairment", a score of 3-4 indicates "moderate impairment", and a score of >4 indicates "severe impairment." (pg. 137)

Case 1 (Age: 70; Sport: Boxing)
Core Clinical Features
- Cognitive Impairment: 1
    - MMSE Score: 3
    - "Neurologic Examination: **Severe dementia**; **severe spasticity**; **agitation**; **dysarthria**; **gait ataxia**" (pg. 138)
- Executive Function Impairment: 1
    - "Neurologic Examination: **Severe dementia**; **severe spasticity**; **agitation**; **dysarthria**; **gait ataxia**" (pg. 138)
- Neurobehavioral Dysregulation: 1
    - "Neurologic Examination: **Severe dementia**; **severe spasticity**; **agitation**; **dysarthria**; **gait ataxia**" (pg. 138)
- Progressive Course: Progressive

Supportive Features
- Depression: 0
- Anxiety: 0
- Apathy: 0
- Paranoia: NM
- Suicidality: NM
- Motor Signs (Parkinsonism / ALS): 1
    - "Neurologic Examination: **Severe dementia**; **severe spasticity**; **agitation**; **dysarthria**; **gait ataxia**" (pg. 138)
- Delayed Onset of Symptoms and Problems: 1

Level of Functional Dependence / Dementia: IV
- CBI Score: 8
- CTBI Classification: Probable

Level of Functional Dependence / Dementia (Including Physical Disability): IV

Case 2 (Age: 25; Sport: Boxing)
Core Clinical Features
- Cognitive Impairment: 0
    - MMSE Score: 28
- Executive Function Impairment: 0
- Neurobehavioral Dysregulation: 0
- Progressive Course: Non-Progressive

Supportive Features
- Depression: 0
- Anxiety: 0
- Apathy: 0
- Paranoia: NM
- Suicidality: NM
- Motor Signs (Parkinsonism / ALS): 0
    - "Neurologic Examination: Normal" (pg. 138)
- Delayed Onset of Symptoms and Problems: 0

Level of Functional Dependence / Dementia: I
- CBI Score: 0
- CTBI Classification: Normal
- "Neurologic Examination: Normal" (pg. 138)
- "Comment: Admitted to use of recreational drugs" (pg. 138)

Level of Functional Dependence / Dementia (Including Physical Disability): I

Case 3 (Age: 48; Sport: Boxing)
Core Clinical Features

- Cognitive Impairment: 2
  - MMSE Score: 28
  - "Neurologic Examination: **Posturing on stressed gait; decreased simple and complex attention; mild dysarthria**" (pg. 138)
- Executive Function Impairment: 1
  - "Neurologic Examination: **Posturing on stressed gait; decreased simple and complex attention; mild dysarthria**" (pg. 138)
- Neurobehavioral Dysregulation: 0
- Progressive Course: Unable to Classify

Supportive Features

- Depression: 0
- Anxiety: 0
- Apathy: 0
- Paranoia: NM
- Suicidality: NM
- Motor Signs (Parkinsonism / ALS): 1
  - "Neurologic Examination: **Posturing on stressed gait; decreased simple and complex attention; mild dysarthria**" (pg. 138)
- Delayed Onset of Symptoms and Problems: NM

Level of Functional Dependence / Dementia: II

- CBI Score: 2
- CTBI Classification: Probable

Level of Functional Dependence / Dementia (Including Physical Disability): II

Case 4 (Age: 29; Sport: Boxing)
Core Clinical Features
- Cognitive Impairment: 0
  - MMSE Score: 29
- Executive Function Impairment: 0
- Neurobehavioral Dysregulation: 0
- Progressive Course: Non-Progressive

Supportive Features
- Depression: 0
- Anxiety: 0
- Apathy: 0
- Paranoia: NM
- Suicidality: NM
- Motor Signs (Parkinsonism / ALS): 0
  - "Neurologic Examination: Normal" (pg. 138)
- Delayed Onset of Symptoms and Problems: 0

Level of Functional Dependence / Dementia: I
- CBI Score: 0
- CTBI Classification: Normal
- "Neurologic Examination: Normal" (pg. 138)

Level of Functional Dependence / Dementia (Including Physical Disability): I

Case 5 (Age: 31; Sport: Boxing)
Core Clinical Features
- Cognitive Impairment: 2
    - MMSE Score: N/A
- Executive Function Impairment: 2
- Neurobehavioral Dysregulation: 0
- Progressive Course: Non-Progressive

Supportive Features
- Depression: 0
- Anxiety: 0
- Apathy:0
- Paranoia: NM
- Suicidality: NM
- Motor Signs (Parkinsonism / ALS): 1
    - "Neurologic Examination: **Difficulty with Tandem gait**" (pg. 138)
    - "Comment: Complained of **difficulties with balance**" (pg. 138)
- Delayed Onset of Symptoms and Problems: 0

Level of Functional Dependence / Dementia: II
- CBI Score: 1
- CTBI Classification: Probable

Level of Functional Dependence / Dementia (Including Physical Disability): II

Case 6 (Age: 29; Sport: Boxing)
Core Clinical Features
- Cognitive Impairment: 0
    - MMSE Score: 30
- Executive Function Impairment: 0
- Neurobehavioral Dysregulation: 0
- Progressive Course: Non-Progressive

Supportive Features
- Depression: 0
- Anxiety: 0
- Apathy: 0
- Paranoia: NM
- Suicidality: NM
- Motor Signs (Parkinsonism / ALS): 0
    - “Neurologic Examination: Normal” (pg. 138)
- Delayed Onset of Symptoms and Problems: 0

Level of Functional Dependence / Dementia: I
- CBI Score: 0
- CTBI Classification: Normal
- “Neurologic Examination: Normal” (pg. 138)

Level of Functional Dependence / Dementia (Including Physical Disability): I

Case 7 (Age: 57; Sport: Boxing)
Core Clinical Features
- Cognitive Impairment: 0
  - MMSE Score: 29
- Executive Function Impairment: 0
- Neurobehavioral Dysregulation: 0
- Progressive Course: Non-Progressive

Supportive Features
- Depression: 0
- Anxiety: 0
- Apathy: 0
- Paranoia: NM
- Suicidality: NM
- Motor Signs (Parkinsonism / ALS): 0
  - "Neurologic Examination: Normal" (pg. 138)
- Delayed Onset of Symptoms and Problems: 0

Level of Functional Dependence / Dementia: I
- CBI Score: 0
- CTBI Classification: Normal
- "Neurologic Examination: Normal" (pg. 138)

Level of Functional Dependence / Dementia (Including Physical Disability): I

Case 8 (Age: 57; Sport: Boxing)
Core Clinical Features
- Cognitive Impairment: 0
  - MMSE Score: 28
- Executive Function Impairment: 0
- Neurobehavioral Dysregulation: 0
- Progressive Course: Non-Progressive

Supportive Features
- Depression: 0
- Anxiety: 0
- Apathy: 0
- Paranoia: NM
- Suicidality: NM
- Motor Signs (Parkinsonism / ALS): 0
  - "Neurologic Examination: Normal" (pg. 138)
- Delayed Onset of Symptoms and Problems: 0

Level of Functional Dependence / Dementia: I
- CBI Score: 0
- CTBI Classification: Normal
- "Neurologic Examination: Normal" (pg. 138)

Level of Functional Dependence / Dementia (Including Physical Disability): I

Case 9 (Age: 36; Sport: Boxing)
Core Clinical Features
- Cognitive Impairment: 0
    - MMSE Score: 30
- Executive Function Impairment: 0
- Neurobehavioral Dysregulation: 0
- Progressive Course: Non-Progressive

Supportive Features
- Depression: 0
- Anxiety: 0
- Apathy: 0
- Paranoia: NM
- Suicidality: NM
- Motor Signs (Parkinsonism / ALS): 0
    - "Neurologic Examination: Normal" (pg. 138)
- Delayed Onset of Symptoms and Problems: 0

Level of Functional Dependence / Dementia: I
- CBI Score: 0
- CTBI Classification: Normal
- "Neurologic Examination: Normal" (pg. 138)

Level of Functional Dependence / Dementia (Including Physical Disability): I

Case 10 (Age: 59; Sport: Boxing)
Core Clinical Features
- Cognitive Impairment: 1
  - MMSE Score: 27
- Executive Function Impairment: NM
- Neurobehavioral Dysregulation: 0
- Progressive Course: Non-Progressive

Supportive Features
- Depression:0
- Anxiety: 0
- Apathy: 0
- Paranoia: NM
- Suicidality: NM
- Motor Signs (Parkinsonism / ALS): 2
  - "Neurologic Examination: **Mild dysarthria**" (pg. 138)
- Delayed Onset of Symptoms and Problems: NM

Level of Functional Dependence / Dementia: II
- CBI Score: 2
- CTBI Classification: Possible
- "Comment: Regular alcohol use" (pg. 138)

Level of Functional Dependence / Dementia (Including Physical Disability): II

Case 11 (Age: 39; Sport: Boxing)
Core Clinical Features
- Cognitive Impairment: 0
    - MMSE Score: 30
- Executive Function Impairment: 0
- Neurobehavioral Dysregulation: 0
- Progressive Course: Non-Progressive

Supportive Features
- Depression: 0
- Anxiety: 0
- Apathy: 0
- Paranoia: NM
- Suicidality: NM
- Motor Signs (Parkinsonism / ALS): 0
    - "Neurologic Examination: Normal" (pg. 138)
- Delayed Onset of Symptoms and Problems: 0

Level of Functional Dependence / Dementia: I
- CBI Score: 0
- CTBI Classification: Normal
- "Neurologic Examination: Normal" (pg. 138)
- "Comment: Occasional recreational drug use; regular alcohol use" (pg. 138)

Level of Functional Dependence / Dementia (Including Physical Disability): I

Case 12 (Age: 70; Sport: Boxing)
Core Clinical Features
- Cognitive Impairment: 0
  - MMSE Score: 27
- Executive Function Impairment: NM
- Neurobehavioral Dysregulation: 0
- Progressive Course: Non-Progressive

Supportive Features
- Depression: 0
- Anxiety: 0
- Apathy: 0
- Paranoia: NM
- Suicidality: NM
- Motor Signs (Parkinsonism / ALS): 0
  - "Neurologic Examination: Normal" (pg. 138)
- Delayed Onset of Symptoms and Problems: NM

Level of Functional Dependence / Dementia: I
- CBI Score: 1
- CTBI Classification: Possible
- "Neurologic Examination: Normal" (pg. 138)
- "Comment: History of meningitis as a child and history of schizophrenia that preceded boxing; regular alcohol use." (pg. 138)

Level of Functional Dependence / Dementia (Including Physical Disability): I

Case 13 (Age: 65; Sport: Boxing)
Core Clinical Features
- Cognitive Impairment: 1
    - MMSE Score: 25
- Executive Function Impairment: NM
- Neurobehavioral Dysregulation: 0
- Progressive Course: Non-Progressive

Supportive Features
- Depression: 0
- Anxiety: 0
- Apathy: 0
- Paranoia: NM
- Suicidality: NM
- Motor Signs (Parkinsonism / ALS): 1
    - "Neurologic Examination: **Difficulty with tandem gait**; **positive Romberg sign**" (pg. 138)
- Delayed Onset of Symptoms and Problems: NM

Level of Functional Dependence / Dementia: III
- CBI Score: 2
- CTBI Classification: Possible
- "Comment: History of alcohol abuse" (pg. 138)

Level of Functional Dependence / Dementia (Including Physical Disability): III

Case 14 (Age: 76; Sport: Boxing)
Core Clinical Features
- Cognitive Impairment: 1
    - MMSE Score: 24
    - "Neurologic Examination: **Mild-moderate memory impairment**" (pg. 138)
- Executive Function Impairment: NM
- Neurobehavioral Dysregulation: 0
- Progressive Course: Non-Progressive

Supportive Features
- Depression: 0
- Anxiety:0
- Apathy: 0
- Paranoia: NM
- Suicidality: NM
- Motor Signs (Parkinsonism / ALS): NM
- Delayed Onset of Symptoms and Problems: NM

Level of Functional Dependence / Dementia: III
- CBI Score: 2
- CTBI Classification: Possible
- "Neurologic Examination: **Mild-moderate memory impairment**" (pg. 138)
- "Comment: Regular alcohol use" (pg. 138)

Level of Functional Dependence / Dementia (Including Physical Disability): III

Case 15 (Age: 69; Sport: Boxing)

Core Clinical Features

- Cognitive Impairment: 1
  - MMSE Score: 22
- Executive Function Impairment: NM
- Neurobehavioral Dysregulation: 0
- Progressive Course: Non-Progressive

Supportive Features

- Depression: 0
- Anxiety: 0
- Apathy: 0
- Paranoia: NM
- Suicidality: NM
- Motor Signs (Parkinsonism / ALS): 1
  - "Neurologic Examination: **Severe dysarthria; slight increases tone in lower extremities; ataxic gait; slight dysmetria on left**" (pg. 138)
- Delayed Onset of Symptoms and Problems: NM

Level of Functional Dependence / Dementia: III

- CBI Score: 4
- CTBI Classification: Probable
- "Neurologic Examination: **Severe dysarthria; slight increases tone in lower extremities; ataxic gait; slight dysmetria on left.**" (pg. 138)

Level of Functional Dependence / Dementia (Including Physical Disability): III

Case 16 (Age: 67; Sport: Boxing)
Core Clinical Features
- Cognitive Impairment: 0
    - MMSE Score: 29
- Executive Function Impairment: 0
- Neurobehavioral Dysregulation: 0
- Progressive Course: Non-Progressive

Supportive Features
- Depression: 0
- Anxiety: 0
- Apathy: 0
- Paranoia: NM
- Suicidality: NM
- Motor Signs (Parkinsonism / ALS): 0
    - "Neurologic Examination: Normal" (pg. 138)
- Delayed Onset of Symptoms and Problems: 0

Level of Functional Dependence / Dementia: I
- CBI Score: 0
- CTBI Classification: Normal
- "Neurologic Examination: Normal" (pg. 138)

Level of Functional Dependence / Dementia (Including Physical Disability): I

Case 17 (Age: 64; Sport: Boxing)
Core Clinical Features
- Cognitive Impairment: 1
  - MMSE Score: 27
- Executive Function Impairment: NM
- Neurobehavioral Dysregulation: 0
- Progressive Course: Non-Progressive

Supportive Features
- Depression: 0
- Anxiety: 0
- Apathy: 0
- Paranoia: NM
- Suicidality: NM
- Motor Signs (Parkinsonism / ALS): 1
  - "Neurologic Examination: **Mild hyperreflexia on left; resting tremor** and **mild cogwheel rigidity**" (pg. 138)
- Delayed Onset of Symptoms and Problems: NM

Level of Functional Dependence / Dementia: II
- CBI Score: 2
- CTBI Classification: Possible

Level of Functional Dependence / Dementia (Including Physical Disability): II

Case 18 (Age: 37: Sport: Boxing)
Core Clinical Features

- Cognitive Impairment: 1
  - MMSE Score: 20
  - "Neurologic Examination: **Flat affect with pseudobulbar-type laughing outburst; dysarthria; mild paratonia of right upper extremity; ankle clonus; slowed rapid alternating movements; ataxic gait; memory impairment**" (pg. 138)
- Executive Function Impairment: 1
  - "Neurologic Examination: **Flat affect with pseudobulbar-type laughing outburst."** (pg. 138)
- Neurobehavioral Dysregulation: 0
- Progressive Course: Progressive

Supportive Features

- Depression: 0
- Anxiety: 0
- Apathy: 1
  - "Neurologic Examination: **Flat affect with pseudobulbar-type laughing outburst; dysarthria; mild paratonia of right upper extremity; ankle clonus; slowed rapid alternating movements; ataxic gait; memory impairment**" (pg. 138)
- Paranoia: NM
- Suicidality: NM
- Motor Signs (Parkinsonism / ALS): 1
  - "Neurologic Examination: **Flat affect with pseudobulbar-type laughing outburst; dysarthria; mild paratonia of right upper extremity; ankle clonus; slowed rapid alternating movements; ataxic gait; memory impairment**" (pg. 138)
- Delayed Onset of Symptoms and Problems: NM

Level of Functional Dependence / Dementia: III

- CBI Score: 5
- CTBI Classification: Probable

Level of Functional Dependence / Dementia (Including Physical Disability): III

Case 19 (Age: 41; Sport: Boxing)
Core Clinical Features

- Cognitive Impairment: 1
    - MMSE Score: 17
    - "Neurologic Examination: **Mild pseudobulbar affect; dysarthria; memory impairment; dysmetria; ataxic gait**" (pg. 138)
- Executive Function Impairment: 1
    - "Neurologic Examination: **Mild pseudobulbar affect; dysarthria; memory impairment; dysmetria; ataxic gait**" (pg. 138)
- Neurobehavioral Dysregulation: 0
- Progressive Course: Unable to Classify

Supportive Features

- Depression: 0
- Anxiety: 0
- Apathy: 0
- Paranoia: NM
- Suicidality: NM
- Motor Signs (Parkinsonism / ALS): 1
    - "Neurologic Examination: **Mild pseudobulbar affect; dysarthria; memory impairment; dysmetria; ataxic gait**" (pg. 138)
- Delayed Onset of Symptoms and Problems: NM

Level of Functional Dependence / Dementia: III

- CBI Score: 5
- CTBI Classification: Probable

Level of Functional Dependence / Dementia (Including Physical Disability): III

Case 20 (Age: 35; Sport: Boxing)
Core Clinical Features
- Cognitive Impairment: 0
    - MMSE Score: 29
- Executive Function Impairment: 0
- Neurobehavioral Dysregulation: 0
- Progressive Course: Non-Progressive

Supportive Features
- Depression: 0
- Anxiety: 0
- Apathy: 0
- Paranoia: NM
- Suicidality: NM
- Motor Signs (Parkinsonism / ALS): 0
    - "Neurologic Examination: Normal" (pg. 138)
- Delayed Onset of Symptoms and Problems: 0

Level of Functional Dependence / Dementia: I
- CBI Score: 0
- CTBI Classification: Normal
- "Neurologic Examination: Normal" (pg. 13)

Level of Functional Dependence / Dementia (Including Physical Disability): I

Case 21 (Age: 64; Sport: Boxing)
Core Clinical Features

- Cognitive Impairment: 0
    - MMSE Score: 30
- Executive Function Impairment: 0
- Neurobehavioral Dysregulation: 0
- Progressive Course: Unable to Classify

Supportive Features

- Depression: 0
- Anxiety: 0
- Apathy: 0
- Paranoia: NM
- Suicidality: NM
- Motor Signs (Parkinsonism / ALS): 1
    - "Neurologic Examination: **Moderate dysarthria and incoordination; slight increased tone in right lower extremity with wide based ataxic gait; hyperreflexia in right upper extremity**" (pg. 138)
- Delayed Onset of Symptoms and Problems: NM

Level of Functional Dependence / Dementia: II

- CBI Score: 2
- CTBI Classification: Probable

Level of Functional Dependence / Dementia (Including Physical Disability): II

Case 22 (Age: 60; Sport: Boxing)
Core Clinical Features
- Cognitive Impairment: 1
    - MMSE Score: 27
- Executive Function Impairment: NM
- Neurobehavioral Dysregulation: 0
- Progressive Course: Unable to Classify

Supportive Features
- Depression: 0
- Anxiety: 0
- Apathy: 0
- Paranoia: NM
- Suicidality: NM
- Motor Signs (Parkinsonism / ALS): 2
    - "Neurologic Examination: **Difficulty with sharpened Romberg sign**" (pg. 138)
- Delayed Onset of Symptoms and Problems: NM

Level of Functional Dependence / Dementia: II
- CBI Score: 2
- CTBI Classification: Probable

Level of Functional Dependence / Dementia (Including Physical Disability): II

Case 23 (Age: 68; Sport: Boxing)
Core Clinical Features
- Cognitive Impairment: 0
    - MMSE Score: 29
- Executive Function Impairment: 0
- Neurobehavioral Dysregulation: 0
- Progressive Course: Unable to Classify

Supportive Features
- Depression: 0
- Anxiety: 0
- Apathy: 0
- Paranoia: NM
- Suicidality: NM
- Motor Signs (Parkinsonism / ALS): 2
    - "Neurologic Examination: **Mild tremor with titubation**" (pg. 138)
- Delayed Onset of Symptoms and Problems: 0

Level of Functional Dependence / Dementia: I
- CBI Score: 1
- CTBI Classification: Possible

Level of Functional Dependence / Dementia (Including Physical Disability): I

Case 24 (Age: 47; Sport: Boxing)
Core Clinical Features
- Cognitive Impairment: 0
    - MMSE Score: 30
- Executive Function Impairment: 0
- Neurobehavioral Dysregulation: 0
- Progressive Course: Unable to Classify

Supportive Features
- Depression: 0
- Anxiety: 0
- Apathy: 0
- Paranoia: NM
- Suicidality: NM
- Motor Signs (Parkinsonism / ALS): 1
    - "Neurologic Examination: **Mild dysarthria** and **cogwheel rigidity**" (pg. 138)
- Delayed Onset of Symptoms and Problems: NM

Level of Functional Dependence / Dementia: II
- CBI Score: 1
- CTBI Classification: Probable
- "Neurologic Examination: **Mild dysarthria** and **cogwheel rigidity**" (pg. 138)

Level of Functional Dependence / Dementia (Including Physical Disability): II

Case 25 (Age: 31; Sport: Boxing)
Core Clinical Features
- Cognitive Impairment: 2
  - MMSE Score: 28
- Executive Function Impairment: NM
- Neurobehavioral Dysregulation: 0
- Progressive Course: Unable to Classify

Supportive Features
- Depression: 0
- Anxiety: 0
- Apathy: 0
- Paranoia: NM
- Suicidality: NM
- Motor Signs (Parkinsonism / ALS): 1
  - "Neurologic Examination: **Mild dysmetria** and **difficulty with Romberg sign**; **hypomania** and **increased talkativeness**" (pg. 138)
- Delayed Onset of Symptoms and Problems: NM

Level of Functional Dependence / Dementia: II
- CBI Score: 2
- CTBI Classification: Possible
- "Neurologic Examination: **Mild dysmetria** and **difficulty with Romberg sign**; **hypomania** and **increased talkativeness.**" (pg. 138)
- "Comment: One episode of head trauma outside of the ring with loss of consciousness; still boxing; history of drug abuse." (pg. 138)
- "One boxer with mild CTBI was diagnosed with bipolar disorder during his boxing career and displayed hypomania and increased talkativeness during the examination." (pg. 139)

Level of Functional Dependence / Dementia (Including Physical Disability): II

Case 26 (Age: 39; Sport: Boxing)

Core Clinical Features

- Cognitive Impairment: 0
  - MMSE Score: 29
- Executive Function Impairment: 1
  - "Neurologic Examination: Anisocoria; abducens palsy diplopia on lateral gaze; dysmetria; ataxia; pseudobulbar affect and **behavioral disinhibition.**" (pg. 138)
- Neurobehavioral Dysregulation: 1
  - "Neurologic Examination: Anisocoria; abducens palsy diplopia on lateral gaze; dysmetria; ataxia; pseudobulbar affect and **behavioral disinhibition.**" (pg. 138)
- Progressive Course: Unable to Classify

Supportive Features

- Depression: 0
- Anxiety: 0
- Apathy: NM
- Paranoia: NM
- Suicidality: NM
- Motor Signs (Parkinsonism / ALS): 1
  - "Neurologic Examination: Anisocoria; abducens palsy diplopia on lateral gaze; dysmetria; ataxia; pseudobulbar affect and **behavioral disinhibition**" (pg. 138)
- Delayed Onset of Symptoms and Problems: NM

Level of Functional Dependence / Dementia: II

- CBI Score: 3
- CTBI Classification: Probable
- "Neurologic Examination: Anisocoria; abducens palsy diplopia on lateral gaze; dysmetria; ataxia; pseudobulbar affect and **behavioral disinhibition.**" (pg. 138)
- "Comment: History of alcohol abuse; cavum septum on CT." (pg. 138)

Level of Functional Dependence / Dementia (Including Physical Disability): II

Case 27 (Age: 30; Sport: Boxing)
Core Clinical Features
- Cognitive Impairment: 0
    - MMSE Score: 29
- Executive Function Impairment: 0
- Neurobehavioral Dysregulation: 0
- Progressive Course: Non-Progressive

Supportive Features
- Depression: 0
- Anxiety: 0
- Apathy: 0
- Paranoia: NM
- Suicidality: NM
- Motor Signs (Parkinsonism / ALS): 0
    - "Neurologic Examination: Normal" (pg. 138)
- Delayed Onset of Symptoms and Problems: 0

Level of Functional Dependence / Dementia: I
- CBI Score: 0
- CTBI Classification: Normal
- "Neurologic Examination: Normal" (pg. 138)

Level of Functional Dependence / Dementia (Including Physical Disability): I

Case 28 (Age: 64; Sport: Boxing)
Core Clinical Features
- Cognitive Impairment: 1
  - MMSE Score: 25
- Executive Function Impairment: NM
- Neurobehavioral Dysregulation: 0
- Progressive Course: Unable to Classify

Supportive Features
- Depression: 0
- Anxiety: 0
- Apathy: 0
- Paranoia: NM
- Suicidality: NM
- Motor Signs (Parkinsonism / ALS): 1
  - "Neurologic Examination: **Mild dysarthria; gait ataxia; resting tremor.**" (pg. 138)
- Delayed Onset of Symptoms and Problems: NM

Level of Functional Dependence / Dementia: II
- CBI Score: 3
- CTBI Classification: Probable
- "Neurologic Examination: **Mild dysarthria; gait ataxia; resting tremor.**" (pg. 138)

Level of Functional Dependence / Dementia (Including Physical Disability): II

Case 29 (Age: 37; Sport: Boxing)
Core Clinical Features
- Cognitive Impairment: 1
    - MMSE Score: 27
    - "Comment: Complained of **memory loss**" (pg. 138)
- Executive Function Impairment: NM
- Neurobehavioral Dysregulation: 0
- Progressive Course: Unable to Classify

Supportive Features
- Depression: 1
    - "Neurologic Examination: **Slight increased tone in lower extremities; abnormal sharpened Romberg sign; mild rigidity; mild depression**" (pg. 138)
- Anxiety: 0
- Apathy: 0
- Paranoia: NM
- Suicidality: NM
- Motor Signs (Parkinsonism / ALS): 1
    - "Neurologic Examination: **Slight increased tone in lower extremities; abnormal sharpened Romberg sign; mild rigidity; mild depression**" (pg. 138)
- Delayed Onset of Symptoms and Problems: NM

Level of Functional Dependence / Dementia: II
- CBI Score: 3
- CTBI Classification: Probable
- "Neurologic Examination: **Slight increased tone in lower extremities; abnormal sharpened Romberg sign; mild rigidity; mild depression**" (pg. 138)

Level of Functional Dependence / Dementia (Including Physical Disability): II

Case 30 (Age: 23; Sport: Boxing)
Core Clinical Features
- Cognitive Impairment: 0
    - MMSE Score: 29
- Executive Function Impairment: 0
- Neurobehavioral Dysregulation: 0
- Progressive Course: Non-Progressive

Supportive Features
- Depression: 0
- Anxiety: 0
- Apathy: 0
- Paranoia: NM
- Suicidality: NM
- Motor Signs (Parkinsonism / ALS): 0
    - "Neurologic Examination: Normal" (pg. 138)
- Delayed Onset of Symptoms and Problems: 0

Level of Functional Dependence / Dementia: I
- CBI Score: 0
- CTBI Classification: Normal
- "Neurologic Examination: Normal" (pg. 138)

Level of Functional Dependence / Dementia (Including Physical Disability): I

Geddes et al. (1999)

Note: The authors provide a very limited description for each case in terms of their clinical features. The article focuses on neuronal cytoskeletal changes in young adults with history of mild head injury. The case descriptions describe the history of each case, but clinical features are very limited and when they are mentioned, their age of onset and progression are not mentioned. The authors make no reference to these features in any other parts of the article. This article included 5 cases. One of the cases (case #5) has been included in prior reviews of the CTE literature, but it does not appear to be a case of CTE or TES. This was a 23-year-old man who had a personal history of a "severe head injury" from which he was deemed to have made a full recovery. He died from an acute subdural hematoma and brain swelling from a head injury in a football match. This case (case #5) was excluded. Other cases who died due to acute subdural hematoma were included because they had a long history of repetitive neurotrauma (case #1 and #3). It is debatable whether to include case #4 because that person had no sporting history and had lifelong severe intractable epilepsy. We included it in part because past reviewers included it.

Case 1 (Age: 23; Sport: Boxing; reported in Geddes, et al., 1996)
Age started: 11 years (professional at 19)
Age retired: 23 years (death from acute subdural haemmorhage sustained in a boxing match)
Length of career: 12 years (4 professional)
Age of symptom onset: Not Applicable
Delay between retirement and symptom onset (Age of symptom onset – Age retired): Not Applicable

Core Clinical Features

- Cognitive Impairment: 2
    - Per Geddes et al., 1996 "[He] has suffered only one knock-out, in his penultimate contest. He had not previously sustained a serious head injury as a result of a fight, had **no neurological symptoms**, although he was described as being **'somewhat forgetful.'** He had not undergone psychometric testing." (pg. 12)
- Executive Function Impairment: NM
- Neurobehavioral Dysregulation: NM
- Progressive Course: Unable to Classify

Supportive Features

- Depression: NM
- Anxiety: NM
- Apathy: NM
- Paranoia: NM
- Suicidality: NM
- Motor Signs (Parkinsonism / ALS): 0
    - Per Geddes et al., 1996 "He had not previously sustained a serious head injury as a result of a fight, had **no neurological symptoms**…" (pg. 12)
- Delayed Onset of Symptoms and Problems: NA

Level of Functional Dependence / Dementia: II

- "He had no history of a severe head injury during his career, until his final fight, as a result of which he developed an acute subdural haematoma. He died 48 h after the contest, despite neurosurgical intervention." (pg. 172)

Level of Functional Dependence / Dementia (Including Physical Disability): II

Case 2 (Age: 28; Sport: Boxing)
Age started: 16 years
Age retired: 21 years
Length of career: 5 years
Age of symptom onset: 20 years (considering hospitalization for paranoid schizophrenia as first "symptom")
Delay between end of career and symptom onset (Age of symptom onset – Age retired): -1 year*
*symptom onset during career

Core Clinical Features

- Cognitive Impairment: NM
- Executive Function Impairment: NM
- Neurobehavioral Dysregulation: NM
- Progressive Course: Unable to Classify

Supportive Features

- Depression: 1
  - "**At the age of 20** he was admitted to a **psychiatric hospital** with a diagnosis of **paranoid schizophrenia** which responded to major tranquillisers. He was readmitted with an **acute psychotic illness at the age of 25**, **this type more depressive in nature, and then again 2 years later**." (pg. 172)
- Anxiety: NM
- Apathy: NM
- Paranoia: 1
  - "**At the age of 20** he was admitted to a **psychiatric hospital** with a diagnosis of **paranoid schizophrenia** which responded to major tranquillisers. He was readmitted with an **acute psychotic illness at the age of 25**, **this type more depressive in nature, and then again 2 years later**." (pg. 172)
- Suicidality: NM
- Motor Signs (Parkinsonism / ALS): NM
- Delayed Onset of Symptoms and Problems: 0

Level of Functional Dependence / Dementia: III

- "He died unexpectedly the following year during a grand mal seizure. He had no history of a serious head injury during his boxing career." (pg. 172)

Level of Functional Dependence / Dementia (Including Physical Disability): III

Case 3 (Age: 28; Sport: Not applicable; Grand mal epilepsy and autistic head banging)
Age started: Not applicable
Age Retired: Not applicable
Length of career: Not applicable
Age of symptom onset: Not mentioned
Delay between end of career and symptom onset (Age of symptom onset – Age retired): Not applicable

Core Clinical Features

- Cognitive Impairment: 2
    - "A mentally subnormal man of 28, who had been slow to achieve childhood developmental milestones, developed grand mal epilepsy at the age of 7 years, at which time he was also diagnosed to be autistic. There was a long history of head banging, but no episode of severe head injury. He lived his adult life in residential care, and died 2 days after a fall from a first floor window, in which he sustained a skull fracture, extradural and acute subdural haematomas, and large basal frontal contusions." (pg. 172)
- Executive Function Impairment: NM
- Neurobehavioral Dysregulation: NM
- Progressive Course: Unable to Classify

Supportive Features

- Depression: NM
- Anxiety: NM
- Apathy: NM
- Paranoia: NM
- Suicidality: NM
- Motor Signs (Parkinsonism / ALS): NM
- Delayed Onset of Symptoms and Problems: NA

Level of Functional Dependence / Dementia: III

- "A mentally subnormal man of 28, who had been slow to achieve childhood developmental milestones, developed grand mal epilepsy at the age of 7 years, at which time he was also diagnosed to be autistic. There was a long history of head banging, but no episode of severe head injury. He lived his adult life in residential care, and died 2 days after a fall from a first floor window, in which he sustained a skull fracture, extradural and acute subdural haematomas, and large basal frontal contusions." (pg. 172)

Level of Functional Dependence / Dementia (Including Physical Disability): III

Case 4 (Age: 27; Sport: Not applicable; Intractable complex partial seizures with secondary generalized seizures)
Age started: Not applicable
Age Retired: Not applicable
Length of career: Not applicable
Age of symptom onset: Not mentioned
Delay between end of career and symptom onset (Age of symptom onset – Age retired): Not applicable

Core Clinical Features

- Cognitive Impairment: 2
    - "A mentally retarded man of 27 years of age who had suffered from intractable complex partial seizures with secondary generalized seizures." (pg. 172)
- Executive Function Impairment: NM
- Neurobehavioral Dysregulation: NM
- Progressive Course: Unable to Classify

Supportive Features

- Depression: NM
- Anxiety: NM
- Apathy: NM
- Paranoia: NM
- Suicidality: NM
- Motor Signs (Parkinsonism / ALS): NM
- Delayed Onset of Symptoms and Problems: NA

Level of Functional Dependence / Dementia: II

- "A mentally retarded man of 27 years of age who had suffered from intractable complex partial seizures with secondary generalized seizures. He was born 2 months prematurely, suffered from birth trauma and had his first seizure at the age of 2.5 years. The seizure frequency gradually increased, until in recent years he had both "major attacks" seven to ten times a week in which he dropped heavily to the ground and "minor attacks" which occurred once a week." (pg. 172)

Level of Functional Dependence / Dementia (Including Physical Disability): II

Newell & Drachman (1999)

"Case reports of the Massachusetts General Hospital. Weekly clinicopathological exercises. Case 12-1999. A 67-year-old man with three years of dementia."

Case 1 (Age: 67; Sport; Boxing)
Age started: Not mentioned
Age retired: Not mentioned
Length of career: 10 years
Age of symptom onset: Likely 64 years (three years before admission; see below)
Delay between retirement and symptom onset: 40 years

Core Clinical Features

- Cognitive Impairment: 1
    - "A 67-year-old, right-handed man was admitted to the hospital because of **increasing dementia**." (pg. 1269)
    - "**Three years before admission**, the patient's **cognitive function began to decline**. **One year later**, a neurologist **diagnosed progressive dementia with parkinsonism**." (pg. 1269)
    - "On neurologic examination, the patient was **alert but was oriented only to himself**. He believed that he was in his house in the city and that the year was 1970. He **lost concentration when attempting serial tasks**. **Abstract tasks were performed poorly**. He recalled none of three objects at five minutes, even with cues. **Remote autobiographical memories were relatively preserved. Left–right confusion was present**." (pg. 1269)
    - "Dementia… may be **progressive**, as in this case." (pg. 1270)
    - "This patient appears to have had a **progressive and apparently degenerative dementia**." (pg. 1270)
    - "His **symptoms began with dementia**…" (pg. 1272)
- Executive Function Impairment: 1
    - "On neurologic examination, the patient was **alert but was oriented only to himself**. He believed that he was in his house in the city and that the year was 1970. He **lost concentration when attempting serial tasks**. **Abstract tasks were performed poorly**. He recalled none of three objects at five minutes, even with cues. **Remote autobiographical memories were relatively preserved. Left–right confusion was present**." (pg. 1269)
- Neurobehavioral Dysregulation: 1
    - "**During the two months before admission**, the patient **became agitated**, and trazodone was prescribed. His family noted **transient periods when he stared into space and did not respond to his surroundings**." (pg. 1269)
    - "**One week before admission, the patient punched his wife because she would not let him drive. Soon thereafter, he began to look under his bed, locked the doors and windows, and became convinced that the neighbors were trying to steal the family's car**. Subsequently, he **struck his teenage grandson without provocation and wandered from home**. He was referred to this hospital." (pg. 1269)
    - "Trazodone was continued, and treatment with risperidone was begun. The patient became less agitated. On the 16th hospital day, he was transferred to a nursing home, where he died suddenly 5 days later." (pg. 1270)
    - "**Abnormal thought and behavior were an important, although not initial, part of the patient's dementia**, with **paranoid ideas**, **loss of inhibition**, and **aggressive acts**." (pg. 1271)
    - "In this case, the patient's **prominent behavioral problems** were the reason for his prolonged hospitalization and treatment with neuroleptic drugs." (pg. 1271)
    - "His **symptoms began with dementia**, he **later had mild parkinsonism**, **his behavior became troublesome and disinhibited, and he had considerable evidence of motor neuron disease**." (pg. 1272)
- Progressive Course: Progressive
    - "Dementia… may be **progressive**, as in this case." (pg. 1270)
    - "This patient appears to have had a **progressive and apparently degenerative dementia**." (pg. 1270)

Supportive Features

- Depression: NM
- Anxiety: NM
- Apathy: NM
- Paranoia: 1
    - "**Soon thereafter, he began to look under his bed, locked the doors and windows, and became convinced that the neighbors were trying to steal the family's car**." (pg. 1269)
    - "**Abnormal thought and behavior were an important, although not initial, part of the patient's dementia**, with **paranoid ideas**, **loss of inhibition**, and **aggressive acts**." (pg. 1271)
- Suicidality: NM
- Motor Signs (Parkinsonism / ALS): 1
    - "**One year later**, a neurologist **diagnosed progressive dementia with parkinsonism**." (pg. 1269)

- “He **fell several times**, with **apparent loss of consciousness but no obvious injury**.” (pg. 1269)
- “The results of a general physical examination were normal.” (pg. 1269)
- The patient’s **language was intact**, and phrases were repeated accurately. His speech was **hypophonic**. His **facial expression was reduced**, and he **drooled**. **Cranial nerve functions were preserved**. There was **severe atrophy of the shoulder girdles and arms and legs**. **Diffuse fasciculations and muscle twitches were observed.** M**otor power was normal**. The patient’s **posture was slightly stooped**. His **spontaneous gait was normal, and tandem walking was unsteady**. **No resting tremor or cogwheel rigidity** was detected. His s**ense of joint position and perception of vibration were intact**. The deep-tendon reflexes were + or ++ and symmetric. **No bradykinesia or postural instability** was evident, and the **glabellar-tap sign and Babinski signs were absent**.” (pg. 1269)
- “**Two years before the patient was hospitalized, a neurologist had diagnosed ‘progressive dementia with parkinsonism**.’ Neurologic examination at this hospital revealed only minor extrapyramidal features: some reduction of facial expression, mild hypophonia, and a slightly stooped posture, but without marked rigidity, a resting tremor, a gait disorder, or a glabellar sign.” (pg. 1271)
- “In this case, the patient’s **prominent behavioral problems** were the reason for his prolonged hospitalization and treatment with neuroleptic drugs. A striking feature of this case, with important diagnostic implications, was the **presence of motor neuron disease**, with severe atrophy of both shoulder girdles and distal muscles, and diffuse fasciculations. Muscle strength was reported to be normal, and Babinski signs were absent. Nevertheless, an electromyogram, which is not part of the routine workup for dementia, showed chronic denervation and reinnervation in all muscle groups, a finding consistent with the presence of a ‘generalized disorder of motor neurons, their axons, or both.’” (pg. 1271)
- “His **symptoms began with dementia**, he **later had mild parkinsonism**, **his behavior became troublesome and disinhibited, and he had considerable evidence of motor neuron disease**.” (pg. 1272)

- Delayed Onset of Symptoms and Problems: 1
  - “The **long period of apparently normal functioning after his retirement from boxing** is somewhat unusual.” (pg. 1273)
  - “We **favored the diagnosis of dementia pugilistica**, although the **40-year period of latency between his retirement from boxing and the emergence of his disease was not consistent with this diagnosis.**” (pg. 1274)

Level of Functional Dependence / Dementia: IV
Level of Functional Dependence / Dementia (Including Physical Disability): IV

Quotes from the Literature Relating to the Course of the Clinical Condition

Martland, 1928: “Many cases remain mild in nature and do not progress beyond this point. In others a very distinct dragging of the leg may develop and with this there is a general slowing down in muscular movements, a peculiar mental attitude characterized by hesitancy in speech, tremors of the hands and nodding movements of the head, necessitating withdrawal from the ring. Later on, in severe cases, there may develop a peculiar tilting of the head, a marked dragging of one or both legs, a staggering, propulsive gait with the facial characteristics of the parkinsonian syndrome, or a backward swaying of the body, tremors, vertigo and deafness. Finally, marked mental deterioration may set in necessitating commitment to an asylum. Of course the symptoms produced by the late manifestations of epidemic encephalitis, by the juvenile and presenile types of paralysis agitans, by syphilis, brain tumors and other forms of cerebral injury may so closely resemble those of the condition punch drunk as to be differentiated only with extreme difficulty or not at all. Nevertheless, the occurrence of the symptoms in almost 50 per cent of fighters who develop this condition in mild or severe form, if they keep at the game long enough, seems to be good evidence that some special brain injury due to their occupation exists.” (pg. 1103)

Martland, 1928: “As far as I know this condition has practically not been described in medical literature. I am of the opinion that in punch drunk there is a very definite brain injury due to single or repeated blows on the head or jaw which cause multiple concussion hemorrhages in the deeper portions of the cerebrum. Such hemorrhages are very apt to occur in or near the corpora striata, in the corona radiata but almost never in the cerebral cortex or below the tentorium cerebelli. These hemorrhages are later replaced by a gliosis or a degenerative progressive lesion in the areas involved. Therefore, in late stages the symptoms often mimic those seen in diseases characterized by the parkinsonian syndrome. I realize that this theory, while alluring, is quite insusceptible of proof at the present time, but I am so convinced from my former studies on post-traumatic encephalitis that this is the logical deduction that I feel it my duty to report this condition.” (pg. 1103)

Martland, 1928: “A replacement gliosis or even a progressive degenerative lesion may be the late manifestations of these former hemorrhages. It is not surprising, then, that some of these cases will mimic the juvenile and presenile forms of paralysis agitans or the late manifestations of epidemic encephalitis. Especially is this so when the frequent location of the hemorrhages in the corpora striata is recalled.” (pg. 1107)

Martland, 1928: “The condition of punch drunk has been described. While most of the evidence supporting the existence of this condition is based at this time on the observations of fight fans, promoters and sporting writers, the fact that nearly one half of the fighters who have stayed in the game long enough develop this condition, either in a mild form or a severe and progressive form which often necessitates commitment to an asylum, warrants this report. The condition can no longer be ignored by the medical profession or the public. It is the duty of our profession to establish the existence or non-existence of punch drunk by preparing accurate statistical data as to its incidence, careful neurologic examinations of fighters thought to be punch drunk, and careful histologie examinations of the brains of those who have died with symptoms simulating the parkinsonian syndrome. The late manifestations of punch drunk will be seen chiefly in the neurologic clinics and asylums, and such material will practically fall to the neuropathologist connected with such institutions.” (pg. 1107)

Parker, 1934: “According to Martland… Lastly, a progressive neurological syndrome may appear, putting an end to all fighting, and leading finally to mental of physical helplessness. It is well understood, moreover, that pugilists are not immune to the ravages of syphilis, alcohol, or even epidemic encephalitis, and certainly the incidence of these diseases is no lower among pugilisits than in the general population. The point that Martland urged, however, was that the high frequency with which professional pugilists develop crippling disease of the central nervous system of one sort or another suggests a result of the repeated injuries to the brain that they received while carrying on the activities of their profession.” (pg. 20-21)

Parker, 1934: “Jokl and Guttman have recently reviewed the subject of symptoms referable to the central nervous system presented by pugilists. They divide the symptoms into three classes: those appearing in the course of the actual bout; those coming on some hours or days thereafter, and those appearing in the later life of the pugilists. The latter they divide into mental and physical. Mental deterioration, they think, can afflict patients who are predisposed to it even before they enter

the ring, but they also think a definite dementia affects former pugilisits as a result of cerebral trauma received during their careers. On the physical side, Jokl and Guttmann describe changes in tendon reflexes, dysarthria, disturbances of coordination and anomalies of speech.” (pg. 27)

Critchley, 1949 (5): “The punch-drunk condition is usually a progressive one, with both psychological and neurological features, which help to explain the variety of case-records.” (pg. 131)

Raevuori-Nallinmaa, 1950: “The morbid changes can appear either immediately after the trauma or after a period of latency. Once the symptoms have appeared they remain permanent. Prognosis is generally considered rather bad and it is made still worse by the scarcity of the subjective symptoms even in long progrediated cases. The lack of a feeling of illness is typical of the clinical picture. An investigator accordingly remarks that it is difficult to convince a boxer who does not feel ill that he has to give up boxing, which means continued exposure to traumas. Parker remarks that a progress of symptoms was noticed in two out of three boxers even a few years after they had left the ring.” (pg. 53)

Critchley, 1957 (8): “Of great interest, pathological as well as practical, is the fact that this traumatic encephalopathy is a progressive condition. Once established it not only does not permit of reversibility, but it ordinarily advances steadily. This is the case even though the boxer has retired from the ring and repeated cranial traumata are at an end.” (pg. 360)

Corsellis & Brierley, 1959: ‘It is generally accepted that injury to the head may be followed at times by a deterioration in behavior and in personality which in rare cases may progress insidiously to a state of advanced dementia.’ (pg. 718)

Corsellis & Brierley, 1959 (10): ‘Critchley (1957) in his review of 69 boxers with chronic neurological disease pointed out that the well-known condition of ‘punchdrunkenness’ might be more accurately described as a ‘chronic progressive traumatic encephalopathy’ since one of its most interesting features, especially from the pathological point of view, is the fact that it does not remit but that it steadily advances even though the boxer has long since retired’ (pg. 718).

Corsellis & Brierley, 1959 (10): ‘In fact the insidious nature of this clinical deterioration and the way in which it may progress long after the last injury would be in many ways better explained, not in terms of scarring, but by the development following trauma of some form of progressive degeneration of cerebral tissue’ (pg. 718).

Corsellis & Brierley, 1959: ‘Grahmann and Ule, after comparing their case with that of Brandenburg and Hallervorden, remarked that “the fundamental similarity of the morphological findings in these, the only two demented boxers so far to have been examined pathologically, is certainly no accident”. They interpreted the findings in much the same way as Brandenburg and Hallervorden and pointed to the slowly progressive type of pathological process that was present in these cases.’ (pg. 719)

Neubuerger, Sinton, & Denst, 1959: ‘In an article published several years ago in a popular magazine, Blitman described the mental status of former boxers. He drew on his personal experience; he knew "what it means to be punch drunk." He vividly depicted momentary loss of coordination, brief mental black-outs, sudden waves of anger, lapses of memory, trembling hands, sharp headaches, dizzy spells, sudden jerkiness, uncontrollable shaking of the head, and ringing in the ears. The author was "one of the lucky ones," however; he "came back almost all the way physically," and his "mind remained whole." But he emphasized that "there are other men who have trouble holding onto the most menial of jobs," owing to steadily progressive physical, nervous, and mental deterioration.’ (pg. 19/403)

Neubuerger, Sinton, & Denst, 1959: ‘Grahmann and Ule… The authors felt that the term “dementia pugilistica” should be reserved for cases of chronic progressive disorders developing after a symptom-free interval, and that neurologic disturbances arising in immediate connection with head injuries should be called traumatic encephalopathy of boxers. To our knowledge, these are the only previously reported cases of progressive mental deterioration in boxers in which histologic examination has been undertaken.’ (pgs. 22/406-23/407)

Courville, 1962: ‘In 1928, Martland reported a symptom complex which had long been known to professional pugilists. It seemed to be the result of repeated episodes of cerebral concussion in the course of the bouts of second rate or older boxers. This state is first manifested by acute symptoms, such as mental confusion, disorientation, and amnesia, with subsequent dizziness and headaches, following a single knockout in the ring. The occurrence of repeated concussive

episodes leads to the development of typical chronic cases. Symptoms tend to progress for a year or more, then become stationary if the individual gives up boxing. In the few who persist, deterioration sometimes progresses to a degree that hospitalization becomes necessary because of a full-blown "traumatic encephalopathy".' (pg. 160)

Courville, 1962: 'This condition was described by Martland (1928) as a progressive slowing down of muscular movements, with tremor of the hands, nodding of the head, dragging of one or both legs, hesitancy of speech, staggering propulsive gait, ironed-out facies, and marked mental deterioration.' (pg. 160)

Courville, 1962: 'The changes found in the case of chronic pugilistic encephalopathy here reported should be considered with those reported in other cases. A study of these changes makes it clear that this clinical complex is not a single entity. Grahmann and Ule (1957) and others have postulated at least three separate states. (1) In *dementia pugilistica* a progressive cerebral disorder becomes evident at some interval after the boxing career, and structural changes comparable to those of postencephalitic parkinsonism gradually develop. There is an associated progressive dementia upon which the diagnosis of boxer's dementia or dementia pugilistica is based. (2) In boxer's *traumatic encephalopathy* the clinical findings are stationary and are directly related to an episode of blows to the head. (3) In the third state, a *paranoid and/or catatonic psychosis*, the specific causal relation between boxing and the psychosis is problematical.' (pg. 166)

Courville, 1962: 'The first syndrome of dementia pugilistica includes the manifestation of parkinsonism plus a progressive dementia.' (pg. 166)

Spillane, 1962: Discussed individual cases as progressive.

Mawdsley & Ferguson, 1962: 'In the remainder of our patients progressive disabilities began when they were relatively young, usually at the end of their boxing careers or shortly after retirement.' (pg. 799)

Mawdsley & Ferguson, 1963 (16): 'Boxing is sometimes the cause of progressive neurological disease' (pg. 801).

Payne, 1968 (18): 'In 1957 Grahmann and Ule... They suggested that the term Dementia Pugilistica should be reserved for a chronic progressive brain disorder which develops after a symptom-free interval and that brain disorders which occur immediately following a boxing contest should be known as 'Traumatic Encephalopathy of Boxers'' (pg. 185).

Johnson, 1969 (47): 'Millspaugh (1937) called the condition "dementia pugilistica." The term "dementia" is, however, clinically inaccurate in many cases, and it has now been replaced by the more acceptable title of the "chronic progressive traumatic encephalopathy of boxers" (Critchley, 1957). Although this stresses the progressive nature of the encephalopathy, not all cases follow such a course. Symonds (1960) stated that in some cases the condition may be mild and non-progressive, whilst Bowman and Blau (1960) maintained that progression occurs for only a few years after the boxer's retirement, the condition thereafter becoming stationary' (pg. 45).

Johnson, 1969: '*Dementia*. This is defined as a progressive, irreversible disorganization of personality, particularly affecting cognitive function, due to organic, cerebral disease. Recent semantic confusions surrounding this term has been reviewed by Stengel (1964)… In Cases 1 and 16, dementia was paralleled by progressive neurological disability.' (pg. 47).

Johnson, 1969: 'Deep-midline lesions in and around the third ventricle may produce severe defects of retention, impaired learning and decay of recent memory (Williams et al., 1954). The chronic amnestic syndrome present in most of these boxers was of a benign, non-progressive form and dated from the end of their fighting career. It was characterized by poor retention and defective immediate recall of impersonal data, associated with a learning defect in psychometric testing: older memories, social habits and acquired skills were not affected. Neuroradiological evidence of brain damage around the third ventricle and neurological evidence of damage in the mid-brain suggest that the lesions are probably widespread throughout the amnestic system.' (pg. 50)

Johnson, 1969: 'In three cases (1, 16, 17) there was dementia due to a progressive cerebral degenerative process. In the three neuropathological studies of boxers' brains already referred to there were clinical histories similar to those of Cases 1 and 16, and at post-mortem these brains showed atrophy of the frontal and temporal cortex with histopathological

appearances resembling those seen in Alzheimer's disease. The possibility that a progressive encephalopathy producing a clinical picture of pre-senile dementia can be initiated by head injury has been considered (McMenemy et al., 1961).' (pg. 50)

Johnson, 1969: 'The neurological features may assume predominance over the psychiatric features and vice versa. They may progress independently of each other, lending further support to the view that they are dependent upon lesions in different neural systems. The accepted title given to this condition by Critchley (1957) implies that progression is inevitable. Certainly this is not so in all cases… The aetiological title "chronic traumatic encephalopathy of boxing" or the clinical title "chronic neuropsychosyndrome of boxing" might be more widely applicable.' (pg. 52)

Johnson, 1969: 'How might repeated head trauma induce a progressive degenerative process in the brain? Neubuerger et al. (1959) applied von Braunmuhls concept of thixotropy to this problem. Thixotropy is the disturbance of a gel-sol equilibrium in a colloidal system such as the brain by mechanical stress. The repetitive mechanical agitation of cerebral colloids through blows to the head received in boxing might induce such a change in the cerebral proteins, producing a progressive degenerative process in the cells, or by altering their protein structure affect the molecular basis of memory function (Hoenig, 1965). Alternatively, it might be caused by an auto-immune response due to cell damage from repeated head trauma. It may well be that many of the cases of traumatic encephalopathy are now anachronisms, arising as they did in the lean economic years of the 1930's when there were few controls and little or no medical surveillance of boxing at either amateur or professional levels. Increased awareness of this syndrome and improved controls over boxing contests may eliminate its development in future years. If, however, head injury can induce changes in cerebral protein structure and in turn affect the molecular basis of memory, minor forms of this syndrome will continue to occur.' (pg. 52)

Roberts, 1969 (2): 'There appears to have been no earlier report in the medical literature of chronic neurological disease attributed to boxing before the account of a single case in a paper by Martland (1928). In his discussion of legitimate doubts that might be raised in implying a causal connection between a career as a professional boxer and the development of a progressive extra-pyramidal syndrome simulating paralysis agitans, Martland said that he had examined five punchdrunk boxers, but thought that only by the careful collection of statistical, clinical and neuropathological data would the existence of the syndrome be definitely established.' (pg. 14)

Roberts, 1969: 'Where independent accounts were available, or the individuals retained insight, there was good evidence that the disabilities had developed in their last years boxing and had been little changed, or become only very slowly more apparent, as they grew older.' (pg. 37)

Roberts, 1969: 'In only three of these cases was there any definite evidence to indicate that the condition had progressed since they gave up boxing. In one the progression had been a sequel to a severe head injury in a road traffic accident and there was no suggestion that his condition had remained other than stationary when he had recovered from this. In the remaining two such mild progression in the lesions, and particularly in their forgetfulness, as had occurred, might as well have been due to normal ageing as to any other process.' (pg. 46)

Roberts, 1969: 'When there is an adequate independent account, or the individual has sufficient insight, most of these boxers appear to have developed the symptoms of the condition during their last years boxing or after a series of particularly hard fights. It seems that some degree of progression may occur which cannot be accounted for simply on the basis of normal ageing, and occurs more commonly in the extra-pyramidal lesions, but apart from a few isolated exceptions reported previously and in one case in the present series (Case 2), this progression is not in general characteristic of that seen in system degenerations, or in the commoner presenile dementias. There is a good deal of evidence in the present study to suggest that in most cases the condition remains stationary when the individual has stopped boxing, and indeed there are excellent independent accounts for a few of undoubted improvement after their retirement.' (pg. 48)

Roberts, 1969: 'It seems reasonable to suppose, in most cases of traumatic encephalopathy of boxers where there has been progression in the evidence of intellectual incapacity and disturbance of motor functions some years after their retirement from the ring, that these reflect the changes associated with ageing in brains in which there has already been some depletion of the neural functional reserves. This, rather than the assumption that some delayed diffuse degenerative

process has been provoked by repetitive trauma in the past, would be in keeping with clinical experience of the sequelae of repeated cerebral trauma outside the boxing ring (Symonds, 1941)' (pg. 98)

Roberts, 1969: 'Certainly, a progressive dementing process attributable to a single head injury is rare (Corsellis and Brierley, 1959), and remarkable degrees of recovery in the evidence of the neural lesions, even after the most severe closed head injury, are not uncommon (Miller and Stern, 1965; Caveness, 1966; Fahy, Irving and Millac, 1967). On the other hand, similar pathological lesions have been found in the four brains of ex-boxers referred to briefly by Ferguson and Mawdsley (1965). The findings in none of these were altogether typical of Alzheimer's disease, in that there were few plaques in proportion to the widespread neurofibrillary changes scattered throughout the hemispheres and these latter were present in unusual profusion in the mid-brain of each specimen (Yates, 1969).' (pg. 106)

Roberts, 1969: 'The only case of traumatic encephalopathy in the present study in which progression had occurred in a manner typical of a system degeneration, was one with a parkinsonian syndrome; and it is of interest that the unilateral rest tremors of three others had become apparent, or had perhaps worsened, since they retired from the ring.' (pg. 107)

Roberts, 1969: 'How far it can be assumed that a progressive degenerative process involving the neuron is mirrored in the widespread neurofibrillary changes found in some ex-boxers' brains remains unanswered. It seems certain that diffuse cellular and axonal depletion has resulted from boxing to account for the clinical syndrome related to occupational exposure found in the present study. It must therefore be assumed that cerebral trauma of this relatively minor degree may result in permanent structural damage that is cumulative. There was good evidence in some cases that the condition had progressed some time after retirement from boxing, particularly in those with evidence of extra-pyramidal lesions, but the information available from the study proved inadequate to settle finally the question of progression unrelated to the changes associated with ageing.' (pgs. 107-108)

Roberts, 1969: '…but as with the other neurological lesions there were indications less clearly demonstrable of all degrees of defective function, particularly of memory. The evidence for progression in these lesions other than that conceivably attributable to normal ageing was poor, except in the case of one individual with a predominantly cerebellar and dementing syndrome, and in the case of three with extra-pyramidal syndromes.' (pg. 110)

Corsellis, Bruton, & Freeman-Browne, 1973: 'The rest, whether they died in psychiatric or in general hospitals, had all been going downhill mentally for some time previously. The deterioration, however, did not follow the course of the common forms of senile or pre-senile dementia. On the contrary, this category of progressive disease was only once encountered (no. 13) and in most cases the mental and the neurological disorder smouldered on for a long time, little or even no worsening being noticed at least until the last few years or months before death, at a time when cerebrovascular disease is liable to make itself felt. The rate at which the syndrome usually progressed is shown by the fact that, although its first signs were observed nearly always in early or middle adult life, the mean age at death was 69 and even the youngest survived until the age of 57 years.' (pg. 300)

Corsellis, Bruton, & Freeman-Browne, 1973: 'Certainly the present picture approximated more to the lower end of the scale of the 'punch-drunk syndrome' as first described by Martland (1928). 'Many cases', he wrote, 'remain mild in nature and do not progress… In others a very distinct dragging of the leg may develop, and with this there is a general slowing down in muscular movements, a peculiar mental attitude characterized by hesitancy in speech, tremors of the hands and nodding movements of the head, necessitating withdrawal from the ring. Later on, in severe cases there may develop a peculiar tilting of the head, a marked dragging of one or both legs, a staggering propulsive gait with the facial characteristics of the Parkinsonian syndrome… Finally, marked mental deterioration may set in necessitating commitment to an asylum'. (pg. 300)

Corsellis, Bruton, & Freeman-Browne, 1973: 'The severity of the condition varies greatly, ranging from a mild clumsiness of speech and movement, with or without some loss of memory to the ataxic, dysarthric, and perhaps Parkinsonian, dement'. (pg. 301)

Corsellis, Bruton, & Freeman-Browne, 1973: 'A single punch, or even many punches, to the head need not visibly alter the structure of the brain but there is still the danger that, at an unpredictable moment and for an unknown reason, one or more blows will leave their mark. The destruction of cerebral tissue will have then begun and, although this will usually

be slight enough in the early states to be undetectable, it may build up, if the boxing continues, until it becomes clinically evident. At this point, however, it could already be too late, for destroyed cerebral tissue can never be replaced, while the further danger exists that the process of degeneration could smoulder on even after the boxing has stopped.' (pg. 302)

Harvey & Newsom Davis, 1974: 'The traumatic encephalopathy of boxers presents as a relatively stereotyped clinical picture characterised by evidence of damage to one or more of the pyramidal, extrapyramidal, or cerebellar systems with associated dementia, psychosis, personality change, and social instability in some cases. Symptoms tend to be progressive and may develop during the boxer's career or begin many years after retirement from the ring.' (pg. 928)

Kaste et al., 1982: 'The progress of boxer's encephalopathy is often slow, and severe symptoms may appear later in life. Because our boxers were quite young, even those without current subjective symptoms or neurological deficits are still at risk of subsequent symptoms and signs.' (pg. 1187)

Roberts et al., 1990: 'Repeated blows to the head of boxers sometimes culminate in the "punch drunk syndrome" (dementia pugilistica), a condition characterised by damage to the pyramidal, extrapyramidal, and cerebellar systems and by progression from affective disturbance and memory loss, through psychosis, to progressive dementia.' (pg. 918)

Roberts et al., 1990: 'Associations between single traumatic head injury and subsequent Alzheimer dementia have been reported and long-term survivors of head injury are at increased risk of degenerative dementia, but this is by no means conclusive evidence for a link between head injury and an increased risk of subsequent degenerative dementing condition. Dementia in former professional boxers is much more tightly linked to the repeated head injury but the relevance of observations on this unusual group to our understanding of Alzheimer's disease in the "normal" aged has been questioned. Our case helps to fill the gap in the evidence for the concept that head injury can be followed by Alzheimer-type degeneration.' (pg. 919)

Hoff et al., 1991: 'Moreover, chronic SIB may cause long-term brain lesions which could be related to the progressive deterioration observed in our patient.' (pg. 325)

Williams & Tannenberg, 1996: 'Dementia pugilistica, otherwise known as chronic progressive post-traumatic encephalopathy of boxing, or the punch-drunk syndrome, describes the chronic neurological deficits which may develop as a sequel to boxing (cite). This progressive disease develops insidiously after a latent period of some years (cite).' (pg. 102)

Jordan et al., 1997: "Chronic traumatic brain injury (CTBI) is the most serious public health concern in modern-day boxing. Also known as dementia pugilistica or chronic traumatic encephalopathy, CTBI represents the long-term and cumulative neurologic consequences of repetitive head trauma. This syndrome has been most typically described in active or retired boxers after a long exposure to the sport. However, CTBI may also be anticipated in other sports such as American football, soccer, or ice hockey. Clinically, CTBI is characterized by a varied constellation of cognitive impairment, parkinsonism, ataxia, pyramidal tract dysfunction, and behavioral changes." (pg. 136)

Newell & Drachman, 1999: "The neurologic impairment usually begins near the end of the fighter's career or shortly after retirement and evolves over a period of a few years, although its onset may be delayed for a considerably longer period." (pg. 1272)

Jordan, 2000 (PMID: 10946737): 'The severe form of CTBI is referred to as dementia pugilistica. The diagnosis of CTBI is dependent upon documenting a progressive neurological condition that is consistent with the clinical symptomatology of CTBI attributable to brain trauma and unexplainable by an alternative pathophysiological process.' (pg. 179)

Jordan, 2000 (PMID: 10946737): 'Chronic traumatic brain injury (CTBI) of boxing, also known as dementia pugilistica, chronic traumatic encephalopathy or the punch drunk syndrome, represents the cumulative, long-term neurological consequences of repetitive concussive and subconcussive blows to the brain.' (pg. 179)

Jordan, 2000 (PMID: 10946737): 'Although the precise duration of time necessary to develop CTBI after the initiation or cessation of a boxing career is variable, retired boxers examined after the age of 50 years are more likely to exhibit

neurological symptoms. Whether this phenomenon reflects a progressive neuropathological lesion and/or an aging process super-imposed on a fixed neurological injury remains to be determined. Early motor impairments may include mild dysarthria and mild difficulty with balance. From a personal experience, abnormalities with tandem (sharpened) Romberg may be an early finding. As the motor abnormalities progress, the boxer can experience ataxia, spasticity, impaired coordination, and parkinsonism. The parkinsonism encountered in CTBI may be an entity distinct from idiopathic Parkinson's disease. Cognitive impairment in CTBI is most likely to affect attention, memory, and executive or frontal function. Early manifestations of CTBI can include decreased complex attention followed by slowed mental speed and mild deficits in memory, attention, and executive ability. As the disease process advances, the boxer may exhibit dementia exemplified by amnesia, profound attentional defects, prominent slowness of thought, and impaired judgment, reasoning, and planning. Behavioral manifestations are an integral component of CTBI but may be difficult to dissociate from premorbid personality characteristics. Behavioral changes encountered can include disinhibition, irritability, euphoria or hypomania, impaired insight, paranoia, and violent outbursts. Debate exists as to whether the behavioral abnormalities occur as an early or late manifestation of the CTBI syndrome.' (pg. 180)

Jordan, 2000 (PMID: 10946737): 'The pathophysiologic mechanisms of CTBI are unknown. It has been hypothesized by Martland that this syndrome is secondary to single or repeated head blows resulting in multiple petechial hemorrhages in the deeper portions of the cerebrum that are later replaced by gliosis or a progressive degenerative lesion. It can also be theorized that for clinical symptoms of CTBI to develop, a critical number or percentage of functional neurons must be damaged or experience cell death. Conceivably, a boxer who terminates a boxing career and has experienced some neuronal loss does not exhibit clinical symptoms consistent with CTBI because he has a critical number or percentage of functional neurons. However, as he experiences normal or accelerated neuronal dropout associated with aging, he may develop clinical signs of CTBI because he has less than the critical threshold level of functioning neurons. This theory would explain why CTBI appears to progress after the termination of a boxing career and the cessation of cerebral trauma.' (pg. 182)

Cases Included in Other Review Papers

McKee et al. (2009) References for Cases

*Case 1: 30. Brandenburg W, Hallervorden J. [Dementia pugilistica with anatomical findings.]. Virchows Arch 1954;325:680Y709

*Case 2: 31. Grahmann H, Ule G. [Diagnosis of chronic cerebral symptoms in boxers (dementia pugilistica & traumatic encephalopathy of boxers)]. Psychiatr Neurol (Basel) 1957;134:261Y83

Case 3: 32. Neubuerger KT, Sinton DW, Denst J. Cerebral atrophy associated with boxing. AMA Arch Neurol Psychiatry 1959;81:403Y8

Case 4: 32. Neubuerger KT, Sinton DW, Denst J. Cerebral atrophy associated with boxing. AMA Arch Neurol Psychiatry 1959;81:403Y8

Case 5: 28. Courville CB. Punch drunk. Its pathogenesis and pathology on the basis of a verified case. Bull Los Angel Neuro Soc 1962;27:160Y68

Case 6: 33. Mawdsley C, Ferguson FR. Neurological disease in boxers. Lancet 1963;2:799Y801

Case 7: 33. Mawdsley C, Ferguson FR. Neurological disease in boxers. Lancet 1963;2:799Y801

*Case 8: 34. Constantinidis J, Tissot R. [Generalized Alzheimer's neurofibrillary lesions without senile plaques (Presentation of one anatomo-clinical case)]. Schweiz Arch Neurol Neurochir Psychiatr 1967;100:117Y30

Case 9: 35. Payne EE. Brains of boxers. Neurochirurgia (Stuttg) 1968;11:173Y88

Case 10: 35. Payne EE. Brains of boxers. Neurochirurgia (Stuttg) 1968;11:173Y88

Case 11: 35. Payne EE. Brains of boxers. Neurochirurgia (Stuttg) 1968;11:173Y88

Case 12: 35. Payne EE. Brains of boxers. Neurochirurgia (Stuttg) 1968;11:173Y88

Case 13: 35. Payne EE. Brains of boxers. Neurochirurgia (Stuttg) 1968;11:173Y88

Case 14: 35. Payne EE. Brains of boxers. Neurochirurgia (Stuttg) 1968;11:173Y88

Case 15: 29. Corsellis JA, Bruton CJ, Freeman-Browne D. The aftermath of boxing. Psychol Med 1973;3:270Y303

Case 16: 29. Corsellis JA, Bruton CJ, Freeman-Browne D. The aftermath of boxing. Psychol Med 1973;3:270Y303

Case 17: 29. Corsellis JA, Bruton CJ, Freeman-Browne D. The aftermath of boxing. Psychol Med 1973;3:270Y303

Case 18: 29. Corsellis JA, Bruton CJ, Freeman-Browne D. The aftermath of boxing. Psychol Med 1973;3:270Y303

Case 19: 29. Corsellis JA, Bruton CJ, Freeman-Browne D. The aftermath of boxing. Psychol Med 1973;3:270Y303

Case 20: 29. Corsellis JA, Bruton CJ, Freeman-Browne D. The aftermath of boxing. Psychol Med 1973;3:270Y303

Case 21: 29. Corsellis JA, Bruton CJ, Freeman-Browne D. The aftermath of boxing. Psychol Med 1973;3:270Y303

Case 22: 29. Corsellis JA, Bruton CJ, Freeman-Browne D. The aftermath of boxing. Psychol Med 1973;3:270Y303

Case 23: 29. Corsellis JA, Bruton CJ, Freeman-Browne D. The aftermath of boxing. Psychol Med 1973;3:270Y303

Case 24: 29. Corsellis JA, Bruton CJ, Freeman-Browne D. The aftermath of boxing. Psychol Med 1973;3:270Y303

Case 25: 29. Corsellis JA, Bruton CJ, Freeman-Browne D. The aftermath of boxing. Psychol Med 1973;3:270Y303

Case 26: 29. Corsellis JA, Bruton CJ, Freeman-Browne D. The aftermath of boxing. Psychol Med 1973;3:270Y303

Case 27: 29. Corsellis JA, Bruton CJ, Freeman-Browne D. The aftermath of boxing. Psychol Med 1973;3:270Y303

Case 28: 29. Corsellis JA, Bruton CJ, Freeman-Browne D. The aftermath of boxing. Psychol Med 1973;3:270Y303

Case 29: 29. Corsellis JA, Bruton CJ, Freeman-Browne D. The aftermath of boxing. Psychol Med 1973;3:270Y303

Case 30: 18. Roberts GW, Whitwell HL, Acland PR, et al. Dementia in a punch-drunk wife. Lancet 1990;335:918Y19

Case 31: 14. Hof PR, Knabe R, Bovier P, et al. Neuropathological observations in a case of autism presenting with self-injury behavior. Acta Neuropathol 1991;82:321Y26

Case 32: 36. Hof PR, Delacourte A, Bouras C. Distribution of cortical neuro- fibrillary tangles in progressive supranuclear palsy: A quantitative analysis of six cases. Acta Neuropathol 1992;84:45Y51

Case 33: 36. Hof PR, Delacourte A, Bouras C. Distribution of cortical neuro- fibrillary tangles in progressive supranuclear palsy: A quantitative analysis of six cases. Acta Neuropathol 1992;84:45Y51

Case 34: 19. Williams DJ, Tannenberg AE. Dementia pugilistica in an alcoholic achondroplastic dwarf. Pathology 1996;28:102Y4

Case 35: 15. Jordan BD, Kanik AB, Horwich MS, et al. Apolipoprotein E epsilon 4 and fatal cerebral amyloid angiopathy associated with dementia pugilistica. Ann Neurol 1995;38:698Y99

Case 36: 13. Geddes JF, Vowles GH, Nicoll JA, et al. Neuronal cytoskeletal changes are an early consequence of repetitive head injury. Acta Neuropathol 1999;98:171Y78 AND 37. Geddes JF, Vowles GH, Robinson SF, et al. Neurofibrillary tangles, but not Alzheimer-type pathology, in a young boxer. Neuropathol Appl Neurobiol 1996;22:12Y16

Case 37: 13. Geddes JF, Vowles GH, Nicoll JA, et al. Neuronal cytoskeletal changes are an early consequence of repetitive head injury. Acta Neuropathol 1999;98:171Y78
Case 38: 13. Geddes JF, Vowles GH, Nicoll JA, et al. Neuronal cytoskeletal changes are an early consequence of repetitive head injury. Acta Neuropathol 1999;98:171Y78
Case 39: 13. Geddes JF, Vowles GH, Nicoll JA, et al. Neuronal cytoskeletal changes are an early consequence of repetitive head injury. Acta Neuropathol 1999;98:171Y78
Case 40: 13. Geddes JF, Vowles GH, Nicoll JA, et al. Neuronal cytoskeletal changes are an early consequence of repetitive head injury. Acta Neuropathol 1999;98:171Y78
Case 41: 38. Newell KL, Drachman DA. Case records of the Massachusetts General Hospital. Weekly clinicopathological exercises. Case 12-1999. A 67-year-old man with three years of dementia. N Engl J Med 1999; 340:1269Y77

Cases After 2000

*Case 42: 39. Schmidt ML, Zhukareva V, Newell KL, et al. Tau isoform profile and phosphorylation state in dementia pugilistica recapitulate Alzheimer's disease. Acta Neuropathol 2001;101:518Y24
*Case 43: 17. Omalu BI, DeKosky ST, Minster RL, et al. Chronic traumatic enceph- alopathy in a National Football League player. Neurosurgery 2005;57: 128Y34
*Case 44: 16. Omalu BI, DeKosky ST, Hamilton RL, et al. Chronic traumatic encephalopathy in a national football league player: Part II. Neuro- surgery 2006;59:1086Y92
*Case 45: 21. Schwarz A. Expert ties ex-player's suicide to brain damage [New York Times Web site]. January 18, 2007. Available at: http://www.nytimes.com/ 2007/01/18/sports/football/18waters.html. Accessed January 26, 2008.
*Case 46: 20. Cajigal S. Brain damage may have contributed to former wrestler's violent demise. Neurology Today 2007;7:1, 16
*Case 47: 40. Schwarz A. Lineman, dead at 36, exposes brain injuries [New York Times Web site]. June 15, 2007. Available at: http://www.nytimes. com/2007/06/15/sports/football/15brain.html. Accessed March 11, 2009
*Case 48: 41. Areza-Fegyveres R, Rosemberg S, Castro RM, et al. Dementia pugilistica with clinical features of Alzheimer's disease. Arq Neuro- psiquiatr 2007;65(3B):830Y33

*Cases that were not included in the present review. There were 3 cases from the 20th century described in articles that were not written in English, and there were 7 cases in articles published in the early 20th century. We included only cases from the 20th century. For the statistical analyses, we included only cases that were boxers.

Victoroff (2013)

*1 Aotsuka, et al., 1990
*2 Areza-Fegyveres et al., 2007
*3 Bouras, Hof, Guntern, & Morrison, 1990
*4 Brandenburg & Halloverden, 1954
*5 Casson, Sham, Campbell, Tarlau, & DiDomenico, 1982
6 Casson et al., 1984
7 Constantinides & Tissot, 1967
*8 Cordero Jr. & de Oliviera, 2001
9 Corsellis, Bruton, & Freeman-Browne, 1973
10 Courville, 1962
11 Critchley, 1949
12 Critchley, 1957
13 Drachman & Newell, 1999
14 Geddes, Vowles, Robinson, & Sutcliffe, 1996
15 Geddes, Vowles, Nicoll, & Revesz, 1999
*16 Grahmann & Ule, 1957
17 Harvey & Newsom Davis, 1974
18 Hof, Knabe, Bovier, & Bouras, 1991
19 Hof et al., 1992
*20 Hof, Delacourte, & Bouras, 1992
*21 Jedlinski, Gatarski, & Szymusik, 1970

22 Johnson, 1969
23 Jordan, 1995
24 Jordan et al., 1997
25 Kaste et al., 1982
26 Martland, 1928
27 Mawdsley & Ferguson, 1963
*28 McKee et al., 2009
*29 McKee et al., 2010
30 Neuberger, Sinton, & Denst, 1959
*31 Nowak, Smith, & Reyes, 2009
*32 Omalu et al., 2005
*33 Omalu et al., 2006
34 Parker, 1934
35 Payne, 1968
36 Raevuori-Nallinmaa, 1951
37 Roberts, 1969
38 Roberts, Allsop, & Bruten, 1990
*39 Rodriguez, Ferrillo, Montano, Rosadini, & Sannita, 1983
*40 Ross, Cole, Thompson, & Kim, 1983
*41 Schmidt, Zhukareva, Newell, Lee, & Trojanowski, 2001
*42 Sercl & Jaros, 1962
43 Spillane, 1962
44 Williams & Tannenberg, 1996
*Cases that were not included in the present review.
